# Revision of the western Palaearctic species of *Aleiodes* Wesmael (Hymenoptera, Braconidae, Rogadinae). Part 2: Revision of the *A. apicalis* group

**DOI:** 10.3897/zookeys.919.39642

**Published:** 2020-03-16

**Authors:** Cornelis van Achterberg, Mark R. Shaw, Donald L.J. Quicke

**Affiliations:** 1 State Key Laboratory of Rice Biology, Ministry of Agriculture Key Lab of Agricultural Biology of Crop Pathogens and Insects, and Institute of Insect Sciences, Zhejiang University, Hangzhou 310058, China Zhejiang University Hangzhou China; 2 Honorary Research Associate, National Museums of Scotland, Chambers Street, Edinburgh EH1 1JF, Scotland, UK National Museums of Scotland Edinburgh United Kingdom; 3 Department of Biology, Faculty of Life Sciences, Chulalongkorn University, Bangkok, Thailand Chulalongkorn University Bangkok Thailand

**Keywords:** *Aleiodes apicalis* group, key, new species, host range, biology, distribution, West Palaearctic, Europe, phenology

## Abstract

The West Palaearctic species of the *Aleiodes apicalis* group (Braconidae: Rogadinae) as defined by van Achterberg & Shaw (2016) are revised. Six new species of the genus *Aleiodes* Wesmael, 1838, are described and illustrated: *A. carbonaroides* van Achterberg & Shaw, **sp. nov.**, *A. coriaceus* van Achterberg & Shaw, **sp. nov.**, *A. improvisus* van Achterberg & Shaw, **sp. nov.**, *A. nigrifemur* van Achterberg & Shaw, **sp. nov.**, *A. turcicus* van Achterberg & Shaw, **sp. nov.**, and *A. zwakhalsi* van Achterberg & Shaw, **sp. nov.** An illustrated key to 42 species is included. Hyperstemma Shestakov, 1940, is retained as subgenus to accommodate A. chloroticus (Shestakov, 1940) and similar species. Fourteen new synonyms are proposed: *Rogas bicolor* Lucas, 1849 (not Spinola, 1808), *Rogas rufo-ater* Wollaston, 1858, *Rhogas bicolorinus* Fahringer, 1932, Rhogas reticulator var. atripes Costa, 1884, and *Rhogas similis* Szépligeti, 1903, of *Aleiodes apicalis* (Brullé, 1832); Rogas (Rogas) vicinus Papp, 1977, of *Aleiodes aterrimus* (Ratzeburg, 1852); *Rogas affinis* Herrich-Schäffer, 1838, of *Aleiodes cruentus* (Nees, 1834); *Bracon dimidiatus* Spinola, 1808, and Rhogas (Rhogas) dimidiatus var. turkestanicus Telenga, 1941, of *Aleiodes gasterator* (Jurine, 1807); *Rogas alpinus* Thomson, 1892, of *Aleiodes grassator* (Thunberg, 1822); *Rhogas jaroslawensis* Kokujev, 1898, of *Aleiodes periscelis* (Reinhard, 1863); Rhogas carbonarius var. giraudi Telenga, 1941, of *Aleiodes ruficornis* (Herrich-Schäffer, 1838); *Ichneumon ductor* Thunberg, 1822, of *Aleiodes unipunctator* (Thunberg, 1822); *Rogas heterostigma* Stelfox, 1953, of *Aleiodes pallidistigmus* (Telenga, 1941). Neotypes are designated for *Rogas affinis* Herrich-Schäffer, 1838; *Rogas nobilis* Haliday (in Curtis), 1834; *Rogas pallidicornis* Herrich-Schäffer, 1838; *Rogas ruficornis* Herrich-Schäffer, 1838. Lectotypes are designated for Rhogas (Rhogas) dimidiatus var. turkestanicus Telenga, 1941, and *Rhogas hemipterus* Marshall, 1897.

## Introduction

In this 2^nd^ part of a revision of western Palaearctic species of *Aleiodes* Wesmael we treat the group identified in Part 1 (van Achterberg and Shaw 2016) as the *A. apicalis* group. It should be noted that our *A. apicalis* group is constituted in a different (wider) way than of Shaw et al. (1998), Fortier and Shaw (1999), and Garro et al. (2017). The *Aleiodes apicalis* group as interpreted here is easily recognised from the key given by van Achterberg and Shaw (2016) and includes a majority of species that are rather large for the genus as a whole. From data presented in this paper, some of the commonest species, as well as being rather morphologically isolated, can now be said to be well-understood biologically and appear to be taxon-specialists (see Shaw 1994, 2017), which suggests to us that they have not been involved in recent speciation events (see Shaw 2003). However, for others, including a substantial number of rare, or at any rate rarely collected species, there is practically no biological information, with the result that this generalisation cannot be extended: indeed, there are some groups of apparently closely related species that are much more difficult to separate and in these parts of the *A. apicalis* group speciation has probably been more recent. We deal here with 42 species, a few of which are included only because they have been recorded from the region by others (i.e., the relevant specimens not examined by us) and/or are considered likely to occur in the eastern part of the area. Several of the most seldom-collected species occur as adults early in the year and may not in reality be as rare as they seem.

## Specimens, methods, and presentation of records

The biological data from rearings of wild-collected hosts is in some cases supplemented by experimentation, and the protocols and means of scoring results are as outlined in van Achterberg and Shaw (2016). The rather full introductory sections of that paper apply here and are not repeated except when not to do so would leave this paper difficult to use by itself. The term plurivoltine is used to indicate more than one generation in the year (very often this would be only two, but it could be more under favourable circumstances).

Overall, many of the species treated here have been widely misinterpreted in the literature and, as in Part 1 of our revision (van Achterberg and Shaw 2016), we have ignored published records when compiling host and distributional data, depending only on specimens we have actually seen ourselves. As previously, we have simply updated the nomenclature of hosts rather than transcribing obsolete names or obvious misspellings from data labels, and unless stated otherwise the reared material cited is in National Museum of Scotland, Edinburgh (NMS). From the host data we are able to give, indicating compact host ranges, in comparison with that expressed in Yu et al. (2016) it should be clear that this was a wise action. By similarly ignoring distribution data (e.g., from Yu et al. (2016)) we do not suggest that published distribution records are necessarily wrong: simply that we are unable to confirm them from the many thousands of specimens we have examined. The sheer number of these prevented us from listing specimen data in full, except for the few species of which we have seen only a very few specimens. The countries we list from the area under consideration (the western Palaearctic) are followed by a list of extralimital countries, in square brackets, from which we have also examined the species in question.

All available collections containing recently collected material of *Aleiodes* from the western Palaearctic region were used for our revision; collections with type material are separately listed under the description of the species. The following collections and acronyms are used:

**AAC** A.A. Allen Collection, Dawlish,

**ALC** A. Lozan Collection, Institute of Entomology, České Budĕjovice,

**ZJUH**Natural History Museum, London,

**BZL** Oberösterreichisches Landesmuseum, Biologiezentrum, Linz,

**CC** M. Čapek Collection, Moravian Museum, Brno,

**CMIM** C. Morley Collection, Ipswich Museum, Ipswich,

**CNC**Canadian National Collection of Insects, Ottawa,

**FC** J.V. Falcó Collection, Valencia,

**FMNH**Finnish Museum of Natural History, Helsinki,

**FRAH** Forest Research, Alice Holt Lodge, Farnham,

**HHC** H. Haraldseide Collection,

**HSC** H. Schnee Collection,

**IKC** I. Kakko Collection,

**KBIN** Koninklijk Belgisch Instituut voor Natuurwetenschappen, Brussels,

**JLC** J. Lukáš Collection, Bratislava,

**MCZ**Museum of Comparative Zoology, Harvard University, Cambridge, U.S.A.,

**MNHN**Muséum national d’Histoire naturelle, Paris,

**MMUM**Manchester Museum, University of Manchester, Manchester,

**MRC** M. Riedel Collection,

**MSC** M. Schwarz Collection, Linz,

**MSNV** Museo de Storia Naturale, Venice,

**MTMA**Hungarian Natural History Museum, Budapest,

**NMI**National Museum of Ireland, Dublin,

**NMS**National Museums of Scotland, Edinburgh,

**NNHM** National Natural History Museum, Oslo,

**NRS**Swedish Natural History Museum, Stockholm,

**OUM**Oxford University Museum of Natural History, Oxford,

**PAN**Museum and Institute of Zoology, Polish Academy of Sciences, Warsaw & Łomna-Las,

**RMNH**Naturalis Biodiversity Center, Leiden,

**SDEI** Senkenberg Deutches Entomologisches Institut, Müncheberg,

**SMNS**Staatliches Museum für Naturkunde, Stuttgart,

**SYKE** Finnish Environment Institute, Friendship Park Research Centre, Kuhmo,

**UMZC**University Museum of Zoology, Cambridge,

**UNS** Department of Biology and Ecology, University of Niš, Serbia,

**USNM**U.S. National Museum of Natural History, Washington D.C.,

**UWIM**University of Wyoming Insect Museum, Laramie,

**WAE** W.A. Ely Collection, Rotherham,

**ZIL**Zoological Institute, Lund University, Lund,

**ZISP**Zoological Institute, Academia NAUK, St. Petersburg,

**ZJUH**Zhejiang University, Hangzhou,

**ZMB**Zoologisches Museum, Humboldt Universität, Berlin,

**ZMC** Zoological Museum, Copenhagen,

**ZMUO**Zoological Museum, University of Oulu, Oulu,

**ZMUU**Zoological Museum of Uppsala University, Uppsala,

**ZSSM**Zoologische Staatssammlung, München; including E. Haeselbarth Collection.

In addition, we have examined specimens from various smaller and private collections, which are cited in significant cases. Unless otherwise specified, reared material is in NMS.

The number of antennal (i.e., flagellar + 2) segments is frequently an important aid to species recognition and of interest also because in some species the female has more segments on average than the male (males have a greater number in other species, which is the normal condition seen in Braconidae). We give counts of antennal segments for the specimens we have examined, but for some species (especially when the segments did not need to be counted for determination) sometimes only for the first hundred or so of the specimens examined of each sex.

Attention has been paid to the apical tergites of males. The medial dorsal pores of *A. fortipes* (Reinhard), which are unique to this species within the *A. apicalis* group as treated here, are described and discussed in the entry for that species (note that the unknown male of *A. caucasicus* (Tobias) is likely to be similar). In the remainder of the species group there is either no evident modification, or a different development is evident to a greater or lesser extent. In some species specialised setae are present on tergites 4–6(7), presumably connected with pheromone dispersal from tergal glands. Broadly, two kinds of specialised setae can occur on these tergites. First, a fringe of short backwards-projecting setae (hereafter “fringe”), possibly associated with pores, originate from close to an apparent sulcus near the extreme base of the tergite (which is normally concealed). The presence and nature of the fringe varies between species, and even when present, it may not be visible in a given specimen owing to telescoping of the tergites. Second, there may be backwards-directed and more or less dense patches of longer setae (hereafter “setal patches”) on each side of the mid-line, the setae to some extent being adpressed in their anterior part but tending to be raised posteriorly (in extreme cases giving the tergites a concave appearance) and appearing different from the arrangement of setae on the more anterior tergites. A median glabrous area is left between the paired setal patches on each tergite, which collectively present as a glabrous and often shiny dorsal stripe along the length of these tergites (hereafter “glabrous stripe”). There is considerable variation in the extent to which these features are developed in the species keyed here, and indeed in some species they are scarcely present or wholly absent. In the species accounts given below we attempt to give a score from 1 to 4 for the development of the setal patches and glabrous stripe in males, with minimal elaboration (but including also mention of the setal fringe in cases for which we have been able to observe it). Type 1 = not at all developed, setae as on anterior tergites and evenly distributed. Type 2 = setal patches hardly developed, but glabrous stripe evident to some extent. Type 3 = setal patches clearly developed but relatively weak or sparse, glabrous stripe strong. Type 4 = setal patches strongly developed, making the tergites appear concave, glabrous stripe also strong. It should be borne in mind that there is some intraspecific variation, much of which may be artefactual (i.e., the condition of the specimen may make it hard to assess and score accurately).

**Figures 1–6.**
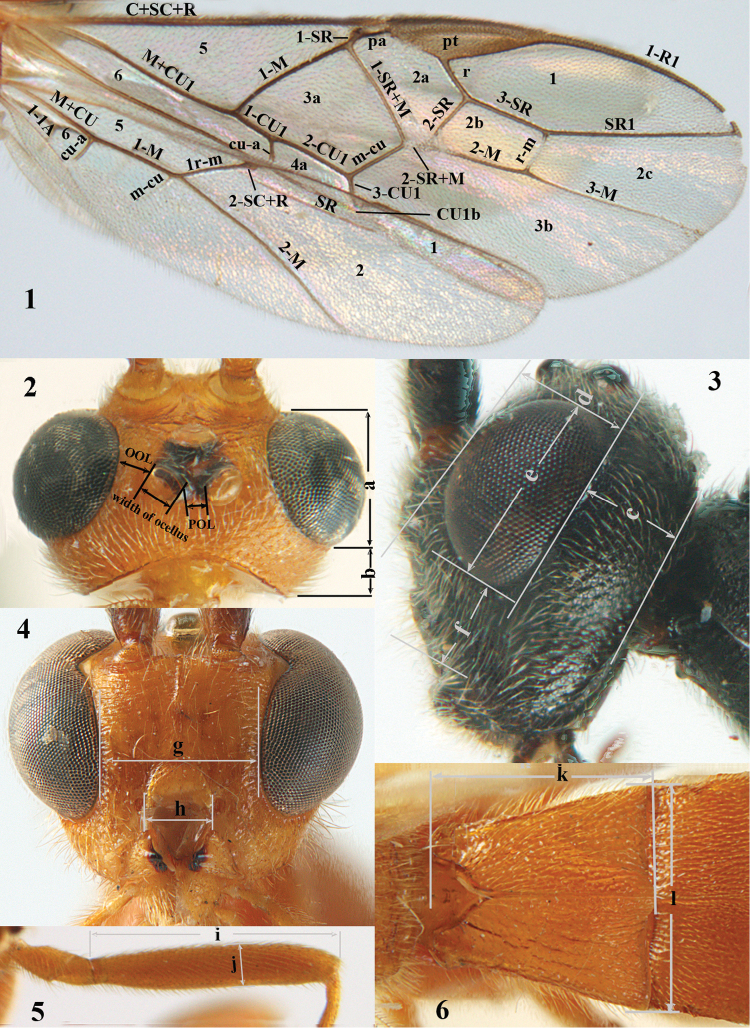
Terminology and measurements used in this paper **1** wing venation: pa = parastigma, pt = pterostigma, 1 = marginal cell, 2a, b, c = 1^st^, 2^nd^ and 3^rd^ submarginal cell, respectively, 3a, b = 1^st^ and 2^nd^ discal cell, respectively, 4a = 1^st^ subdiscal cell, 5 = basal cell, 6 = subbasal cell **2** head, dorsal aspect: a = length of eye, b = length of temple **3** head, lateral aspect: c = width of temple, d = width of eye, e = height of eye, f = width of malar space (measured as actual true distance in its own plane) **4** head, anterior aspect: g = width of face, h = width of hypoclypeal depression **5** fore femur, lateral aspect: i = length, j = width **6** 1^st^ metasomal tergite, dorsal aspect: k = length of tergite (measured from adductor), l = apical width of tergite.

For the recognition of braconid subfamilies, see van Achterberg (1990, 1993, 1997), for the identification of *Aleiodes* Wesmael, see van Achterberg (1991) and Chen and He (1997). For additional references see Yu et al. (2016). For the terminology used in this paper see Figs 1–6 or van Achterberg (1988, 1993; note, however, that in the present work the distance between eye and lateral ocellus is measured differently). An asterisk indicates a new country record according to Yu et al. (2016).

### Molecular methods

A molecular dataset of the barcode region of cytochrome oxidase c subunit 1 (CO1) was compiled for a total of 141 *Aleiodes* specimens and three of *Heterogamus* (Fig. 1) which are the sister group of *Aleiodes* (Zaldívar-Riverón et al. 2008, Quicke et al. unpublished) and were used for rooting the trees. Most of the recent DNA extractions were carried out using normal procedures for 96-well plates (Ivanova et al. 2006), and PCR and sequencing reactions were carried out using standard protocols (Hajibabaei et al. 2005). Most sequences were obtained using the LCO-HCO primer pair combination (Folmer et al. 1994: LCO 5’- GGT CAA CAA ATC ATA AAG ATA TTG G-3’, HCO 5’ - TAA ACT TCA GGG TGA CCA AAA AAT CA-3’) or, less often, LepF1-LepR1 (Smith et al. 2005: LepF1 5’-ATT CAA CCA ATC ATA AAG ATA TTG G-3’, LepR1 5’-TAA ACT TCT GGA TGT CCA AAA AAT CA-3’). Sequence alignment was carried out manually and was largely trivial as there was no length variation apart from a three base pair deletion uniting most species of the *A. risaae* Quicke and Butcher species group as previously noted (Butcher et al. 2012) and its precise location determined by reference to amino acid identities and the known codon positions. Sequences were analysed using maximum likelihood with the programme RAxML (v.8) (Stamatakis 2014), using a GTR + G rate model with three data partitions corresponding to the three codon positions. Each analysis comprised 100 replicates with two threads. Trees were visualised using Figtree (1.4.3) (Rambaut 2016).

GenBank accessions numbers are given in Appendix 1. Specimens with an identifier code comprising MRS followed by a number are deposited in NMS, with the exception of the paratype of *A. coriaceus* (MRS311) which is in RMNH. The specimens indicated CollHH with a number are retained in the personal collection of Håkon Haraldseide (Norway); voucher locations of samples prefixed by BCLDQ are as follows: for Thai specimens depositories are given in Butcher et al. (2012), USA specimens are in University of Wyoming collection; others and that of *A. mexicanus* (BMNHE897778) are in the collection of the Natural History Museum, London; the voucher of *A.* cameroniiJanz01 (DHJPAR0021064) is in the collection of Prof. Dan Janzen (Philadelphia); the voucher of *A. trianguliscleroma* (CCDB27844-E03) is in the collection of Tel Aviv University, Tel Aviv, Israel; the voucher of A. aff. wyomingensis (BIOUG01036-F12) is in the collection of the Center for Biodiversity Genomics, University of Guelph, Canada.

### Phylogeny

Three datasets were investigated with different levels of taxonomic and sequence inclusion.

**Figure 7.**
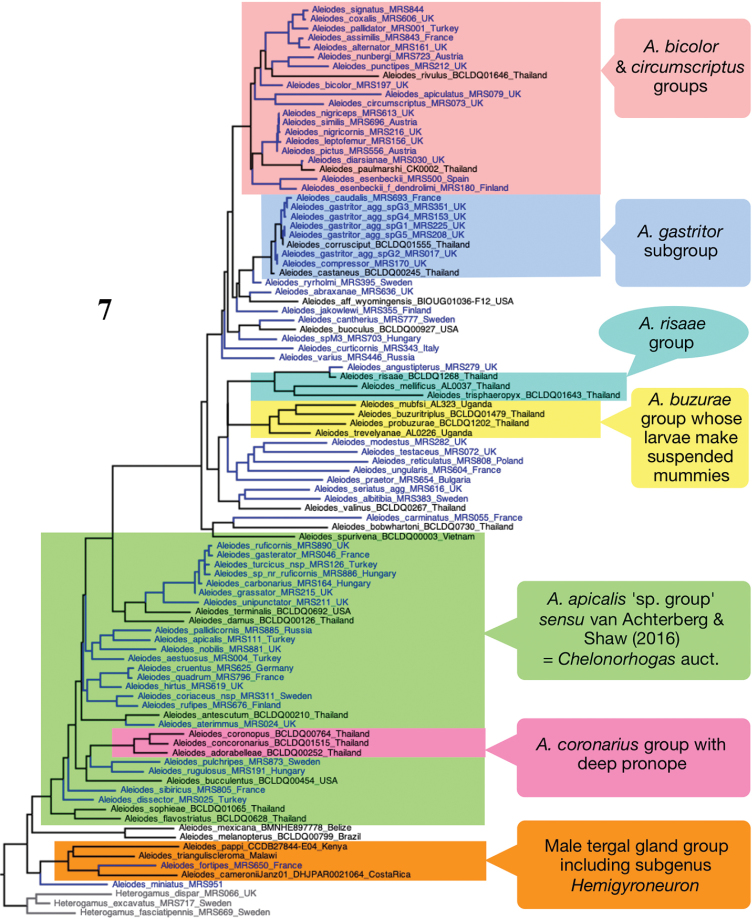
Maximum likelihood tree based on DNA barcode sequence data for representatives of taxa included in this paper (‘*Chelonorhogas*’ group) together with data from additional West Palaearctic and extra-limital species showing broad picture of relationships. Terminal text show specimen voucher code and provenance (when known).

Firstly, we conducted an overview analysis including representatives of a wide range of extra-limital species groups of *Aleiodes*, single representatives of the species treated in this paper for which molecular data were available (22 of the 42 species), and representatives of other West Palaearctic species groups, with three members of the genus *Heterogamus* used as outgroups (Fig. 7). In close agreement with the molecular tree presented for Thai *Aleiodes* (Butcher et al. 2012: fig. 5 loc. cit.), our results show that most of the *A. apicalis* species group *sensu*van Achterberg and Shaw (2016) (= *Chelonorhogas* auctt.) form a grade together with various generally large bodied extralimital species, notable among which are the Oriental and East Palaearctic *A. coronarius* group which are characterised by having a deep pronope. Immediately basal to this grade are two large bodied species (*A. melanopterus* (Erichson) and *A. mexicanus* Cresson) that had previously been included in a separate genus, *Eucystomastax* Brues, but which Shaw (1993) showed to be a distinctive monophyletic species group of Aleiodes within which he treated them as a subgenus. The sister to all other *Aleiodes* as recovered in this analysis are a group of species most members of which have males with metasomal tergal glands that open at a single medial subposterior pore on one or more of tergites four to six, although they are lacking, for example, in *A. miniatus*. This clade includes the West Palaearctic *A. fortipes* (Reinhard), the Nearctic *A. cameronii* (Dalla Torre) and some other species within the *A. pulchripes* group *sensu*Shaw et al. (1997), and the Palaeotropical subgenus Hemigyroneuron Baker (Shaw et al. 1997, Delfin and Wharton 2000, Butcher and Quicke 2011). From within this large basal grade emerges, on a relatively long branch, a monophyletic group which includes the vast majority of *Aleiodes* species. The West Palaearctic and Nearctic members of this clade have been placed in various species groups including the *A. bicolor*, *A. circumscriptus* and *A. gastritor* complexes. However, many species even within the Palaearctic fauna fall outside of these as isolated groups, often more closely related to extralimital taxa.

**Figure 8.**
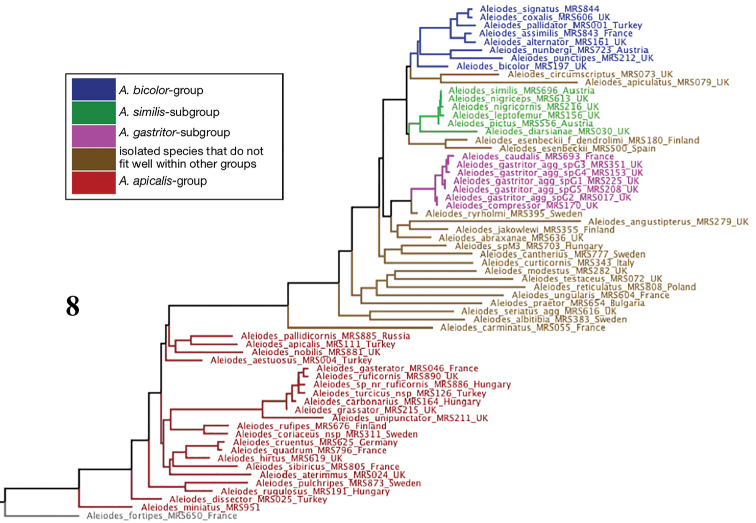
Maximum likelihood tree based on DNA barcode sequence data for taxa included in this paper (“*Chelonorhogas*” group – the dark red grade) together with a larger subset of West Palaearctic species from other species groups.

Secondly, we analysed a matrix comprising the most complete available sequence for each West Palaearctic species and using *A. fortipes* as the outgroup based on the results of the first analysis (Fig. 8). The *A. circumscriptus* group in the sense of van Achterberg and Shaw (2016) (including the *A. similis* and *A. gastritor* subgroups and the rather isolated *A. circumscriptus* (Nees) itself, as well as some other species) and the *A. bicolor* group *sensu*van Achterberg and Shaw (2016) were recovered together as a monophyletic clade but without strong indication of its comprising two separate groups. Indeed, the previous concept of the *A. circumscriptus* group was challenged by its paraphyly with respect to the *A. bicolor* group. The clade comprising the *A. bicolor* group and the *A. similis* subgroup (and a few extraneous species including *A. circumscriptus*) was not treated as a unit by van Achterberg and Shaw (2016) because of rather clear apparent differences: the clade indicated both in that work and here as the *A. bicolor* group comprises rather stocky and heavily sculptured species that have various morphological features (such as a long malar space and margined T4) in common and they, and several additional similar species, will be dealt with in Part 3 of this work. The species indicated as the *A. similis* subgroup and the *A. gastritor* subgroup will be treated together in Part 4. In practice, these two subgroups are less easy to separate on morphological grounds, and again there are many additional species. Throughout most of this large clade radiation seems to have been relatively recent and/or rapid with many morphologically and biologically clearly distinguishable species often having CO1 sequences that differ from one another by less than 1 %. The remainder of the species outside the *A. apicalis* grade are rather well characterised and indeed isolated, with little evidence of recent radiation, and the species for which host relations are known are taxon-specialists (cf. Shaw 2003). All of these species (with the exception of spM3) have already been treated in Part 1 of this work (van Achterberg and Shaw 2016).

**Figure 9.**
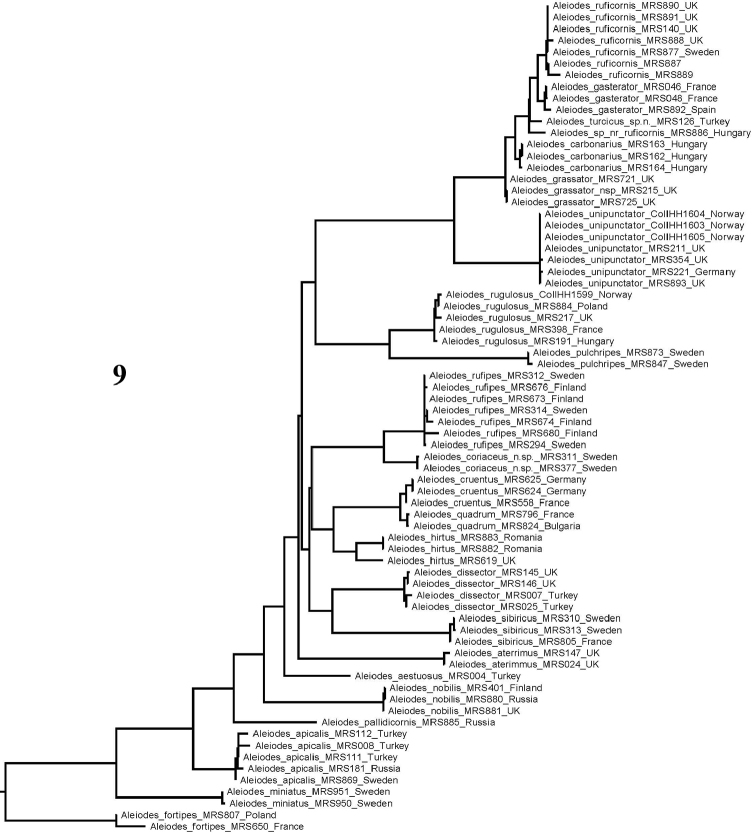
Maximum likelihood tree based on all DNA barcode sequence data for taxa included in this paper (“*Chelonorhogas*” group).

Thirdly, we constructed a tree for the available barcodes for the species treated in this paper (Fig. 9). Each species represented by multiple sequences is recovered as a monophyletic cluster, mostly with relatively little intraspecific variation. Some of the differences observed are likely due to particular sequences being quite short compared to the full-length barcode, others no doubt due to reading errors particularly for those samples that were sequenced more than ten years ago with different methodologies. Re-examination of existing electropherograms has usually confirmed the generality of this and such reading errors are typically at the 5’ or 3’ ends of sequence reads. The most notable exception is provided by the three specimens of *A. hirtus* (Thompson), with the sequence from the British specimen differing at 15 positions (2.5 %) along the 606-base region of overlap with the two Romanian individuals (which were identical). These specimens are briefly discussed in the species entry for *A. hirtus*.

## Taxonomy

### Aleiodes

Taxon classificationAnimaliaHymenopteraBraconidae

Wesmael, 1838

8EE21655-5B9C-5413-B4C9-D48B32191822

Figs 10–21 , 22–25 , 26–37 , 38, 39 , 40–49 , 50–53 , 54–65 , 66–71 , 72, 73 , 74–79 , 80 , 81–92 , 93–97 , 98–100 , 101 , 102–115 , 116–118 , 119–131 , 132–137 , 138–141 , 142–153 , 154–160 , 161–163 , 164–177 , 178, 179 , 180–191 , 192–195 , 196–198 , 199–211 , 212–216 , 217–220 , 221–233 , 234–239 , 240–242 , 243–255 , 256–263 , 264, 265 , 266–277 , 278–288 , 289–291 , 292–302 , 303–306 , 307–309 , 310–321 , 322–324 , 325–338 , 339–342 , 343–346 , 347–359 , 360–364 , 365–367 , 368–380 , 381–384 , 385–389 , 390–399 , 400–408 , 409, 410 , 411–424 , 425–427 , 428–431 , 432–444 , 445–452 , 453, 454 , 455–466 , 467, 468 , 469–481 , 482, 483 , 484–495 , 496 , 497–503 , 504, 505 , 506–518 , 519–521 , 522–535 , 536 , 537–542 , 543, 544 , 545–557 , 558, 559 , 560–572 , 573–576 , 577–589 , 590 , 591–603 , 604–607 , 608–621 , 622–626 , 627, 628 , 629–641 , 642 , 643–651 , 652, 653 , 654–666 , 667–669 , 670–681 , 682–685 , 686, 687 , 688–700 , 701–703 , 704–716 , 717, 718 , 719 , 720–727 , 728, 729 , 730–742 , 743 , 744–757 , 758, 759 , 760–762 , 763–776 , 777, 778 , 779–791 , 792–794 , 795–807 , 808–812 , 813, 814 , 815–826 , 827–829 , 830–840

Aleiodes Wesmael, 1838: 194; Shenefelt 1975: 1163–1185; Marsh 1979: 177–178; Papp 1985a: 143–164, 1985b: 347–349; Shaw and Huddleston 1991: 95–96 (biology); van Achterberg 1991: 24; Zaldívar-Riverón et al. 2004: 225, 2008: 329 (phylogeny); van Achterberg and Shaw 2016: 8–11 (groups). Type species (designated by Viereck 1914): Aleiodes heterogaster Wesmael, 1838 [examined; = A. albitibia (Herrich-Schäffer, 1838)].
Petalodes Wesmael, 1838: 123; Tobias 1971: 218 (transl. 1975: 86–87); Shenefelt 1975: 1209–1211; Tobias 1976: 90; Marsh 1979: 179; van Achterberg 1991: 24 (as synonym of Aleiodes Wesmael, 1838); van Achterberg and Shaw 2016: 8. Type species (by monotypy): Petalodes unicolor Wesmael, 1838 [examined; = Aleiodes compressor (Herrich-Schäffer, 1838)].
Schizoides Wesmael, 1838: 94. Unavailable name.
Nebartha Walker, 1860: 310; Shenefelt 1975: 1216; Marsh 1979: 179; van Achterberg 1991: 24 (as synonym of Aleiodes Wesmael, 1838). Type species (by monotypy): Nebartha macropodides Walker, 1860 [examined].
Tetrasphaeropyx Ashmead, 1889: 634; Shenefelt 1975: 1260; Marsh 1979: 179; Fortier and Sherman 2008: 445 (as subgenus of Aleiodes Wesmael, 1838); Zaldívar-Riverón et al. 2008: 329 (as synonym of subgenus Aleiodes Wesmael, 1838). Type species (by monotypy): Rogas pilosus Cresson, 1872 [examined].
Neorhogas Szépligeti, 1906: 605; Shenefelt 1975: 1205; van Achterberg 1991: 24 (as subgenus of Aleiodes Wesmael, 1838); Zaldívar-Riverón et al. 2008: 329 (included in subgenus Aleiodes Wesmael, 1838). Type species (by monotypy): Neorhogas luteus Szépligeti, 1906 [examined; = Aleiodes praetor (Reinhard, 1863)].
Chelonorhogas Enderlein, [Sept. 1^st^] 1912a: 258; Shenefelt 1975: 1187; van Achterberg 1991: 24 (as subgenus of Aleiodes Wesmael, 1838); Zaldívar-Riverón et al. 2008: 329 (as subgenus of Aleiodes Wesmael, 1838). Type species (by monotypy): Chelonorhogas rufithorax Enderlein, 1912 [examined; not Aleiodes rufithorax (Cameron, 1911) = A. convexus van Achterberg, 1991].
Eucystomastax Brues, [(end of?) Sept.] 1912: 223; Shaw 1993: 5 (as subgenus of Aleiodes Wesmael, 1838); Zaldívar-Riverón et al. 2004: 225 (included in Aleiodes Wesmael, 1838); Shimbori & Penteado-Dias 2011: 17 (as subgenus of Aleiodes Wesmael, 1838). Type species (by monotypy): Eucystomastax bicolor Brues, 1912 (= Rogas melanopterus Erichson, 1848).
Leluthinus Enderlein, 1912b: 96; Shenefelt 1975: 1202–1203; van Achterberg 1991: 24 (as synonym of Aleiodes Wesmael, 1838). Type species (by monotypy): Leluthinus lividus Enderlein, 1912 [examined].
Aleirhogas Baker, 1917b: 383, 411; Shenefelt 1975: 1185–1186; van Achterberg 1991: 24 (as synonym of Aleiodes Wesmael, 1838). Type species (designated by Viereck 1921): Rhogas (Aleirhogas) schultzei Baker, 1917 [examined].
Hemigyroneuron Baker, 1917a: 284, 322–327; Zaldívar-Riverón et al. 2008: 329 (as subgenus of Aleiodes Wesmael, 1838); Butcher and Quicke 2011: 1405 (as subgenus of Aleiodes Wesmael, 1838, and Hemigyroneuron*sensu*Zaldívar-Riverón et al. (2008) is not Hemigyroneuron)); Butcher and Quicke 2015: 275–279. Type species (original designation): Hemigyroneuron speciosus Baker, 1917 [examined].
Heterogamoides Fullaway, 1919: 43; Shenefelt 1975: 1188; van Achterberg 1991: 24 (as synonym of Aleiodes Wesmael, 1838). Type species (by monotypy): Heterogamoides muirii Fullaway, 1919 [examined].
Cordylorhogas Enderlein, 1920: 153; Shenefelt 1975: 1195; van Achterberg 1991: 31; Zaldívar-Riverón et al. 2004: 232, 2008: 329 (as synonym of subgenus Aleiodes Wesmael, 1838). Type species (by monotypy): Cordylorhogas trifasciatus Enderlein, 1920 [examined].
Hyperstemma Shestakov, 1940: 10; Shenefelt 1975: 1200; van Achterberg 1991: 24 (as synonym of Aleiodes Wesmael, 1838). Type species (by monotypy): Hyperstemma chlorotica Shestakov, 1940 [examined].
Dimorphomastax Shenefelt, 1979: 131–133; Shaw et al. 1998: 66 (as synonym of Aleiodes Wesmael, 1838). Type species (by original designation): Dimorphomastax peculiaris Shenefelt, 1979 [examined; = Aleiodes atriceps Cresson, 1869].
Pholichora van Achterberg, 1991: 48–53; Quicke and Shaw 2005: 532; Zaldívar-Riverón et al. 2008: 329 (as synonym of Aleiodes Wesmael, 1838); Butcher and Quicke 2011: 1405 (as synonym of subgenus Hemigyroneuron Baker, 1917); Butcher et al. 2012: 9 (id.). Type species (original designation): Hemigyroneuron madagascariensis Granger, 1949 [examined].
Arcaleiodes Chen & He, 1997: 60–62; Zaldívar-Riverón et al. 2008: 329 (as subgenus of Aleiodes Wesmael, 1838); Butcher et al. 2012: 18–19 (id.). Type species (original designation): Aleiodes unifasciatus Chen & He, 1991 [examined].
Vietorogas Long & van Achterberg, 2008: 313–314; Butcher et al. 2012: 15–17 (as synonym of Aleiodes Wesmael, 1838). Type species (original designation): Vietorogas bachma Long, 2008 [examined].
*R* (*h*)*ogas* auct; Tobias, 1971: 215–217 (transl. 1975: 83–86); Shenefelt, 1975: 1215–1256; Tobias, 1976: 81–89; Marsh, 1979: 179–181; Tobias, 1986: 74–84.

#### Notes.

**Figures 10–21.**
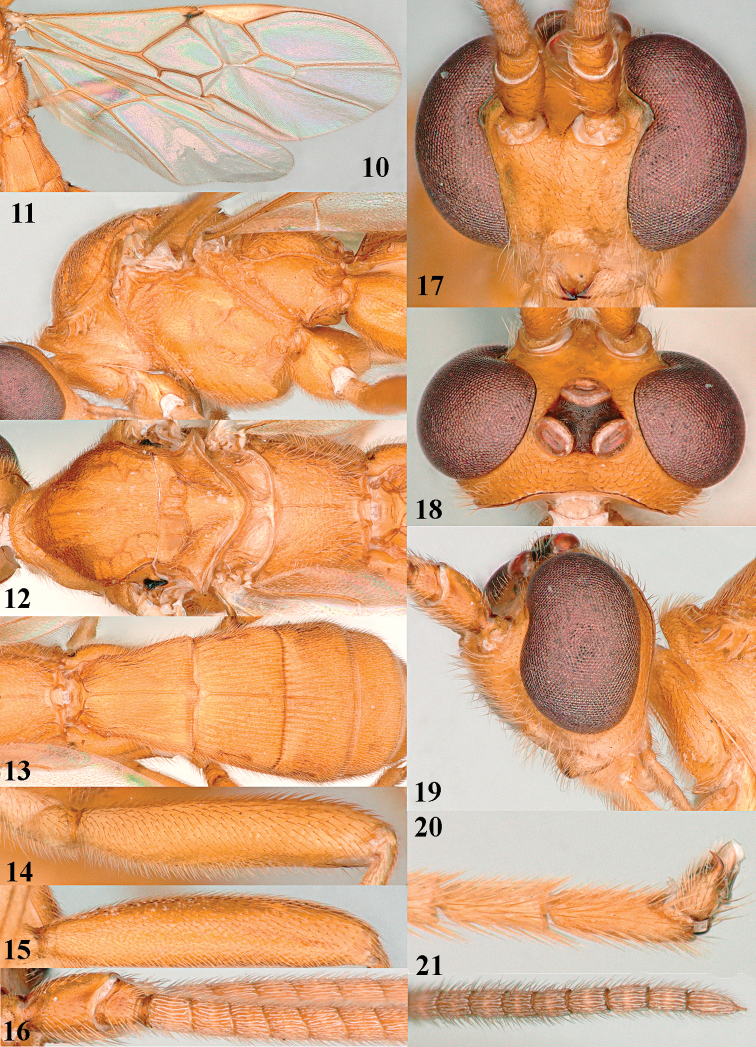
*Aleiodes chloroticus* (Shestakov), ♂, Japan **10** wings **11** mesosoma lateral **12** mesosoma dorsal **13** 1^st^–3^rd^ metasomal tergites dorsal **14** fore femur lateral **15** hind femur lateral **16** base of antenna **17** head anterior **18** head dorsal **19** head lateral **20** outer hind tarsal claw **21** apex of antenna.

*Hyperstemma* Shestakov, 1940, is traditionally included in the genus *Heterogamus* Wesmael, 1838 (Shenefelt 1975) or in the subgenus Heterogamus of the genus *Aleiodes* Wesmael (e.g., Belokobylskij 2000), but differs by the shape of the head (Figs 17–19) and of the tarsal claws (Fig. 20), the position of the clypeus (Fig. 17), the elongate 2^nd^ submarginal cell of the fore wing (but folded in Fig. 10), the widened 1^st^ subdiscal cell of the fore wing and distinctly widened marginal cell of the hind wing (Fig. 10). Therefore, we retain the subgenus Hyperstemma Shestakov of *Aleiodes* Wesmael for at least the following species: *A. chloroticus* (Shestakov, 1940) from China (Palaearctic and Oriental), *Japan (RMNH), Korea, and Russia (Far East), *A. albigenus* Chen & He, 1997, from China (Oriental) and Vietnam, *A. crassinervis* Chen & He, 1997, from China (Oriental) and Vietnam, *A. naevius* Chen & He, 1997, from China (Oriental), and *A. pallidinervis* (Cameron, 1910) from China (Palaearctic and Oriental), Japan, Korea, and Russia (Far East).

### Aleiodes apicalis

Taxon classificationAnimaliaHymenopteraBraconidae

group

D8E9E2A4-D2C7-5C97-B629-DB1DC2567902

#### Diagnosis.

Apical half of marginal cell of hind wing distinctly widened, its maximum width 1.6 × its width near hamuli or wider (Fig. 27) **and** vein r of fore wing shorter than vein 3-SR (Figs 180, 608), **if** marginal cell largely parallel-sided (Figs 506, 609, 704) then tarsal claws comparatively robust and with often blackish pecten (Figs 517, 621, 716) or brachypterous (Fig. 390); occipital carina usually reduced ventrally, not reaching hypostomal carina (Figs 600, 663, 713, 788); mesopleuron partly smooth (at least between punctures), but largely densely sculptured in both sexes of *A. hemipterus* and *A. krulikowskii*, as well in some males of *A. ruficornis* and allies; lateral carina of scutellum absent or if present then weakly developed and lunula wide (Fig. 508); 2^nd^ metasomal tergite with distinct and smooth triangular area medio-basally (Fig. 509); ovipositor sheath distinctly setose apically (Fig. 483); males are often darker than females, most extremely so in *A. arnoldii*, *A. carbonarius* and *A. carbonaroides*; brachypterous specimens of *Aleiodes* are included in this group.

#### Biology.

All species of the *A. apicalis* group for which host data exist are parasitoids of Noctuidae. However, the putatively more basal *A. fortipes* belonging to the *Hemigyroneuron* clade (see below) is a parasitoid of Geometridae. Also, only *A. fortipes* and *A. sibiricus* are known to parasitise hosts only in spring although these hosts would have been available in autumn of the previous year. Possibly others in the *A. apicalis* group will be found to do this too, and we consider the habit putatively as ancestral, in contrast with the more derived *A. circumscriptus* and *A. bicolor* groups in which species using hosts that overwinter as larvae invariably (as far as known) parasitise the host in the autumn and overwinter as a young larva inside it.

While we have no host data for a disappointingly large number of species of the *A. apicalis* group, the form of the clypeus may give important clues as to the site at which host mummification occurs, as those species in which mummification is known to take place in open situations (e.g., on a twig or in a leaf curl) invariably have a relatively small hypoclypeal depression and the clypeal margin blunt (*A. apicalis*, *A. aterrimus*, *A. fortipes*, *A. nobilis*, *A. pulchripes*, *A. rugulosus*) while species known to cause their hosts to mummify in concealed situations tend to have the hypoclypeal opening wider and the margin sharper (e.g., *A. cruentus*, *A. dissector*, *A. ruficornis*, *A. sibiricus*, *A. unipunctator*).

#### Notes.

According to the 28S + COI analysis by Zaldívar-Riverón et al. (2008) the following former subgenera or genera belong to this group: *Chelonorhogas* Enderlein, [1^st^ Sept.] 1912 (worldwide), *Eucystomastax* Brues, [(end of?) Sept.] 1912 (Neotropical group with 2^nd^ and 3^rd^ maxillary palp segments enlarged), *Hemigyroneuron* Baker, 1917 (Old World group with distal half of subbasal cell of fore wing modified and glabrous), and *Dimorphomastax* Shenefelt, 1979 (males of this monotypic Neotropical group have a large curved tooth near the base of the mandible (an outgrowth of the condylar carina) and the hind tibial spurs are blunt apically; females have the tooth smaller and triangular, and the hind tibial spurs are acute). Butcher et al. (2012) indicate in their cladogram based on the analysis of COI sequences that *A. fortipes* (Reinhard) forms together with *Hemigyroneuron* Baker and *Arcaleiodes* Chen & He the most basal clade of *Aleiodes* Wesmael, and it is noteworthy that all known hosts of this clade are Geometridae (see species entry for *A. fortipes*). According to the same analysis the *A. apicalis* group consists of three clades: (i) the *Hemigyroneuron* clade (see above; likely also includes *A. caucasicus* (Tobias)), (ii) the *A. rugulosus* clade (including the Asian group with modified pronotum), and (iii) the *A. gasterator* clade. Since we do not have the COI sequences of all species, we unite these three clades in the *A. apicalis* group to allow identification based on their morphology.

### Key to West Palaearctic species of the *Aleiodes apicalis* group

**Table:** 

1	Basal half of fore wing (except anteriorly) largely glabrous (a), or rather inconspicuously setose as remainder of wing; width of hypoclypeal depression 0.8–1.0 × minimum width of face (b) and anterior part of clypeus very narrow (c); vein r of fore wing 0.5–0.7 × vein 3-SR (d); mandibles massive triangular and coarsely punctate (e); [mandible with thick ventral lamella; antennal segments 47–63 and 4^th^ segment of ♀ 1.1–1.3 × longer than wide; head (except clypeus and mandible) and mesosoma (except partly prothorax and mesoscutum) black; tarsal claws slender and only setose]	**2**
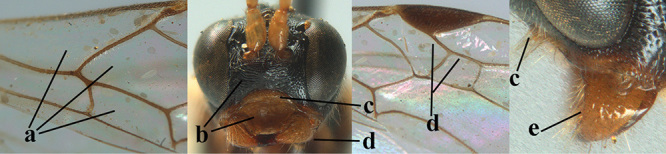		
–	Basal half of fore wing normally setose (except sometimes near veins) as remainder of wing (aa) or brachypterous (♀ *A. hemipterus*), **if** rarely with reduced setosity (*A. venustulus*) then width of hypoclypeal depression less than 0.7 × width of face (bb) and/or anterior part of clypeus moderately wide (cc), or vein r of fore wing 0.2–0.4 × vein 3-SR (dd); shape of mandible variable, often less massive and largely smooth (ee)	**3**
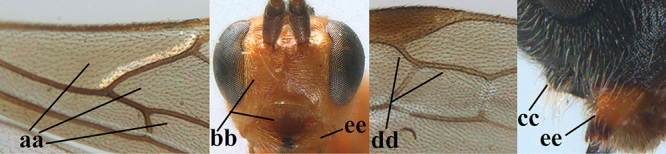		
2	Lateral lobes of mesoscutum whitish setose and with satin sheen (a); flagellum of antenna somewhat darker than scapus and pedicellus (b); middle lobe of mesoscutum distinctly punctate (c; more or less obscured by setosity); height of eye approx. 6 × length of malar space (d); basal half of metasoma dark brown, but laterally more or less yellowish (e)	***A. agilis* (Telenga, 1941)**
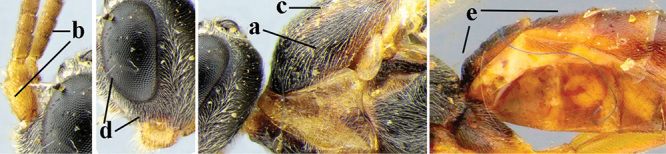		
–	Lateral lobes of mesoscutum largely glabrous or sparsely setose and strongly shiny (aa); flagellum of antenna brownish yellow, similar to colour of scapus and pedicellus (bb); middle lobe of mesoscutum largely smooth (cc); height of eye nearly 7 × length of malar space (dd); basal half of metasoma brownish yellow, at most 1^st^ tergite darker brown medio-basally (ee)	***A. desertus* (Telenga, 1941)**
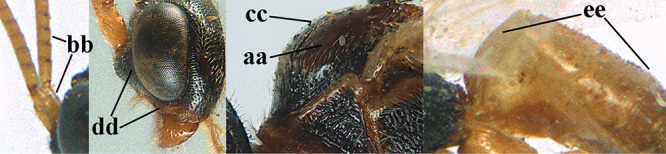		
3	Mesoscutum densely rugose or rugulose (a), with medio-longitudinal ridge or carina (b); mesopleuron mainly rugose (c); 3^rd^ metasomal tergite densely sculptured (d) and convex posteriorly (e); propodeum angulate posteriorly (f); ♀ brachypterous and ♂ macropterous; N Africa	***A. hemipterus* (Marshall, 1897)**
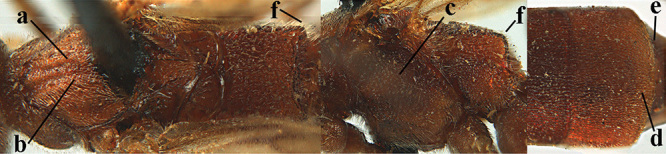		
–	Mesoscutum largely smooth and punctate or punctulate, mainly granulate or coriaceous (aa), usually without medio-longitudinal ridge or carina (bb); mesopleuron at most medially and antero-dorsally rugose (cc); 3^rd^ metasomal tergite truncate posteriorly or nearly so (dd) and/or largely smooth posteriorly (ee); **if** mesopleuron largely sculptured (ccc) combined with 3^rd^ tergite convex (ddd) and densely sculptured (eee) posteriorly, then propodeum rounded posteriorly (ff); both sexes macropterous	**4**
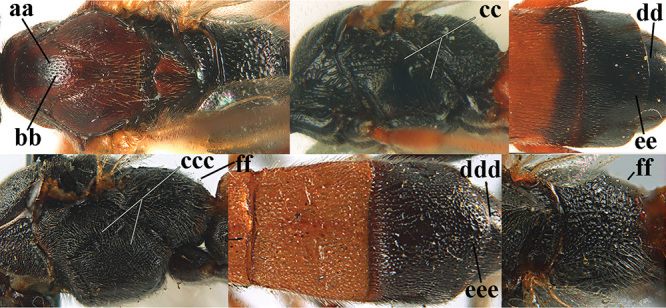		
4	Anterior part of clypeus short and subparallel-sided, near lower level of eyes (a) **and** hind femur slender (b); antenna with 65–72 segments and 5^th^–10^th^ segments approx. as wide as long (c); tarsal claws slender (d) and without distinct pecten (e); marginal cell of hind wing slightly constricted subbasally (f) or subparallel-sided (fff below); [temple behind eye densely setose, convex and curved in dorsal view; 2^nd^–5^th^ metasomal tergites more or less yellowish to reddish brown and head except mouthparts black]	***A. sibiricus* (Kokujev, 1903)**
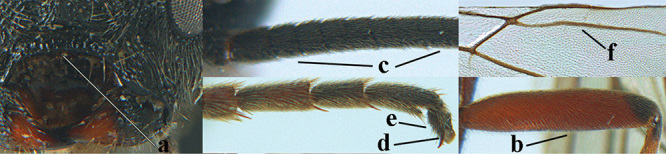		
–	Anterior part of clypeus medially distinctly wider than laterally (aa); **if** intermediate then partly above lower level of eyes or hind femur inflated (bb); antennal segments usually 62 or less, 4^th^–10^th^ segments variable, often longer than wide (cc); tarsal claws often rather robust (dd), **if** slender (ddd) then either with distinct pecten (ee) and/or marginal cell of hind wing directly widened subbasally (ff)	**5**
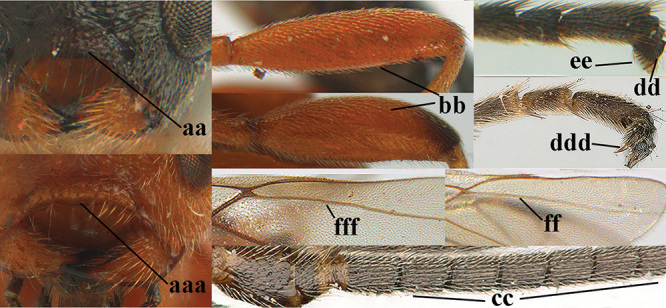		
5	Temples extremely short (a), approx. 0.2 × as long as eye in dorsal view; basal half of marginal cell of hind wing parallel-sided (b) **and** pterostigma pale-yellowish or light brown (c); malar space slightly shorter than basal width of mandible (d); hind tibial spurs of ♂ obtuse apically (e); [OOL distinctly less than diameter of posterior ocellus; tarsal claws with coarse pecten; mesopleuron, mesosternum and scutellum brownish yellow]	***A. pulchripes* Wesmael, 1838**
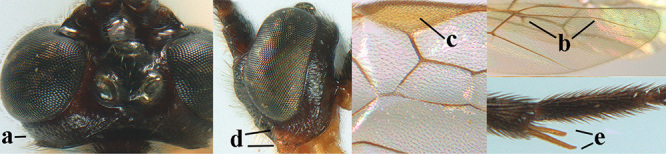		
–	Temples medium-sized to long (aa), at least 0.3 × as long as eye in dorsal view; basal half of marginal cell of hind wing gradually widened (bb), **if** parallel-sided (bbb) then pterostigma dark brown (cc); malar space usually as long as basal width of mandible (dd) or longer; hind tibial spurs of ♂ usually acute apically (ee)	**6**
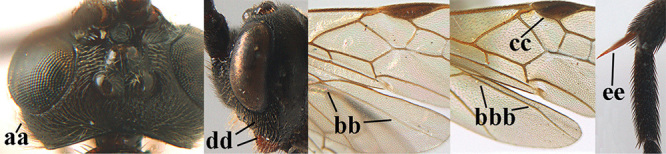		
6	Vein 2-SR+M of fore wing 0.8–1.0 × vein m-cu (a); pronotum and mesoscutum similarly coloured; clypeus width 0.3 × minimum width of face (b); length of fore wing 3.7–5.0 mm; length of hind femur 3.5–3.9 × its maximum width (c) **and** occipital carina reduced or anteriorly angled medio-dorsally (d); [4^th^–6^th^ metasomal tergites of ♂ with setose round pits (but ♂ of *A. caucasicus* unknown); vein m-cu of fore wing more or less subvertical and relatively short; 3^rd^–10^th^ antennal segments of ♀ pale yellowish, contrasting with entirely dark brown scapus]	**7**
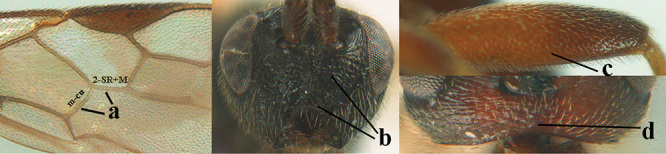		
–	Vein 2-SR+M of fore wing 0.2–0.6 × vein m-cu (aa), if more (some *A. nobilis*) then pronotum orange in contrast with blackish mesopleuron; clypeus width 0.4–0.8 × minimum width of face (bb); fore wing almost always longer than 4.9 mm; length of hind femur either more than 3.9 × its maximum width (cc) or occipital carina complete medio-dorsally (dd)	**8**
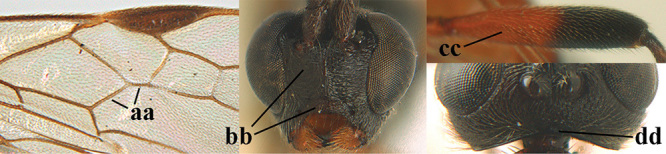		
7	Posterior half of mesosoma largely black or dark brown (a); precoxal area largely smooth, at most with some aciculae or punctures medially (b); tegulae brown (c); antero-dorsally mesopleuron coarsely rugose (d); [body of ♂ completely black and antenna completely blackish, dark brown or with some segments yellowish subbasally]; N & C Europe	***A. fortipes* (Reinhard, 1863)**
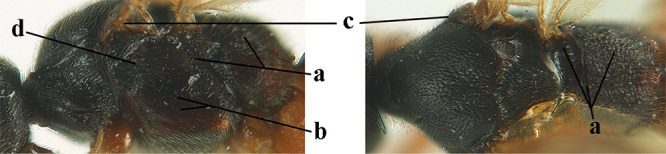		
–	Posterior half of mesosoma largely yellowish brown (aa); precoxal area more or less vertically striate (bb); tegulae usually yellow (cc), but sometimes dark brown; mesopleuron antero-dorsally moderately rugose (dd); SE Europe [♂ unknown]	***A. caucasicus* (Tobias, 1976)**
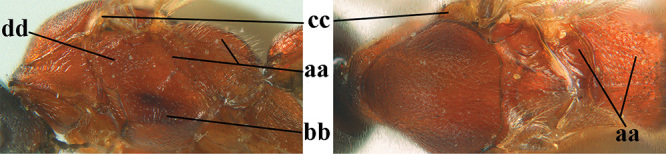		
8	Tarsal claws gradually narrowed submedially, slender and hardly curved (a) **and** 4^th^ hind tarsal segment brownish yellow and 1.8–2.0 × as long as wide (c); clypeus yellowish brown, distinctly protruding anteriorly and ventrally thick (b); tarsal segments ventrally with long apical spiny bristles (d); [4^th^ antennal segment of ♀ distinctly longer than wide; basal half of antenna and mesosoma anteriorly of ♀ largely yellowish brown, in ♂ more or less dark brown or infuscated; clypeus of ♂ yellowish and contrasting with black face]	***A. schewyrewi* (Kokujev, 1898)**
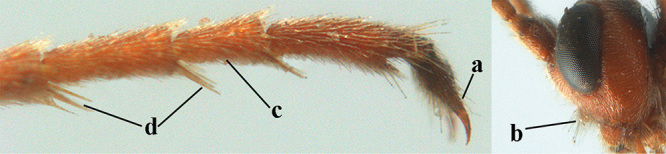		
–	Tarsal claws more directly narrowed submedially, moderately robust and apically curved (aa); **if** slender (ccc) and hardly curved (aaa) then clypeus black, with thin ventral margin (bb) **or** 4^th^ hind tarsal segment dark brown or infuscate and at most 1.5 × longer than its maximum width (cc) and tarsal segments ventrally with shorter apical bristles (dd)	**9**
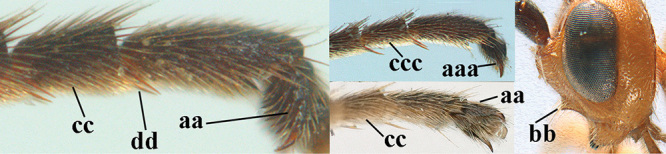		
9	Vein 1-CU1 of fore wing 0.7–1.5 × as long as vein m-cu (a); **if** 0.7–0.9 × (*A. aestuosus*, *A. zwakhalsi*) then base of hind tibia yellowish dorsally (b), hind trochanter orange or yellowish (c) and pecten up to apical tooth of tarsal claw (d)	**10**
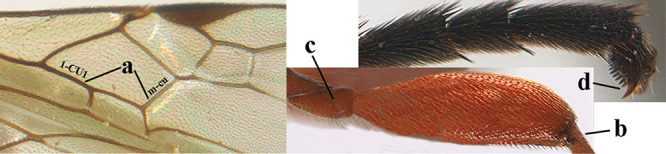		
–	Vein 1-CU1 of fore wing 0.3–0.8 × as long as vein m-cu (aa); **if** 0.7–0.8 × then base of hind tibia with dark brown patch dorsally (bb) or hind trochanter dark brown (cc) and in both cases pecten remaining removed from apical tooth of tarsal claw (dd)	**19**
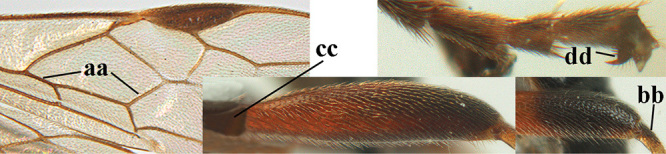		
10	Pronotum orange (except antero-medially), distinctly contrasting with black posterior half of mesosoma in lateral view (a, rarely black) **and** 3^rd^–6^th^ antennal segments of ♀ pale yellowish, contrasting with dorsally entirely dark brown scapus (b) **and** angle of vein m-cu of fore wing with vein 3-CU1 distinctly larger than 90° (c) **and** apex of hind femur black or dark brown (e); palpi yellow (d); [head black; hind basitarsus brownish yellow, contrasting with dark brown telotarsus]	**11**
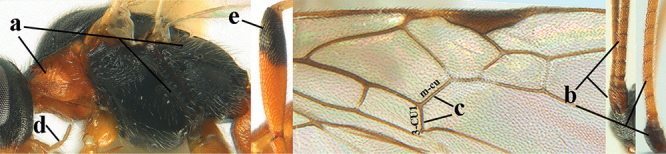		
–	Pronotum black or reddish brown and less contrasting with posterior half of mesosoma in lateral view (aa); **if** pronotum orange brown and contrasting with dark posterior parts (*A. venustulus*) then 3^rd^–6^th^ antennal segments of ♀ and scapus similarly dark brown (bb), angle of vein m-cu of fore wing with vein 3-CU1 closer to 90° (cc) and palpi dark brown (dd) **or** apex of hind femur yellowish or reddish brown (ee)	**12**
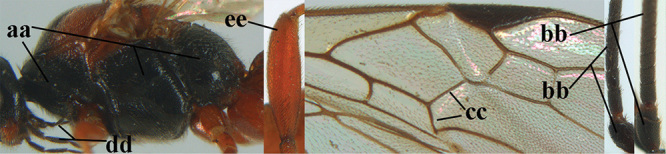		
11	Mesoscutum and scutellum black (a); temple rather mat and mainly granulate between punctulation (b); frons mat and strongly granulate (c); base of hind tibia pale yellowish (d); [precoxal area usually with some very superficial short rugulae or crenulae medially]	***A. nobilis* (Haliday, 1834)**
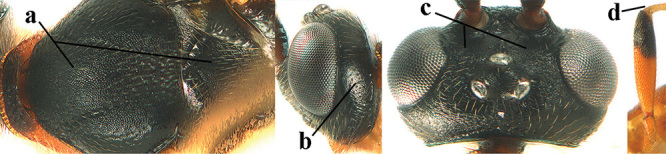		
–	Mesoscutum and scutellum orange brown (aa); temple shiny and smooth between punctures, striae or rugae (bb); frons shiny and with distinct striae or rugae (cc); base of hind tibia more or less infuscate (dd)	***A. schirjajewi* (Kokujev, 1898)**
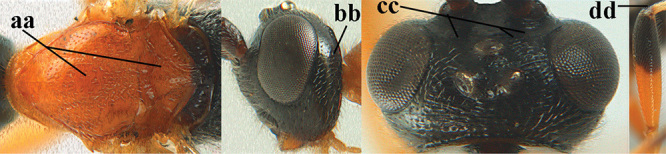		
12	Tarsal claws without pecten near apical tooth (a); vein m-cu of hind wing absent (b); wing membrane subhyaline to slightly infuscate (c); [5^th^–10^th^ antennal segments of ♀ distinctly longer than wide]	**13**
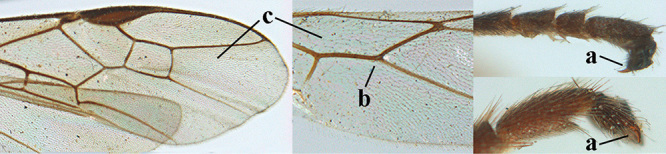		
–	Tarsal claws with pecten near apical tooth (aa); vein m-cu of hind wing at least weakly present (bb); wing membrane moderately infuscate or brownish (cc)	**14**
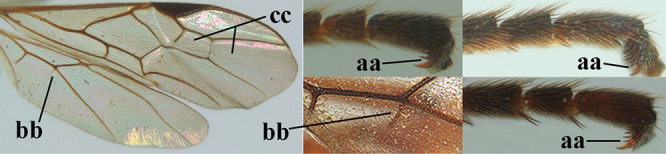		
13	Head of ♀ entirely yellowish brown or orange (a); ventral margin of clypeus thick and not protruding (b); vertex and OOL with smooth interspaces between punctures (c); mesopleuron remotely punctate and precoxal area coarsely punctate (d); 3^rd^ tergite nearly flat in lateral view (e) and medio-posteriorly nearly truncate in dorsal view (f); [fore femur elongate and hind femur 4.3–4.7 × longer than wide; vertex at least partly densely punctate]	***A. venustulus* (Kokujev, 1905)**
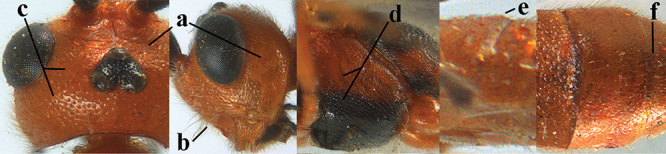		
–	Head of ♀ largely black (aa); ventral margin of clypeus thin and protruding anteriorly (bb); vertex and OOL without distinct smooth interspaces, rugose (cc); mesopleuron very densely and coarsely punctate and precoxal area rugose-punctate (dd); 3^rd^ tergite convex in lateral view (ee) and medio-posteriorly convex in dorsal view (f); [3^rd^ tergite coarsely punctate; fore and middle femora with dark patch]	***A. krulikowskii* (Kokujev, 1898)**
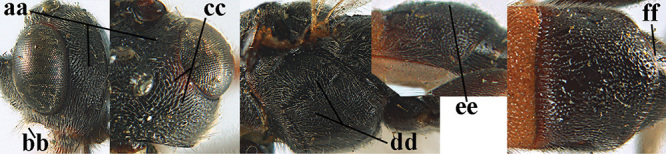		
14	Head of ♀ entirely yellowish brown or orange (a); ventral margin of clypeus thin and protruding anteriorly (b); eye 0.8–1.2 × temple in dorsal view (c); apical third of metasoma of ♀ completely yellowish (d; but ♂ often with 1^st^ tergite partly and 4^th^–6^th^ tergites blackish); hind femur of ♀ distinctly inflated (e), but sometimes less so; [antenna of ♀ with 49–56 segments; hind tibia of ♀ ivory except dark brown apex]	***A. aestuosus* (Reinhard, 1863)**
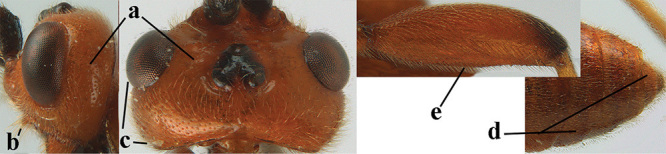		
–	Head of ♀ black (aa); ventral margin of clypeus thick and hardly protruding anteriorly (bb); eye 1.0–1.9 × temple in dorsal view (cc); apical third of metasoma of ♀ black (dd); hind femur of ♀ slender to moderately wide (ee)	**15**
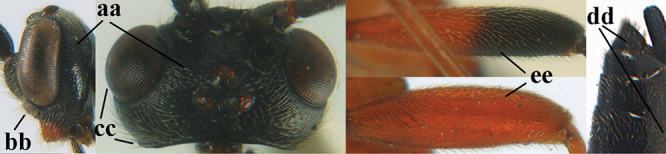		
15	Maximum width of hypoclypeal depression 0.3–0.4 × minimum width of face (a); 5^th^–10^th^ antennal segments of ♀ distinctly longer than wide (b); posterior half of mesoscutum black (c); 1^st^ metasomal tergite robust (d); [surroundings of veins M+CU1 and 1-+2-CU1 largely setose; vein M+CU of hind wing distinctly longer than vein 1-M; apical fifth of hind femur always blackish; 4^th^–6^th^ metasomal tergites of ♂ appearing concave and with conspicuous setosity]; C Europe, Mediterranean area, Central Asia. Examined specimens from S England (ZJUH), C Netherlands (RMNH) and S Sweden (NMS) are almost certainly passive migrants and do not represent breeding populations	***A. apicalis* (Brullé, 1832)**
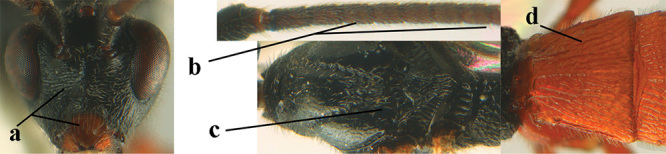		
–	Maximum width of hypoclypeal depression 0.5–0.7 × minimum width of face (aa); 5^th^–10^th^ antennal segments of ♀ approx. as long as wide (bb); posterior half of mesoscutum at least partly red (cc); 1^st^ metasomal tergite rather slender (dd); [surroundings of veins M+CU1 and 1-+2-CU1 largely glabrous; vein r of fore wing 0.3–0.4 × vein 3-SR]	**16**
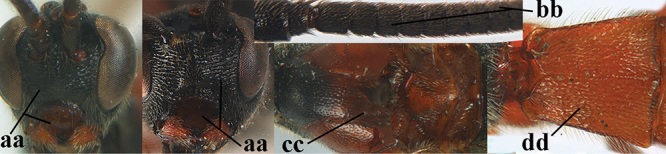		
16	Female: 2^nd^ metasomal tergite of ♀ as long as wide basally (a; of ♂ 0.9 ×); 1^st^ tergite only slightly widened posteriorly and 1.3–1.4 × as long as wide posteriorly (b; of ♂ 1.2 ×); 3^rd^ tergite largely smooth basally, only sparsely punctulate (c; of ♂ rugose); OOL distinctly rugose anteriorly (d); [hind femur ca 4.5 × longer than wide; ♂ may be easily confused with *A. cruentus*]	***A. quadrum* (Tobias, 1976)**
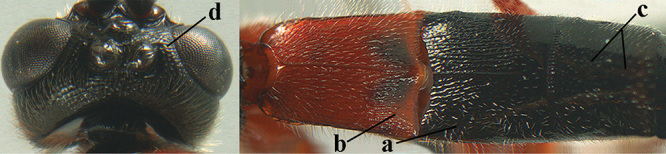		
–	Both sexes: 2^nd^ tergite of ♀ 0.7–0.9 × as long as wide basally (aa); 1^st^ tergite distinctly widened posteriorly and 1.0–1.1 × as long as wide posteriorly (bb); 3^rd^ tergite distinctly punctate or punctate-rugulose medio-basally (cc); OOL usually densely and coarsely punctate anteriorly (dd), rarely striate or rugose (ddd), but less sculptured in males and in *A. zwakhalsi*	**17**
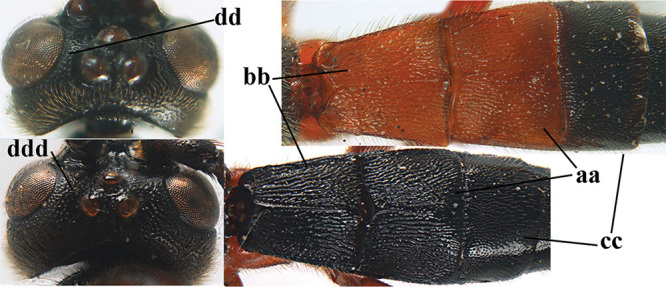		
17	Ocelli medium-sized to large (a), OOL of ♀ 0.5–0.8 × diameter of posterior ocellus, rarely up to 1.0 times; length of eye 1.5–1.9 × temple in dorsal view (b); 1^st^ and 2^nd^ metasomal tergites at least partly reddish or orange brown (c); [hypoclypeal depression usually 0.6–0.7 × width of face; hind femur 3.1–4.0 × longer than wide. If hind femur is 5 × longer than wide and hypoclypeal depression 0.5 × width of face, cf. *A. parvicauda* (Tobias, 1985) from Afghanistan]	***A. cruentus* (Nees, 1834)**
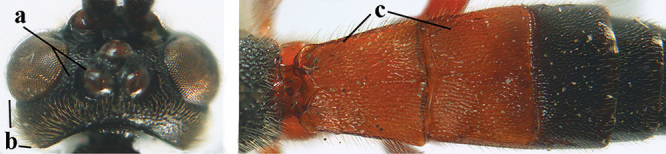		
–	Ocelli smaller (aa), OOL of ♀ 0.9–1.2 × diameter of posterior ocellus; length of eye 1.0–1.3 × temple in dorsal view (bb); 1^st^ and 2^nd^ metasomal tergites entirely black or dark brown (cc)	**18**
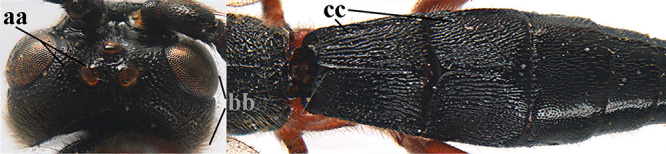		
18	Vein 1-CU1 of fore wing distinctly shorter than vein m-cu (a); hind femur 4.0–4.2 × longer than wide (b); vein cu-a inclivous and parallel with vein 3-CU1 (c); 5^th^–10^th^ antennal segments of ♀ as long as wide (d); vertex and OOL remotely punctate (e); width of hypoclypeal depression 0.7 × minimum width of face (f); [1^st^ metasomal tergite slender and rounded latero-basally; 3^rd^ tergite densely punctulate basally; metasoma of ♀ strongly compressed posteriorly; **if** body completely black, precoxal sulcus extensively rugose, pterostigma medially pale brown and OOL densely rugulose, cf. *A. morio* (Reinhard)]	***A. zwakhalsi* sp. nov.**
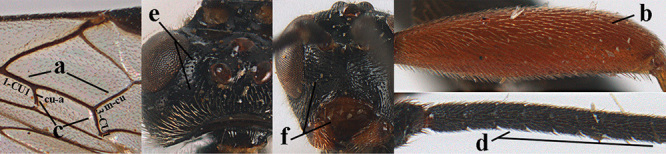		
–	Vein 1-CU1 of fore wing approx. as long as vein m-cu (aa); hind femur 3.0–3.3 × longer than wide (bb); vein cu-a vertical and vein 3-CU1 diverging posteriorly (cc); 5^th^–10^th^ antennal segments of ♀ shorter than wide (dd); vertex and OOL moderately to densely punctate (ee); width of hypoclypeal depression 0.5–0.7 × minimum width of face (ff); [metasoma of ♀ less compressed posteriorly; ovipositor sheath rather robust]	***A. diversus* (Szépligeti, 1903)**
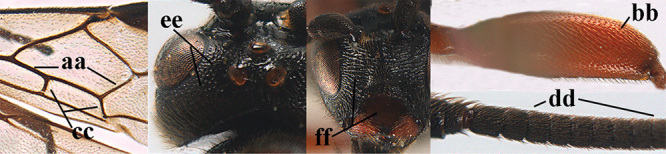		
19	Third metasomal tergite largely coarsely punctate and yellowish brown (a); 2^nd^ submarginal cell of fore wing short and square (b); medio-longitudinal carina at least in middle part of propodeum absent, obsolescent or incomplete (c); eye much narrower than temple in lateral view (d) **and** 4^th^–10^th^ antennal segments of ♀ distinctly longer than wide (e); [clypeus distinctly protruding in lateral view and ventrally thin (Fig. 478); vein 1r-m of hind wing much longer than vein 1-M; OOL twice as long as diameter of posterior ocellus; antennal segments of ♀ 64–70; 2^nd^ tergite coarsely reticulate-punctate; 4^th^–6^th^ metasomal tergites of ♂ flat and with longer (than of basal tergites) backwards directed setae with a narrow glabrous stripe centrally]	***A. miniatus* (Herrich-Schäffer, 1838)**
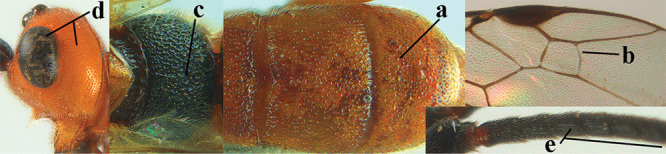		
–	Third tergite rugose, striate, rugulose or smooth, **if** punctate then black (aa); 2^nd^ submarginal cell longer than high (bb); medio-longitudinal carina of posterior half of propodeum complete or nearly so (cc); eye usually approx. as wide as temple in lateral view (dd), **if** distinctly narrower (ddd) then 4^th^–10^th^ antennal segments of ♀ approx.as long as wide (ee)	**20**
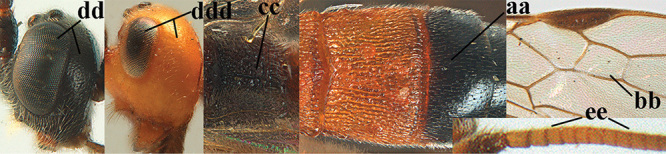		
20	Pecten of hind tarsal claws of ♀ robust (a), close to apical tooth (b) and often dark brown or blackish (c); [pecten of ♂ sometimes less developed than in ♀ (e.g., of *A. periscelis*) but then with some robust teeth medially (aaa)]	**21**
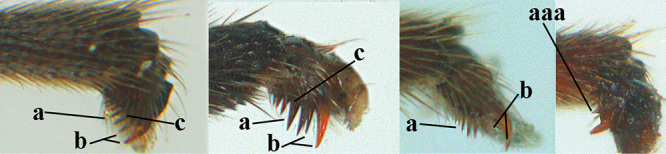		
–	Pecten of hind tarsal claws absent or inconspicuous (aa), **if** present then remaining removed from apical tooth (bb) and often yellowish or brownish (cc), but dark brown in *A. hirtus*	**26**
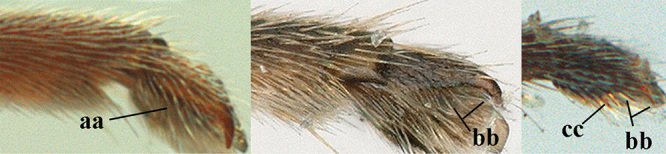		
21	Ventral margin of [anterior part of] clypeus comparatively sharp (a), clypeus more or less protruding anteriorly (b); palpi yellowish (c); vein 1-M of fore wing dark brown (d); basal half of metasoma weakly sculptured (e); hind femur largely or completely reddish or brownish (f); width of hypoclypeal depression 0.6–0.7 × minimum width of face (g); [precoxal area completely smooth or nearly so; length of malar space 0.2 × length of eye in lateral view; outer side of posterior ocellus with deep groove; vertex flattened. **If** palpi black or dark brown, temple with long setae, width of hypoclypeal depression 0.5 × minimum width of face, OOL more than diameter of posterior ocellus, 1^st^ tergite coarsely sculptured, and length of malar space 0.40–0.45 × length of eye in lateral view, cf. *A. hirtus* (Thomson)]	***A. dissector* (Nees, 1834)**
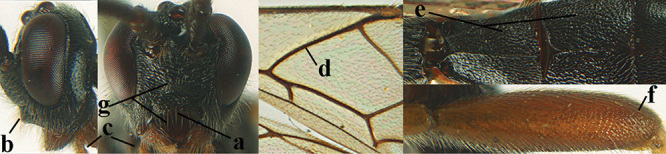		
–	Ventral margin of clypeus (rather) obtuse apically (aa) and clypeus hardly protruding anteriorly (bb); palpi dark brown at least basally (cc) **or** vein 1-M of fore wing yellowish brown (dd; *A. rugulosus*); basal half of metasoma distinctly sculptured (ee); hind femur dark brown or black dorso-apically (ff; but yellowish in *A. rugulosus*); width of hypoclypeal depression usually 0.3–0.4 × minimum width of face (gg)	**22**
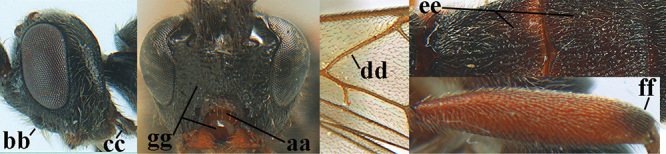		
22	Only apical two fifths of marginal cell of hind wing distinctly widened and remainder parallel-sided or nearly so (a), rarely hardly widened apically; vertex flattened behind ocelli (b) **and** apex of hind femur yellowish or reddish (c); first metasomal tergite with coarse sublongitudinal rugae (d); ovipositor sheath distinctly narrowed apically (e); [vein 1-M of fore wing yellowish brown; basal half of hind tibia pale yellowish/ivory or orange and its apical half black; mesopleuron nearly or completely smooth medio-ventrally; propodeum with pair of crest-like protuberances laterally]	***A. rugulosus* (Nees, 1811)**
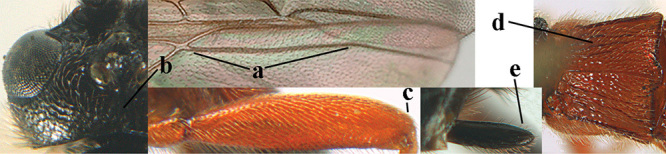		
–	At least apical half of marginal cell of hind wing gradually widened (aa); **if** less distinctly so, then vertex declivous behind ocelli (bb) and hind femur black or dark brown apically (cc); 1^st^ tergite moderately striate, rugulose or vermiculate-rugose (dd); ovipositor sheath truncate apically or nearly so (ee)	**23**
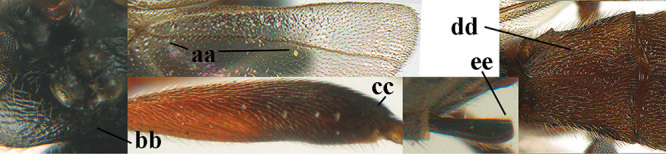		
23	Second metasomal tergite of both sexes black (a); scutellum densely and finely coriaceous (b); hind tibia largely black (c), dorsally paler at extreme base; 3^rd^ metasomal tergite of ♀ mainly punctate (d), but basal half more or less rugose in ♂; mesoscutum with satin sheen (e); [vein 2-SC+R of hind wing subquadrate or vertical; 4^th^–6^th^ tergites of ♂ with medium-sized dense setosity and with narrow glabrous central stripe; mesosternal sulcus shallow, obsolescent or absent. **If** hind tibia completely dark brown basally and temple roundly narrowed in dorsal view, cf. *A. sapporensis* (Watanabe) from East Palaearctic region]	***A. aterrimus* (Ratzeburg, 1852)**
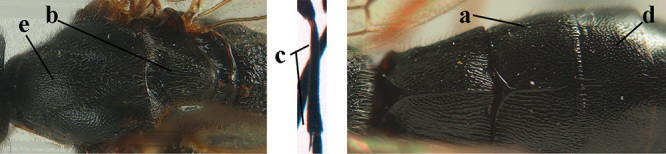		
–	Second tergite of ♀ yellowish or dark reddish brown (aa; up to almost black in ♂ of *A. periscelis*); scutellum partly smooth and punctate (bb); basal half of hind tibia (largely) pale yellowish or ivory (cc), rarely brownish; 3^rd^ tergite largely rugulose-striate basally (dd); mesoscutum rather shiny (ee)	**24**
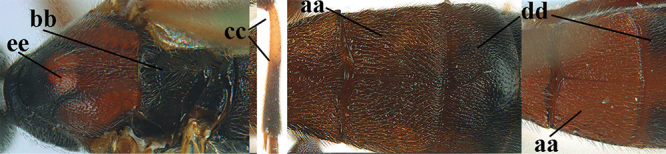		
24	Antennal segments of ♀ 39–45 (of ♂ 50–56) and subbasal segments of ♀ yellowish (a; of ♂ darkened but basal half of hind tibia ivory); fore femur of ♀ more robust (b); antenna of ♀ robust (c), 0.8–1.0 × longer than fore wing (= 0.7–0.8 × body length); fore coxa dark brown (d); vertex of ♀ coarsely rugose laterally (e); [mandible blackish basally; fore femur 4.8 × as long as wide]	***A. periscelis* (Reinhard, 1863)**
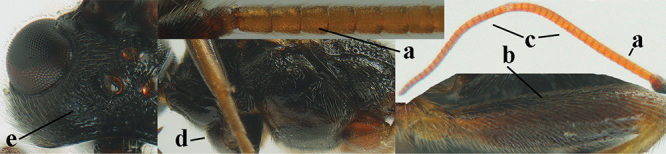		
–	Antennal segments of both sexes 52–62 and subbasal segments dark brown or blackish (aa); basal half of hind tibia of ♂ reddish to dark brown; fore femur of ♀ slenderer (bb); antenna of ♀ elongate (cc), 1.0–1.1 × longer than fore wing; fore coxa (brownish) orange (dd); vertex punctate-rugulose to coriaceous laterally (ee)	**25**
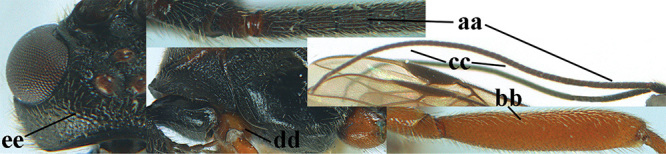		
25	Mesoscutum largely matt (a); base of fore femur, fore trochanter and trochantellus at least partly dark brown or infuscate (b); 2^nd^ and 3^rd^ metasomal tergites comparatively slender (maximum width of 2^nd^ tergite ca 1.5 × its median length; c); OOL of ♀ 0.9–1.1 × longer posterior ocellus (d); posterior half of hind femur blackish dorsally (e)	***A. coriaceus* sp. nov.**
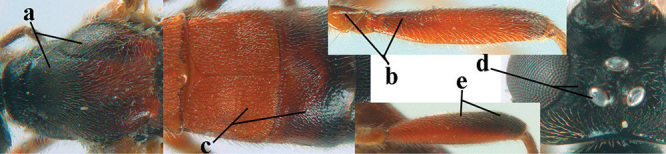		
–	Mesoscutum rather shiny (aa); base of fore femur, fore trochanter and trochantellus yellowish brown (bb); 2^nd^ and 3^rd^ metasomal tergites robust (maximum width of 2^nd^ tergite ca 1.6 × its median length; cc); OOL of ♀ 1.1–1.5 × longer posterior ocellus (dd); posterior half of hind femur partly yellowish brown dorsally (ee)	***A. rufipes*** (Thomson, 1892)
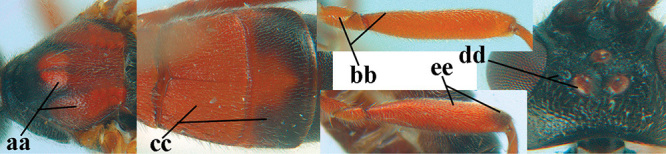		
26	Length of malar space of ♀ 0.45–0.70 × height of eye (a) and clypeus below lower level of eye in lateral view (b), **if** intermediate (in *A. ruficornis*) then basal antennal segments of ♀ very short (4^th^ segment approx. as long as wide; c); lateral lobes of mesoscutum mainly smooth, (rather) densely punctate, punctulate or rugose-punctate, interspaces (as far as present) between punctures usually largely smooth and shiny (d), but sometimes distinctly granulate; marginal cell of fore wing of ♀ usually robust and ending further removed from wing apex (e); [wing membrane more or less infuscate; precoxal area coarsely vermiculate-rugose medially; hind femur at least apico-dorsally dark brown or black; maximum width of hypoclypeal depression usually 0.3–0.4 × minimum width of face, if 0.5 × then ventral margin of clypeus thick; vein 1-R1 of fore wing 1.0–1.2 × length of pterostigma]	**27**
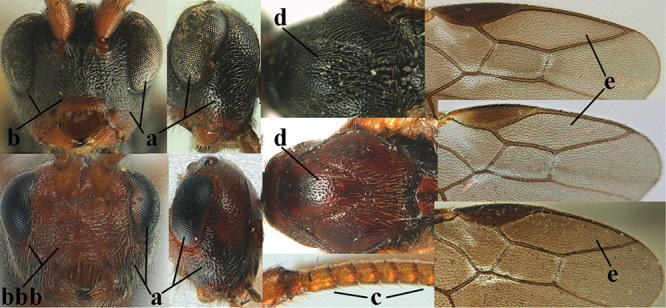		
–	Length of malar space of ♀ 0.20–0.45 × height of eye (aa) and clypeus near lower level of eye in lateral view (bb); basal antennal segments of ♀ usually moderately slender (with 4^th^ segment distinctly longer than wide; cc); lateral lobes of mesoscutum finely granulate, punctulate or moderately punctate, and often with a satin sheen (dd), but sometimes shiny (*A. hirtus*); marginal cell of fore wing of ♀ slender and ending closer to wing apex (ee), except in *A. morio* (eee); [wing membrane usually subhyaline; basal half of hind tibia largely pale yellowish or reddish, but less so in *A. morio*; vein 1-R1 of fore wing usually at least 1.4 × length of pterostigma, but approx. equal in *A. morio* (eee)]	**35**
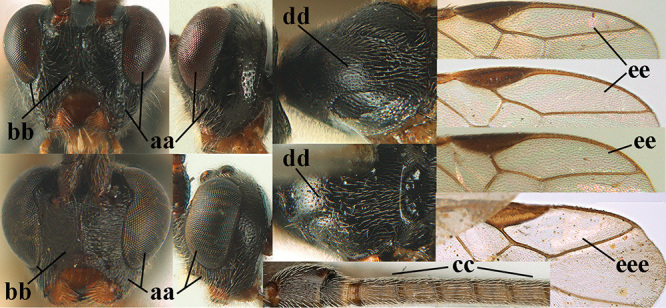		
27	Area between ocellus and eye, vertex and temple sparsely punctate (a); head of ♀ entirely brownish yellow (b; of ♂ variable, at least stemmaticum black); 1^st^ metasomal tergite 1.5–1.7 × wider posteriorly than subbasally (c); length of eye 1.0–1.2 ×temple in dorsal view (d)	**28**
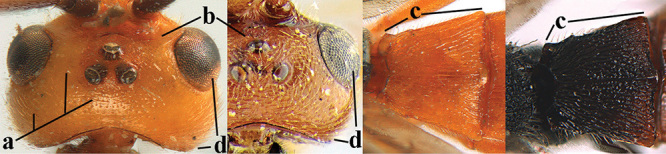		
–	Area between ocellus and eye, vertex and temple at least moderately densely punctate or rugose (aa); head of ♀ black (bb) or more or less dark red (bbb; of ♂ black); 1^st^ tergite 1.3–1.4 × wider posteriorly than subbasally (cc); length of eye 1.2–1.4 × temple in dorsal view (dd)	**29**
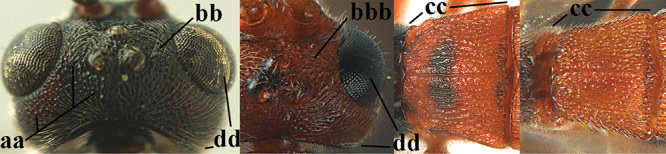		
28	Eye small (a) and in lateral view maximum width of temple 1.5–1.6 × maximum width of eye (b); ventral margin of clypeus thin and protruding anteriorly (c); antennal segments of ♀ 45–47 (of ♂ 56–58); mesoscutum of ♀ comparatively convex (d); [apex of hind tibia and basal part of palpi of ♀ dark brown]	***A. ruficeps* (Telenga, 1941)**
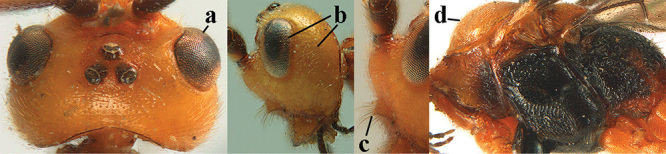		
–	Eye medium-sized (aa) and in lateral view temple hardly wider than eye (bb); ventral margin of clypeus thick and hardly protruding anteriorly (cc); antennal segments of ♀ 35–37; mesoscutum of ♀ less convex (dd); [apex of hind tibia and palpi of ♀ yellowish brown]	***A. arnoldii* (Tobias, 1976)**
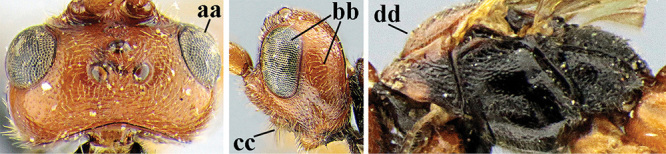		
29	Area between posterior ocellus and eye moderately punctate (a); posterior half of notauli shallow (b); head in anterior view rather trapezoid (c); [antenna of ♀ 1.1–1.2 × fore wing; 4^th^ antennal segment of ♀ moderately robust; pterostigma blackish; antennal segments of ♀ approx. 47]	***A. turcicus* sp. nov.**
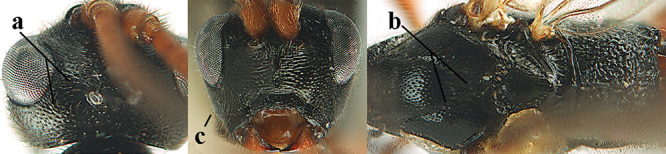		
–	Area between posterior ocellus and eye densely (and finely) rugose (aa), sometimes superficially so and rugulose or with some punctures; posterior half of notauli deep (bb); head in anterior view less trapezoid (cc); [antenna of ♀ with 30–47 segments; **if** antenna of ♀ with 54–64 segments, cf. *A. ferrugiteli* (Shenefelt, 1975) from C. Asia]	**30**
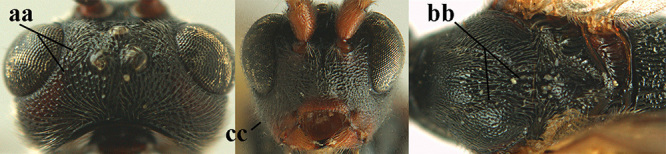		
30	Fore femur of ♀ subparallel-sided and 3.9–4.0 × longer than wide (a; this character is less reliable for ♂); antenna of ♀ 0.8–0.9 × fore wing (b); hypoclypeal depression usually slightly wider, 0.45–0.50 × minimum width of face (c); head of ♀ largely black (d), rarely face partly reddish; antennal segments of ♂ 36–46(–51) (usually 39–44); [tegulae usually (partly) dark brown; antennal segments of ♀ 29–41]	**31**
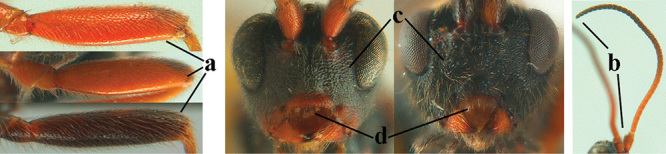		
–	Fore femur of ♀ inflated and 3.0–3.6 × longer than wide (aa); antenna of ♀ 0.9–1.2 × fore wing (bb); hypoclypeal depression usually narrower, ca 0.40 × minimum width of face (cc); head of ♀ at least partly reddish brown (dd); antennal segments of ♂ 47–63 (usually 48–54); [pale males have whole frons and stemmaticum yellowish; palpi dark brown or blackish, rarely brown; OOL of ♂ 1.5–2.0 × diameter of ocellus]	**32**
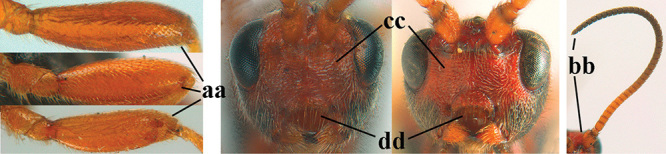		
31	Antennal segments of ♀ ca 41; subbasal antennal segments of ♀ dark brown and robust, with 4^th^ segment as long as wide (a); basal half of 3^rd^ tergite entirely coarsely striate (b; of ♂ sometimes with curved striae posteriorly); hind trochanter and trochantellus largely dark brown (c); inner and dorsal side of hind tibia apically dark brown (d); parastigma mostly brown (e); vein 1-CU1 of fore wing slightly longer than vein cu-a (f); [palpi dark brown, if largely ivory, cf. *A. periscelis*; 3^rd^ tergite only anteriorly reddish or yellowish; marginal cell of ♂ wide (Fig. 445); if slender, cf. *A. ruficornis*]; C. Europe (Alpine)	***A. improvisus* sp. nov.**
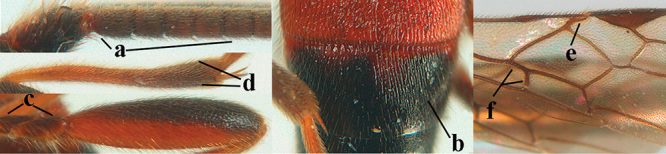		
–	Antennal segments of ♀ 29–39; subbasal antennal segments of ♀ yellow and comparatively slender, with 4^th^ segment ca 1.2 × as long as wide (aa); 3^rd^ tergite weakly sculptured, with (faint) curved or antero-medially transverse rugulae or striae (bb) or largely smooth (but sometimes with basal longitudinal striae laterally and often with distinct punctures laterally); hind trochanter and trochantellus yellowish or reddish brown (cc); inner and/or dorsal side of hind tibia (largely) yellowish or red apically (dd); parastigma mostly yellowish (ee); vein 1-CU1 distinctly longer than vein cu-a (ff); [palpi usually brownish or yellowish, but sometimes dark brown; pale males nearly always have frons medially and stemmaticum black; hind tibial spurs of male are usually blunt apically]; Mediterranean, C. Asia	***A. gasterator* (Jurine, 1807)**
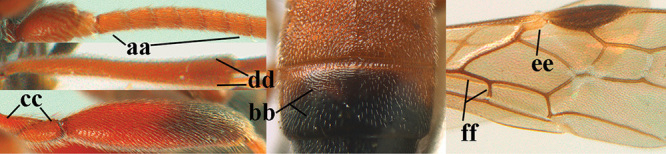		
32	OOL of ♀ approx. 2.6 × diameter of posterior ocellus (a); vein 3-SR of fore wing of ♀ 1.7–2.0 × vein 2-SR (b; of ♂ 1.2–1.5 ×); penultimate antennal segment of ♂ ca 1.2 × longer than wide (c); stemmaticum of ♀ usually black or dark brown (d), rarely reddish; telotarsi of ♀ dark brown (e); scapus of ♀ often black dorsally (f); [antenna of ♂ 0.9 × as long as body; inner side of hind tibia of ♀ dark brown apically]	***A. carbonarius* Giraud, 1857**
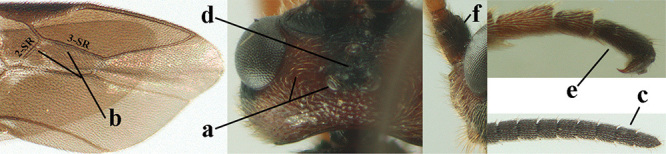		
–	OOL of ♀ 1.4–2.3 × diameter of posterior ocellus (aa); vein 3-SR of fore wing of ♀ 1.5–1.6 × vein 2-SR (bb; of ♂ 1.0–1.4 ×); penultimate antennal segment of ♂ approx. as long as wide (cc); stemmaticum of ♀ yellowish brown or reddish (dd); telotarsi of ♀ yellowish brown or reddish (ee); scapus of ♀ variable, brownish yellow dorsally (ff) to blackish	**33**
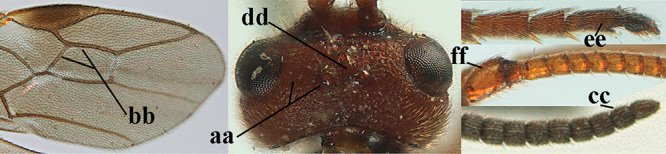		
33	Length of eye 1.5–2.0 × temple in dorsal view (a; if measured with posterior ocelli up to posterior level of eyes); OOL of ♀ 1.2–1.8 × diameter of posterior ocellus (b); subbasal antennal segments of ♀ slightly less moniliform (c); [inner side of hind tibia of ♀ usually dark brown or blackish apically; colour of legs of ♂ usually similar to legs of ♀ and usually partly yellowish; antenna of ♂ approx. as long as body and 1.2–1.4 × fore wing; antennal segments of ♀ 34–39(–47), of ♂ (44–)47–60]	***A. ruficornis* (Herrich-Schäffer, 1838)**
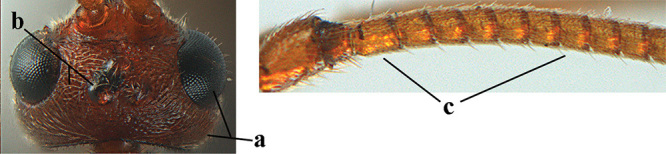		
–	Length of eye 1.1–1.5 × temple in dorsal view (aa; if measured with posterior ocelli up to posterior level of eyes); OOL of ♀ usually 1.9–2.3 × diameter of posterior ocellus (bb); subbasal antennal segments of ♀ distinctly submoniliform (cc); [inner side of hind tibia of ♀ yellowish; hind femur and basitarsus of both sexes more robust (but in ♂ sometimes rather slender); legs of males of W. European specimens strongly infuscate, darker than legs of females, but legs of N. European specimens paler; antenna of ♂ 0.8–0.9 × body and 1.0–1.1 × fore wing; antennal segments of ♀ 35–45(–46), of ♂ (44–)47–61]	**34**
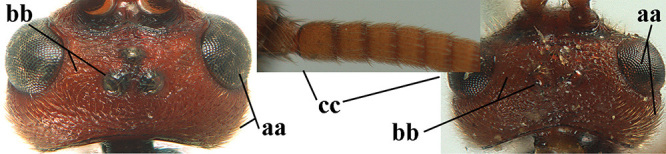		
34	Apical tooth of hind tarsal claws of ♀ robust (a); 2^nd^ metasomal tergite of ♂ orange brown (b); hind femur (c), tibia and basitarsus (d) of ♂ more robust and femur basally yellowish brown (e), if rarely almost black then hind tibia basally yellowish brown (f); clypeus less protruding in front of face (g); boreal and highland species	***A. grassator* (Thunberg, 1822)**
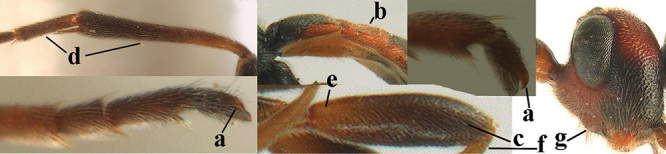		
–	Apical tooth of hind tarsal claws of ♀ slender (aa); 2^nd^ metasomal tergite of ♂ black (bb); hind femur (cc), tibia and basitarsus (dd) of ♂ comparatively slender and femur basally black (ee); hind tibia basally black (ff); clypeus more protruding in lateral view (gg); lowland species	***A. carbonaroides* sp. nov.**
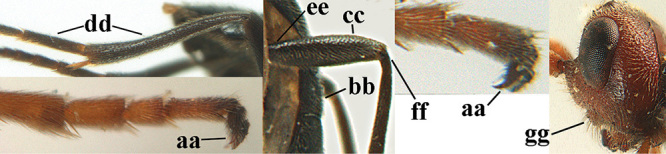		
35	Head brownish yellow (a); ventral margin of clypeus thin and distinctly protruding anteriorly (b); vertex (c) and mesoscutum (d) shiny; maximum width of hypoclypeal depression 0.6–0.7 × minimum width of face (e); pterostigma brownish yellow (f); [tarsal claws medium-sized and yellowish pectinate; body laterally and dorsally (except more or less dark brown propodeum and 1^st^ tergite) yellowish brown; occipital carina weakly indicated medio-dorsally]	***A. fahringeri* (Telenga, 1941)**
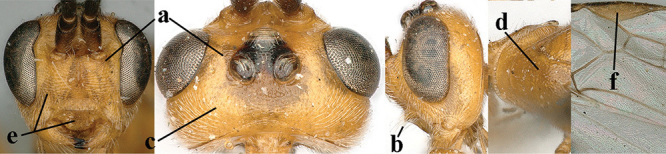		
–	Head black (aa); ventral margin of clypeus thick and usually hardly protruding (bb); vertex (cc) and mesoscutum (dd) usually rather dull and with satin sheen (cc); **if** shiny (ccc) then maximum width of hypoclypeal depression 0.5–0.6 × minimum width of face (ee) and pterostigma dark brown (ff); [pterostigma yellowish (fff) in *A. pallidistigmus*]	**36**
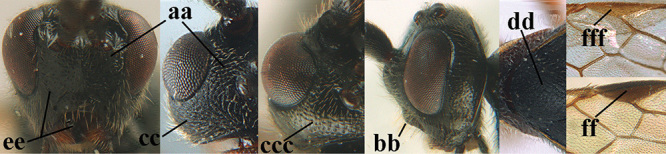		
36	Vertex (a) and mesoscutum (b) distinctly shiny because of smooth interspaces between punctures or rugae; head conspicuously setose because of long setae (c; less distinctive in ♀), as are propodeum and first tergite laterally; trochanters (and often also trochantelli) nearly always at least somewhat infuscate, darker than orange part of femora (d); subbasal antennal segments of ♀ dark brown; [palpi blackish or dark brown; tarsal claws with small pecten; 3^rd^–6^th^ antennal segments robust, hardly longer than wide; fore femur slender]	***A. hirtus* (Thomson, 1892)**
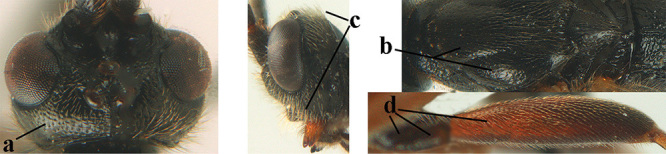		
–	Vertex (aa) and mesoscutum (bb) rather dull and with satin sheen, interspaces finely coriaceous-granulate between punctures or rugulae; head usually less conspicuously setose (cc); **if** sculpture and setosity are intermediate then trochanters and trochantelli have same colour as basal part of femora (dd) and subbasal antennal segments of ♀ brownish yellow	**37**
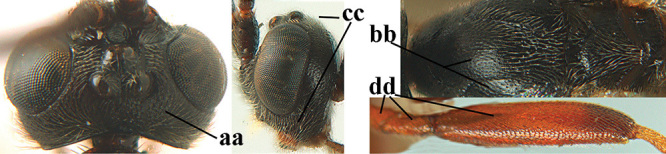		
37	Vein cu-a of fore wing at least as long as vein 1-CU1 (a); 1^st^ tergite of ♀ strongly widened apically (b); marginal cell of fore wing shorter (c); subbasal antennal segments of ♀ subquadrate (d); hind coxa (as femur) completely black (e); clypeus distinctly protruding anteriorly (f); fore femur largely dark brown (g); [OOL distinctly longer than diameter of posterior ocellus; 2^nd^ tergite finely and densely sculptured; **if** fore and hind femora yellowish brown, clypeus hardly protruding and marginal cell of fore wing elongate, cf. *A. sibiricus* (Kokujev)]	**38**
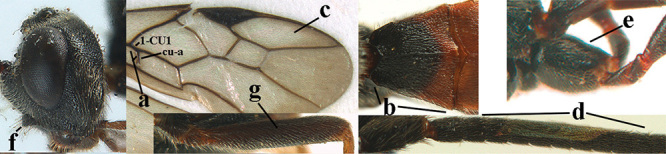		
–	Vein cu-a of fore wing distinctly shorter than vein 1-CU1 (aa); 1^st^ tergite moderately widened apically (bb); marginal cell of fore wing long (cc); subbasal antennal segments of ♀ longer than wide (dd); hind coxa orange or yellowish brown (ee); clypeus hardly or not protruding anteriorly (ff); fore femur brownish yellow (gg)	**39**
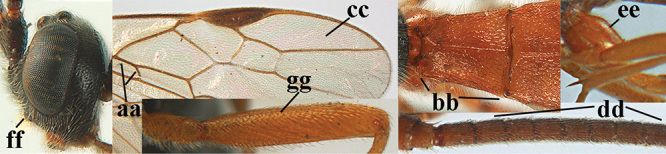		
38	Wings infuscate apically (a); 2^nd^ submarginal cell of fore wing less widened posteriorly (b); pterostigma medially dark brown (c); basal 0.4 of hind tibia yellowish (d); 2^nd^–5^th^ metasomal tergites orange brown (e); middle lobe of mesoscutum densely punctate, without distinct granulation in between punctures (f); [vein 2-1A of hind wing comparatively long; pecten of tarsal claws present and claws rather robust]	***A. nigrifemur* sp. nov.**
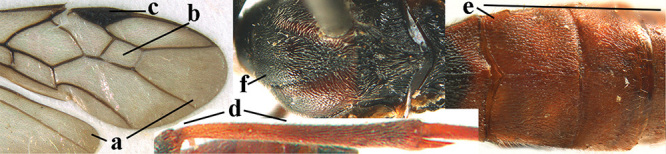		
–	Wings subhyaline apically (aa); 2^nd^ submarginal cell of fore wing widened posteriorly (bb); pterostigma medially pale or yellowish brown (cc); hind tibia mainly dark brown, only basally narrowly pale yellowish (dd); 2^nd^–5^th^ tergites black (ee); middle lobe of mesoscutum coriaceous (ff); [basal half of 3^rd^ tergite and OOL rugulose]	***A. morio* (Reinhard, 1863)**
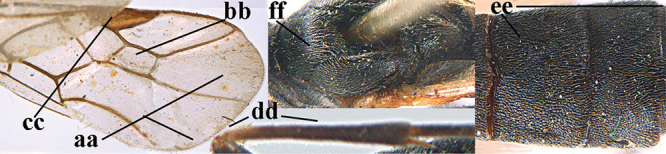		
39	Apex of hind tibia reddish or yellowish (a); pterostigma brownish yellow medially (b), rarely darkened; 3^rd^ tergite dull (c); [antenna of ♀ with 54–64 segments]	***A. pallidistigmus* (Telenga, 1941)**
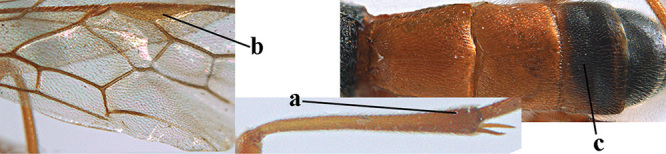		
–	Apex of hind tibia dark brown or infuscated (aa); pterostigma more or less dark brown medially (bb); 3^rd^ tergite usually shiny (cc), but sometimes rather dull (ccc)	**40**
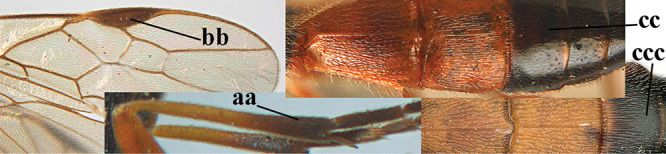		
40	Tarsal claws with medium-sized pecten (a); precoxal area of mesopleuron smooth medially, but sometimes with sparse weak punctures or some rugae below it (b); temple shiny (c); basal half of antenna of ♀ largely yellowish brown (d); [tegula and humeral plate equally yellowish orange; hind tarsus partly yellowish or brownish; hind tibia dorsally dark brown at extreme base, then pale subbasally and infuscate apically]	***A. pallidicornis* (Herrich-Schäffer, 1838)**
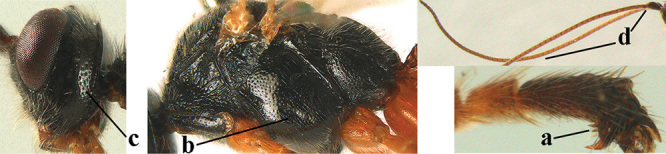		
–	Tarsal claws without pecten (aa) or with fine pale pecten; precoxal area of mesopleuron moderately rugose medially (bb); temple rather dull (cc); basal half of antenna blackish (dd)	**41**
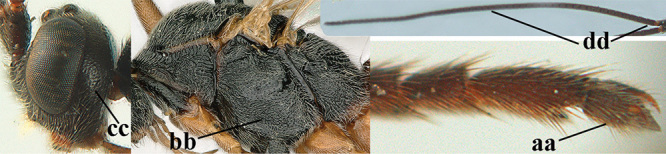		
41	Area between posterior ocellus and eye mainly granulate or coriaceous, at most with some punctures or rugulae (a); eyes larger in lateral view (b) and less protuberant in dorsal view (c); 3^rd^ metasomal tergite usually largely smooth, especially in ♀ (but basal half in ♂ sometimes extensively striate-rugulose) and as strongly glossy as following tergite (d); precoxal area comparatively narrow and posteriorly largely or completely smooth (e); antennal segments of ♀ 47–57(–58); hind femur largely reddish apically, with only slight infuscation (f); N & C Europe	***A. unipunctator* (Thunberg, 1822)**
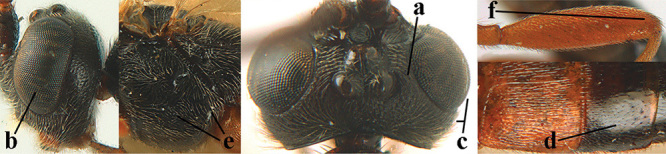		
–	Area between posterior ocellus and eye densely rugose, coarsely punctate or rugulose (aa); eyes smaller in lateral view (bb) and more protuberant in dorsal view (cc); basal half of 3^rd^ tergite distinctly striate or densely rugulose and less shiny (dd), but intermediates occur; precoxal area comparatively wide and usually posteriorly rugose or distinctly punctate (ee); antennal segments of ♀ 54–62; hind femur apically more or less smudged dark brown or black (ff), but sometimes very indistinct; [**if** mesoscutum rather steep anteriorly and width of hypoclypeal depression 0.4–0.5 × minimum width of face, and long malar space, cf. *A. gasterator* (Jurine) and related spp.]; S & E Palaearctic	***A. eurinus* (Telenga, 1941)**
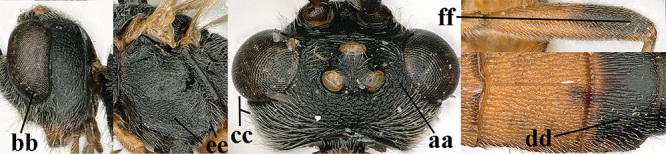		

### Biology and descriptions

#### Aleiodes aestuosus

Taxon classificationAnimaliaHymenopteraBraconidae

(Reinhard, 1863)

5C91497F-3402-56BB-9449-19348F7F57ED

Figs 22–25 , 26–37

Rogas aestuosus Reinhard, 1863: 265; Shenefelt 1975: 1216–1217; Zaykov 1980a: 112; Tobias 1976: 84, 1986: 78 (transl.: 129); Kotenko 1992: 96 [examined].
Rhogas aestuosus ; Fahringer 1931: 232–234.
Aleiodes (Neorhogas) aestuosus ; Papp 1985a: 152, 1989: 52, 1990: 90, 1991a: 67–68.
Aleiodes (Chelonorhogas) aestuosus ; Chen and He 1997: 38; He et al. 2000: 665; Belokobylskij 2000: 26; Chen et al. 2003: 211; Papp 2012: 187; Farahani et al. 2015: 238–240.
Aleiodes aestuosus ; Fortier and Shaw 1999: 230; Zaldívar-Riverón et al. 2004: 234.
Rhogas (Rhogas) aestuosus var. desertus Telenga, 1941: 152–153, 404 (not Rhogas (R.) desertus Telenga, 1941).

##### Type material.

Holotype, ♀ (MNHN), “Cipro [= **Cyprus**]”, “Muséum Paris, 1867, coll. O. Sichel”, “*Rogas aestuosus* Rhd.”.

##### Additional material.

Albania, Bulgaria, Cyprus, Greece, Russia, Turkey, Tunisia, [Azerbaijan, Georgia, Iran, Iraq, Israel, Jordan, Syria, Turkmenistan, Uzbekistan]. Specimens in ZJUH, BZL, CNC, HSC, MRC, MTMA, NMS, RMNH, ZSSM, ZISP. Distributed principally in Asia Minor, extending to Cyprus where it has been collected plentifully. Only single specimens examined from Albania (MTMA) and mainland Greece (BZL), but in North Africa it apparently extends westwards to Tunisia (one specimen in BZL).

##### Molecular data.

MRS004 (Turkey).

##### Biology.

Collected March–July, often at light, but it is not clear how many generations are represented nor how the winter is passed. Reared from Noctuidae: *Heliothis peltigera* (Denis & Schiffermüller) (4 [1 CNC/Iraq, 1 ZISP (with mummy)/Uzbekistan, 2 MTMA/Iraq]), *Sesamia* sp. (2 [ZJUH/Iran]). This indicates a host range of both endophagous and exophagous larvae, but the individuals purporting to be from *Sesamia* are labelled [no doubt incorrectly] “ex pupa” and lack mummies, suggesting that they may have resulted from substrate rearings (presumably from stems of crop species of Poaceae, inside which *Sesamia* larvae feed and pupate) rather than from isolated hosts, with a consequent reduction in the reliability of the host determination and suspicion that mummies of other hosts could have been overlooked on the stems (see also remark under *A. apicalis*). On the other hand, the large hypoclypeal depression and somewhat protruding clypeus does indicate that *A. aestuosus* adults are equipped to chew their way out of mummies made in concealed sites. The hosts given above are regular crop pests, but the paucity of reared material examined may suggest that *A. aestuosus* is not especially associated with cultivated habitats. The single mummy seen (Fig. 25) is rather elongate, scarcely arched, and the cocoon occupies most of the host abdomen. It has the appearance of not being securely stuck to the substrate.

##### Diagnosis.

Maximum width of hypoclypeal depression 0.6–0.7 × minimum width of face (Fig. 34); clypeus rather protruding anteriorly and rather thick ventrally (Fig. 36); head brownish yellow; vertex finely punctate; lateral lobes of mesoscutum sparsely and finely punctate, with wide smooth interspaces; precoxal sulcus absent, area only sparsely finely punctate or smooth; 1-CU1 of fore wing subequal to vein 2-CU1 (Fig. 26); hind tarsal claws with brownish pecten (Fig. 37); only apex of hind tibia dark brown; metasoma of ♀ completely yellowish and distinctly depressed subapically, 1^st^ tergite partly and 4^th^–6^th^ tergites of ♂ often blackish. Sometimes entire body (including propodeum and 1^st^ metasomal tergite) yellowish (“var. *desertus*”).

##### Description.

Redescribed ♀ (RMNH) from Turkey (Icil). Length of fore wing 6.8 mm, of body 8.3 mm.

***Head.*** Antennal segments of ♀ 52, length of antenna 1.1 × fore wing, its subapical segments approx. as long as wide; frons with irregular curved rugae, shiny, and rugose behind antennal sockets; OOL 2.4 × diameter of posterior ocellus, and finely remotely punctate, interspaces much larger than diameter of punctures; vertex spaced punctate, shiny; clypeus short, coarsely and densely punctate; ventral margin of clypeus thick and rather protruding forwards (Fig. 36); width of hypoclypeal depression 0.65 × minimum width of face (Fig. 34); length of eye 0.8 × temple in dorsal view (Fig. 35); vertex behind stemmaticum sparsely punctate; clypeus near lower level of eyes; length of malar space 0.3 × length of eye in lateral view.

***Mesosoma.*** Mesoscutal lobes largely smooth, shiny, sparsely and finely punctate; prepectal carina medium-sized, reaching anterior border; precoxal area of mesopleuron and metapleuron remotely punctate, interspaces much wider than diameter of punctures, shiny; mesopleuron above precoxal area (except speculum) sparsely punctate; scutellum slightly convex, remotely punctate and evenly rounded laterally, no carina; propodeum evenly convex and coarsely rugose, medio-longitudinal carina complete, but irregular posteriorly, without tubercles.

***Wings.*** Fore wing: r 0.4 × 3-SR (Fig. 26); 1-CU1 horizontal, nearly as long as (0.9 x) 2-CU1; r-m 0.9 × 2-SR, and 0.7 × 3-SR; second submarginal cell medium-sized (Fig. 26); cu-a vertical, not parallel with CU1b, straight; 1-M rather curved posteriorly. Hind wing: marginal cell gradually and evenly widened, its apical width 2.3 × width at level of hamuli (Fig. 27); 2-SC+R shortly longitudinal; m-cu distinct; M+CU:1-M = 23:19; 1r-m 0.7 × 1-M.

***Legs.*** Tarsal claws subpectinate, with four brown medium-sized pectinal bristles (Fig. 37); hind coxa remotely punctate, shiny; hind trochantellus robust; length of hind femur and basitarsus 3.0 and 3.5 × their width, respectively; length of inner hind spur 0.55 × hind basitarsus; hind tibia slender (Fig. 23).

**Figures 22–25.**
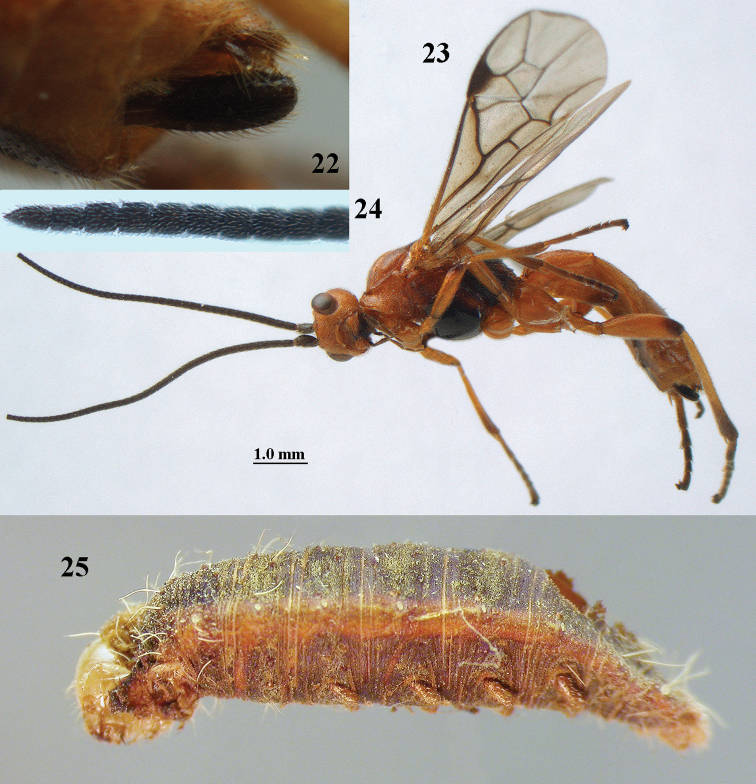
*Aleiodes aestuosus* (Reinhard), ♀, Cyprus, Yermasoyja River, but 25 from Uzbekistan, Qamashi **22** ovipositor sheath lateral **23** habitus lateral **24** apex of antenna **25** mummy of *Heliothis peltigera* (Denis & Schiffermüller).

***Metasoma.*** First tergite rather flattened, as long as wide apically; 1^st^ and 2^nd^ tergites coarsely and densely rugose, robust, with distinct median carina; medio-basal area of 2^nd^ tergite wide and short; 2^nd^ suture deep medially and shallow laterally; basal half of 3^rd^ tergite finely rugose, remainder of metasoma largely smooth, punctulate; 4^th^ and apical half of 3^rd^ tergite without sharp lateral crease; ovipositor sheath with medium-sized setae and apically rounded (Fig. 22).

***Colour.*** Brownish yellow; antenna, mesosternum (except anteriorly) and mesopleuron (except anteriorly and dorsally), metapleuron, propodeum, ovipositor sheath and stemmaticum black; hind tibia (except apically) pale yellowish; apices of femora (dorsally) and tibiae, palpi, tarsi (except basally), veins and pterostigma dark brown; wing membrane rather infuscate.

***Variation.*** Size of eyes and ocelli rather variable. Mesopleuron, mesosternum, metapleuron and propodeum brownish yellow or black; 1^st^ tergite entirely brownish yellow or with dark brown patch basally; in desert areas body can be wholly orange. Antennal segments: ♀ 49 (1), 50 (3), 51 (9), 52 (13), 53 (10), 54 (3), 55 (5), 56 (2); ♂ 51 (10), 52 (11), 53 (5), 54 (4), 55 (3), 56 (1). The two sexes have comparable numbers of antennal segments. Apical tergites of ♂ type 3 and fringe moderately strong; inner hind tibial spur 0.50 × as long as hind basitarsus.

##### Distribution.

Albania, Azerbaijan, Bulgaria, Cyprus, Georgia, *Greece, Iran, *Iraq, Israel, *Jordan, Russia, Syria, Turkey, Tunisia, *Turkmenistan, Uzbekistan.

**Figures 26–37.**
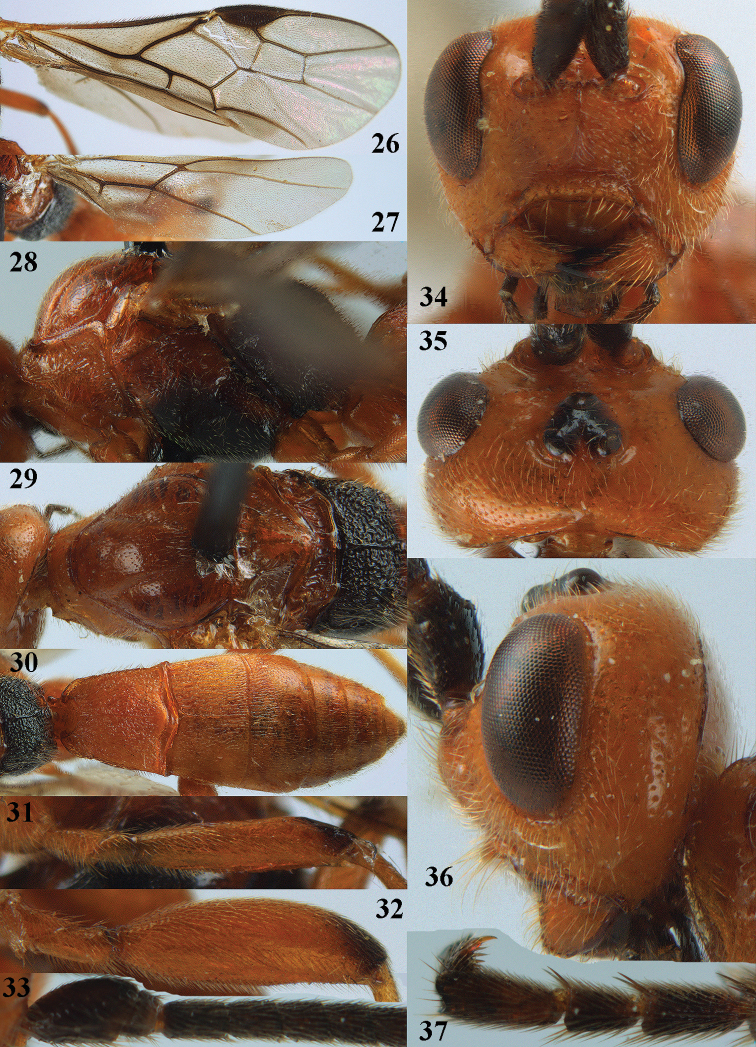
*Aleiodes aestuosus* (Reinhard), ♀, Cyprus, Yermasoyja River **26** fore wing **27** hind wing **28** mesosoma lateral **29** mesosoma dorsal **30** metasoma dorsal **31** fore femur lateral **32** hind femur lateral **33** base of antenna **34** head anterior **35** head dorsal **36** head lateral **37** inner hind tarsal claw.

#### Aleiodes agilis

Taxon classificationAnimaliaHymenopteraBraconidae

(Telenga, 1941)

AE4E13C8-7737-5FFB-BC33-9181B93B6C14

Figs 38–39 , 40–49

Rhogas (Rhogas) agilis Telenga, 1941: 165–166, 417.
Rogas agilis ; Shenefelt 1975: 1217.
Rogas (Rogas) agilis ; Tobias, 1976: 83, 1986: 76 (transl. 122, 124) (lectotype designation).
Aleiodes agilis ; Fortier and Shaw 1999: 230.
Aleiodes (Chelonorhogas) agilis ; Ma et al. 2002: 98; Farahani et al. 2015: 240.
Rhogas desertus var. armenica Telenga, 1959: 85; Tobias, 1976: 83 (as synonym of A. agilis (Telenga, 1941)), 1986: 76 (transl. 122, 124; id.).

##### Type material.

Lectotype, ♀ (ZISP; examined via photos), “Persiya [= **Iran**], Tavriz, 21.iii.[19]14, Andrievskij”, “*Rhogas agilis* sp. n., N. Telenga det.”, “Syntypus *agilis* Tel.”, “Lectotypus *Rogas agilis* Tel., design. Tobias, 1980”; paralectotype, ♀ (ZISP; id.), “**Armenia**, pr. Eriwan [= Yerevan], A. Schelkovnikow / Ragakag, 19.iii.[19]25”, “Paralectotypus *Rhogas agilis* Telenga, design. Tobias, 1986”. In the original description the latter date is incorrectly cited as 24.vii.1925.

##### Molecular data.

None.

##### Biology.

Unknown. It appears to fly very early in the year (March).

##### Diagnosis.

**Figures 38, 39.**
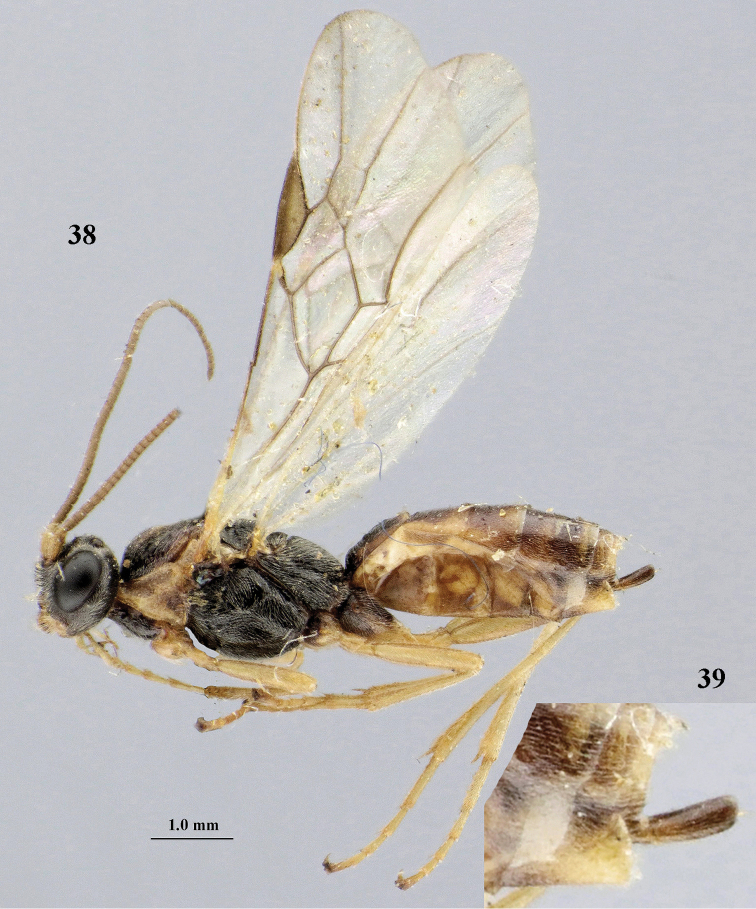
*Aleiodes agilis* (Telenga), ♀, paralectotype **38** habitus lateral **39** ovipositor sheath lateral. Photographs by K. Samartsev.

**Figures 40–49.**
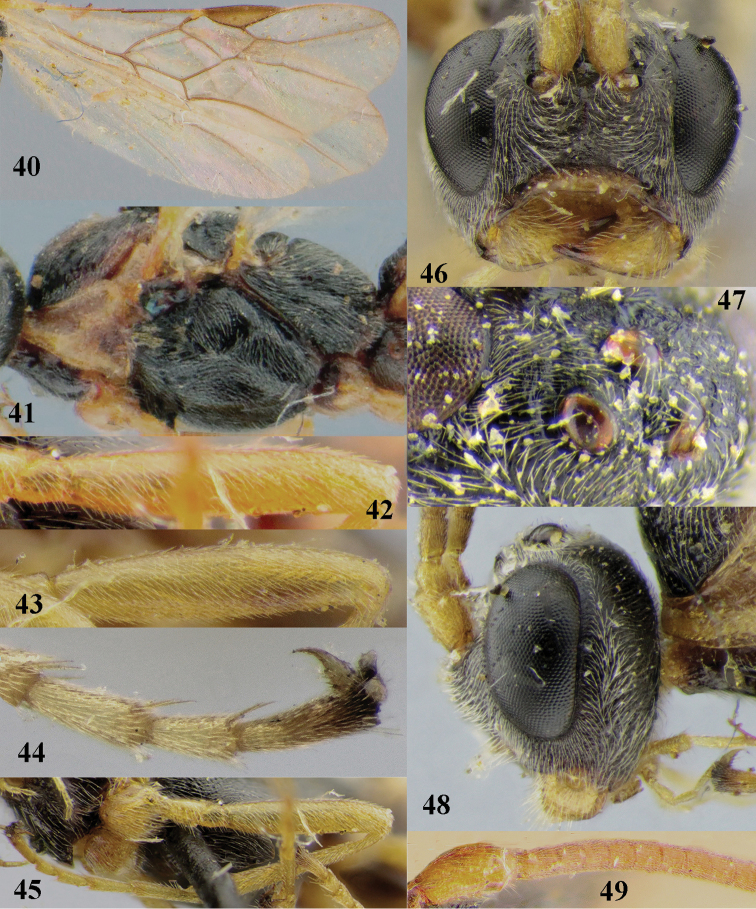
*Aleiodes agilis* (Telenga), ♀, paralectotype, but 47 of lectotype **40** wings **41** mesosoma lateral **42** fore femur lateral **43** hind femur lateral **44** outer hind tarsal claw **45** fore leg **46** head anterior **47** head dorso-lateral **48** head lateral **49** base of antenna. Photographs by K. Samartsev.

Maximum width of hypoclypeal depression approx. 0.8 × minimum width of face; anterior part of clypeus very narrow (Fig. 46); OOL 1.0–1.3 × diameter of posterior ocellus and coarsely remotely punctate with some weak rugulosity; head and mesosoma (except pronotal side partly and mesoscutum medio-posteriorly and laterally) blackish; mandible massive triangular, coarsely punctate and with thick ventral lamella (Fig. 46); face largely transversely rugose and conspicuously whitish setose; frons rugose and shiny; vertex and temple coarsely remotely punctate and shiny; area of precoxal sulcus (but posteriorly superficially) distinctly rugose; lateral lobes of mesoscutum largely smooth (anteriorly becoming densely punctate and somewhat rugose), whitish setose and with satin sheen, middle lobe distinctly punctate; basal half of wings (except anteriorly) largely glabrous and remainder of wing inconspicuously setose; vein r of fore wing approx. 0.6 × vein 3-SR (Fig. 40); vein 1-CU1 0.2–0.3 × as long as 2-CU1, narrow and subhorizontal; tarsal claws long, slender, hardly bent and simple (Fig. 44); 1^st^ and base of 2^nd^ tergite weakly longitudinally rugulose with some superficial punctures; metasoma dark brown but with yellow patches (Fig. 38), clypeus and antenna (except yellow scapus and pedicellus) yellowish brown; legs and palpi pale yellowish, but hind coxa and most of middle coxa dark brown.

##### Description.

Paralectotype, ♀, length of fore wing 6.6 mm, of body 7.0 mm.

***Head.*** Antennal segments of ♀ 47, antenna as long as body and its subapical segments moderately slender; frons rugose, shiny; OOL 1.3 × diameter of posterior ocellus; OOL and vertex remotely punctate, with satin sheen, OOL also with some rugulae; anterior part of clypeus 9 × wider than high, coarsely punctate and rather convex; clypeus above lower level of eyes; ventral margin of clypeus thick and not protruding forwards (Fig. 48); width of hypoclypeal depression 0.8 × minimum width of face (Fig. 46); length of eye 1.8 × temple in dorsal view; vertex behind stemmaticum convex and sparsely punctate; length of malar space 0.19 × length of eye in lateral view; occipital carina nearly complete, fine and ventrally strongly curved; mandible massive triangular, coarsely punctate and with thick ventral lamella (Fig. 46).

***Mesosoma.*** Lateral lobes of mesoscutum largely smooth, with satin sheen and whitish setose, middle lobe distinctly punctate and setose; prepectal carina complete and lamelliform; precoxal area of mesopleuron widely rugose, but posterior 0.2 narrowly striate; mesopleuron largely weakly and sparsely punctate, shiny, but anteriorly becoming densely punctate and somewhat rugulose; scutellum largely smooth, with some punctures; propodeum evenly convex, finely rugose and with medio-longitudinal carina, without tubercles.

***Wings.*** Fore wing: basal half largely glabrous; r 0.6 × 3-SR (Fig. 40); 1-CU1 subhorizontal, 0.25 × as long as 2-CU1; r-m 0.7 × as long as 3-SR; 2^nd^submarginal cell robust (Fig. 40); cu-a distinctly inclivous; 1-M weakly curved posteriorly. Hind wing: basal 0.4 of marginal cell slightly widened and distally strongly widened, its apical width approx. twice width at level of hamuli; 2-SC+R subquadrate; m-cu indistinct; M+CU:1-M = 24:19; 1r-m 0.7 × 1-M.

***Legs.*** Tarsal claws slender, slightly curved and only setose (Fig. 44); hind coxa partly obliquely striate dorsally; tarsi slender, segments (except telotarsus) with long apical spiny bristles; length of hind femur and basitarsus 5.0 and 6.0 × their width, respectively; length of inner hind spur 0.3 × hind basitarsus.

***Metasoma.*** First tergite robust, as long as wide apically, distinctly narrowed anteriorly and rather flat posteriorly; 1^st^ and 2^nd^ tergites finely longitudinally striate-rugulose; medio-longitudinal carina of 1^st^ and 2^nd^ tergites indistinct; 2^nd^ tergite 0.7 × longer than its basal width; medio-basal area of 2^nd^ tergite wide triangular, rather short; 2^nd^ suture shallow and narrow; 3^rd^ tergite mainly smooth and with satin sheen; 4^th^ and apical half of 3^rd^ tergite without sharp lateral crease; ovipositor sheath rather slender, with short setae and apically truncate (Fig. 39).

***Colour.*** Black; pronotal side largely yellowish brown; mesoscutum medio-posteriorly and postero-laterally partly chestnut brown; tegulae, clypeus and antenna (except yellow scapus and pedicellus) yellowish brown; mandible, legs and palpi pale yellowish, but hind coxa and most of middle coxa dark brown; metasoma dark brown but with yellow patches (Fig. 38); pterostigma brown medially and dark brown laterally; ovipositor sheath dark brown; veins of fore wing (but pale yellow in basal 0.2 of fore wing) brown; wing membrane hyaline.

##### Distribution.

Armenia, Iran. Included in this revision, because it may occur in Turkey.

#### Aleiodes apicalis

Taxon classificationAnimaliaHymenopteraBraconidae

(Brullé, 1832)

C39FE455-96C0-50F4-9FD1-84B0711DC24F

Figs 50–53 , 54–65 , 66–71

Bracon apicalis Brullé, 1832: 381 [examined].
Rhogas apicalis ; Fahringer 1932: 317–318.
Rogas apicalis ; Shenefelt 1975: 1218.
Aleiodes apicalis ; Shaw et al., 1998: 63; Fortier and Shaw 1999: 227; Zaldívar-Riverón et al. 2004: 234, 2008: figs 2–6.
Aleiodes (Chelonorhogas) apicalis ; Falco et al. 1997: 60; Farahani et al. 2015: 240–242; Abdolalizadeh et al. 2017: 36.
Rogas reticulator Nees, 1834: 211; Shenefelt 1975 (as synonym of A. ductor); Papp 2005: 176 (id.). Syn. nov.
Aleiodes reticulator ; Papp, 1991a: 70 (as synonym of A. ductor).
Rogas bicolor Lucas, 1849: 336–337 (not Spinola 1808); Shenefelt 1975: 1219; Papp 1985a: 157 (lectotype designation), 2005: 176 (as synonym of A. ductor). Syn. nov.
Rogas rufo-ater Wollaston, 1858: 24; Shenefelt 1975: 1247; Papp 1990: 90 (as synonym of A. ductor) [examined]. Syn. nov.
Rhogas rufoater ; Fahringer 1934: 321.
Rhogas bicolorinus Fahringer, 1932: 318 (replacement name for Rogas bicolor Lucas). Syn. nov.
Rhogas reticulator var. atripes Costa, 1884: 13; Papp 1990: 90 (as synonym of R. rufoater). Syn. nov.
Rhogas ductor var. atripes ; Fahringer 1932: 244.
Aleiodes (Neorhogas) ductor var. atripes ; Papp 1985a: 157.
Rhogas similis Szépligeti, 1903: 114 (not Curtis 1834); Papp 1985a: 157–158 (lectotype designation and as synonym of A. ductor), 2005: 176 (id.). Syn. nov.
Rhogas ductor var. similis ; Fahringer 1932: 245.
Rogas ductor auct. p.p.; Shenefelt, 1975: 1226–1227; Zaykov 1980a: 112; Tobias 1976: 85, 1986: 80 (transl.: 133); Samartsev and Belokobylskij 2013: 765.
Aleiodes ductor auct. p.p.; Bergamasco et al. 1995: 5.

##### Type material.

Holotype of *B. apicalis*, ♂ (MNHN) “[**Greece**], Morée, Muséum Paris, Brullé 4187-33”, “Type”, “*Bracon apicalis* Brullé, Type”. Lectotype of *R. similis*, ♂ (MTMA) “[**Hungary**], Kecskemét, Szépligeti”, “Hym. Typ. No. 7021, Mus. Budapest”, “Lectotypus”, “*Rhogas similis* Szépl. 1903 ♂, Papp, 1984”, “*Rhogas reticulator* var. *similis* Sz., det. Szépligeti, 1906”, “*Aleiodes ductor* Thunbg., det. Papp, J., 1983”. Holotype of *R. rufo-ater*, ♂ (ZJUH) “[**Portugal**], Madeira, Wollaston, 55.7”, “*Rogas rufo-ater*, W.”, Type, H.T.”, “B.M. Type Hym., 3.c.241”.

##### Additional material.

Albania, Austria, Bosnia & Herzegovina, Bulgaria, Croatia, Cyprus, Czech Republic, France (including Corsica), Germany, Greece (including Chios, Corfu, Crete, Lesbos, Rhodes), Hungary, Italy (including Sardinia, Sicily), Malta, Moldova, Montenegro, Morocco, North Macedonia, Portugal (including Madeira), Romania, Russia (including Dagestan), Serbia, Slovakia, Spain (including Mallorca and Canary Islands: Tenerife, Fuerteventura), Switzerland, Tunisia, Turkey, Ukraine, [Georgia, Kazakhstan, Oman, Iran, Iraq, Israel, Syria, Turkmenistan]. Specimens in ZJUH, BZL, CMIM, CNC, HSC, MRC, MSC, MTMA, NMS, RMNH, SDEI, UNS, ZISP, ZSSM. This is a mainly Mediterranean species, extending into Central Europe and West Asia, and one of the commonest species of the group in the Mediterranean area. One surprising female from Sweden (Skåne, Käseberga, MV light 17-vii-14.ix.2013, N. Ryrholm & C. Källander, in NMS) is presumed, like two British specimens (England, V.C. 3, S. Devon, Slapton Ley 7–14.vi.1932, H.St.J. Donisthorpe, in ZJUH; V.C. 22, Berkshire, Beale Park, 25–27.vii.2018, Rothamsted trap, in coll. A.C. Galsworthy destined for ZJUH) and one specimen from Netherlands (Lexmond, ZH, 10.viii.2004, C. Gielis in RMNH) to have been deposited there by winds from southern Europe or N. Africa rather than representing an established breeding population. Whether or not *A. apicalis* can eventually establish permanent populations, i.e., with winter survival, in these relatively northerly parts of Europe may depend on whether its host can do likewise.

##### Molecular data.

MRS008 (Turkey), MRS111 (Turkey), MRS112 (Turkey), MRS181 (Russia), MRS869 (Sweden).

##### Biology.

Time of flight varies according to harshness of summer. In its more temperate sites plurivoltine April-September(October), overwintering in the mummy, but in Cyprus (and presumably other places with extremely hot dry summers) it appears to be most active from autumn to spring (October–May), with a prolonged summer diapause (June–October or later) in the mummy (reared series ex “*Plusia*” in ZJUH and NMS, W.R. Ingram, six with mummification dates recorded in May or June and adult emergence in the following October–December, further specimens in the series have only one date, which is ambiguous). Reared from Noctuidae: *Autographa gamma* (Linnaeus) (6 [4 ZISP/Moldova, 1 HSC/Germany, 1 NMS/Malta]; J.L. Gregory, H. Schnee), indet. Plusiinae (14). There is no reason to suppose that the hosts recorded as indet. Plusiinae are anything except *A. gamma*. A further specimen labelled as ex *Peribroma* [sic] *saucia* is accompanied by a clearly Plusiinae mummy (Sicily, NMS). Also, one labelled as from *Anarsia lineatella* Zeller (Gelechiidae) (Ukraine, ZISP), but without a mummy and clearly in error on grounds of size alone. Another specimen labelled as “ex *Sesamia* pupa” (Iran, ZJUH) lacks its mummy but accompanies two individuals of *A. aestuosus* (q. v.) from the same source, and the remarks made under that species apply also to this record – but with the added objection that the small hypoclypeal opening and flat clypeus of *A. apicalis* strongly suggest that its hosts do not mummify in deep concealment. The mummy (Fig. 52) is of a pale chalky buff colour, and the cocoon occupies approx. abdominal segments 4–7 of the host larva. Several of the mummies examined, all of which seem to be penultimate instar, have been formed in a more or less curled leaf beneath a web (Fig. 53) that the host had been induced to spin before being mummified, and were weakly stuck to the substrate.

##### Diagnosis.

Maximum width of hypoclypeal depression 0.3–0.4 × minimum width of face (Fig. 60); antennal segments of ♀ 44–51 and flagellar segments moderately robust (Figs 64, 65); ventral margin of clypeus thick and obtuse apically and clypeus not protruding outwards (Fig. 62); vertex, mesoscutum, metapleuron and scutellum normally shiny and without dense granulation, at most with some superficial micro-sculpture; frons (and more or less vertex) with striae (Fig. 61) or rugae; scutellum largely smooth and shiny; mesopleuron largely smooth; vein 2-CU1 of fore wing approx. as long as vein 1-CU1 (Fig. 54); vein M+CU of hind wing distinctly longer than vein 1-M (Fig. 54); hind tarsal claws of ♀ with rather slender and brownish pecten (Fig. 63); basal half of hind tibia (largely) pale yellowish, **or** if black (var. rufoater) then also fore femur black; 3^rd^ tergite (except basally) largely smooth; medially 4^th^–6^th^ tergites of ♂ slightly concave and with dense band of medium-sized setae (Figs 68, 69); head, mesoscutum and scutellum black; 2^nd^ tergite yellowish or reddish.

##### Description.

Redescribed ♀ (RMNH) from Hungary (Budapest), length of fore wing 5.1 mm, of body 6.7 mm.

***Head.*** Antennal segments of ♀ more than 40, but apical segments missing (length of antenna of ♀ from Lesbos 1.4 × fore wing and its subapical segments robust); frons with coarse curved rugae, shiny; OOL 1.5 × diameter of posterior ocellus, and distinctly striate; vertex transversely striate, rather weak; clypeus normal, punctulate and convex; ventral margin of clypeus thick and not protruding forwards; width of hypoclypeal depression 0.3 × minimum width of face (Fig. 60); length of eye 1.6 × temple in dorsal view (Fig. 61); vertex behind stemmaticum transversely striate; clypeus near lower level of eyes; length of malar space 0.4 × length of eye in lateral view; occipital carina complete, fine.

***Mesosoma.*** Mesoscutal lobes largely smooth, punctulate, shiny; prepectal carina complete, rather strong; precoxal area of mesopleuron largely smooth; mesopleuron above precoxal area weakly and sparsely punctate, especially posteriorly; scutellum largely smooth, with striae laterally; propodeum evenly convex, coarsely vermiculate-rugose, only anteriorly with median carina, without tubercles.

***Wings.*** Fore wing: r 0.6 × 3-SR (Fig. 54); 1-CU1 horizontal, equal to or slightly longer than 2-CU1; r-m 0.9 × 3-SR; 2^nd^ submarginal cell comparatively short (Fig. 54); cu-a vertical, slightly curved posteriorly; 1-M straight posteriorly. Hind wing: marginal cell basally slightly and distally strongly widened, its apical width 2.6 × width at level of hamuli (Fig. 54); 2-SC+R subquadrate; m-cu indistinct; M+CU:1-M = 5:3; 1r-m 0.7 × 1-M.

***Legs.*** Tarsal claws with rather slender and medium-sized brownish pecten (Fig. 63); hind coxa largely densely punctate; hind trochantellus medium-sized; length of hind femur and basitarsus 5.1 and 6.0 × their width, respectively; length of inner hind spur 0.5 × hind basitarsus.

***Metasoma.*** First tergite robust, evenly convex; 1^st^ and 2^nd^ tergites rather coarsely obliquely rugose; 1^st^ tergite and basal half of 2^nd^ tergite with median carina; 2^nd^ tergite robust and with striae diverging posteriorly; medio-basal area of 2^nd^ tergite wide triangular, rather short; 2^nd^ suture rather deep medially; 3^rd^ tergite largely smooth, except anteriorly with some striae; 4^th^ and apical half of 3^rd^ tergite without sharp lateral crease; ovipositor sheath with rather long setae and apically rounded (Fig. 51).

***Colour.*** Black; scapus, pedicellus, tegulae (but humeral plate brownish yellow), base of hind tibia narrowly, apical half of hind tibia, telotarsi, hind tarsus largely, ventral apical half of metasoma, pterostigma and veins (except C+SC+R of fore wing) dark brown; remainder of basal half of antenna and palpi yellowish brown; remainder of legs (but apical two-fifths of hind femur black), 1^st^ and 2^nd^ tergites, 3^rd^ tergite basally and laterally orange brown; remainder of hind tibia pale yellowish; apex of middle femur and wing membrane somewhat infuscate.

*Variation*. *A. apicalis* is very variable in colour and the colour patterns are not restricted to certain areas, but in general southern Palaearctic specimens are darker than northern ones (or specimens from high altitudes). The tegula is dark brown or black, and the humeral plate usually paler than the tegula or equally black, but both usually yellowish in southern specimens; the hind tarsus is dark brown or black, but sometimes 3^rd^ and 4^th^ segments yellowish; the hind tibia variably reddish to black, but palest at extreme base; the pronotum is very occasionally reddish. The extent of black colouration of the legs is especially variable, and sometimes all legs are entirely black (var. rufoater (Wollaston, 1858)). Antenna, especially in females, can be more or less light reddish brown, especially basally, or dark brown/black throughout. Antennal segments: ♀ 44(1), 46(3), 47(11), 48(20), 49(31), 50(41), 51(19), 52(10), 54(3), 55(1), 57(1); ♂ 46(3), 47(7), 48(17), 49(29), 50(30), 51(32), 52(11), 53(5), 54(1). Males have on average approx. one antennal segment more than females. Apical tergites of ♂ type 4, setosity dense (making the tergites appear concave; Figs 68, 69) and fringe weak.

##### Distribution.

*Albania, Austria, *Bosnia & Herzegovina, *Bulgaria, *Croatia, Cyprus, *Czech Republic, *France (including Corsica), *Georgia, *Germany, Greece (including Chios, Corfu, Crete, Lesbos, Rhodes), *Hungary, Iran, *Iraq, *Israel, *Italy (including Sardinia, Sicily), *Kazakhstan, *Malta, *Moldova, *Montenegro, *Morocco, *North Macedonia, *Oman, *Portugal (including Madeira), *Romania, *Russia (including Dagestan), *Serbia, *Slovakia, Spain (including Mallorca and Canary Islands: Tenerife, Fuerteventura), *Syria, Switzerland, *Tunisia, Turkey, *Turkmenistan.

##### New synonymy.

The synonymy of *Rogas rufo-ater* Wollaston, 1858, and *Rhogas similis* Szépligeti, 1903, are based on examination of the types listed above. The lectotype of *Rogas bicolor* Lucas, 1849 (not Spinola, 1808) and of *Rhogas bicolorinus* Fahringer, 1932, has been examined by Dr Jenö Papp and we agree with his opinion that it is a synonym of *A. ductor* auct. (= *A. apicalis*). The types of *Rogas reticulator* Nees, 1834, and Rhogas reticulator var. atripes Costa, 1884, are lost or unavailable and their synonymy is based on the original description and the interpretation by later authors.

**Figures 50–53.**
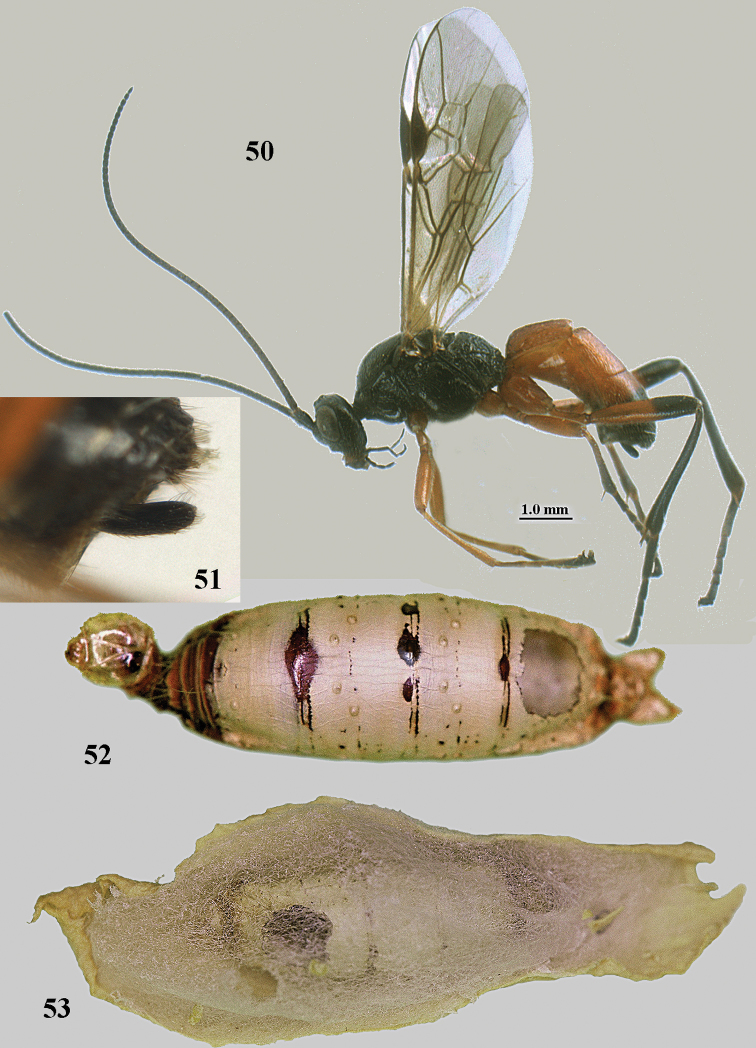
*Aleiodes apicalis* (Brullé), ♀, Greece, Thimiana Chios, but 52 mummies of *Autographa gamma* (Linnaeus) from Malta and 53 of undetermined plusiine host from Cyprus **50** habitus lateral **51** ovipositor sheath lateral **52** mummy dorsal **53** mummy covered by silk of host.

**Figures 54–65.**
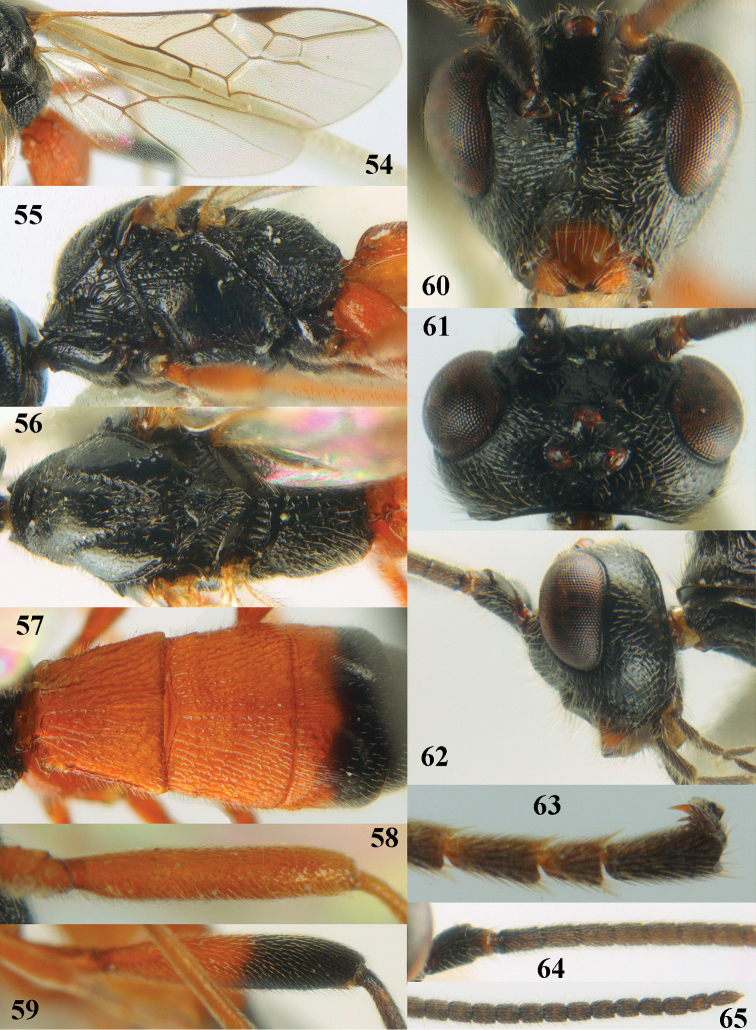
*Aleiodes apicalis* (Brullé), ♀, Bulgaria, Rodopi **54** wings **55** mesosoma lateral **56** mesosoma dorsal **57** 1^st^ –3^rd^ metasomal tergites dorsal **58** fore femur lateral **59** hind femur lateral **60** head anterior **61** head dorsal **62** head lateral **63** outer hind tarsal claw **64** base of antenna **65** apex of antenna.

**Figures 66–71.**
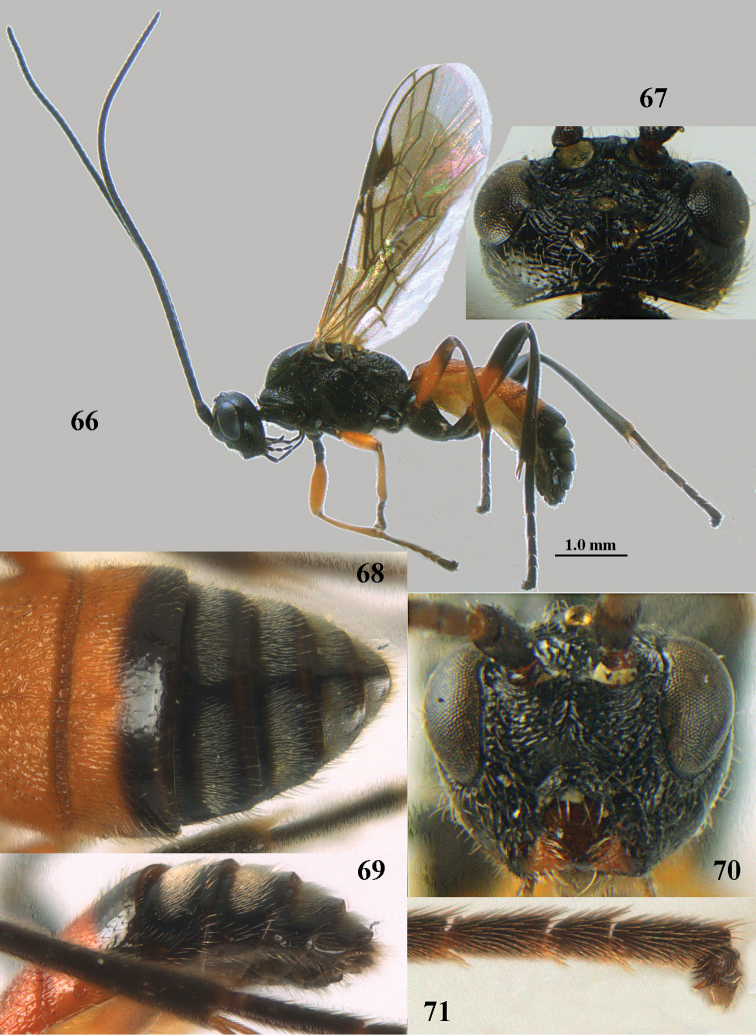
*Aleiodes apicalis* (Brullé), ♂, Turkey, Sivas **66** habitus lateral **67** head dorsal **68** 3^rd^ –7^th^ metasomal tergites dorsal **69** 3^rd^ –7^th^ metasomal tergites lateral **70** head anterior **71** outer hind tarsal claw.

#### Aleiodes arnoldii

Taxon classificationAnimaliaHymenopteraBraconidae

(Tobias, 1976)

1E255C34-5930-595A-9BD5-24D03A2D81BF

Figs 72–73 , 74–79 , 80 , 81–92

Rogas (Rogas) arnoldii Tobias, 1976: 84, 222, 1986: 78 (transl.: 128).
Aleiodes (Neorhogas) arnoldi [sic!]; Papp 1985a: 152.
Aleiodes (Neorhogas) arnoldii ; Papp 1991a: 87.

##### Type material.

Holotype, ♀ (ZISP) “[**Azerbaijan**], Kosmoljan, Zuvan, 19.v.[1]936, Arnoldi”, “Holotypus *Rogas arnoldii* Tobias”.

##### Additional material.

1 ♂ (RMNH), “**Turkey**, Hakkâri, Tanin Tanin Pass, 25.vi.1985, 2200 m, C.J. Zwakhals”. Male is provisionally associated with this species; it may belong to a related species.

##### Molecular data.

None.

##### Biology.

Unknown. The holotype was collected in May.

##### Diagnosis.

**Figures 72, 73.**
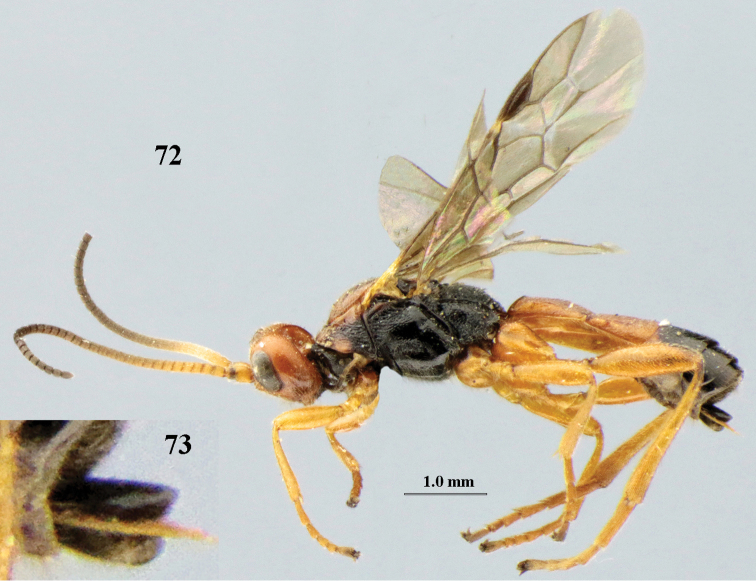
*Aleiodes arnoldii* (Tobias), ♀, holotype **72** habitus lateral **73** ovipositor sheath lateral. Photographs: K. Samartsev.

Maximum width of hypoclypeal depression 0.4–0.5 × minimum width of face (Fig. 78); clypeus obtuse apically and not protruding in lateral view (Fig. 77); length of malar space of ♀ 0.5–0.6 × height of eye in lateral view; antennal segments of ♀ 35–37 and length of antenna of ♀ 0.8–0.9 × fore wing; OOL sparsely punctate; lateral lobes of mesoscutum largely smooth; posterior half of notauli shallow; precoxal area coarsely vermiculate-rugose medially; head, palpi and part of mesosoma of ♀ yellowish brown; pterostigma dark brown; apex of hind tibia of ♀ yellowish; hind tarsal claws yellowish or brownish setose (Fig. 72); 4^th^–6^th^ tergites of ♂ flat and normally setose, but setae slightly longer than on basal tergites (Fig. 92).

##### Description.

Holotype, ♀, length of fore wing 4.4 mm, of body 5.7 mm.

***Head.*** Antennal segments of ♀ 37, length of antenna 0.85 × fore wing, its subapical segments quadrate; frons with rather coarse curved rugae, shiny, and rugose behind antennal sockets; OOL 2.0 × diameter of posterior ocellus, and finely remotely punctate, interspaces much larger than diameter of punctures; vertex spaced punctate, shiny; face transversely rugose; clypeus finely rugulose and with long setae; ventral margin of clypeus thick and not protruding forwards; width of hypoclypeal depression 0.45 × minimum width of face; length of eye 1.1 × temple in dorsal view (Fig. 79); vertex behind stemmaticum rugulose; clypeus near lower level of eyes; length of malar space 0.55 × length of eye in lateral view.

***Mesosoma.*** Mesoscutal lobes largely smooth, shiny, sparsely and finely punctate; precoxal area of mesopleuron coarsely rugose, but absent posteriorly; metapleuron remotely punctate, interspaces much wider than diameter of punctures, shiny; mesopleuron above precoxal area (except speculum) punctate and dorsally rugose; scutellum sparsely punctate or punctulate, medio-posteriorly rugulose and with some striae laterally, no carina; propodeum evenly convex and coarsely vermiculate-rugose, medio-longitudinal carina strong in basal 0.6, and without tubercles.

**Figures 74–79.**
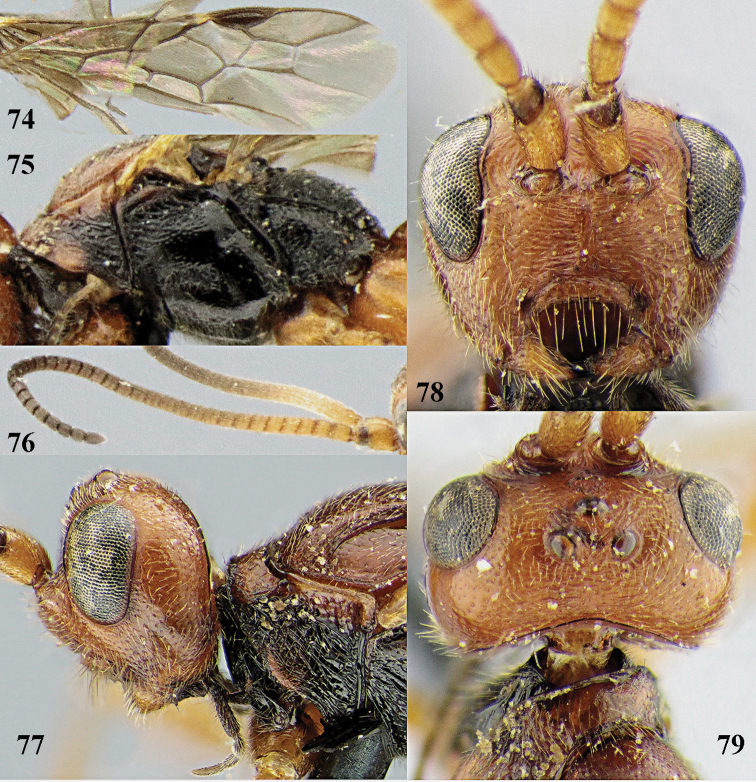
*Aleiodes arnoldii* (Tobias), ♀, holotype **74** wings **75** mesosoma lateral **76** antenna **77** head lateral **78** head anterior **79** head dorsal. Photographs: K. Samartsev.

***Wings.*** Fore wing: just reaching apex of metasoma; r 0.35 × 3-SR (Fig. 74); 1-CU1 horizontal, 0.45 × 2-CU1; r-m unsclerotized; 2^nd^ submarginal cell medium-sized (Fig. 74); cu-a vertical, straight; 1-M nearly straight posteriorly; 1-SR wide. Hind wing: marginal cell linearly widened, its apical width 2.2 × width at level of hamuli (Fig. 72); 2-SC+R subquadrate; m-cu distinct, but unsclerotized and as long as cu-a; M+CU:1-M = 15:9; 1r-m 0.7 × 1-M.

***Legs.*** Tarsal claws subpectinate, with six yellowish medium-sized pectinal bristles; hind coxa obliquely striated dorsally, punctulate laterally; hind trochantellus robust; length of hind femur and basitarsus 3.6 and 4.6 × their width, respectively; length of inner hind spur 0.5 × hind basitarsus.

***Metasoma.*** First tergite rather flattened, as long as wide apically; 1^st^ and 2^nd^ tergites coarsely longitudinally and densely rugose, robust and posterior corners of 1^st^ protruding outside base of 2^nd^ tergite, with distinct median carina; medio-basal area of 2^nd^ tergite wide and short; 2^nd^ suture moderately deep and crenulate; basal half of 3^rd^ tergite longitudinally striate, remainder of metasoma largely smooth, punctulate; 4^th^ and apical half of 3^rd^ tergite without sharp lateral crease; ovipositor sheath wide, setose and apically truncate (Fig. 73).

***Colour.*** Yellowish brown; mesosoma (except mesoscutum, scutellum medially, pronotum anteriorly and dorsally), ovipositor sheath, 3^rd^ tergite (except antero-lateral corners) and following segments black; apical half of antenna, pedicellus, palpi, hind femur apico-dorsally, telotarsi, veins, parastigma basally and pterostigma dark brown; wing membrane rather brownish infuscate.

**Figure 80.**
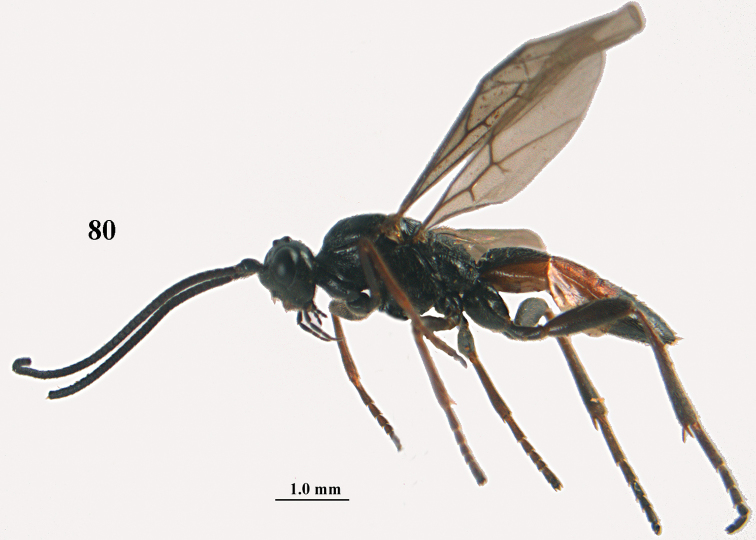
*Aleiodes arnoldii* (Tobias), ♂, Turkey, Tanin Pass, habitus lateral.

***Variation.*** Antennal segments of ♀ 37(1). Male is largely black, except for 2^nd^ tergite and anterior half of 3^rd^ tergite (Fig. 80).

##### Distribution.

Azerbaijan, *Turkey.

##### Notes.

Easily confused with *A. ruficornis* (Herrich-Schäffer); the relative size of the clypeus (wider and somewhat shorter in *A. arnoldii* than in *A. ruficornis*) seems to be the main difference in both sexes. In addition, the female of *A. arnoldii* has the temple ventrally and the malar space yellowish brown (dark brown in *A. ruficornis*). The male has darker legs and 1^st^ metasomal tergite than the female (the sexes more similar in *A. ruficornis*). Also reported from Uzbekistan (Yuldashev, 2006); the record from Poland (Huflejt, 1997) most likely concerns *A. ruficornis* (Herrich-Schäffer). *Aleiodes arnoldiisensu*Farahani et al. (2015) concerns a species closely related to *A. gasterator* (Jurine) but has basal half of 3^rd^ tergite coarsely longitudinally rugose, antenna of ♀ with 30–35 segments (of ♂ 36), head linearly narrowed ventrally and subbasal antennal segments of ♀ slightly slenderer.

**Figures 81–92.**
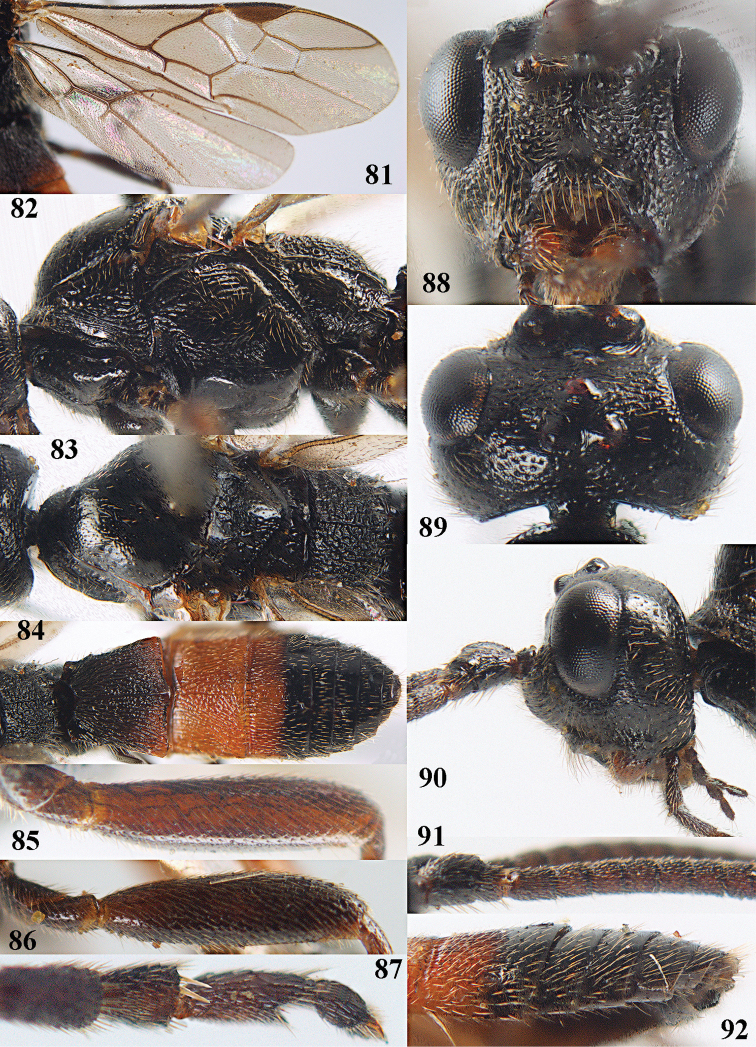
*Aleiodes arnoldii* (Tobias), ♂, Turkey, Tanin Pass **81** wings **82** mesosoma lateral **83** mesosoma dorsal **84** metasoma dorsal **85** fore femur lateral **86** hind femur lateral **87** inner hind tarsal claw **88** head anterior **89** head dorsal **90** head lateral **91** base of antenna **92** apex of metasoma lateral.

#### Aleiodes aterrimus

Taxon classificationAnimaliaHymenopteraBraconidae

(Ratzeburg, 1852)

D5B255F1-2788-5659-BE7B-E2FCF928FF9E

Figs 93–97 , 98–100 , 101 , 102–115

Bracon aterrimus Ratzeburg, 1852: 35; Shenefelt 1978: 1467.
Aleiodes aterrimus ; Belokobylskij et al. 2003: 398; Zaldívar-Riverón et al. 2004: 234.
Aleiodes grandis Giraud, 1857: 178; Papp 1991a: 77; Bergamasco et al. 1995: 5; Belokobylskij et al. 2003: 398; Papp 2005: 176 (as valid species) [examined].
Aleiodes (Neorhogas) grandis ; Papp 1985a: 159 (lectotype designation and as synonym of A. aterrimus); Riedel et al. 2002: 106.
Aleiodes (Chelonorhogas) aterrimus ; Falco et al. 1997: 60.
Rogas grandis ; Shenefelt 1975: 1232.
Rogas (Rogas) grandis ; Tobias 1976: 87, 1986: 81 (transl.: 134).
Rhogas malaisei Shestakov, 1940: 7.
Rogas malaisei ; Shenefelt 1975: 1237.
Aleiodes malaisei ; Shaw et al. 1998: 63 (as synonym of A. grandis Giraud); Belokobylskij et al. 2003: 398 (as synonym of A. aterrimus (Ratzeburg)); Papp 2005: 176 (as synonym of A. grandis Giraud).
Rogas (Rogas) vicinus Papp, 1977a: 114, 115 [examined]. Syn. nov.
Aleiodes (Neorhogas) vicinis ; Papp 1991a: 78.

##### Type material.

Lectotype of *A. grandis*, ♂ (MNHN), “[**Austria**:] environs de Vienne”. Holotype of *R. vicinus* (MTMA), ♀, “Yugoslavia, [**Serbia**:] Vojvonida, Fruška Gora Mts., Sremska Kamenica, 1–2.v.1972, Papp & Horvatovich”, “Holotypus ♀ *Rogas vicinus* sp. n., Papp, 1977”, “Hym. Typ. No. 2375, Mus. Budapest”; paratype of *R. vicinus*, ♀ (MTMA), “[**Romania**:] Transylvania, Szászkezd%, Silbernagel”, “Paratypus ♀ *Rogas vicinus* sp. n., Papp, 1977”, “Hym. Typ. No. 2376, Mus. Budapest”; 1 ♂ (MTMA), id., but No. 2375.

##### Additional material.

Austria, Belgium, British Isles (England V.C.s 8, 9, 10, 11, 12, 14, 15, 20, 22, 28, 29, 39), Czech Republic, Finland, Germany, Hungary, Netherlands (GE: Brummen (Leuvenheim); LI: Epen; ZH: Schoonrewoerd), Poland, Romania, Russia, Slovakia, Spain, Switzerland. Specimens in ZJUH, BZL, CNC, FMNH, HSC, MRC, MSC, MTMA, NMS, OUM, RMNH, SDEI, ZSSM.

##### Molecular data.

MRS024 (UK), MRS147 (UK).

##### Biology.

Univoltine, spending ca ten months of the year in the exposed mummy on an aerial twig. Collected from April–June, among broadleaved trees (but see paragraph below). Reared from arboreal *Amphipyra* spp.: *A. pyramidea* (Linnaeus) (29; M.G. Bloxham, C. Bystrowski, J. Connell, A.P. Fowles, G.M. Haggett, B.T. Parsons, D.L.J. Quicke, M.R. Shaw); *A. berbera* (Rungs) (5:1 [5 OUM]; G.C. Varley); *Amphipyra* sp. (8). Some of the forgoing specimens were reared and labelled in the period before it was known that there are two closely related and sympatric arboreal species of *Amphipyra* in Britain, and it is possible that British records from *A. pyramidea* (especially when collected on *Quercus*; cf. Shaw, 1981) have been overstated at the expense of *A. berbera*; however, both certainly serve as host. An account of frequency at one site is given by Shaw (1981).

Before becoming mummified the host moves to a narrow twig, to which the mummy will be very strongly glued. In the early stage of the mummification process (Fig. 99), in which the anterior end of the host is particularly contracted, the parasitoid larva strongly protrudes anteriorly to spread the necessary glue (Fig. 101). The resultant almost semi-circularly domed and hard mummy (Fig. 100), in which the parasitoid occupies approximately abdominal segments 4–7 of the host, forms in ca May–June and persists through the remainder of the summer and the following winter until the adult emerges in ca April–May. (The univoltine hosts overwinter in the egg stage.) The swollen part of the mummy, which is moderately densely lined with silk, is externally usually matt chalky buff in colour, but dark brown diamond-shaped patches centred dorsally on intersegmental areas tend to remain (Fig. 97), and sometimes (perhaps especially when the mummy is unable to dry as it forms) these are coalesced to leave a single shiny dark brown patch covering most of the dorsal surface. Some of the mummies examined might be of somewhat stunted final instar hosts, but others are more clearly penultimate instar. The outcomes of an experiment involving six females and cultured *A. pyramidea* larvae were unfortunately marred by unavoidably high temperatures and then disease overcoming the cultures so that no mummies resulted, but the following observations were made: (i) 2^nd^ to 5^th^ instar hosts were potentially attractive; (ii) 2^nd^ instar hosts were, however, often ignored or else tended to be abandoned after being paralysed with a single jab (i.e., without oviposition subsequently taking place); (iii) 3^rd^ instar hosts were often ignored, but when attacked seemed the most smoothly parasitised, sometimes with a single paralysing jab being followed, after a short pause, by a single insertion of the ovipositor for presumed oviposition, although the pattern observed for 4^th^ and 5^th^ instars also occurred with 3^rd^ instars; (iv) 4^th^ and (2 only) 5^th^ instar hosts were embraced the most enthusiastically, but it required several (3–5) injections to subdue them, and then there were usually several (3–4) separate sequential and lengthy (often as long as 80 seconds) insertions of the ovipositor (which may or may not all have been actual ovipositions), the parasitoid turning between insertions and always grasping the host with all six legs during the insertion; (v) antennation of the host was minimal, and there was no post-oviposition association; (vi) all temporarily paralysed hosts hung from the substrate by one or usually more prolegs until they recovered, presumably preventing their falling from their pabulum; (vii) there is no long-term physiological venom effect. The behaviour of adults observed toward the different instars is intriguing, and the experiment would be well worth repeating under better circumstances.

Although the above is a consistent pattern for this species, it does not account for a small number of specimens (14 ♀, 4♂ in BZL, MRC, MTMA, NMS, SDEI, ZSSM) examined from various localities in central Europe (Czech Republic, Germany, Hungary) and S. Russia. These specimens share small but rather consistent morphological differences from the usual form, in particular tendencies towards: more intense sculpture on the metasomal tergites (T3 being more or less strongly punctate or even rugose-punctate); the hind wing marginal cell parallel-sided in basal three fifths; shorter 3-SR in fore wing; basal cell of fore wing with more, and more evenly distributed, setae; fewer antennal segments; wing membrane slightly brownish. These differences are not absolutely consistent and would be easy to let pass without comment were it not for the fact that they are correlated with an apparently different phenology, as (of the ten specimens with dates recorded) five ♀ were collected in July and one in August, with only three ♀ in May and one in June (none in April). This is in marked contrast with the earlier flight time of the usual form, and the usual hosts (arboreal *Amphipyra* spp.) are not available after early June. A further ♀ specimen (MTMA) examined and returned in 1997 by MRS but apparently no longer in the main MTMA collection was labelled “Hungaria, Fót, Somlyó-hegy, 30.vii.1958, Ehik”; “Ex *Panchrysia deaurata* Esp [J. Papp’s handscript]”; “ex *Pytometra deautate* [sic]”. Unfortunately, no mummy had been preserved, but this plusiine noctuid feeds on *Thalictrum* (a low plant, not a tree) and it is unlikely for an arboreal *Amphipyra*, even if fallen from a tree above, to have been mistaken for it. The date, whether referring to collection of the host larva or emergence of the adult parasitoid, is also out of step with arboreal *Amphipyra* species. We considered but rejected the possibility that these specimens belong to a separate species, and instead conclude that under certain circumstances *A. aterrimus* can have a partial 2^nd^ brood (in the southern part of its range) which uses different hosts, and that the morphological variation is merely seasonal. The material (which does not conform to *A. sapporensis* (Watanabe), see below) is being returned to holding institutions determined as *A. aterrimus* but with “var: T3 sculpture etc.” appended to facilitate recall if necessary. It should be added that this form has (on account of its heavy metasomal sculpture and extensively parallel-sided marginal cell in the hind wing) sometimes been misidentified as *A. rugulosus*, but the two species are always easily separated by the sculpture of the mesoscutum and scutellum, as well as by leg colour.

##### Diagnosis.

Maximum width of hypoclypeal depression 0.3–0.4 × minimum width of face (Fig. 109); ventral margin of clypeus obtuse apically and not protruding outwards (Fig. 111); OOL of ♀ distinctly longer than diameter of posterior ocellus; mesoscutal lobes densely and finely punctate-coriaceous, rather matt; scutellum densely and finely coriaceous; mesosternal sulcus shallow, obsolescent or absent; vein 1-CU1 of fore wing 0.2 × vein 2-CU1 (Fig. 102); vein 2-SC+R of hind wing subquadrate or vertical (Fig. 102); hind tarsal claws with conspicuous and robust blackish pecten (Fig. 113); head black; hind tibia largely to completely black; metasoma of both sexes black; 4^th^–6^th^ tergites of ♂ flat and densely short setose, except a narrow glabrous strip centrally.

Dr K. Samartsev (in litt.) kindly brought to the first author’s attention that the East Palaearctic *A. sapporensis* (Watanabe, 1937) occurs in southern European Russia (Middle and Lower Volga territories). *Aleiodes aterrimus* and *A. sapporensis* differ only slightly, mainly by the colour of the extreme base of the hind tibia (completely dark brown in *A. sapporensis* and usually narrowly pale yellowish in *A. aterrimus*) and by the shape of temple in dorsal view (roundly narrowed in *A. sapporensis* and rather linearly narrowed in *A. aterrimus*). There is also a slight difference in the proportions of the face (*A. sapporensis* has facial width 1.50–1.60 × medial height including clypeus and *A. aterrimus* 1.65–1.75 ×). *A. sapporensis* seems to have the lateral carinae of propodeum more protruding and has 58–66 antennal segments.

##### Description.

Redescribed ♀ (RMNH) from England (Pamber Forest). Length of fore wing 7.3 mm, of body 8.6 mm.

***Head.*** Antennal segments of ♀ 59, length of antenna 1.1 × fore wing, its subapical segments rather robust; frons largely superficially granulate; OOL 1.8 × diameter of posterior ocellus, and superficially rugulose-granulate and shiny; vertex superficially rugulose-granulate, rather shiny; clypeus with some punctures; ventral margin of clypeus thick and not protruding forwards (Fig. 111); width of hypoclypeal depression 0.3 × minimum width of face (Fig. 109); length of eye 1.3 × temple in dorsal view (Fig. 110); vertex behind stemmaticum superficially granulate-rugulose; clypeus near lower level of eyes; length of malar space 0.4 × length of eye in lateral view.

***Mesosoma.*** Mesoscutal lobes densely and finely punctate-coriaceous, rather matt; precoxal area of mesopleuron largely smooth medially, densely punctate anteriorly and posteriorly; metapleuron densely punctate; metanotum with nearly complete median carina; scutellum punctate-coriaceous; propodeum rather convex and coarsely reticulate-rugose, medio-longitudinal carina nearly complete, and with slightly protruding carinae laterally.

***Wings.*** Fore wing: r 0.4 × 3-SR (Fig. 102); 1-CU1 slightly oblique, 0.2 × 2-CU1; r-m 0.6 × 3-SR; 2^nd^ submarginal cell medium-sized (Fig. 102); cu-a inclivous, straight; 1-M nearly straight posteriorly; 1-SR wide; surroundings of M+CU1, 1-M and 1-CU1 largely glabrous. Hind wing: marginal cell linearly widened, its apical width 2.0 × width at level of hamuli (Fig. 103); 2-SC+R short and vertical; m-cu absent; M+CU:1-M = 12:11; 1r-m 0.7 × 1-M.

***Legs.*** Tarsal claws with conspicuous and robust blackish pecten (Fig. 113); hind coxa largely densely punctate; hind trochantellus rather robust; length of hind femur and basitarsus 4.7 and 6.5 × their width, respectively; length of inner hind spur 0.4 × hind basitarsus.

***Metasoma.*** First tergite evenly convex, as long as wide apically; 1^st^ and 2^nd^ tergites with medio-longitudinal carina and coarsely longitudinally rugose, but posterior quarter of 2^nd^ tergite irregularly rugose and no median carina; medio-basal area of 2^nd^ tergite triangular and rather distinct (Fig. 106); 2^nd^ suture deep and narrow; basal half of 3^rd^ tergite finely punctate-rugose, remainder of metasoma superficially micro-sculptured; 4^th^ and apical half of 3^rd^ tergite without sharp lateral crease; ovipositor sheath wide, with long setae and apically truncate (Fig. 95).

***Colour.*** Black; antenna (except scapus and pedicellus), palpi, tegulae, fore and middle telotarsi, veins and pterostigma dark brown; coxae, trochanters and trochantelli, apical third of hind femur (ventrally extended to its apical two-thirds), hind tibia (except pale yellowish basal ring) and hind tarsus black, remainder of legs yellowish brown; wing membrane subhyaline.

***Variation.*** Hind femur usually only apically dark brown, but sometimes entirely dark brown; coxae black or sometimes largely yellowish brown. Two females (both NMS, from different localities) have vein r-m of fore wing absent. Males are very similar, apical tergites type 3, with fringe very weak to negligible; hind femur often only apically blackish, but sometimes up to apical 0.6 darkened. Antennal segments: ♀ 57(3), 58(1), 59(5), 60(7), 61(3), 62(6), 63(5), 64(1); ♂ 51(1), 52(1), 53(3), 54(1), 55(5), 56(7), 57(6), 58(5), 59(3), 60(1), 62(2); females have on average ca four more antennal segments than males. The antennal segments for the specimens of the abnormal series (see above) are scored separately here: ♀ 54(2), 55(1), 56(2), 57(3), 58(3), 60(1); ♂ 54(1).

##### Distribution.

Austria, *Belgium, British Isles (England), Czech Republic, *Finland, Germany, Hungary, *Netherlands, Poland, *Romania, Russia, Serbia, Slovakia, Spain, *Switzerland.

##### New synonymy.

The synonymy of *Rogas vicinus* Papp, 1977, with *Aleiodes aterrimus* (Ratzeburg, 1852) is based on the examination of the types listed above. The differences between *R. vicinus* and *R. grandis* (= *A. aterrimus*) listed in the original description (head less constricted posteriorly, apical antennal segments more robust, 1^st^ metasomal tergite less robust and 2^nd^ tergite somewhat longer) fall within the normal variation of *A. aterrimus*.

**Figures 93–97.**
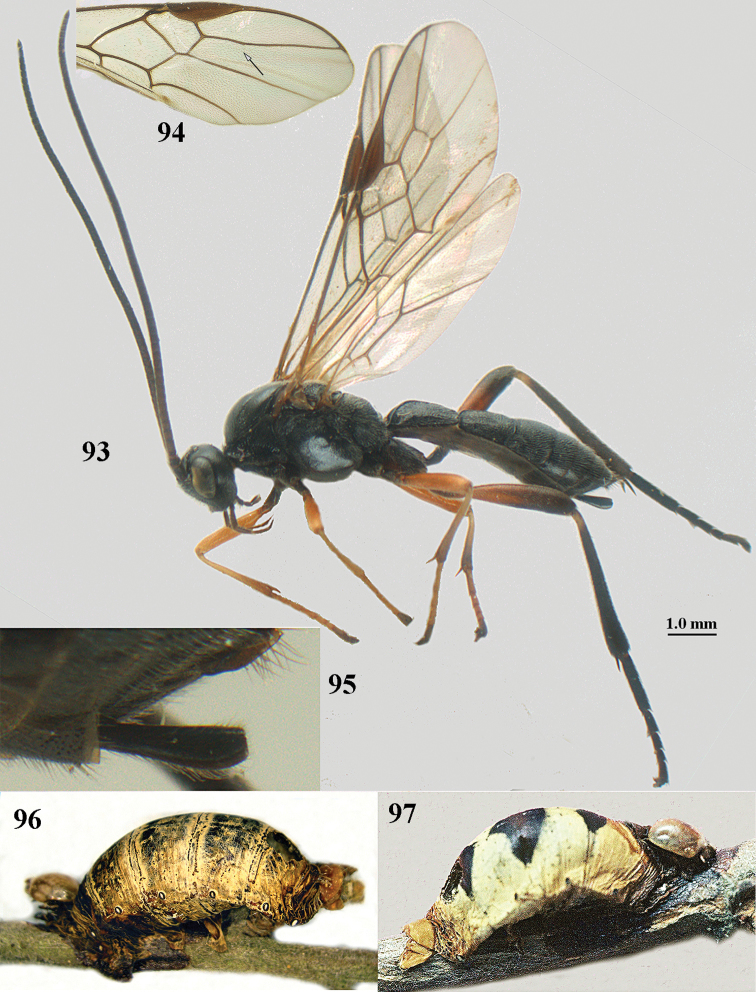
*Aleiodes aterrimus* (Ratzeburg), ♀, England, Pamber Forest **93** habitus lateral **94** detail of fore wing with arrow indicating lost vein r-m **95** ovipositor sheath lateral **96, 97** mummies of *Amphipyra* sp. showing variation in markings.

**Figures 98–100.**
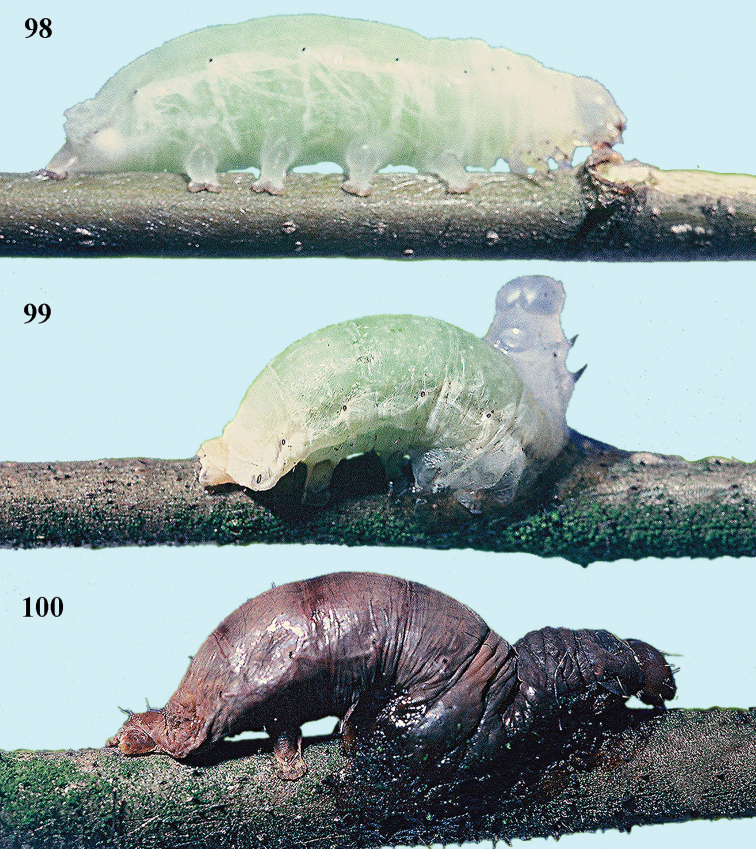
*Aleiodes aterrimus* (Ratzeburg), ♀, England, Pamber Forest **98** parasitised caterpillar of *Amphipyra* sp. **99** early stage of mummy **100** later stage of mummy.

**Figure 101.**
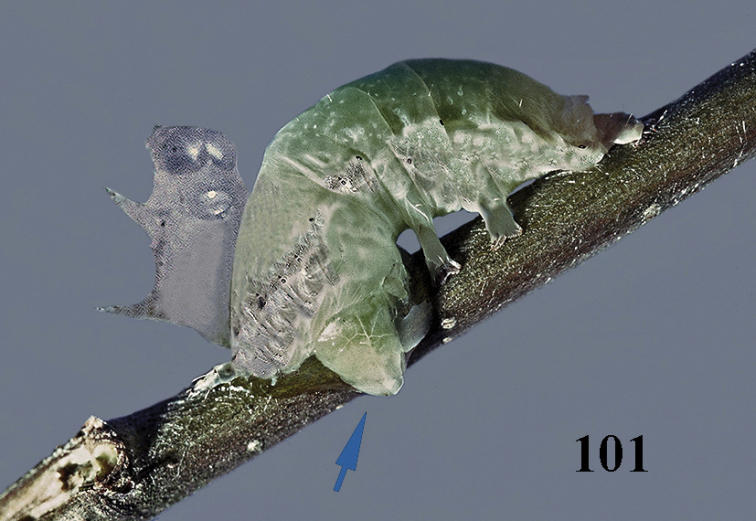
Larva of *Aleiodes aterrimus* (Ratzeburg) mummifying *Amphipyra pyramidea* (Linnaeus), with its anterior (indicated by the arrow) projecting from the ventral opening in the host to spread adhesive over a wide area.

**Figures 102–115.**
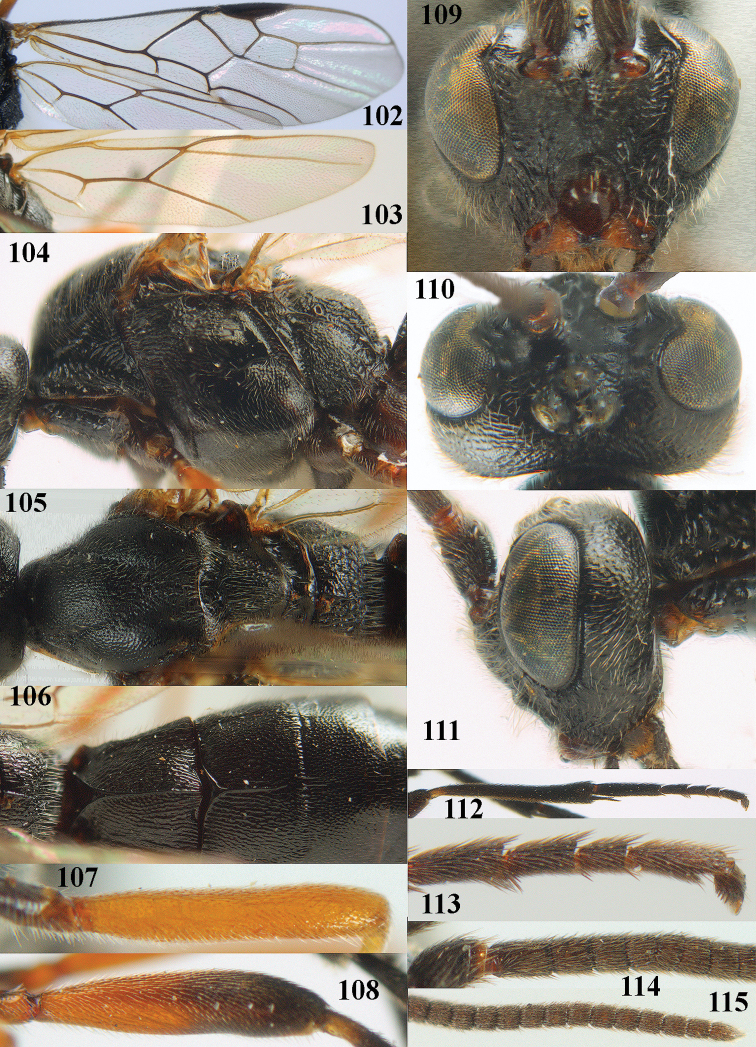
*Aleiodes aterrimus* (Ratzeburg), ♀, England, Pamber Forest, but 102 from Austria, Wien **102** fore wing **103** hind wing **104** mesosoma lateral **105** mesosoma dorsal **106** metasoma dorsal **107** fore femur lateral **108** hind femur lateral **109** head anterior **110** head dorsal **111** head lateral **112** hind tibia and tarsus lateral **113** outer hind tarsal claw **114** base of antenna **115** apex of antenna.

#### Aleiodes carbonarius

Taxon classificationAnimaliaHymenopteraBraconidae

Giraud, 1857

218FBB39-B50D-5B6F-8A64-62568C765B70

Figs 116–118 , 119–131 , 132–137

Aleiodes carbonarius Giraud, 1857: 177–178 [examined].
Aleiodes (Neorhogas) carbonarius ; Papp 1985a: 156 (lectotype designation), 1991a: 88.
Aleiodes carbonarius ; Papp 2005: 176.
Rogas carbonarius ; Shenefelt 1975: 1220–1221.
Rhogas (Rhogas) carbonarius ab. giraudi Fahringer, 1931: 236; Shenefelt 1975: 1221 (invalid name).

##### Type material.

Lectotype of *A. carbonarius*, ♂ (MNHN), “**Hunga**[**ry**]”, “2”, “Hungaria”, “Neusiedlersee/teste Papp J., 1979”, “Lectotypus”, “*Aleiodes carbonarius* Gir., 1857, ♂, Papp, 1979”. Paralectotype ♂ (MNHN) from **Austria** (near Vienna).

##### Additional material.

3 ♀ (NMS), “**Hungary**: Veszprém, nr Tótvázsony, larva coll. 21.v.2001, *Tholera decimalis*, mum. c. 12.vi.[20]01, em. 19.v., 24.v. and 25.v.[20]02, M.R. Shaw”; 1 ♂ (MSC), “A[ustria], Oberösterreich, Wels, Flughaven, 48°10'N, 14°2'E, 30.iv.2012, M. & J. Schwarz”; 1 ♂ (MTMA), “Hungaria, Csákvár”, “Vértes Hgs., Hajduvágás”, “12.v.1961, Sólymosné”, “*Rogas carbonarius* Gir. ♂, det. Papp, 1979 / compared with lectotype ♂”; 1 ♂ (NMS), “[Hungaria,] P. Szt. Lelek, Ujhelyi”, “*Rogas morio* Reinh. ♂, det. Szépligeti”, “*Rogas carbonarius* Gir. ♂, det. Papp, 1979”; 1 ♂ (MTMA), id., but Budapest, Svabhegy; 2 ♂ (MRC) “**Russia**, E. Siberia Lake Baikal, Biakalo-Lenskiy res. 20.vi. and 19.vii. [19]05, leg. Berlov”; 1 ♂ (BZL), “CSR [**Czech Rep.**], envir. Prague, 1968, Dr. Pádr”. This species appears to be sporadic in central and eastern Europe. The specimens from which Morley (1937) recorded this species as new to Britain have been examined and belong to *A. carbonaroides* sp. nov.

##### Molecular data.

MRS162 (Hungary), MRS163 (Hungary), MRS 164 (Hungary).

##### Biology.

Adults of this lowland species have been collected from the very end of April to July (see also Papp, 1999), and it is found in grassland habitats. Reared from the noctuid *Tholera decimalis* (Poda) (3:1; M.R. Shaw/Hungary). The decidedly large mummy is very similar to that of *A. grassator* and forms underground (Fig. 118). Univoltine, overwintering in the mummy.

##### Diagnosis.

Maximum width of hypoclypeal depression 0.4–0.5 × minimum width of face (Fig. 126); OOL of ♀ ca 2.6 × as long as diameter of posterior ocellus (Fig. 127) and distinctly rugose; length of 4^th^ antennal segment of ♀ ca 0.9 × its width (Fig. 129; in ♂ 0.9–1.0 times; Fig. 135); clypeus thick apically and not protruding anteriorly (Fig. 128); lobes of mesoscutum densely punctate, interspaces superficially granulate and with satin sheen; precoxal area coarsely vermiculate-rugose medially; marginal cell of fore wing of ♀ ending near level of apex of vein 3-M (Fig. 119); vein 1-CU1 of fore wing 0.4–0.5 × as long as vein 2-CU1 (Fig. 119); vein 3-SR of ♀ 1.7–2.0 × as long as vein 2-SR; vein 3-SR ca 0.7 × vein SR1 (Fig. 119; of ♂ ca 0.5×); hind tarsal claws yellowish or brownish bristly setose (Fig. 131); inner side of hind tibia of ♀ yellowish; tegulae yellowish brown; 4^th^ and 5^th^ tergites black. Probably a lowland species in C. Europe.

##### Description.

Redescribed ♀ (NMS) from Hungary (Veszprém). Length of fore wing 4.1 mm, of body ca 6.0 mm.

***Head.*** Antennal segments of ♀ 46, 4^th^ segment 0.9 × longer than wide (Fig. 129); length of antenna 1.15 × fore wing, its subapical segments robust (Fig. 130); frons with coarse curved rugae and rather shiny; OOL 2.6 × diameter of posterior ocellus and rugulose; vertex rugose and shiny; clypeus coarsely punctate; ventral margin of clypeus thick and not protruding forwards (Fig. 128); width of hypoclypeal depression 0.4 × minimum width of face (Fig. 126); length of eye 1.4 × temple in dorsal view (Fig. 127); vertex behind stemmaticum rugose; clypeus distinctly below lower level of eyes; length of malar space 0.7 × length of eye in lateral view (Fig. 128).

***Mesosoma.*** Mesoscutal lobes densely punctate, interspaces superficially granulate and with satin sheen; precoxal area of mesopleuron coarsely rugose medially and punctate posteriorly; remainder of mesopleuron mainly coarsely punctate; scutellum flat, sparsely finely punctate and with lateral carina; propodeum coarsely rugose, medio-longitudinal carina indistinct, rounded posteriorly and dorsal part rather short.

***Wings.*** Fore wing: r 0.4 × 3-SR; marginal cell ends near level of apex of 3-M (Fig. 119); 1-CU1 horizontal and slightly widened, 0.45 × 2-CU1; r-m 0.3 × 3-SR; 2^nd^ submarginal cell elongate (Fig. 119), 3-SR twice as long as 2-SR; cu-a vertical, straight; 1-M nearly straight posteriorly; 1-SR slender and medium-sized; surroundings of M+CU1, 1-M and 1-CU1 setose. Hind wing: marginal cell linearly widened, its apical width 2.0 × width at level of hamuli (Fig. 120); 2-SC+R slightly longer than wide; m-cu short, postfurcal; M+CU:1-M = 61:36; 1r-m 0.75 × 1-M.

***Legs.*** Tarsal claws robust and with only brownish bristly setae (Fig. 131); hind coxa largely rather densely punctate; hind trochantellus robust; length of hind femur and basitarsus 3.6 and 4.5 × their width, respectively; length of inner hind spur 0.5 × hind basitarsus.

***Metasoma.*** First tergite rather flattened, 0.9 × as long as wide apically; 1^st^ and 2^nd^ tergites with medio-longitudinal carina and coarsely longitudinally rugose, but posterior quarter of 2^nd^ tergite irregularly rugose and no median carina; medio-basal area of 2^nd^ tergite triangular and short (Fig. 123); 2^nd^ suture deep and crenulate; basal third of 3^rd^ tergite finely longitudinally striate, remainder of metasoma superficially micro-sculptured; 4^th^ and apical half of 3^rd^ tergite without sharp lateral crease; ovipositor sheath wide, with long setae and apically truncate (Fig. 117).

***Colour.*** Dark orange brown; apical two-thirds of antenna, patch on hind femur dorso-apically, and telotarsi, dark brown; temple ventrally, malar space, mesosternum, mesopleuron, metapleuron, propodeum, pair of patches on 2^nd^ tergite and most of apical 0.4 of tergite, and 3^rd^–7^th^ tergites black; palpi (especially labial palp), veins and pterostigma dark brown, basal third of antenna (but scapus dorsally blackish) rather pale yellowish brown; tegulae and remainder of legs; yellowish brown; wings strongly infuscate.

***Variation.*** Antennal segments: ♀ 46(2), 49(1); ♂ 47(1), 50(1), 52(1), 54(1), 56(1), 57(1); length of fore wing of ♀ ca two-thirds of body length (0.8 × in ♂); males always darker than females; mainly black with legs mainly dark brown or blackish, but male from Austria has basal half of metasoma orange brown and legs partly yellowish brown. Males have 2^nd^ submarginal cell distinctly shorter than in females (as in *A. grassator*), antenna 0.9 × length of body and slightly less robust subapically, temple and face long setose and malar space 0.5–0.7 × length of eye in lateral view; metasoma black or 1–2 basal tergites reddish and apical tergites type 1, fringe not observed (Fig. 132).

##### Distribution.

Austria, Czech Republic, Hungary, *Russia (Lake Baikal).

##### Notes.

Very similar to *A. grassator* (Thunberg), and especially *A. carbonaroides*; males of *A. carbonarius* and *carbonaroides* are normally black but males with partly orange brown metasoma occur. The three species exhibit sexual dimorphism of the 2^nd^ submarginal cell (less robust (and also longer in *A. carbonarius*) in female than in male). Giraud (1857) gave an incomplete description of the only two males he possessed, but clearly indicated that the antenna is slightly shorter than the body. The female of this species is reported for the first time.

**Figures 116–118.**
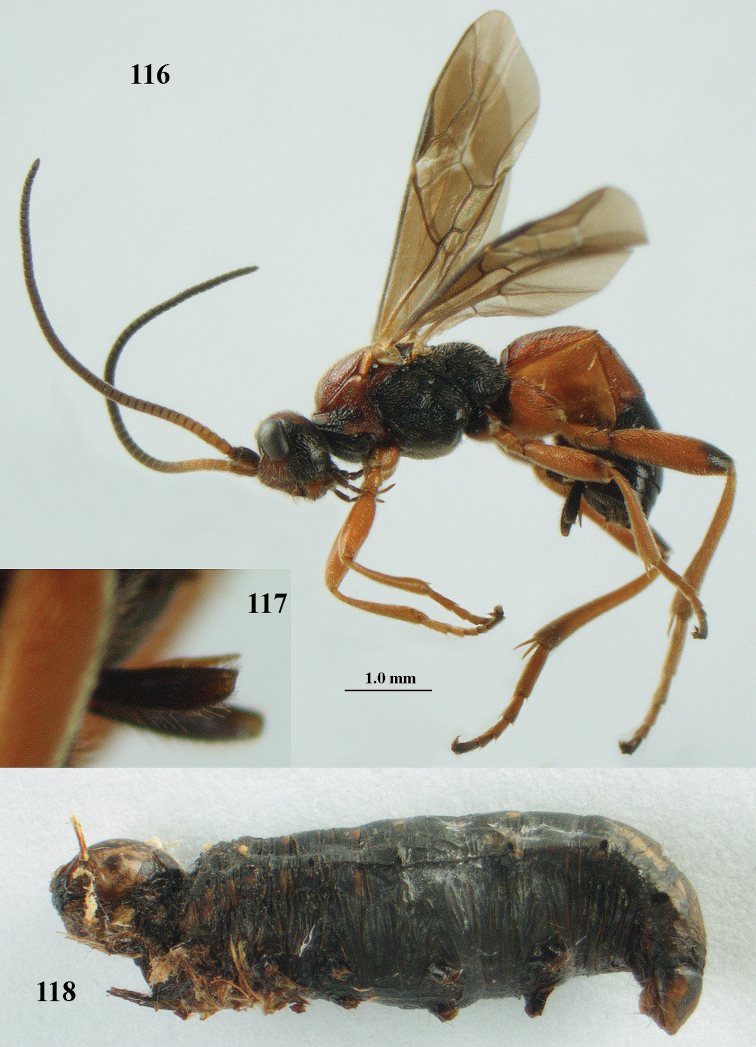
*Aleiodes carbonarius* Giraud, ♀, Hungary, Veszprém **116** habitus lateral **117** ovipositor sheath lateral **118** mummy of *Tholera decimalis* (Poda).

**Figures 119–131.**
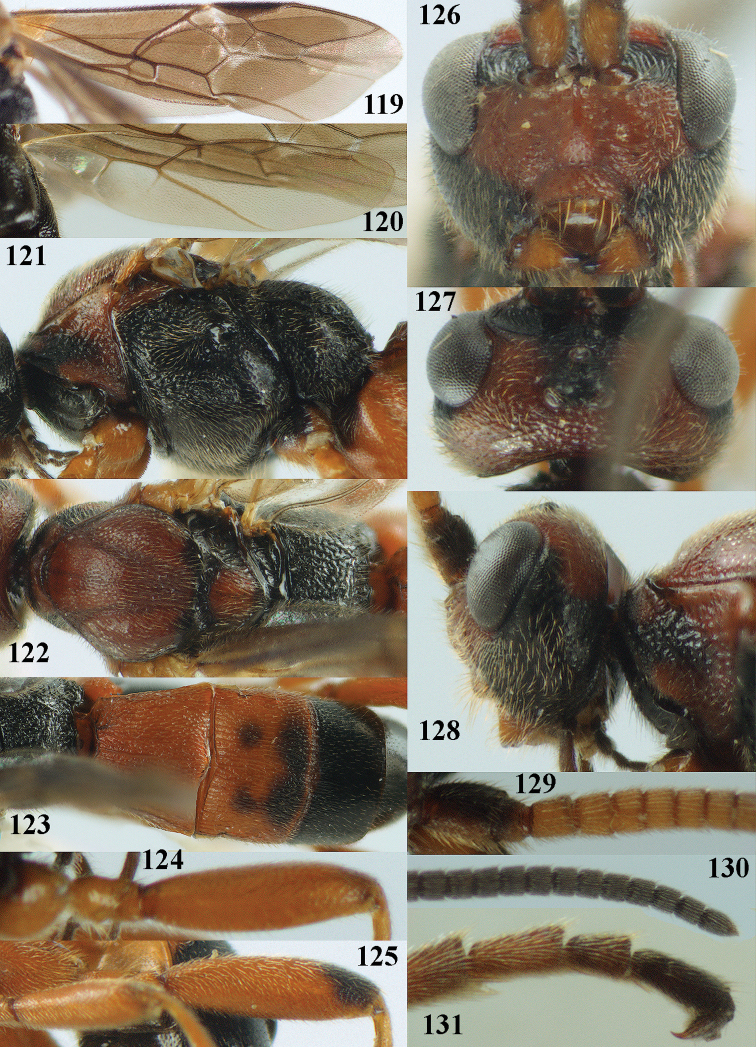
*Aleiodes carbonarius* Giraud, ♀, Hungary, Veszprém **119** fore wing **120** hind wing **121** mesosoma lateral **122** mesosoma dorsal **123** metasoma dorsal **124** fore femur lateral **125** hind femur lateral **126** head anterior **127** head dorsal **128** head lateral **129** base of antenna **130** apex of antenna **131** inner hind tarsal claw.

**Figures 132–137.**
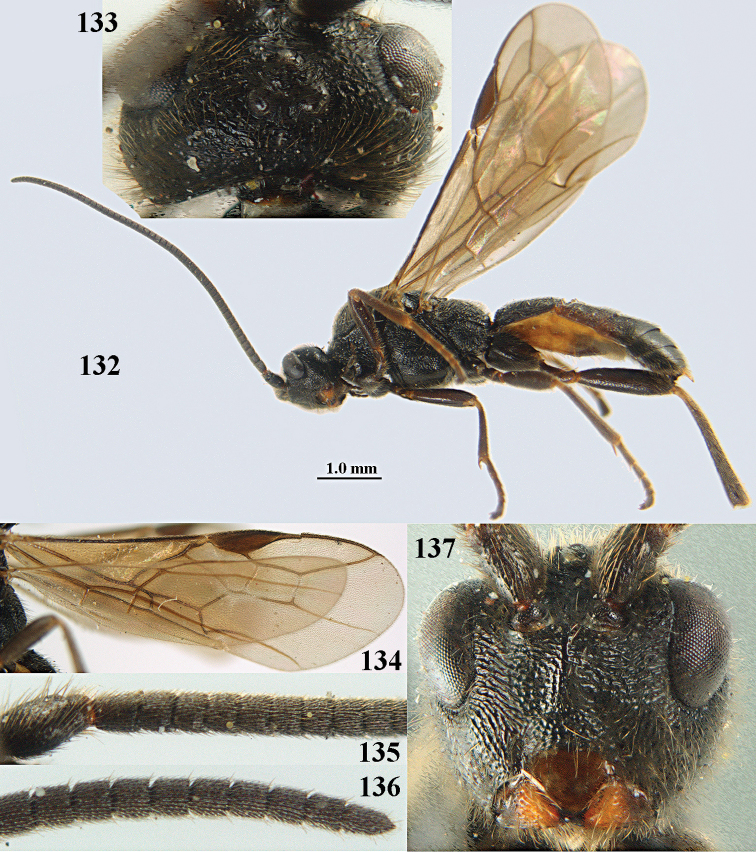
*Aleiodes carbonarius* Giraud, ♂, Hungary, Csákvár **132** habitus lateral **133** head dorsal **134** wings **135** base of antenna **136** apex of antenna **137** head anterior.

#### Aleiodes carbonaroides

Taxon classificationAnimaliaHymenopteraBraconidae

van Achterberg & Shaw sp. nov.

F9211028-9578-55D5-BA05-1033F2974422

http://zoobank.org/0BE2C69B-E310-4DFB-BE5C-07218AC6F018

Figs 138–141 , 142–153 , 154–160

##### Type material.

Holotype, ♀ (NMS), “[**Netherlands**: Friesland], Holland [sic!], Schiermonnikoog, em. 20.v.[19]82”, “ex *Cerapteryx graminis* larva”. Paratypes: 2 ♀ (NMS, RMNH), 3 ♂ (NMS, RMNH), topotypic and from same host, em. 19 or 20.v.1982; 1 ♂ (ZSSM) “[**Germany**], Münehey, 26.iv.[18]85 R7”, “1-653”; 2 ♂ (CMIM) “[**England**] 25.v.[19]22, Bdn. [= Brandon, Suffolk] HF”, “Named by Claude Morley 2 *Rhogas carbonarius* Giraud. NEW TO BRIT. CM V.22”; 1 ♂ (ZJUH) “[England], Totternhoe, [Bedfordshire], 30.v.[19]64 [V.H. Chambers]”. Sporadic in western Europe.

##### Molecular data.

None.

##### Biology.

Adults of this lowland species have been collected in April and May. The two paratypes from Suffolk were swept from Breck grassland (Morley, 1937, misidentified as *A. carbonarius*). Reared from the grass-feeding noctuid *Cerapteryx graminis* (Linnaeus) (6 [2 are RMNH]; K.P. Carl/Netherlands). If it is a specialist, it is presumably univoltine and overwinters in the mummy (the univoltine known host overwinters in the egg stage). Mummy similar to that of the closely related *A. carbonarius* and *A. grassator*, but slightly smaller.

##### Diagnosis.

Maximum width of hypoclypeal depression 0.4–0.5 × minimum width of face (Fig. 149); OOL of ♀ 1.8–2.0 × as long as diameter of posterior ocellus (Fig. 150) and distinctly rugose or rugulose; length of 4^th^ antennal segment of ♀ 0.7–0.9 × its width (Fig. 152; in ♂ up to 1.0 times); clypeus thick apically and not protruding anteriorly (Fig. 151); lobes of mesoscutum punctate, interspaces largely coriaceous and superficially coriaceous; precoxal area coarsely vermiculate-rugose medially; marginal cell of fore wing of ♀ ending rather removed from wing apex (Fig. 142); vein 1-CU1 of fore wing 0.5–0.6 × as long as vein 2-CU1 (Fig. 142); 2^nd^ submarginal cell of fore wing medium-sized (Fig. 142); hind tarsal claws slender and yellowish or brownish bristly setose; hind femur at least apico-dorsally dark brown or black; inner side of hind tibia of ♀ yellowish; head and mesoscutum of ♀ reddish; palpi and tegulae of ♀ brownish yellow; males entirely black, with palpi, tegulae and antenna dark brown or blackish.

##### Description.

Holotype, ♀, length of fore wing 4.2 mm, of body 7.1 mm.

***Head.*** Antennal segments of ♀ 45, 4^th^ segment 0.9 × longer than wide (Fig. 152); length of antenna 1.1 × fore wing, its subapical segments robust (Fig. 153) and scapus oblique apically; frons with coarse curved rugae and shiny; OOL 1.8 × diameter of posterior ocellus and rugulose; vertex rugose and shiny; clypeus coarsely punctate; ventral margin of clypeus thick and not protruding forwards (Fig. 151); width of hypoclypeal depression 0.4 × minimum width of face (Fig. 149); length of eye 1.2 × temple in dorsal view (Fig. 150); vertex behind stemmaticum rugose; clypeus below lower level of eyes; length of malar space 0.6 × length of eye in lateral view.

***Mesosoma.*** Mesoscutal lobes moderately punctate, interspaces superficially granulate-coriaceous and with satin sheen; precoxal area of mesopleuron coarsely rugose medially, but largely smooth posteriorly; remainder of mesopleuron mainly punctate; scutellum flat, sparsely finely punctate and with irregular lateral carina; propodeum coarsely rugose, medio-longitudinal carina complete, rounded posteriorly and dorsal part approx. as long as posterior part.

***Wings.*** Fore wing: r 0.4 × 3-SR (Fig. 142); marginal cell ends basad of level of apex of 3-M; 1-CU1 horizontal, 0.5 × 2-CU1; r-m 0.5 × 3-SR; 2^nd^ submarginal cell robust (Fig. 142), 3-SR 1.4 × as long as 2-SR; cu-a vertical, straight; 1-M slightly curved posteriorly; 1-SR similar to 1-M and medium-sized; surroundings of M+CU1, 1-M and 1-CU1 setose. Hind wing: marginal cell linearly widened, its apical width 1.7 × width at level of hamuli (Fig. 143); 2-SC+R subquadrate; m-cu short; M+CU:1-M = 27:15; 1r-m 0.7 × 1-M.

***Legs.*** Tarsal claws robust and with only brownish bristly setae (Fig. 140); hind coxa largely rugulose dorsally; hind trochantellus robust; length of hind femur and basitarsus 3.2 and 4.6 × their width, respectively; length of inner hind spur 0.4 × hind basitarsus.

***Metasoma.*** First tergite rather flattened, 0.7 × as long as wide apically; 1^st^ and 2^nd^ tergites with medio-longitudinal carina and coarsely longitudinally rugose, but posterior quarter of 2^nd^ tergite without medio-longitudinal carina; medio-basal area of 2^nd^ tergite triangular and short; 2^nd^ suture deep and crenulate; basal half of 3^rd^ tergite finely longitudinally rugose, remainder of metasoma superficially micro-sculptured; 4^th^ and apical third of 3^rd^ tergite without sharp lateral crease; ovipositor sheath wide, with long setae and apically truncate (Fig. 139).

***Colour.*** Dark orange brown; apical half of antenna, patch on hind femur dorso-apically, and telotarsi apically, dark brown; mesosternum, mesopleuron (except dorsally and postero-ventrally), metapleuron (except medio-dorsally), propodeum (except pair of posterior patches), 3^rd^–7^th^ tergites (except antero-lateral corners of 3^rd^ tergite) black; palpi, basal half of antenna, tegulae and remainder of legs rather pale yellowish brown; veins and pterostigma dark brown; wings strongly infuscate but hind wing less than fore wing.

***Variation.*** Basal third or half of antenna of ♀ pale yellowish brown; vein 3-SR 1.4–1.6 × as long as vein 2-SR; hind femur of ♀ 3.2–3.5 × longer than wide; 1^st^ metasomal tergite 0.7–0.8 × its apical width; temple and occiput ventrally, and malar space ventrally orange brown or black. Antennal segments: ♀ 43(1), 45(1); ♂ 48(1), 49(2), 51(1), 50(1), 53(2); males clearly have many more antennal segments than females. Males are much darker than females; body black with palpi and legs mainly dark brown or blackish (Fig. 154). Males have 2^nd^ submarginal cell slightly smaller than females (Fig. 158), temple and face long setose, malar space 0.5–0.7 × length of eye in lateral view, and apical tergites type 1 and fringe not observed (Fig. 154); sometimes superficial granulosity of 3^rd^ tergite and of mesoscutum are absent.

##### Distribution.

Germany, Netherlands, U.K.

##### Etymology.

The suffix “-oides” indicates similar to; in this case the high similarity to *A. carbonarius* Giraud.

**Figures 138–141.**
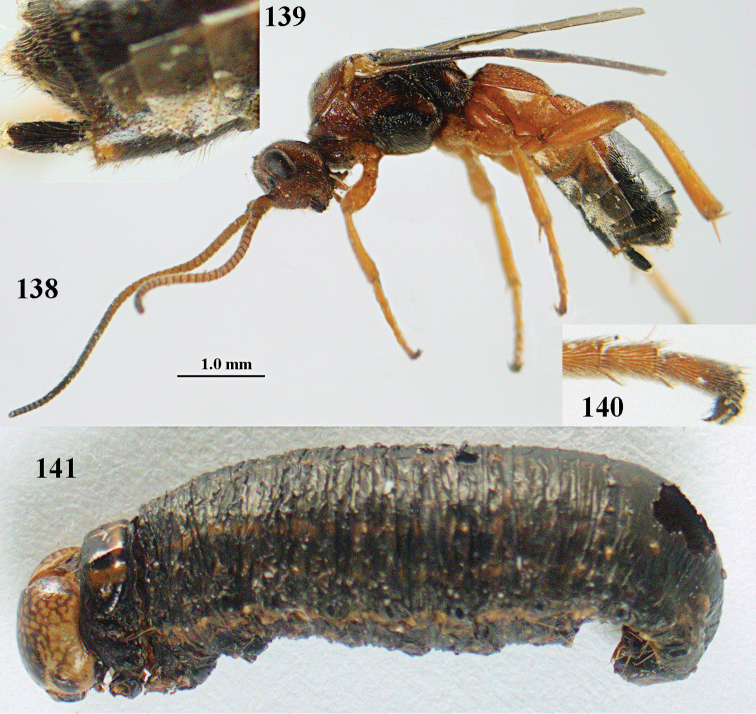
*Aleiodes carbonaroides* sp. nov., ♀, holotype **138** habitus lateral **139** ovipositor sheath lateral **140** outer hind tarsal claw lateral **141** mummy of *Cerapteryx graminis* (Linnaeus).

**Figures 142–153.**
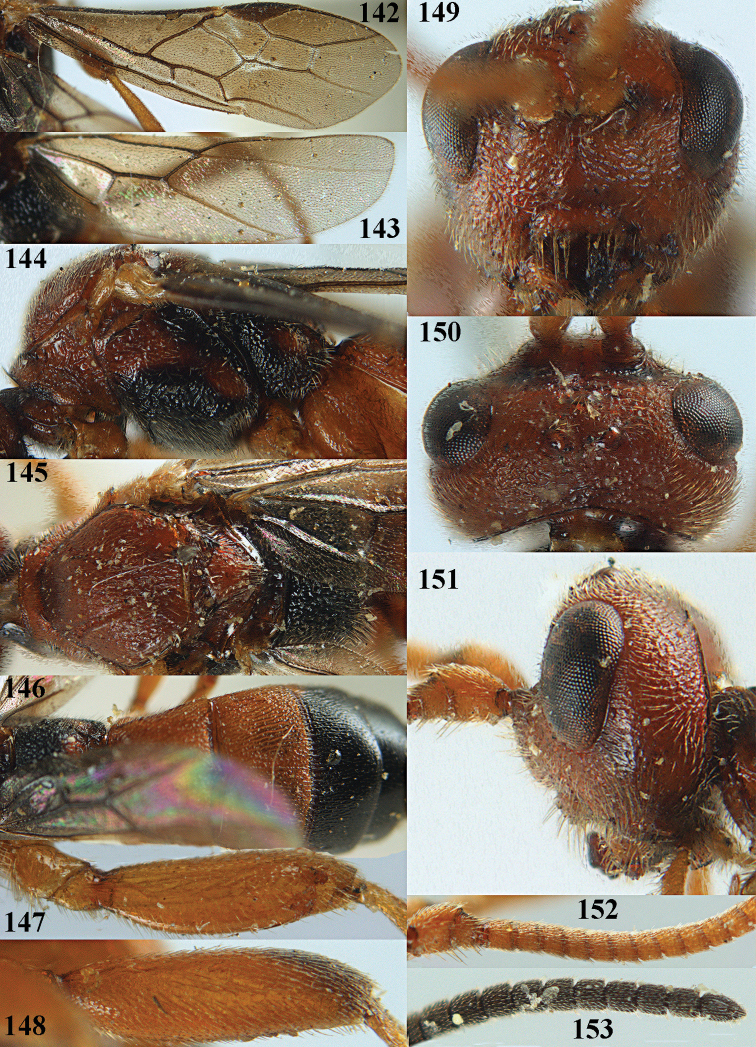
*Aleiodes carbonaroides* sp. nov., ♀, holotype **142** fore wing **143** hind wing **144** mesosoma lateral **145** mesosoma dorsal **146** metasoma dorsal **147** fore femur lateral **148** hind femur lateral **149** head anterior **150** head dorsal **151** head lateral **152** base of antenna **153** apex of antenna.

**Figures 154–160.**
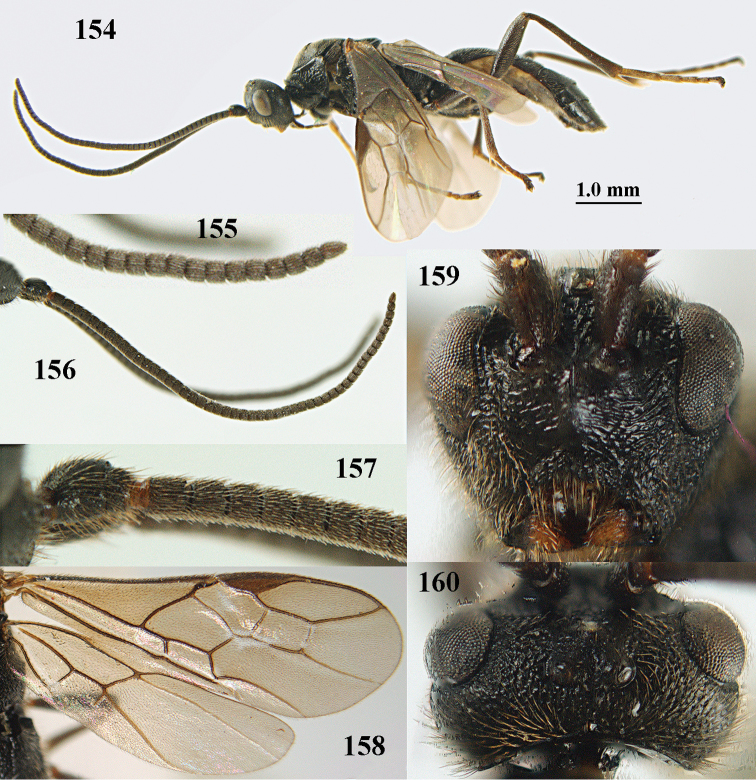
*Aleiodes carbonaroides* sp. nov., ♂, paratype **154** habitus lateral **155** apex of antenna **156** antenna **157** base of antenna **158** wings lateral **159** head anterior **160** head dorsal.

#### Aleiodes caucasicus

Taxon classificationAnimaliaHymenopteraBraconidae

(Tobias, 1976)

F81E320E-FEC2-5292-ACFC-800C6F9C8DEE

Figs 161–163 , 164–177

Rogas (Rogas) caucasicus Tobias, 1976: 86, 222, 1986: 81 (transl.: 133) [examined].
Aleiodes (Neorhogas) caucasicus ; Papp 1985a: 152.
Aleiodes caucasicus ; Papp 1991a: 75 (as synonym of A. fortipes), 2005: 176 (id.); Fortier and Shaw 1999: 227; Žikić et al. 2002: 108; Aydogdu and Beyarslan 2005: 191.

##### Type material.

Holotype, ♀ (ZISP), “[**Russia**], Sotchi, Lazarevskoe [terras], 26.iv.[1]973, V. Tobias”, “Holotypus *Rogas caucasicus* Tobias”; 2 ♀, paratype (MTMA), id., but 29.iv.1973.

##### Additional material.

Figured ♀ (NMS), “[**Russia**], Sotchi, Lazarevskoe terras. Sklony, les [= forest], 25.iv.1988, V. Tobias”, “*Rogas caucasicus* Tob.”, “*Aleiodes caucasicus* (Tobias), det. Belokobylskij, 2005. ♀ Ant. 40”; 2 ♀ (ALC, RMNH), id., but 7.v.1975; 1 ♀ (MTMA), “**Bulgaria**”, “Rhodopi, St[ara] Zagora, 17.iv.1977, J. Kolarov”, “*Rogas* sp. n.?, det. Zaykov, 1983”, “*Aleiodes fortipes* Rh. ♀, det. Papp J., 1985”.

##### Molecular data.

None.

##### Biology.

Unknown. Specimens collected in April-May and flight time probably April–May. We have not seen reared material. Probably, like *A. fortipes*, it will be found to be univoltine, overwintering in the mummy, but direct evidence is lacking.

##### Diagnosis.

Maximum width of hypoclypeal depression approx. 0.3 × minimum width of face (Fig. 171); antenna of ♀ with 38–41 segments and 2^nd^ – 10^th^ antennal segments yellowish, contrasting with remaining segments; OOL coarsely transversely striate; clypeus obtuse apically and not protruding in lateral view (Fig. 173); precoxal area finely striate (Fig. 166); tegulae yellow; lobes of mesoscutum finely coriaceous-granulate and rather dull, with satin sheen; vein 1-CU1 of fore wing much shorter than vein 2-CU1 (Fig. 164); posteriorly vein m-cu of fore wing diverging from anterior half of vein 1-M; length of hind femur 3.6–3.8 × its maximum width (Fig. 170); hind tarsal claws brownish setose (Fig. 177); length of fore wing 3.7–5.0 mm. Very similar to *A. fortipes* (Reinhard) and differs mainly by its body colour and sculpture of mesopleuron.

##### Description.

Holotype, ♀, length of fore wing 3.7 mm, of body 4.6 mm.

***Head.*** Antennal segments of ♀ 41, length of antenna 1.3 × fore wing, its subapical segments rather robust; frons largely finely rugulose medially; OOL 2.2 × diameter of posterior ocellus, and coarsely transversely striate; vertex transversely striate and rather shiny; clypeus rugulose, but ventrally depressed and smooth; ventral margin of clypeus thick and not protruding forwards (Fig. 173); width of hypoclypeal depression 0.3 × minimum width of face (Fig. 171); length of eye twice temple in dorsal view (Fig. 172); vertex behind stemmaticum rugulose; clypeus below lower level of eyes; length of malar space 0.6 × length of eye in lateral view; occipital carina largely absent dorsally and weakly developed ventrally.

***Mesosoma.*** Mesoscutal lobes largely rugulose-granulate, rather matt; precoxal area of mesopleuron transversely striate medially, distinctly rugose antero-dorsally and remainder largely punctulate; pleural sulcus moderately crenulate (Fig. 166); ventral half of metapleuron rugose; metanotum with nearly complete median carina; scutellum coriaceous; propodeum densely and finely granulate-rugose and medio-longitudinal carina medium-sized.

***Wings.*** Fore wing: r 0.6 × 3-SR; 1-CU1 horizontal, 0.5 × 2-CU1; r-m unsclerotized and 0.7 × 3-SR; 2^nd^ submarginal cell medium-sized (Fig. 164); cu-a vertical, straight and rather short; 1-M slightly curved posteriorly; posteriorly vein m-cu diverging from anterior half of vein 1-M. Hind wing: marginal cell linearly widened, its apical width 2.0 × width at level of hamuli (Fig. 165); 2-SC+R subquadrate; m-cu absent; M+CU:1-M = 5:3; 1r-m 0.7 × 1-M.

***Legs.*** Tarsal claws robust and with brownish bristles (Fig. 177); hind coxa densely rugulose and rather dull; hind trochantellus robust; length of hind femur and basitarsus 3.6 and 5.0 × their width, respectively; length of inner hind spur 0.4 × hind basitarsus.

***Metasoma.*** First tergite evenly convex, 0.9 × longer than wide apically; 1^st^ and 2^nd^ tergites with indistinct medio-longitudinal carina and coarsely longitudinally rugose, but posterior quarter of 2^nd^ tergite irregularly rugose and no median carina; medio-basal area of 2^nd^ tergite triangular and rather distinct (Fig. 168); 2^nd^ suture rather shallow and crenulate; medio-basally 3^rd^ tergite striate, remainder of metasoma superficially micro-sculptured; 4^th^ and apical half of 3^rd^ tergite without sharp lateral crease; ovipositor sheath wide, with long setae and apically truncate (Fig. 162).

***Colour.*** Orange brown; head, 3^rd^ tergite (except antero-laterally) and subsequent tergites black; scapus, pedicellus basally, 11^th^ and following antennal segments, palpi, veins, parastigma, pterostigma and femora apico-dorsally, tibia and tarsal segments apically, ventral half of metasoma and ovipositor sheath dark brown; tegulae, 3^rd^–10^th^ antennal segments brownish yellow; wing membrane subhyaline.

***Variation.*** Head black or mainly dark brown, specimen from Bulgaria also anterior half of mesosoma; antenna of ♀ with 38 or 41 segments according to the original description; 11^th^ and 12^th^ antennal segments of ♀ dark brown or brownish yellow; hind femur 3.6–3.8 × as long as wide. The male is unknown, or possibly has not been distinguished from that of *A. fortipes*.

##### Distribution.

*Bulgaria, Russia (SW).

##### Notes.

It remains unclear whether this predominantly rather yellowish orange species is distinct from *A. fortipes*, which in its more western localities is a much darker insect. Females intermediate in colour (and included in *A. fortipes*) seem to predominate in eastern Europe. More material (preferably with biological data) is needed to clarify the status of *A. caucasicus*.

**Figures 161–163.**
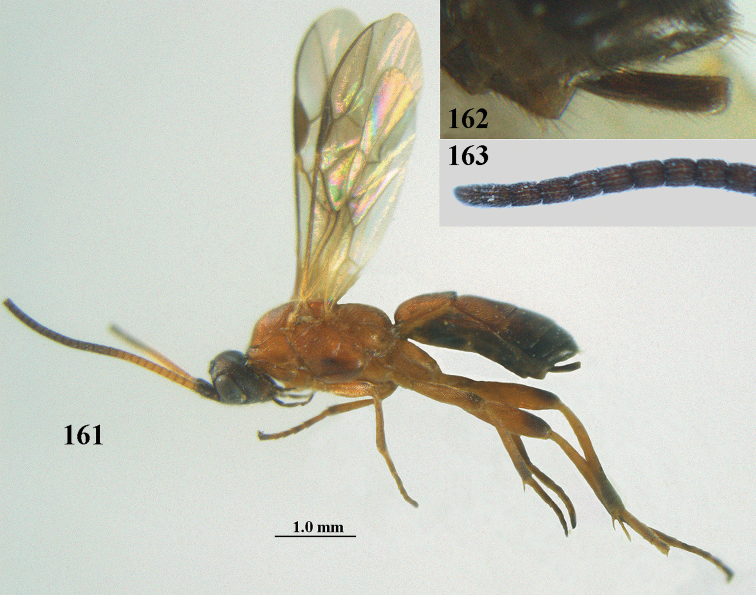
*Aleiodes caucasicus* (Tobias), ♀, Russia, Sotchi **161** habitus lateral **162** ovipositor sheath lateral **163** apex of antenna (of paratype).

**Figures 164–177.**
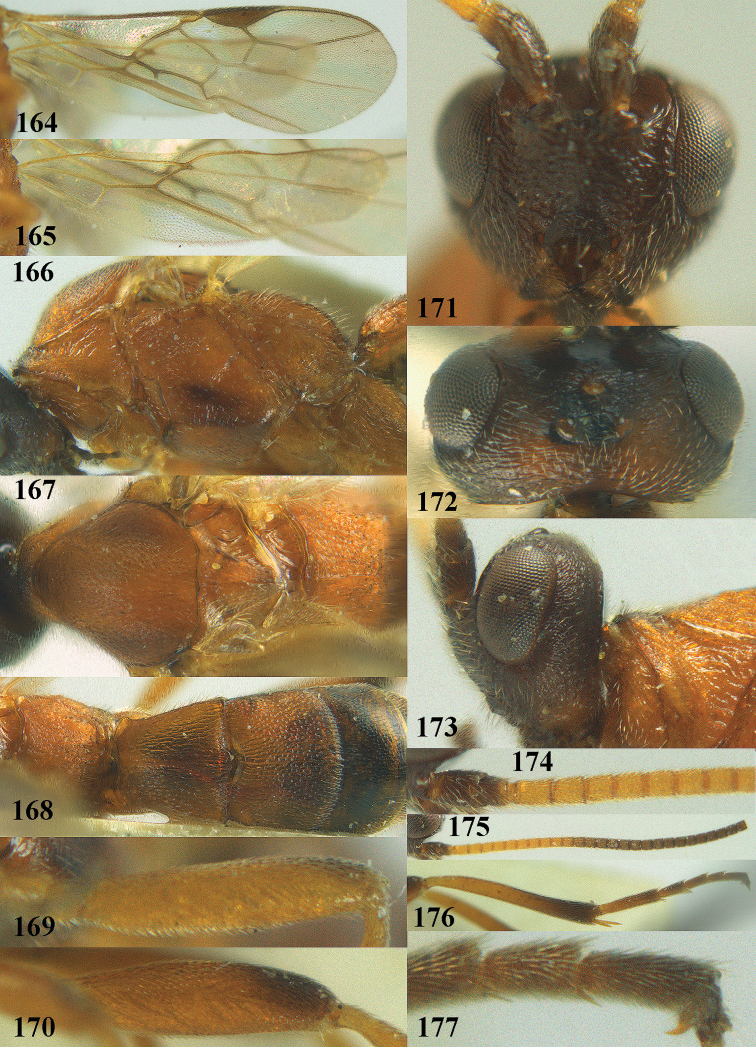
*Aleiodes caucasicus* (Tobias), ♀, Russia, Sotchi **164** fore wing **165** hind wing **166** mesosoma lateral **167** mesosoma dorsal **168** metasoma dorsal **169** fore femur lateral **170** hind femur lateral **171** head anterior **172** head dorsal **173** head lateral **174** base of antenna **175** antenna **176** hind tibia and tarsus lateral **177** outer hind tarsal claw.

#### Aleiodes coriaceus

Taxon classificationAnimaliaHymenopteraBraconidae

van Achterberg & Shaw sp. nov.

29D9CDFF-ED7A-537C-8452-99B316FC6122

http://zoobank.org/EA99A74A-AA7C-460F-85F5-AC7405FE67B9

Figs 178–179 , 180–191 , 192–195

##### Type material.

Holotype, ♀ (NMS), “**Sweden**: Hr, Sveg, Duybergshammaren, 17.vii.2004, N. Ryholm, NMSZ 2004.167”, “MRS *Aleiodes* DNA 377”, “COI worked”. Paratypes: 1 ♂ (NMS), same label data as holotype; 1 ♀ (RMNH), “Sweden: Ås. Lilla, Vammasj. Window trap on *Betula* F2, 8, vii.2003, J. Hilszczanski”, “MRS *Aleiodes* DNA 311”, “COI worked”.

##### Molecular data.

MRS311 (Sweden), MRS377 (Sweden).

##### Biology.

Unknown. The available specimens were collected in July, and it is almost certainly univoltine, but we have not seen reared material.

##### Diagnosis.

**Figures 178, 179.**
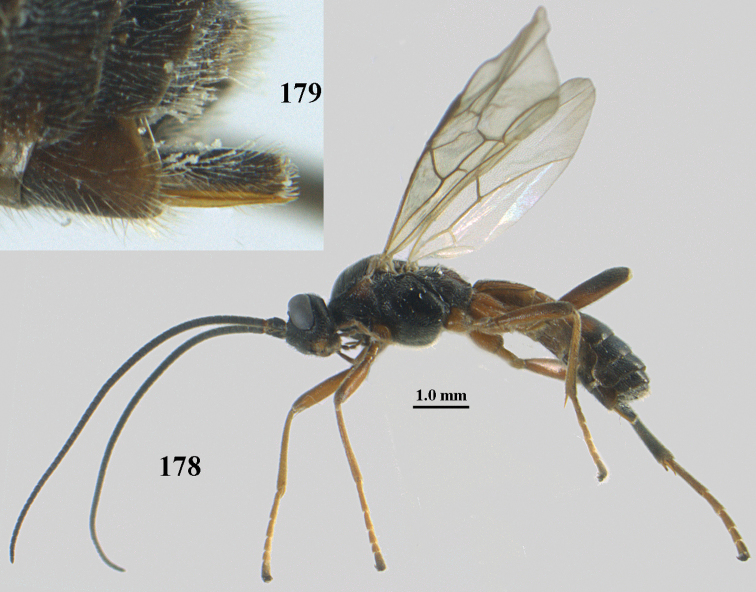
*Aleiodes coriaceus* sp. nov., ♀, holotype **178** habitus lateral **179** ovipositor sheath lateral.

**Figures 180–191.**
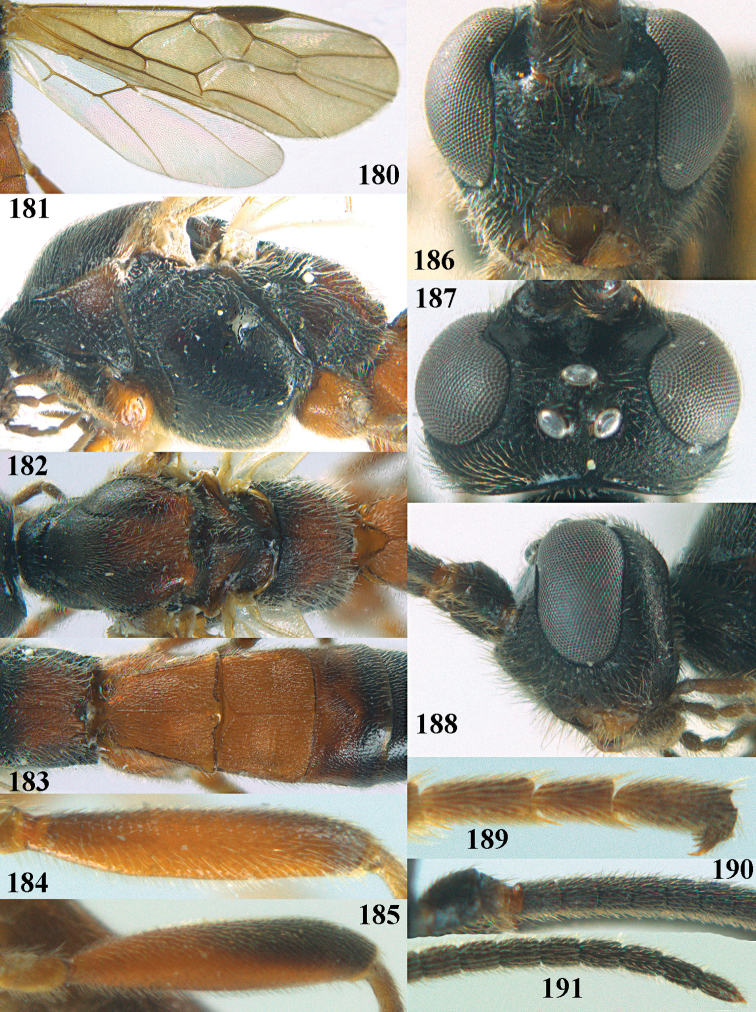
*Aleiodes coriaceus* sp. nov., ♀, holotype **180** wings **181** mesosoma lateral **182** mesosoma dorsal **183** propodeum and 1^st^–3^rd^ metasomal tergites dorsal **184** fore femur lateral **185** hind femur lateral **186** head anterior **187** head dorsal **188** head lateral **189** outer hind tarsal claw **190** base of antenna **191** apex of antenna.

Maximum width of hypoclypeal depression approx. 0.4 × minimum width of face (Fig. 186); OOL of ♀ 0.9–1.1 × as long as diameter of posterior ocellus (Fig. 187), and rugulose-coriaceous or only coriaceous; ventral margin of clypeus rather thin or blunt and not protruding forwards (Fig. 188); vertex mainly coriaceous and rather dull; mesoscutal lobes coriaceous and largely matt; scutellum remotely punctate; area of precoxal sulcus largely smooth, with some punctulation; length of vein 1-CU1 of fore wing 0.3–0.4 × vein 2-CU1 and 0.4–0.5 × vein m-cu; marginal and 2^nd^ submarginal cells of fore wing elongate (Fig. 180); tarsal claws with robust apical tooth and with medium-sized dark brown pecten (Fig. 190); hind femur and basitarsus slender (Figs 178, 185); 1^st^ metasomal tergite comparatively steep anteriorly (Fig. 178); basal half of 3^rd^ tergite with posteriorly diverging rugulae; head black; dorsal half of hind femur largely black dorsally; basal half of hind tibia largely dark brown; fore and middle trochanters and trochantelli infuscate or dark brown; 2^nd^ tergite yellowish or reddish and rather slender (Fig. 183); 5^th^–7^th^ tergites of ♂ medially glabrous and convex, and laterally with long setae (Figs 194, 195). Closely related to *A. rufipes* (Thomson) and differs mainly by the sculpture of the mesoscutum (matt instead of rather shiny), darker colour of legs, different COI and less robust 2^nd^ and 3^rd^ metasomal tergites.

##### Description.

Holotype, ♀, length of fore wing 6.1 mm, of body 6.7 mm.

***Head.*** Antennal segments of ♀ 54, antenna 1.1 × as long as fore wing, its basal segments robust, subapical segments medium-sized and apical segment with spine; frons largely smooth, except for some micro-sculpture; OOL 0.9 × diameter of posterior ocellus, rugulose-coriaceous and rather dull, groove beside posterior ocellus deep and smooth; vertex coriaceous with some rugulae, rather dull; face transversely rugose; clypeus densely rugulose; ventral margin of clypeus thin and not protruding forwards (Fig. 188); width of hypoclypeal depression 0.4 × minimum width of face (Fig. 186); length of eye 2.1 × temple in dorsal view (Fig. 187); vertex behind stemmaticum coriaceous; clypeus partly above lower level of eyes; length of malar space 0.3 × length of eye in lateral view.

***Mesosoma.*** Mesoscutal lobes largely coriaceous and matt; precoxal area of mesopleuron partly remotely punctulate and superficially micro-sculptured; medio-longitudinal carina of metanotum distinct posteriorly; scutellum punctate and with lateral carina; propodeum convex and rugose, medio-longitudinal carina absent posteriorly, and without protruding carinae laterally.

***Wings.*** Fore wing: r 0.35 × 3-SR (Fig. 180); 1-CU1 slightly oblique, 0.35 × 2-CU1; r-m 0.4 × 3-SR; 2^nd^ submarginal cell long (Fig. 180); cu-a slightly inclivous, straight but posteriorly slightly curved; 1-M nearly straight posteriorly; 1-SR widened; surroundings of M+CU1, 1-M and 1-CU1 densely setose. Hind wing: marginal cell linearly widened, its apical width 2.3 × width at level of hamuli (Fig. 180); 2-SC+R slightly longer than wide; m-cu absent; M+CU:1-M = 50:46; 1r-m 0.6 × 1-M.

***Legs.*** Tarsal claws with rather conspicuous and medium-sized dark brown pecten (Fig. 189); hind coxa (except depression) coriaceous and with some rugulae dorsally; hind trochantellus robust and with long setae; length of hind femur and basitarsus 4.5 and 5.8 × their width, respectively; length of inner hind spur 0.5 × hind basitarsus.

***Metasoma.*** First tergite convex and basally rather steep, as long as wide apically; 1^st^ and 2^nd^ tergites with medio-longitudinal carina and longitudinally rugose; maximum width of 2^nd^ tergite 1.5 × its median length; medio-basal area of 2^nd^ tergite medium-sized triangular and rather short (Fig. 183); 2^nd^ suture distinct and moderately crenulate; basal half of 3^rd^ tergite finely rugulose and rugulae diverging posteriorly, remainder of metasoma nearly smooth; 4^th^ and apical half of 3^rd^ tergite without sharp lateral crease; ovipositor sheath wide, with long setae and apically truncate (Fig. 179).

***Colour.*** Black; mesoscutum posteriorly, legs (but fore and middle telotarsi, fore and middle femora basally and apically, fore and middle trochanters and trochantelli, hind tarsus dark brown or infuscate, posterior half of hind femur dorsally and hind tibia largely blackish), propodeum and 1^st^ –3^rd^ metasomal tergites (but posterior half of 3^rd^ tergite blackish posteriorly) reddish brown; tegulae brownish yellow, but humeral plate largely dark brown; palpi, pterostigma and veins dark brown; wing membrane slightly infuscate.

***Variation.*** Antennal segments: ♀ 52(1), 54(1); ♂ 53(1). Length of fore wing 5.3–6.1 mm. Male is very similar to female (Figs 195–195). Apical tergites of male type 1–2, and fringe scarcely visible in the single male seen.

##### Distribution.

Sweden.

##### Etymology.

*Coriaceus* is Latin for leathery, because of the coriaceous sculpture of vertex and mesoscutum.

**Figures 192–195.**
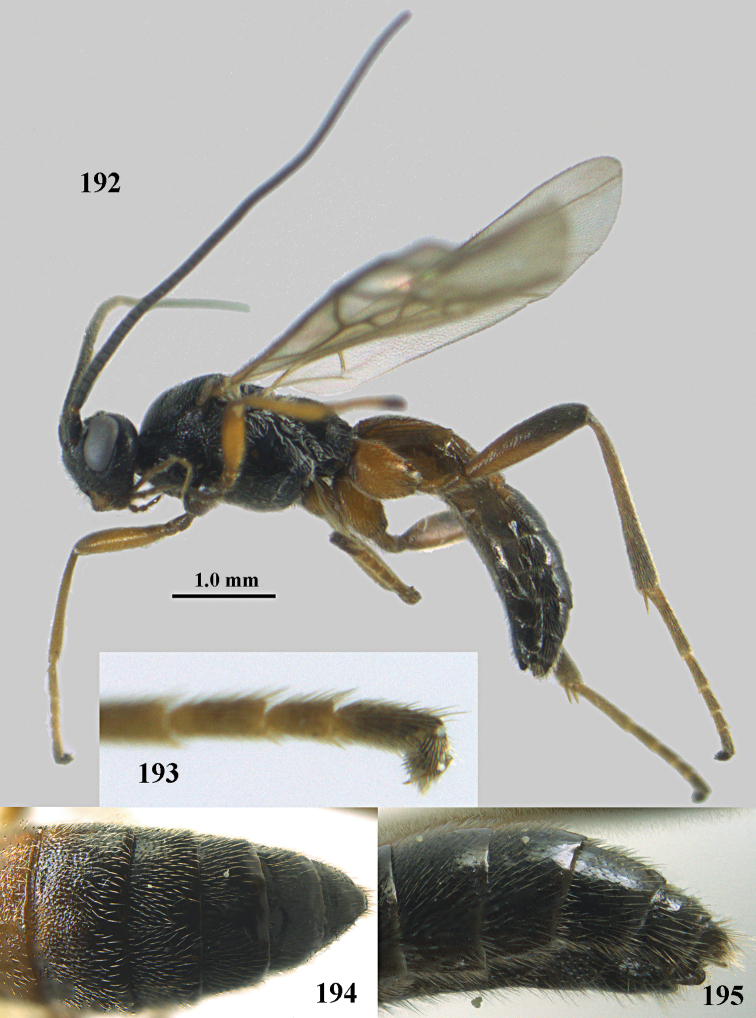
*Aleiodes coriaceus* sp. nov., ♂, paratype **192** habitus lateral **193** inner hind claw lateral **194** 3^rd^–7^th^ metasomal tergites dorsal **195** 3^rd^ –7^th^ metasomal tergites lateral.

#### Aleiodes cruentus

Taxon classificationAnimaliaHymenopteraBraconidae

(Nees, 1834)

29199599-262E-5DC0-AB00-C26D2EC2466D

Figs 196–198 , 199–211 , 212–216

Rogas cruentus Nees, 1834: 212; Shenefelt 1975: 1222; Zaykov 1980a: 112; Kotenko 1992: 96.
Rogas (Rogas) cruentus ; Tobias 1976: 85, 1986: 80 (transl.: 130).
Aleiodes (Neorhogas) cruentus ; Papp 1985a: 156–157 (neotype designation), 1987b: 35, 1991a: 83; Belokobylskij 1996: 6; Riedel et al. 2002: 106.
Aleiodes (Chelonorhogas) cruentus ; Chen and He 1997: 39; Belokobylskij 2000: 32.
Aleiodes cruentus ; Bergamasco et al. 1995: 5; Belokobylskij et al. 2003: 398; Papp 2005: 176.
Rhogas cruentus ab. nigricans Fahringer, 1932: 238; Papp 1991a: 83 (invalid name).
Rhogas cruentus ab. basalis Hellén, 1927: 22 (invalid name).
Rhogas cruentus ab. nigromaculata Hellén, 1927: 22 (invalid name).
Rhogas cruentus ab. rufofasciata Hellén, 1927: 22 (invalid name).
Rogas dorsalis Herrich-Schäffer, 1838: 154; Shenefelt 1975: 1222 (as synonym of A. cruentus); Papp 2005: 176 (id.).
Rogas affinis Herrich-Schäffer, 1838: 124 (key only); Shenefelt 1975: 1174–1175 [neotype designated below]. Syn. nov.
Aleiodes affinis ; Belokobylskij et al. 2003: 398.

##### Type material.

Neotype of *A. affinis* here designated, ♀ (RMNH), “Museum Leiden, **Nederland**, Melissant (ZH), [at light], 10.viii.1980, K.J. Huisman”. It is important for nomenclatorial stability to fix our interpretation of *A. affinis* because the types of Braconidae described by Herrich-Schäffer are lost (Horn and Kahle 1935–37; the first author could not find any specimen in ZMB), the original description is rudimentary and there are very similar species in Europe. The specimen from Netherlands is selected because it fits best the original description, Netherlands is relatively close to the probable German (but unknown) type location and it is in good condition. Another complication is that the neotype of *A. cruentus* by Papp (1985) is an old male from uncertain origin in the Gravenhorst Collection (Wroclaw).

##### Additional material.

Austria, Bulgaria, Croatia, Czech Republic, Finland, France, Germany, Greece, Italy (including Sicily), Moldova, Netherlands (FR: Ried, GE: Beusichem; Heerde; Voorst (Twello), LI: Thorn, NB: Eindhoven; Tilburg (Kaaistoep), OV: Buurse; Hasselt, ZH: Lexmond; Melissant; Middelharnis; Oostvoorne, ZL: Oostkapelle), Norway, Romania, Slovakia, Slovenia, Spain, Sweden, Ukraine, [Mongolia]. Specimens in ZJUH, BZL, FMNH, HSC, IKC, MSC, MTMA, NMS, NRS, RMNH, SDEI, ZSSM. Widespread in the region but rather sporadic. The specimen (CMIM) from which Morley (1915) recorded this species as new to Britain has been examined and proves to be *A. alternator* (Nees). A further specimen in CMIM recorded by Lyle (1919) as *A. cruentus* has been examined and belongs to *A. diversus* (Szépligeti), q. v., as do another three British specimens in ZJUH and one in NMS, and there is no evidence that *A. cruentus* has ever occurred in Britain.

##### Biology.

Probably univoltine, certainly overwintering as a mummy. Collected June-August, often at light and including around *Dianthus barbatus* harbouring larvae of the noctuid *Hadena confusa* (Hufnagel) (H. Schnee/Germany). In Austria it has been collected up to 2000 m. Only one reared specimen seen, from *H. confusa* [FMNH], the adult emerging in June in the year following host mummification. Extensive rearings of this host in various parts of Britain in recent years by one of us (MRS) has not produced *A. cruentus*, strengthening the view that it does not occur in Britain. The predominantly dark mummy seen (Fig. 198) is stout, rather short and weakly swollen dorsally, and has a paler and moderately strong lateral keel. The cocoon is substantially silk-lined and occupies most of the host’s abdomen (approx. 2^nd^–7^th^ abdominal segments). The mummy probably forms underground, albeit from penultimate instar hosts, and the somewhat reflexed and sideways twisted head suggests that it is not or scarcely stuck down; the caudal segments are also somewhat recurved ventrally. Although oviposition has not been witnessed, the somewhat laterally compressed apex of the female’s metasoma appears to be an adaptation for attacking the host at rest or feeding within the seed capsules of its food plants (*Dianthus*, *Silene*, etc.).

##### Molecular data.

MRS558 (France), MRS624 (Germany), MRS625 (Germany).

##### Diagnosis.

Maximum width of hypoclypeal depression (0.5–)0.6–0.7 × minimum width of face (Fig. 206); OOL of ♀ coarsely punctate and 0.5–0.8(–1.0) × diameter of posterior ocellus; ventral margin of clypeus (rather) obtuse apically and not protruding (Fig. 208), but sometimes intermediate; length of eye 1.5–1.9 × temple in dorsal view; lobes of mesoscutum densely finely punctate, with interspaces approx. equal to diameter of punctures; precoxal area with some rugae medially; vein cu-a of fore wing vertical; surroundings of veins M+CU1 and 1-+2-CU1 largely glabrous; vein r of fore wing 0.3–0.4 × vein 3-SR (Fig. 199); vein 1-CU1 of fore wing 0.8–1.1 × vein 2-CU1 (Fig. 199), rarely shorter; hind tarsal claws with conspicuous dark brown pecten (Fig. 205); 1^st^ tergite widened apically; 2^nd^ tergite 0.7–0.9 × as long as wide (Fig. 202), its colour variable, often reddish; head black; vein 1-M of fore wing brownish; wing membrane subhyaline; 4^th^–6^th^ tergites of ♂ with long setae, but flattened and narrowly glabrous medially.

##### Description.

Neotype of *A. affinis*, ♀, length of fore wing 7.3 mm, of body 10.2 mm.

***Head.*** Antennal segments of ♀ 61, length of antenna 1.2 × fore wing, its subapical segments rather robust; frons largely smooth and shiny, but rugulose near stemmaticum; OOL 0.6 × diameter of posterior ocellus, and coarsely punctate, interspaces approx. equal to diameter of punctures; vertex mainly densely punctate, shiny; clypeus coarsely punctate-rugose; ventral margin of clypeus thick and not protruding forwards (Fig. 208); width of hypoclypeal depression 0.6 × minimum width of face (Fig. 206); length of eye 1.9 × temple in dorsal view and temple rather long and densely setose (Fig. 207); vertex behind stemmaticum punctate-rugose; clypeus near lower level of eyes; length of malar space 0.2 × length of eye in lateral view (Fig. 208).

***Mesosoma.*** Mesoscutal lobes densely and finely punctate, with satin sheen; precoxal area of mesopleuron with some rugae medially, rather densely punctate anteriorly and posteriorly; metapleuron mainly sparsely punctate, shiny; scutellum rather weakly punctate and slightly convex; propodeum evenly convex and coarsely rugose, medio-longitudinal carina complete and straight.

***Wings.*** Fore wing: r 0.4 × 3-SR (Fig. 199); 1-CU1 horizontal, as long as 2-CU1; r-m 0.7 × 3-SR; 2^nd^ submarginal cell rather short (Fig. 199); cu-a vertical, straight; 1-M slightly curved posteriorly; 1-SR wide; anterior half of subbasal and of subdiscal cells largely glabrous. Hind wing: basal half of marginal cell slightly widened, but apical half wide, apical width of cell 2.5 × width at level of hamuli (Fig. 199); 2-SC+R subquadrate; m-cu short and obsolescent; surroundings of M+CU and 1-M glabrous; M+CU:1-M = 75:47; 1r-m 0.8 × 1-M.

***Legs.*** Tarsal claws with conspicuous and robust dark brown pecten (Fig. 205); hind coxa largely punctate; hind trochantellus robust; length of hind femur and basitarsus 4.3 and 5.2 × their width, respectively; length of inner hind spur 0.5 × hind basitarsus.

***Metasoma.*** First tergite rather flattened, as long as wide apically; 1^st^ and 2^nd^ tergites with medio-longitudinal carina and largely coarsely longitudinally rugose, but posterior quarter of 2^nd^ tergite irregularly rugose and no median carina; medio-basal area of 2^nd^ tergite triangular and rather distinct (Fig. 202); 2^nd^ suture deep medially, shallow laterally and crenulate; 2^nd^ tergite 0.7 × as long as wide (Fig. 202); anterior 0.7 of 3^rd^ tergite densely and finely punctate, remainder of metasoma largely smooth; 4^th^ and apical half of 3^rd^ tergite without sharp lateral crease; ovipositor sheath wide, with rather long setae and apically rather rounded (Fig. 197).

***Colour.*** Black; posterior half of mesoscutum, scutellum largely, apical rim of 1^st^ tergite and basal rim of 2^nd^ tergite reddish brown; fore coxa, bases of middle and hind coxae blackish; apex of hind tibia, telotarsi, hind tarsus, palpi, veins and pterostigma dark brown; tegulae and remainder of hind tibia pale yellowish; remainder of legs reddish brown; wing membrane subhyaline.

***Variation.*** Vein 1-CU1 of fore wing 0.8–1.1 × as long as 2-CU1; mesoscutum, scutellum, metanotum, 1^st^ and 2^nd^ metasomal tergites are most often entirely reddish or orange brown but variably partly blackish, in particular 1^st^ tergite sometimes with dark medial patch; pronotum and mesopleuron black or reddish dorsally; parastigma narrowly dark brown or yellowish brown; coxae entirely reddish to entirely dark brown. Antennal segments: ♀ 53(1), 55(1), 56(3), 57(5), 58(9), 59(9), 60(10), 61(9), 62(3), 63(1), 65(2), 67(1). ♂ 60(6), 61(7), 62(2), 63(5), 64(3), 65(1), 66(1), 67(5), 69(1). The males have on average approx. three more antennal segments than females. Males are very similar but often darker than females, 2^nd^ tergite 0.9–1.0 × as long as basal width of tergite and apical tergites type 1 and (usually) type 2, with fringe present in the latter (Fig. 215); hind femur at most apically blackish, and hind tibial spurs sometimes blunt.

##### Distribution.

*Austria, Bulgaria, Croatia, Czech Republic, Finland, France, Germany, *Greece, Italy, *Moldova, Mongolia, *Netherlands, Norway, *Romania, Slovakia, *Slovenia, Spain, Sweden, Ukraine.

##### Notes.

An examined female (NMS) from Albania (Mt Mali me Gropa, above Shengiergi, 1400 m, 13.viii.2019, MV light, C.W. Plant) has a CO1 sequence (MRS940) 3 % different from *A. cruentus* (19 differences in 626 bp of overlap) and although superficially similar in colour is clearly distinct in having OOL shorter (0.5 × lateral ocellus), a smaller hypoclypeal depression (0.5 × width of face), slenderer hind femur (5 × as long as wide), and several other differences. It may be *A. parvicauda* (Tobias, 1985) described from Afghanistan, but it has more (64; 58–60 in type series) and somewhat more elongate antennal segments than described for *A. parvicauda*, as well as other small deviations. Additional material as well as comparison with the type series of *A. parvicauda* are needed to settle the status of the Albanian species.

**Figures 196–198.**
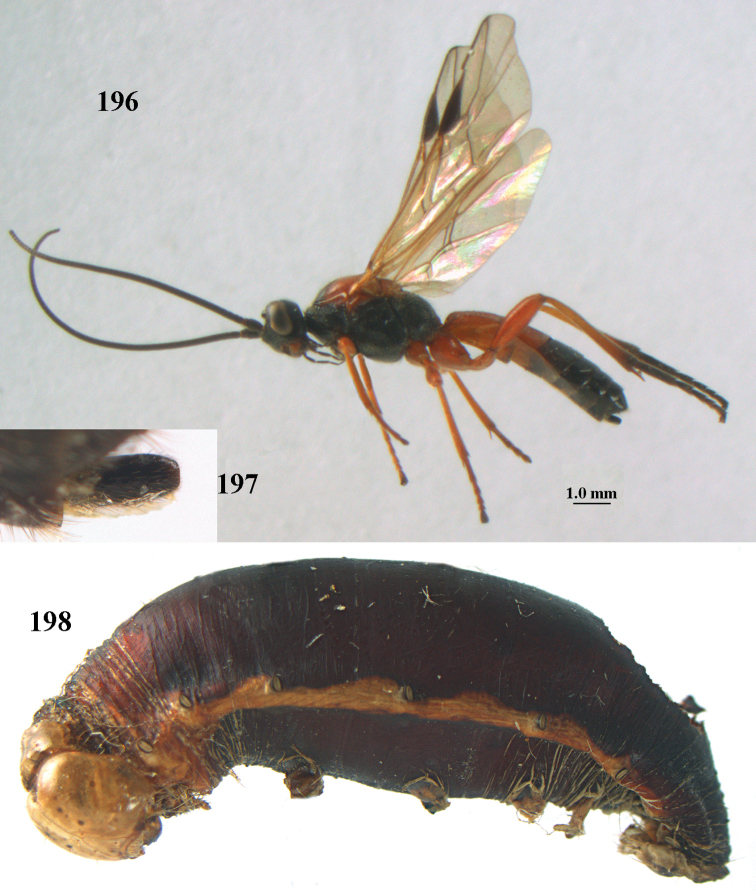
*Aleiodes cruentus* (Nees), ♀, Germany, Markkleeberg, but 198 from Finland, Mäntyharju **196** habitus lateral **197** ovipositor sheath lateral **198** mummy of *Hadena confusa* (Hufnagel).

**Figures 199–211.**
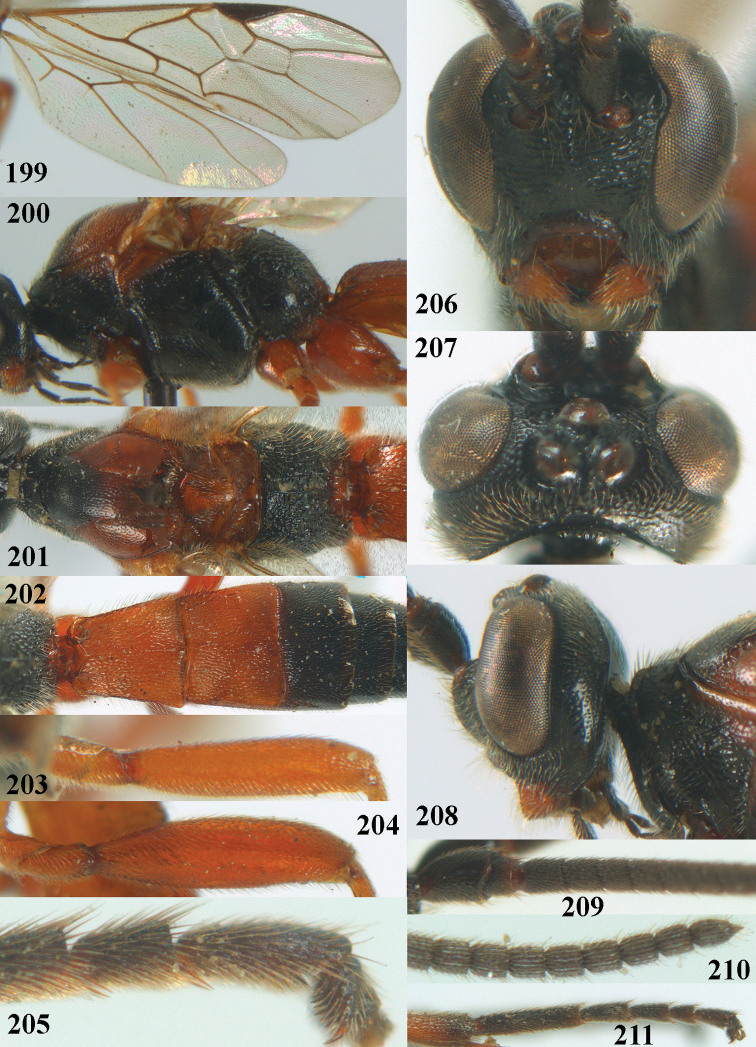
*Aleiodes cruentus* (Nees), ♀, Germany, Markkleeberg **199** wings **200** mesosoma lateral **201** mesosoma dorsal **202** metasoma dorsal **203** fore femur lateral **204** hind femur lateral **205** outer hind tarsal claw **206** head anterior **207** head dorsal **208** head lateral **209** base of antenna **210** apex of antenna **211** hind tarsus lateral.

**Figures 212–216.**
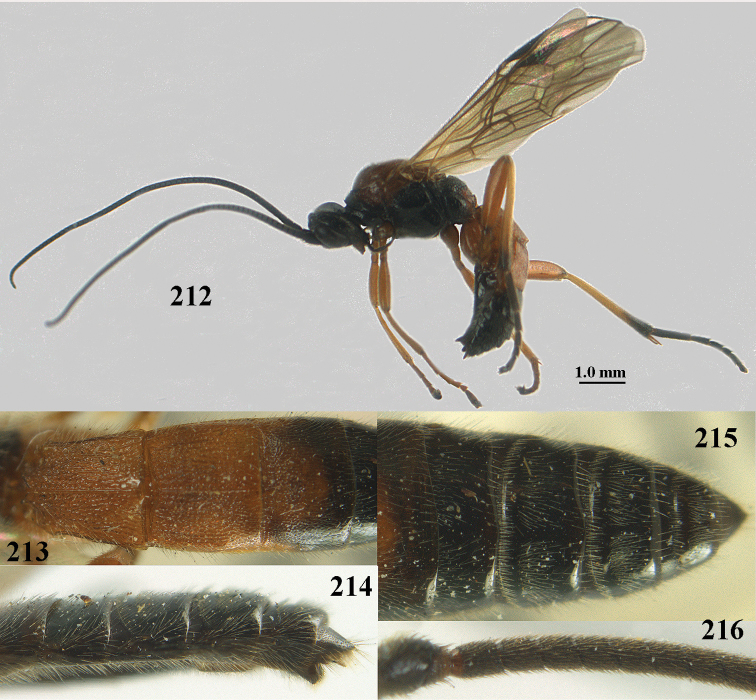
*Aleiodes cruentus* (Nees), ♂, Hungary, Hársbokorhegy, but 213–215 from Germany, Markkleeberg **212** habitus lateral **213** 1^st^–3^rd^ metasomal tergites dorsal **214** 4^th^–7^th^ metasomal tergites lateral **215** id. dorsal **216** basal antennal segments.

#### Aleiodes desertus

Taxon classificationAnimaliaHymenopteraBraconidae

(Telenga, 1941)

737A772F-5088-5899-8105-03925C5ACB00

Figs 217–220 , 221–233

Rhogas (Rhogas) desertus Telenga, 1941: 184–185, 423 (not R. aestuosus var. desertus Telenga, 1941, from China) [examined].
Rogas desertus ; Shenefelt 1975: 1223.
Rogas (Rogas) desertus ; Tobias 1986: 76 (transl. 124) (lectotype designation).
Aleiodes desertus ; Fortier and Shaw 1999: 230.

##### Type material.

Lectotype, ♀ (ZISP), “[**Uzbekistan**:] Khiva, 30.iv.[1]927, V. Gussakovskij/ S.Kh.Op.Ot., at light”, “Lectotypus *Rogas desertus* Tel., design. [V.I.] Tobias, 1980”. Paralectotypes: 1 ♀ (ZISP), “[**Turkmenistan**:] Ashkhabad [= Ashgabat], 25.iii.[1]905, S. Ahnger”, “Paralectotypus *Rogas desertus* Tel., design. [V.I.] Tobias, 1980”; 1 ♀ (ZJUH, figured), “Khiva, Rabat, 3.v.[1]927, V. Gussakovskij/collected at light”, “Paratypus *Rogas desertus* Telenga”, “Rec[eived] in exchange Academy of Science, Leningrad, B.M.1963-211”.

##### Molecular data.

None.

##### Biology.

Unknown. It seems to fly in spring (March–May) and may be univoltine.

##### Diagnosis.

Maximum width of hypoclypeal depression 0.9–1.0 × minimum width of face; anterior part of clypeus very narrow, most of clypeus depressed (Fig. 229); OOL approx. 0.9 × diameter of posterior ocellus and remotely punctate; mandible massive triangular, coarsely punctate and with thick ventral lamella (Figs 229, 231); face largely transversely rugose; malar space 0.15 × as long as height of eye and 0.27 × basal width of mandible; area of precoxal sulcus (but posteriorly superficially) and anteriorly area above it distinctly rugose; lateral lobes of mesoscutum largely smooth, strongly shiny and glabrous, middle lobe remotely punctulate and with satin sheen; basal half of wings (except anteriorly) largely glabrous and remainder of wing inconspicuously setose; vein r of fore wing 0.7–0.8 × vein 3-SR (Fig. 221) vein 1-CU1 0.1 × as long as 2-CU1, narrow and oblique; tarsal claws long, slender, hardly bent and simple (Fig. 232); tarsal segments (except telotarsus) with four apical spines; 1^st^ and base of 2^nd^ tergite aciculate-rugulose, 3^rd^ tergite micro-sculptured and matt, remainder of metasoma shiny and rather smooth; head and mesosoma (except prothorax anteriorly and mesoscutum posteriorly) black; pterostigma dark brown; legs and palpi pale yellowish. According to original description antenna of ♀ with 50–52 segments, but ZJUH paralectotype has 63 segments.

##### Description.

Lectotype, ♀, length of fore wing 7.5 mm, of body 8.2 mm.

***Head.*** Antennal segments of ♀ more than 45, but apical segments missing, length of antenna of paralectotype 1.1 × body and its subapical segments moderately slender; frons rugose, shiny; OOL 0.9 × diameter of posterior ocellus; OOL and vertex remotely punctate, shiny; anterior part of clypeus 9 × wider than high, coarsely punctate and rather convex; clypeus above lower level of eyes; ventral margin of clypeus thick and not protruding forwards; width of hypoclypeal depression 0.9 × minimum width of face (Fig. 229); length of eye 1.7 × temple in dorsal view (Fig. 230); vertex behind stemmaticum convex and sparsely punctate; length of malar space 0.15 × length of eye in lateral view; mandible massive triangular, coarsely punctate and with thick ventral lamella (Figs 229, 231); occipital carina nearly complete, fine and ventrally strongly curved.

***Mesosoma.*** Lateral lobes of mesoscutum largely smooth, strongly shiny and glabrous, middle lobe remotely punctulate and with satin sheen; prepectal carina complete and lamelliform; precoxal area of mesopleuron widely rugose, but posterior 0.2 narrowly striate; mesopleuron above precoxal area anteriorly rugose and remainder weakly and sparsely punctate, shiny; axilla crenulate but posteriorly densely and coarsely rugose; scutellum largely smooth, with some punctures; propodeum evenly convex, finely rugose and with strong medio-longitudinal carina, without tubercles.

***Wings.*** Fore wing: basal half largely glabrous; r 0.7 × 3-SR (Fig. 219); 1-CU1 oblique, 0.1 × as long as 2-CU1; r-m nearly as long as 3-SR; 2^nd^ submarginal cell comparatively short (Fig. 221); cu-a inclivous; 1-M nearly straight posteriorly. Hind wing: basal 0.4 of marginal cell slightly widened and distally strongly widened, its apical width 2.7 × width at level of hamuli (Fig. 222); 2-SC+R subquadrate; m-cu indistinct; M+CU:1-M = 3:2; 1r-m 0.8 × 1-M.

***Legs.*** Tarsal claws slender, slightly curved and only setose (Fig. 232); hind coxa partly obliquely striate dorsally; tarsi slender, segments (except telotarsus) with long apical spines; length of hind femur and basitarsus 5.0 and 6.8 × their width, respectively; length of inner hind spur 0.3 × hind basitarsus.

***Metasoma.*** First tergite robust, 0.9 × longer than wide apically, strongly narrowed anteriorly (Fig. 225) and rather flat posteriorly; 1^st^ and 2^nd^ tergites finely longitudinally striate-rugulose; medio-longitudinal carina of 1^st^ and 2^nd^ tergites indistinct; 2^nd^ tergite 0.6 × longer than its basal width; medio-basal area of 2^nd^ tergite wide triangular, rather short; 2^nd^ suture shallow and narrow; 3^rd^ tergite matt and micro-sculptured, anteriorly finely striate; 4^th^ and apical half of 3^rd^ tergite without sharp lateral crease; ovipositor sheath with rather short setae and apically truncate (Fig. 220).

***Colour.*** Black; mesoscutum posteriorly partly chestnut brown; antenna, clypeus, malar space ventrally, mandible, pronotum and propleuron anteriorly and metasoma, brownish yellow; tegulae, legs and palpi pale yellowish; pterostigma and ovipositor sheath dark brown; veins of fore wing (but pale in basal 0.3 of fore wing) brown; wing membrane hyaline.

***Variation.*** Length of body 7.0–8.2 mm, of fore wing 7.5–7.9 mm; temple punctate to smooth; precoxal sulcus area finely to rather coarsely rugose; pronotal side largely black (except ventrally) black or brownish yellow; lateral lobes of mesoscutum entirely dark chestnut brown or only posteriorly so, or mesoscutum largely yellowish brown posteriorly and prolonged to base of notauli; first tergite usually entirely brownish yellow, but sometimes dark brown and only posteriorly and laterally yellowish; pterostigma dark brown or brown. Antennal segments: ♀ 63(1).

##### Distribution.

Turkmenistan, Uzbekistan.

##### Notes.

We have included this extralimital species from Central Asia because we suspect it may occur in Turkey. It should not be confused with Rogas aestuosus var. desertus Telenga, 1941, described from China in the same paper. The latter is an unavailable name (a primary homonym) and most likely a colour variety of *R. aestuosus*.

**Figures 217–220.**
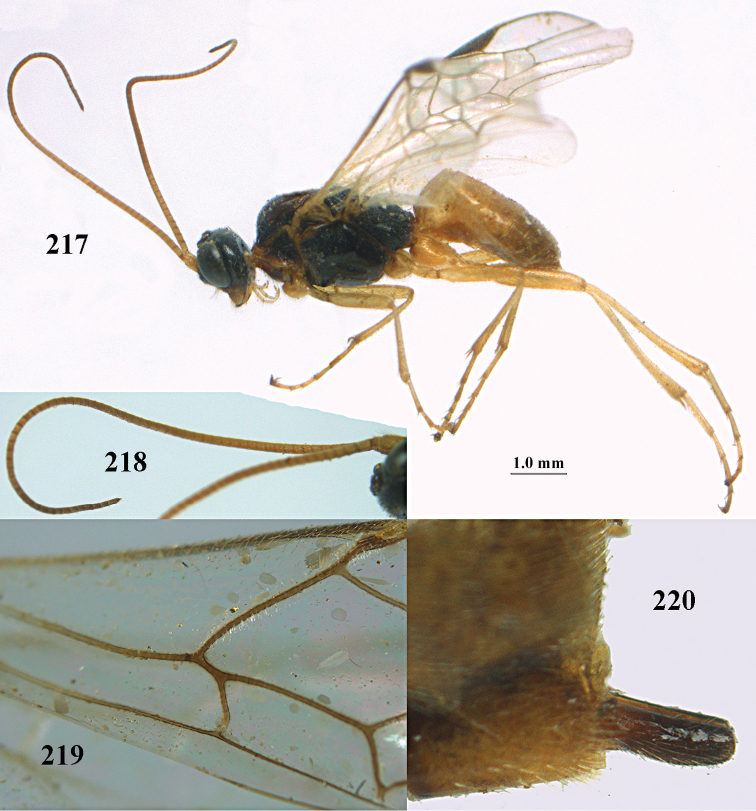
*Aleiodes desertus* (Telenga), ♀, paralectotype **217** habitus lateral **218** antenna lateral **219** detail of fore wing **220** ovipositor sheath lateral.

**Figures 221–233.**
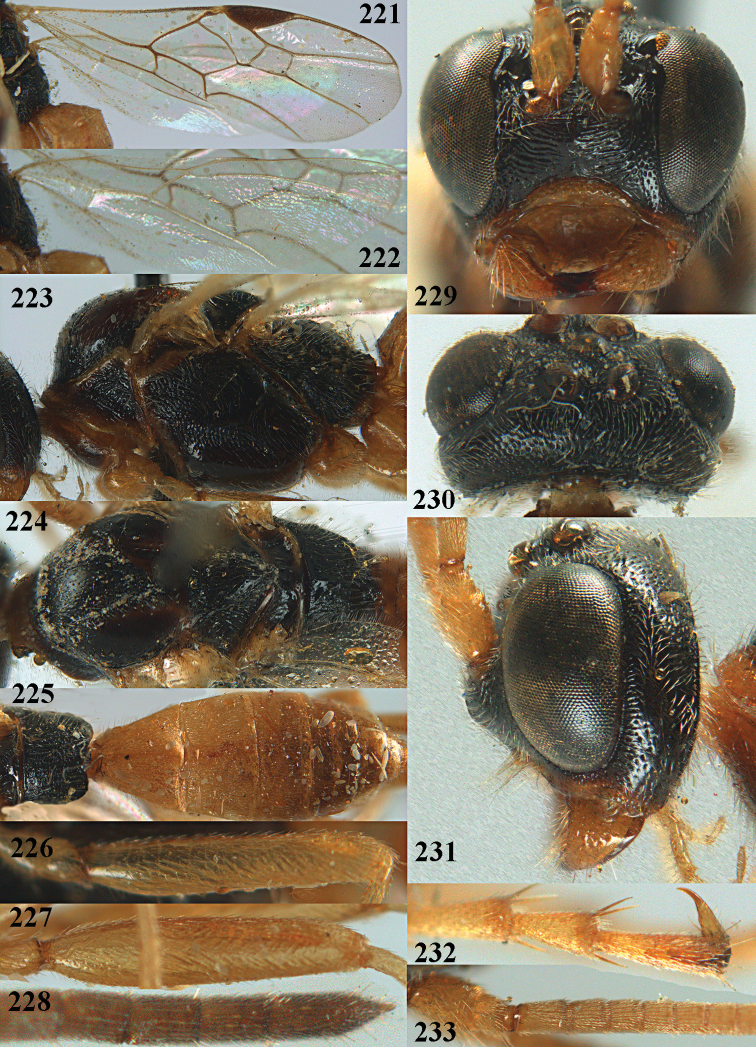
*Aleiodes desertus* (Telenga), ♀, paralectotype, but 224 and 230 of lectotype **221** fore wing **222** hind wing **223** mesosoma lateral **224** mesosoma dorsal **225** metasoma dorsal **226** fore femur lateral **227** hind femur lateral **228** apex of antenna **229** head anterior **230** head dorsal **231** head lateral **232** outer hind tarsal claw **233** base of antenna.

#### Aleiodes dissector

Taxon classificationAnimaliaHymenopteraBraconidae

(Nees, 1834)

446B0A23-445D-52A8-905F-648AEDEF5326

Figs 234–239 , 240–242 , 243–255 , 256–263

Rogas dissector Nees, 1834: 208; Shenefelt 1975: 1225–1226; Papp 1977a: 110.
Rogas (Rogas) dissector ; Tobias 1976: 81, 1986: 75 (transl.: 121).
Aleiodes (Neorhogas) dissector ; Papp, 1985a: 145, 1987b: 35, 1991a: 74, 1991d: 5, 1999: 550; Belokobylskij, 1996: 9; Riedel et al., 2002: 106.
Aleiodes (Chelonorhogas) dissector ; Belokobylskij 2000: 34; Ku et al. 2001: 234, 235.
Aleiodes dissector ; Fortier and Shaw 1999: 230; Belokobylskij et al. 2003: 398; Zaldívar-Riverón et al. 2004: 234, 2008: 392; Papp 2005: 176; Belokobylskij et al. 2008: 136–137.
Phylax aestivalis Snellen van Vollenhoven, 1858: 282; Shenefelt, 1975: 1226 (as synonym of A. dissector); van Achterberg 1992: 363 (id.); Papp 2005: 176 (id.) [examined].

##### Type material.

Holotype of *A. aestivalis*, ♀ (RMNH), “[**Netherlands**], Haag [= near The Hague], 6 [= June], v.Voll.”. According to the original description the ♂ holotype of *R. dissector* from Germany should be in the Gravenhorst collection (Museum of Natural History, University of Wrocław, Wrocław), but so far it has not been found.

##### Additional material.

Austria, British Isles (England: V.C.s 15, 17, 20, 22, 23, 24, 30, 31, 34, 37, 58; Scotland: V.C.s 73, 88, 89, 95, 96, 97, 107), Croatia, Czech Republic, Finland, France, Germany, Greece, Hungary, Montenegro, Italy, Netherlands (FL: Lelystad, GE: Barneveld, OV: Raalte (Heino), ZH: Wassenaar), Norway, Russia, Serbia, Slovakia, Switzerland, Ukraine, [Armenia]. Specimens in ZJUH, BZL, CNC, IKC, MRC, MSC, MSNV, MTMA, NMS, OUM, RMNH, SDEI, UNS, UWIM, ZSSM.

##### Molecular data.

MRS007 (Turkey), MRS025 (Turkey), MRS145 (UK), MRS146 (UK).

##### Biology.

**Figures 234–239.**
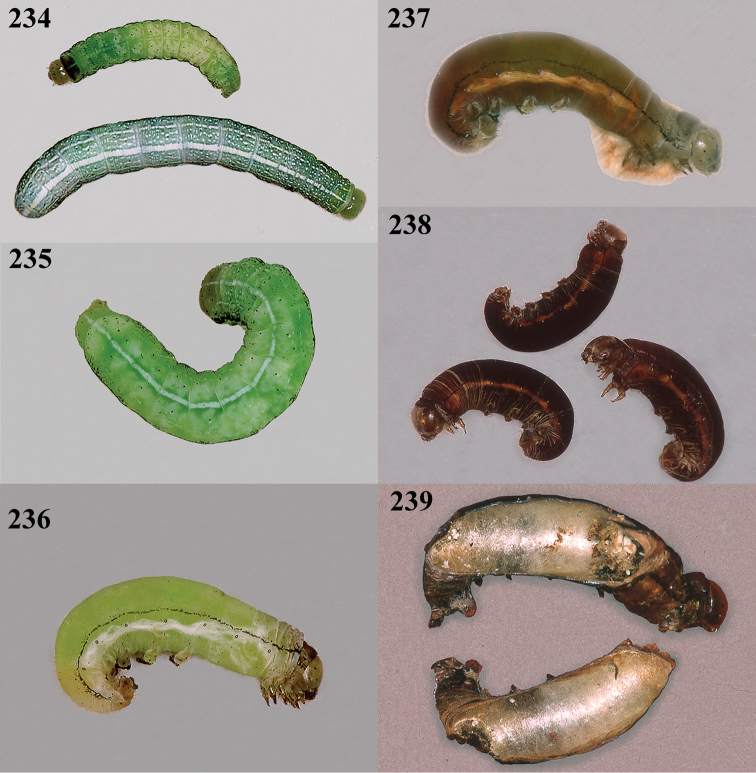
*Aleiodes dissector* (Nees), U.K., Scotland (in culture) parasitising *Orthosia incerta* (Hufnagel) **234** pre-mummy, removed from its hideaway, with unparasitised control from the same egg batch (below) **235** pre-mummy **236** early mummification **237** mummy with ventral ooze **238** three fully hard mummies **239** emerged mummy, cut open to expose silken lining.

Univoltine, collected in May and June in deciduous scrub and woodland. In Britain it is widespread but particularly common in birch-dominated woodland in upland Scotland. Reared from the noctuids *Orthosia incerta* (Hufnagel) (17, M.R. Shaw), *O. gothica* (Linnaeus) (1, J.L. Yela) and *Orthosia* sp. (3), overwintering in the concealed mummy. An additional specimen, lacking a mummy but labelled as reared doubtfully from the sesiid *Paranthrene tabaniformis* (Rottemburg) (RMNH), which normally feeds under *Populus* bark at ground level or below, can be discounted as a probable substrate rearing in which the mummy of the true host was overlooked. Parasitised host larvae in their penultimate instar leave their feeding sites and enter the soil or other site of moderate concealment (including below loose bark), where they prepare a chamber as though to pupate. At this time the parasitoid larva within the strongly retarded host (Fig. 234) is around half its final length, and the host lies quiescent for approx. a week until the parasitoid has completed its feeding (Fig. 235). During mummification (Figs 236, 237) the caudal end of the host recurves ventrally as the host’s body becomes weakly retracted. A ventral opening at the head end is made, but the head (as with the caudal segments) is usually tucked downwards rather than becoming raised, and so the resulting expelled fluid (Fig. 237) usually dries without the mummy becoming stuck down. The eventual outcome is a rather distinctive (Fig. 238) elongate and curved dark brown structure with a paler and weakly raised lateral keel. The parasitoid’s pupation chamber occupies ca 2^nd^–8^th^ abdominal segments of the host, which are moderately strongly lined with silk (Fig. 239).

The moderately large hypoclypeal opening and protruding sharp-rimmed clypeus of *A. dissector* is seen in some other species (e.g., *A. modestus* (Reinhard), treated in part 1 of this work) whose hosts also pupate in shallow soil. In culture experiments *A. dissector* was found to prefer hosts in the early to middle part of the 3^rd^ instar, although late 2^nd^ instar host were often also acceptable. Oviposition into suitable hosts was rapid (1–2 seconds) and accomplished with a single insertion of the ovipositor, following only brief antennation and no use of the legs. There was no clear temporary paralysis. Experimental rearings from *O. incerta* (6:107\85\\75+10) and *O. gothica* (6:61\49\\34+15) were comparable (given that some insertions of less than a full second might have been scored as ovipositions incorrectly; and furthermore that some failures to oviposit into these hosts might be ascribed to temporary egg depletion, as the protocol of normally ceasing to offer hosts to a particular female after four apparent ovipositions on the day had not been developed until after the experiments were undertaken), and clearly demonstrated the suitability of both species as hosts. In contrast, no parasitoids developed (and indeed possibly no ovipositions occurred) in the other species of *Orthosia* tested, which were all found to be clearly outside the host range: *O. cerasi* (Fabricius) (3:32\?3\\0+3); *O. cruda* (Denis & Schiffermüller) (2:12\0\\-); *O. munda* (Denis & Schiffermüller) (3:10\0\\-); *O. gracilis* (Denis & Schiffermüller) (2:11\?1\\0+1). Of these four, only *O. gracilis* is not fully arboreal. There is no adverse venom effect on host development.

##### Diagnosis.

Maximum width of hypoclypeal depression 0.6–0.7 × minimum width of face (Fig. 251); OOL of ♀ 0.6–0.7 × diameter of posterior ocellus (Fig. 252) and sparsely punctate; ventral margin of anterior part of clypeus comparatively sharp and more or less protruding outwards (Fig. 253); length of malar space 0.2 × length of eye in lateral view (Fig. 253); head transverse in dorsal view and eye 1.5–2.0 × as long as temple in dorsal view (Fig. 252); lobes of mesoscutum punctulate, with interspaces smooth to superficially micro-sculptured; precoxal area completely smooth or nearly so; vein 1-CU1 of fore wing 0.2–0.3 × vein 2-CU1 and horizontal (Fig. 243); hind tarsal claws with conspicuous dark brown pecten close to apical tooth (Fig. 250); 1^st^ tergite rounded antero-laterally and 1.0–1.1 × as long as wide apically; basal half of metasoma black and weakly sculptured; 3^rd^ tergite smooth; head black; palpi yellowish; basal half of hind tibia pale yellowish, but in some males almost uniformly dark; 4^th^–6^th^ tergites of males depressed medially and conspicuously setose (Fig. 258).

##### Description.

Redescribed ♀ (RMNH) from Austria (Burgenland, Winden am See). Length of fore wing 8.5 mm, of body 9.0 mm.

***Head.*** Antennal segments of ♀ 60, antenna as long as fore wing, its subapical segments rather slender, slightly longer than wide; frons largely smooth; OOL 0.7× diameter of posterior ocellus, sparsely punctate, shiny and with deep groove near posterior ocellus (Fig. 252); vertex sparsely punctate, rather shiny; clypeus coarsely punctate; ventral margin of clypeus rather thin and forward protruding (Fig. 253); width of hypoclypeal depression 0.7 × minimum width of face (Fig. 251); length of eye 1.5 × temple in dorsal view (Fig. 252); vertex behind stemmaticum superficially rugose-punctate; clypeus near lower level of eyes; length of malar space 0.2 × length of eye in lateral view.

***Mesosoma.*** Mesoscutal lobes punctulate with interspaces superficially micro-sculptured and shiny; precoxal area of mesopleuron smooth except some punctulation, mesopleuron punctulate anteriorly and posteriorly; metapleuron densely punctate; metanotum with nearly complete median carina; scutellum flat (but with rugulose depression medio-posteriorly), remainder punctulate and with weak lateral carinae; propodeum evenly convex and coarsely rugose, and medio-longitudinal carina absent posteriorly.

***Wings.*** Fore wing: r 0.4 × 3-SR; 1-CU1 horizontal, 0.3 × 2-CU1; r-m 0.3 × 3-SR; 2^nd^ submarginal cell medium-sized (Fig. 243); cu-a inclivous, straight; 1-M straight posteriorly; 1-SR medium-sized; surroundings of M+CU1, 1-M and 1-CU1 largely setose. Hind wing: marginal cell rather narrow basally, apical half gradually widened, its apical width 3.1 × width at level of hamuli (Fig. 244); 2-SC+R subquadrate; m-cu absent; M+CU:1-M = 35:33; 1r-m 0.7 × 1-M.

***Legs.*** Tarsal claws with conspicuous and robust dark brown pecten (Fig. 250); hind coxa distinctly punctate and with some oblique striae postero-dorsally; hind trochantellus robust; length of hind femur and basitarsus 3.8 and 5.3 × their width, respectively; length of inner hind spur 0.45 × hind basitarsus.

***Metasoma.*** First tergite flattened, basally narrowed, as long as wide apically; 1^st^ and 2^nd^ tergites with medio-longitudinal carina and largely finely punctate-rugose, but posterior quarter of 2^nd^ tergite irregularly rugose and no median carina; medio-basal area of 2^nd^ tergite wide and triangular, distinct (Fig. 247); 2^nd^ suture rather deep and micro-sculptured; 3^rd^ and subsequent tergites largely smooth; apical half of 3^rd^ and 4^th^ tergites without sharp lateral crease; ovipositor sheath wide, with long and medium-sized setae and apically truncate (Fig. 241).

***Colour.*** Black; apical half of hind tibia and hind tarsus blackish; basal half of hind tibia pale yellowish; remainder of legs, palpi and tegulae yellowish brown; most veins and pterostigma dark brown; wing membrane slightly yellowish basally and remainder slightly infuscate.

***Variation.*** Interspaces between punctulation of mesoscutum smooth to superficially micro-sculptured; medio-longitudinal carina of propodeum complete or absent posteriorly; 3^rd^ metasomal tergite largely finely sculptured (except posteriorly) to largely smooth; mesopleuron black or with brownish longitudinal stripe; hind tibia usually ivory or pale yellowish basally. Antennal segments: ♀ 51(2), 55(2), 56(7), 57(4), 58(7), 59(12), 60(14), 61(18), 62(6), 63(4); ♂ 51(1), 53(2), 54(2), 55(4), 56(8), 57(29), 58(29), 59(27), 60(15), 61(4), 62(4), 63(2), 64(2). Females have on average ca one to two more antennal segments than males. Males are very similar but hind femur more or less blackish and, in some males, hind tibia almost uniformly dark, OOL approx. as long as diameter of posterior ocellus (Fig. 257) and apical tergites type 3–4 with fringe long and strong (Figs 258, 260).

##### Distribution.

*Armenia, *Austria, British Isles (England, Scotland), Croatia, Czech Republic, Finland, France, Germany, *Greece, Hungary, *Montenegro, *Italy, Netherlands, Norway, Russia, *Serbia, Switzerland, Ukraine.

**Figures 240–242.**
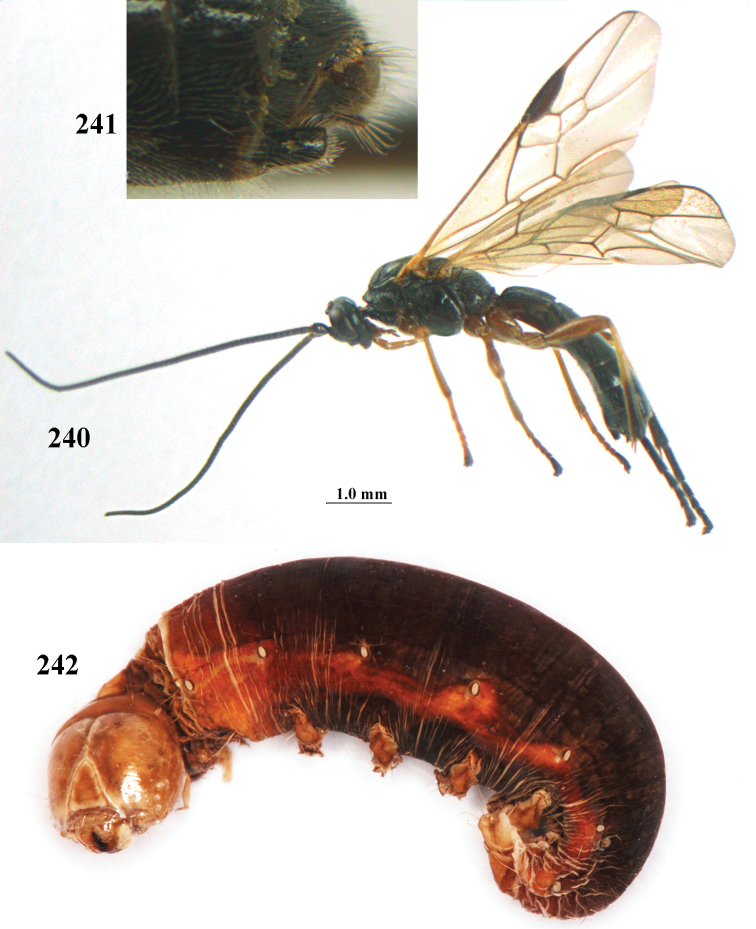
*Aleiodes dissector* (Nees), ♀, Switzerland, Tessin, but 242 from Scotland (culture) **240** habitus lateral **241** ovipositor sheath lateral **242** mummy of *Orthosia incerta* (Hufnagel).

**Figures 243–255.**
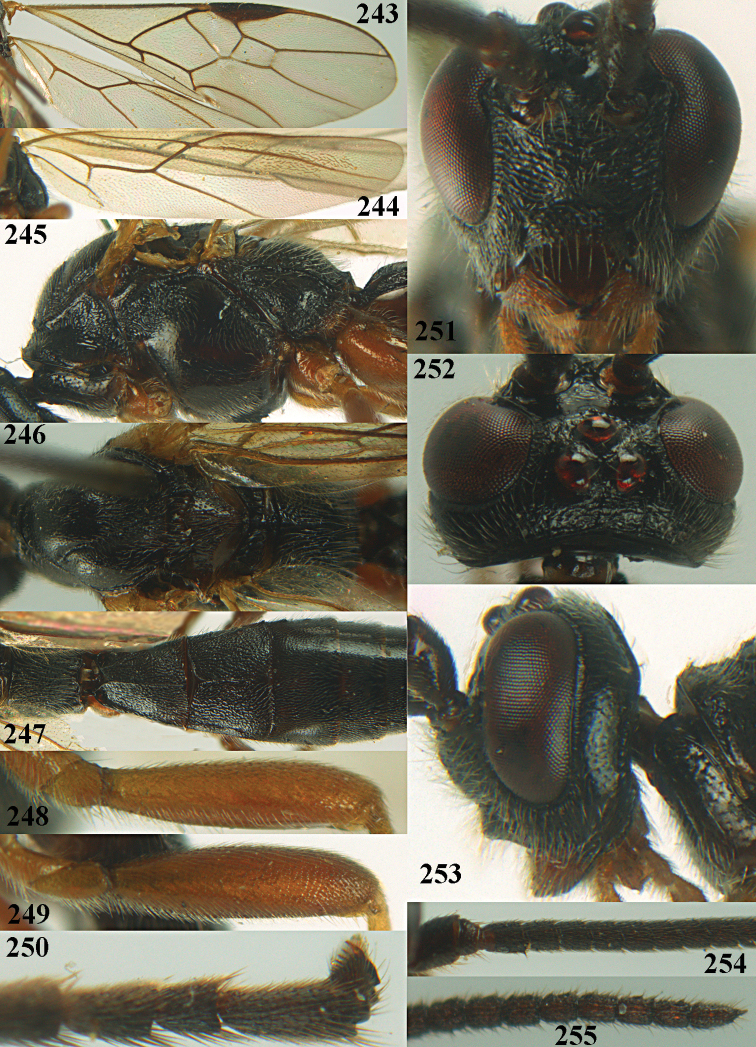
*Aleiodes dissector* (Nees), ♀, Switzerland, Tessin **243** fore wing **244** hind wing **245** mesosoma lateral **246** mesosoma dorsal **247** propodeum and 1^st^ –4^th^ metasomal tergites dorsal **248** fore femur lateral **249** hind femur lateral **250** outer hind tarsal claw **251** head anterior **252** head dorsal **253** head lateral **254** base of antenna **255** apex of antenna.

**Figures 256–263.**
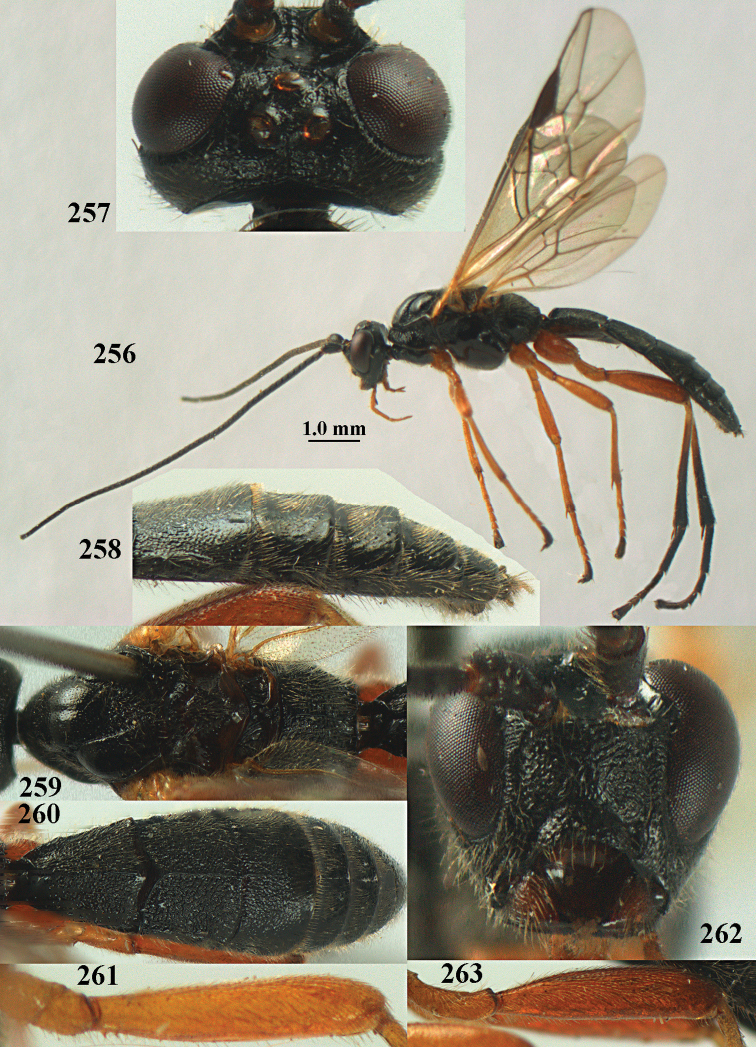
*Aleiodes dissector* (Nees), ♂, Austria, Kärnten **256** habitus lateral **257** head dorsal **258** 3^rd^–7^th^ tergites lateral **259** mesosoma dorsal **260** 1^st^–6^th^ metasomal tergites dorsal **261** fore femur lateral **262** head anterior **263** hind femur lateral.

#### Aleiodes diversus

Taxon classificationAnimaliaHymenopteraBraconidae

(Szépligeti, 1903)

CF7B3762-3684-53C6-8D8C-C0F3B0486369

Figs 264–265 , 266–277 , 278–288

Rhogas diversus Szépligeti, 1903: 114; Papp 2004: 216 (as synonym of A. dissector) [examined].
Rogas dissector var. diversus ; Shenefelt 1975: 1226 (lectotype designation).
Aleiodes (Neorhogas) diversus ; Papp 1977a: 110–112 (re-instated), 1985: 145, 1991a: 81.
Aleiodes diversus ; Belokobylskij et al., 2003: 398 (as synonym of A. dissector); Papp 2005: 176; Merz and Pasche 2012: 244; van Achterberg 2014: 209.

##### Type material.

Lectotype, ♀ (MTMA), “**Croatia**, Buccari [= Bakar], 1893, Pavel”, “Lectotypus, ♀”, Rogas (s. str.) diversus Szépligeti, 1906 [sic!], Papp, 1968”, “Hym. Typ. No. 1011, Mus. Budapest”.

##### Additional material.

Austria, British Isles (England: V.C.s 8, 25, 70), Bulgaria, Croatia, Hungary, Italy (Sicily), Norway, Switzerland. Specimens in ZJUH, BZL, CMIM, MHNG, MRC, MTMA, NMS, RMNH, SDEI, ZSSM. The most recent of the five English specimens seen is dated 1931, and it seems likely that this rather large and showy insect is extinct in Britain.

##### Molecular data.

None.

##### Biology.

Unknown. Female specimens have been collected in (May–)June, and also September, suggesting that it may be plurivoltine. This is reinforced by the date of capture of the two available males (which would not have hibernated as an adult) in Sicily on 30.iv.1965 (ZJUH) and 1.v.1994 (RMNH). There is no indication of habitat on data labels and we have not seen reared material.

##### Diagnosis.

Maximum width of hypoclypeal depression 0.5–0.6 × minimum width of face (Fig. 273); OOL of ♀ coarsely punctate and 1.0–1.2 × diameter of posterior ocellus; ventral margin of clypeus (rather) obtuse apically and clypeus not protruding outwards (Fig. 275), but sometimes intermediate; length of eye 1.0–1.2 × temple in dorsal view; lobes of mesoscutum densely finely punctate, with interspaces approx. equal to diameter of punctures, shiny and smooth; precoxal area with some rugae medially; vein cu-a of fore wing vertical; surroundings of veins M+CU1 and 1-+2-CU1 largely glabrous; vein 1-CU1 of fore wing 0.7–1.1 × vein 2-CU1 and approx. as long as vein m-cu (Fig. 266), rarely shorter; hind femur 3.0–3.3 × longer than wide; hind tarsal claws with medium-sized dark brownish pecten up to apical tooth (Fig. 272); 1^st^ tergite widened apically and moderately wide basally (Fig. 269); 2^nd^ tergite 0.7–0.8 × as long as wide (Fig. 269) and black; 4^th^–7^th^ tergites of males flat and with long yellowish setae (Figs 279, 282); head black; vein 1-M of fore wing brownish; wing membrane subhyaline.

##### Description.

Lectotype, ♀, length of fore wing 7.0 mm, of body 10.0 mm.

***Head.*** Antennal segments of ♀ 56, antenna as long as fore wing, its subapical segments robust; frons largely smooth behind antennal sockets; OOL 1.2 × diameter of posterior ocellus, and coarsely punctate, interspaces less than diameter of puncture; vertex coarsely punctate; clypeus rugose; ventral margin of clypeus thick and not protruding forwards (Fig. 275); width of hypoclypeal depression 0.6 × minimum width of face (Fig. 273); length of eye 1.2 × temple in dorsal view (Fig. 274); vertex behind stemmaticum superficially punctate-rugose; clypeus near lower level of eyes; length of malar space 0.3 × length of eye in lateral view.

***Mesosoma.*** Mesoscutal lobes densely and finely punctate, interspaces largely smooth, shiny; precoxal area of mesopleuron coarsely punctate and without rugae medially, mesopleuron coarsely punctate anteriorly and posteriorly; metapleuron moderately punctate; scutellum remotely punctate; propodeum rather convex and coarsely rugose.

***Wings.*** Fore wing: r 0.5 × 3-SR (Fig. 266); 1-CU1 horizontal, 0.7 × 2-CU1; r-m 0.5 × 3-SR; 2^nd^ submarginal cell rather long (Fig. 266); cu-a vertical, straight; 1-M rather curved posteriorly; surroundings of M+CU1, 1-M and 1-CU1 largely glabrous. Hind wing: marginal cell gradually widened, its apical width 2.3 × width at level of hamuli (Fig. 266); 2-SC+R transverse; m-cu largely absent, only as short antefurcal remnant (Fig. 266); M+CU:1-M = 35:23; 1r-m 0.7 × 1-M.

***Legs.*** Tarsal claws with rather conspicuous, medium-sized dark brown pecten up to apical tooth (Fig. 272); hind coxa largely punctate; hind trochantellus robust; length of hind femur and basitarsus 3.1 and 4.4 × their width, respectively; length of inner hind spur 0.5 × hind basitarsus.

***Metasoma.*** First tergite rather flattened, as long as wide apically; 1^st^ and 2^nd^ tergites with medio-longitudinal carina and coarsely vermiculate-rugose; medio-basal area of 2^nd^ tergite triangular and distinct (Fig. 269); 2^nd^ suture deep; 2^nd^ tergite 0.7–0.8 × as long as wide (Fig. 269); 3^rd^ tergite densely punctate and interspaces largely smooth, remainder of metasoma largely smooth; 4^th^ and apical half of 3^rd^ tergite without sharp lateral crease; apical third of metasoma rather compressed; ovipositor sheath wide, with rather short setae and apically truncate (Fig. 265).

***Colour.*** Black; mesoscutum (except anterior third), scutellum, clypeus ventrally, mandible, tegulae and legs largely brownish red; palpi, fore coxa largely, telotarsi, hind tarsus and apex of hind tibia (excluding spurs) dark brown; pterostigma blackish brown; veins dark brown, but near wing base yellowish; wing membrane slightly infuscate.

***Variation.*** OOL 1.0–1.2 × diameter of posterior ocellus; mesoscutum of ♀ entirely brownish red or yellowish brown, or anteriorly black; 1^st^ tergite 1.0–1.1 × longer than wide apically; metasoma rarely partly obscurely reddish dark brown; mesopleuron may be just punctate or may have some rugae in lower half. Antennal segments ♀: 55(3), 56(3), 57(3), 58(1), 59(1); ♂ 58(1). Males have mesosoma black (Fig. 278), 2^nd^ tergite 0.8–0.9 × as long as basal width of tergite (Fig. 282) and apical tergites type 2, setae rather long, fringe long and strong (Fig. 279).

##### Distribution.

*Austria, *British Isles (England; probably extinct), *Bulgaria, Croatia, Hungary, *Italy (Sicily), *Norway, *Switzerland.

##### Notes.

Close to *A. cruentus* which, however, almost always has much or all of 1^st^ and 2^nd^ metasomal tergites orange-red (usually wholly black or dark brown in *A. diversus*). In addition to characters given in the key *A. diversus* is a more robust insect, and females have broader antennal segments (distinctly transverse near middle of flagellum) and on average they are fewer in number (although with overlap).

**Figures 264, 265.**
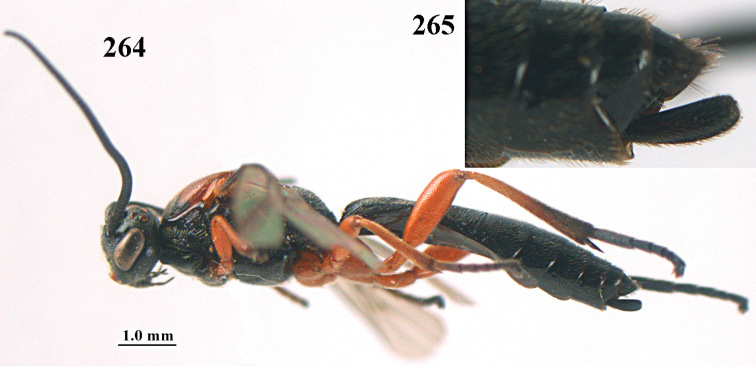
*Aleiodes diversus* (Szépligeti), ♀, Italy, Sicily **264** habitus lateral **265** ovipositor sheath lateral.

**Figures 266–277.**
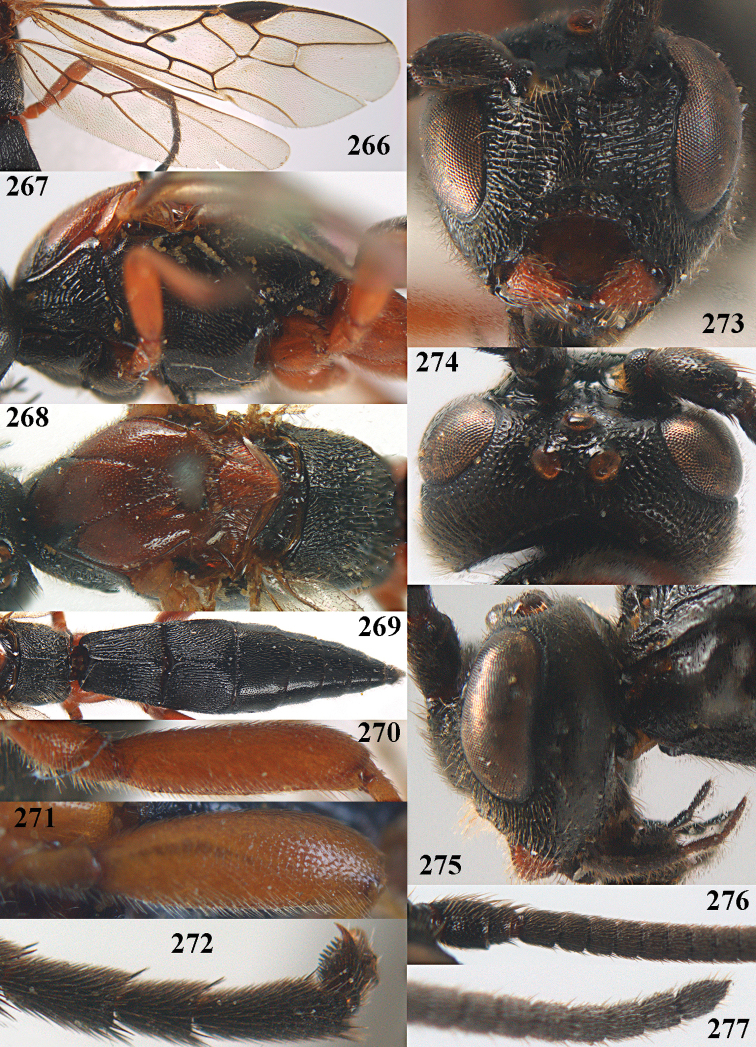
*Aleiodes diversus* (Szépligeti), ♀, Italy, Sicily **266** wings **267** mesosoma lateral **268** mesosoma dorsal **269** propodeum and metasoma dorsal **270** fore femur lateral **271** hind femur lateral **272** outer hind tarsal claw **273** head anterior **274** head dorsal **275** head lateral **276** base of antenna **277** apex of antenna.

**Figures 278–288.**
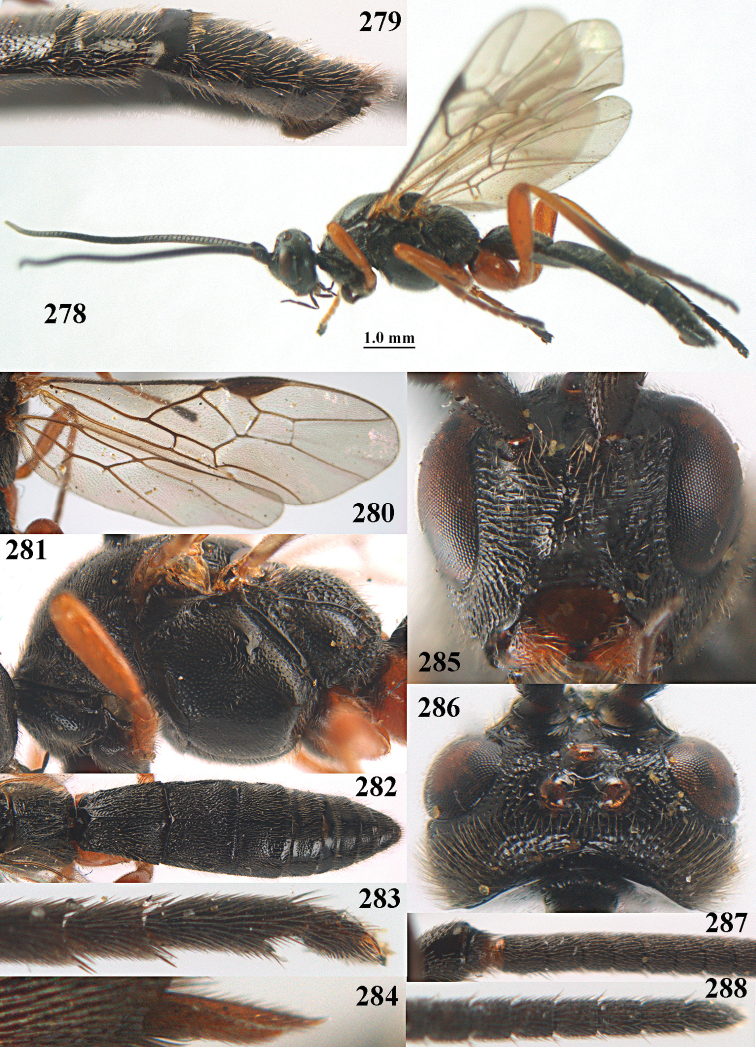
*Aleiodes diversus* (Szépligeti), ♂, Italy, Sicily **278** habitus lateral **279** 3^rd^–7^th^ tergites lateral **280** wings **281** mesosoma lateral **282** metasoma dorsal **283** outer hind claw **284** hind tibial spurs lateral **285** head anterior **286** head dorsal **287** base of antenna **288** apex of antenna.

#### Aleiodes eurinus

Taxon classificationAnimaliaHymenopteraBraconidae

(Telenga, 1941)

596C7289-B8C1-5E50-AA27-7E3F46A5D296

Figs 289–291 , 292–302 , 303–306

Rhogas (Rhogas) eurinus Telenga, 1941: 422.
Rogas eurinus ; Shenefelt 1975: 1228; Papp 1971: 359.
Rogas (Rogas) eurinus ; Tobias 1976: 85, 1986: 80 (transl.: 130; lectotype designation).
Aleiodes (Neorhogas) eurinus ; Papp 1985a: 145; 1991a: 94.
Aleiodes (Chelonorhogas) eurinus ; Chen and He, 1997: 39; He et al. 2000: 667; Belokobylskij 2000: 49; Ku et al. 2001: 235; Farahani et al. 2015: 242–243; Beyarslan et al. 2017: 330.
Aleiodes eurinus ; Fortier and Shaw 1999: 223, 230; Belokobylskij et al. 2003: 398; Papp 2005: 176.
Rogas eurinus ab. nigratus Papp, 1967: 223 (invalid name).
Rogas eurinus ab. nigrimaculatus Papp, 1967: 223 (invalid name).
Rogas eurinus ab. nigripes Papp, 1967: 223 (invalid name).

##### Type material.

None seen.

##### Additional material.

Italy, Russia (Siberia and Far East), Spain, Turkey, [China, Mongolia]. Specimens in ZJUH, BZL, MRC, MSNV, MTMA, NMS, RMNH, SDEI, ZISP.

##### Molecular data.

None.

##### Biology.

Specimens have been collected from April to August, and the presence of males in both April and July clearly demonstrates that it is plurivoltine and overwinters in the mummy. We have not seen reared material, but specimen labelling indicates that it occurs among *Ammophila* and *Schoenus* in the Venice Lido and *Triticum* (presumably cultivated wheat) in Turkey, suggesting that its hosts will occur in open grassland habitats.

##### Diagnosis.

Maximum width of hypoclypeal depression 0.5–0.6 × minimum width of face (Fig. 298); OOL of ♀ approx. as long as diameter of posterior ocellus (Fig. 299) and densely rugose; clypeus rather thin apically and rather protruding anteriorly (Fig. 300); eyes prominent (Fig. 299); lobes of mesoscutum distinctly punctate-granulate and rather matt; precoxal area more or less rugose and comparatively wide medially, and posteriorly punctate; vein 1-CU1 of fore wing 0.3 × vein 2-CU1 (Fig. 292); hind tarsal claws slender, brownish setose and without pecten (Fig. 302); basal half of 3^rd^ tergite striate; 3^rd^ antennal segment of ♀ dark brown; basal half of hind tibia pale yellowish or ivory, at least inner side contrasting with reddish or dark brown colour of basal half of hind femur (usually less pronounced in ♂).

##### Description.

Redescribed ♀ (RMNH) from Turkey (Ankara). Length of fore wing 7.0 mm, of body 8.0 mm.

***Head.*** Antennal segments of ♀ 51 remaining, but apical segments missing, length of antenna 1.2 × fore wing; frons with coarse curved rugae and dorsally coarsely rugose; OOL equal to diameter of posterior ocellus, and densely rugose; vertex spaced rugose, rather dull; clypeus medium-sized and coarsely rugose (as face); ventral margin of clypeus rather thin and rather protruding forwards (Fig. 300); width of hypoclypeal depression 0.5 × minimum width of face (Fig. 298); length of malar space in anterior view 0.7–1.0 × maximum width of hypoclypeal depression (Fig. 298); head in anterior view trapezoid; length of eye 1.3 × temple in dorsal view and temples directly narrowed behind eyes (Fig. 299); vertex behind stemmaticum densely rugose; clypeus near lower level of eyes; length of malar space 0.4 × length of eye in lateral view.

***Mesosoma.*** Mesoscutal lobes distinctly punctate-granulate, and with satin sheen; precoxal area of mesopleuron coarsely rugose, rather wide medially and posteriorly coarsely punctate and some short rugae, densely punctate; remainder of mesopleuron mainly sparsely and finely punctate; metapleuron densely punctate; metanotum with nearly complete median carina; scutellum punctulate and weakly granulate; propodeum coarsely vermiculate-rugose, medio-longitudinal carina irregular.

***Wings.*** Fore wing: r 0.4 × 3-SR (Fig. 292); m-cu far antefurcal; 1-CU1 horizontal, slightly widened, 0.3 × 2-CU1; r-m 0.7 × 3-SR; 2^nd^ submarginal cell medium-sized (Fig. 292); cu-a inclivous, somewhat curved posteriorly; 1-M rather curved posteriorly; 1-SR wide; surroundings of M+CU1, 1-M and 1-CU1 largely glabrous. Hind wing: marginal cell gradually widened, its apical width 2.6 × width at level of hamuli (Fig. 292); 2-SC+R subquadrate; m-cu medium-sized and only pigmented; M+CU:1-M = 50:43; 1r-m 0.7 × 1-M.

***Legs.*** Tarsal claws slender and brownish setose (Fig. 302); hind coxa rather finely and densely punctate; hind trochantellus robust; length of hind femur and basitarsus 5.0 and 6.5 × their width, respectively; length of inner hind spur 0.45 × hind basitarsus.

***Metasoma.*** First tergite rather flattened, 1.1 × longer than wide apically; 1^st^ and 2^nd^ tergites with medio-longitudinal carina and rather regularly longitudinally rugose; medio-basal area of 2^nd^ tergite narrow triangular (Fig. 295); 2^nd^ suture deep and crenulate; basal half of 3^rd^ tergite longitudinally striate, remainder of metasoma smooth; 4^th^ and apical half of 3^rd^ tergite without sharp lateral crease; ovipositor sheath wide, with medium-sized setae and apically truncate (Fig. 290).

***Colour.*** Black; palpi and basal half of antenna (except scapus and pedicellus) brown; scapus, pedicellus, clypeus largely, apex of hind femur (but ventrally reddish), apex of hind tibia, hind tarsus, all telotarsi, pterostigma (but basally narrowly pale) and veins (except yellowish veins of basal quarter of wings) dark brown; remainder of legs and 1^st^–3^rd^ tergites orange brown; tegulae and hind tibia (except apically) pale yellowish; wing membrane subhyaline.

***Variation.*** Coxae and hind femur (except its basal third) largely dark brown, black or orange brown; apical half of hind tibia dark brown or only apically so; 1^st^ tergite largely dark brown (except posteriorly), with pair of dark brown spots or entirely orange or reddish brown; apical half of 3^rd^ tergite orange brown or largely black. Antennal segments: ♀ 54(1), 55(2), 57(2), 58(3), 59(1), 60(2); ♂ 52(1), 60(1). Male is very similar and has apical tergites type 1–2, setae moderately dense, glabrous stripe only rarely evident and fringe very short, negligible (Figs 303, 305).

##### Distribution.

China, *Italy, Mongolia, Russia (Siberia and Far East), Spain, *Turkey.

**Figures 289–291.**
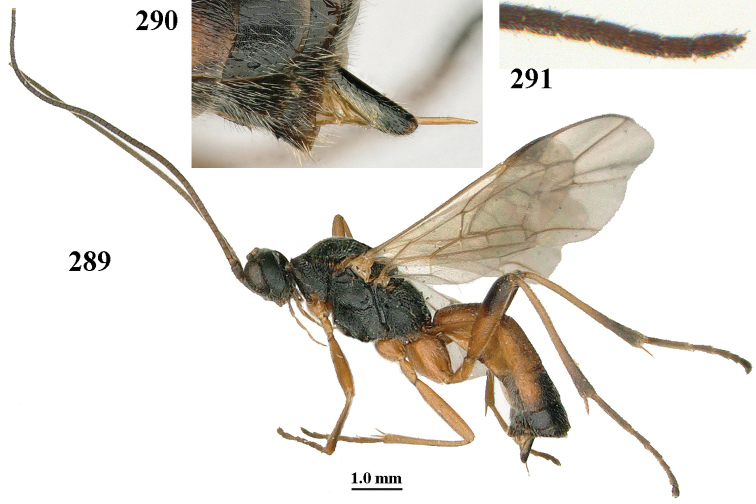
*Aleiodes eurinus* (Telenga), ♀, Mongolia, but 291 Russia, Chelyabinskoi Obl. **289** habitus lateral **290** ovipositor sheath lateral **291** apex of antenna.

**Figures 292–302.**
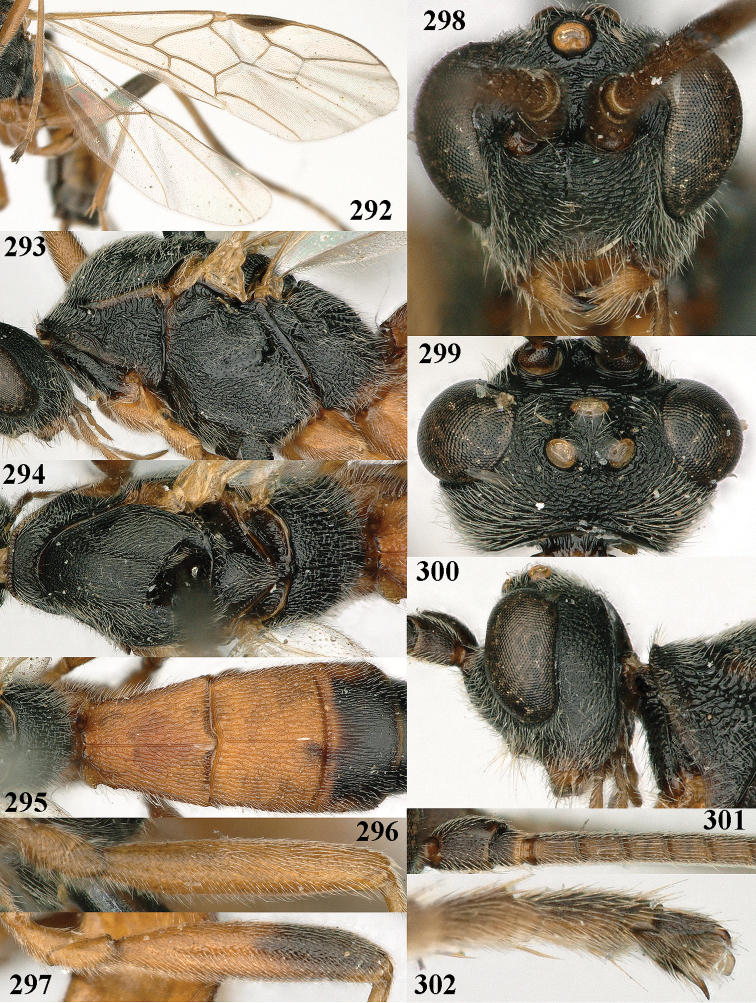
*Aleiodes eurinus* (Telenga), ♀, Mongolia **292** wings **293** mesosoma lateral **294** mesosoma dorsal **295** 1^st^–3^rd^ metasomal tergite dorsal **296** fore femur lateral **297** hind femur lateral **298** head anterior **299** head dorsal **300** head lateral **301** base of antenna **302** outer hind tarsal claw.

**Figures 303–306.**
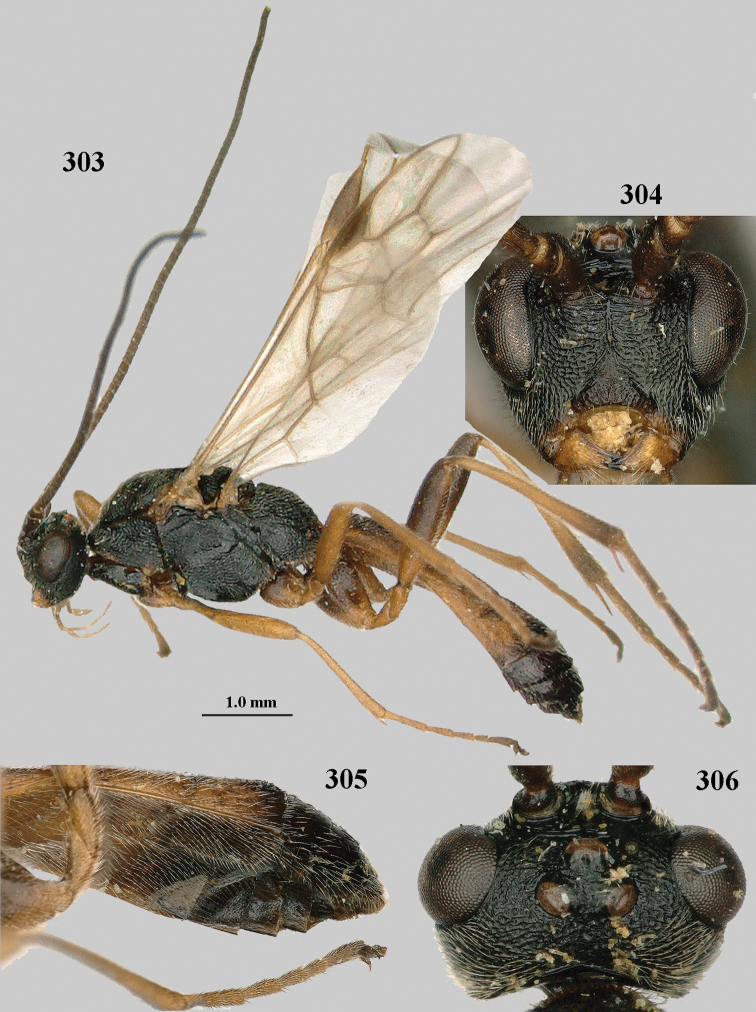
*Aleiodes eurinus* (Telenga), ♂, Mongolia **303** habitus lateral **304** head anterior **305** apical half of metasoma lateral **306** head dorsal.

#### Aleiodes fahringeri

Taxon classificationAnimaliaHymenopteraBraconidae

(Telenga, 1941)

69C3C3F4-C019-54A9-BCE3-C4C0073CBB92

Figs 307–309 , 310–321

Rhogas (Rhogas) fahringeri Telenga, 1941: 173.
Rogas fahringeri ; Shenefelt 1975: 1228.
Rogas (Rogas) fahringeri ; Papp 1977b: 113.
Aleiodes (Chelonorhogas) fahringeri ; Chen and He 1997: 40; He et al. 2000: 666; Belokobylskij 2000: 39 (lectotype designation); Papp 2009: 149.
Aleiodes fahringeri ; Chen and He, 1992: 125; Fortier and Shaw 1999: 230.
Rhogas (Rhogas) flavipennis Telenga, 1941: 174, 419.
Rogas flavipennis ; Shenefelt 1975: 1229.
Aleiodes (Chelonorhogas) flavipennis ; Belokobylskij 2000: 39 (lectotype designation and synonymised with A. fahringeri (Telenga, 1941)).

##### Type material.

None examined.

##### Additional material.

3 ♀ (MTMA, NMS, RMNH), “**Mongolia**: Südgobi aimak, Somon Bulgan, Talyn bulag, 1350 m, Exp. Dr. Z. Kaszab, 1967”, “Nr. 889, 5.vii.1967”, “*Rogas fahringeri* Tel., ♀, det. Papp, 1976”; 1 ♀ (RMNH, ZJUH), “**China**: Ningxia, Yinchuan, 6.vii.1983, no. 840994, Xu Wenzhong, RMNH’99”.

##### Molecular data.

None.

##### Biology.

Unknown. Specimens have been collected in June–August. Presumed to be univoltine, but we have not seen reared material and the means of overwintering is unclear.

##### Diagnosis.

**Figures 307–309.**
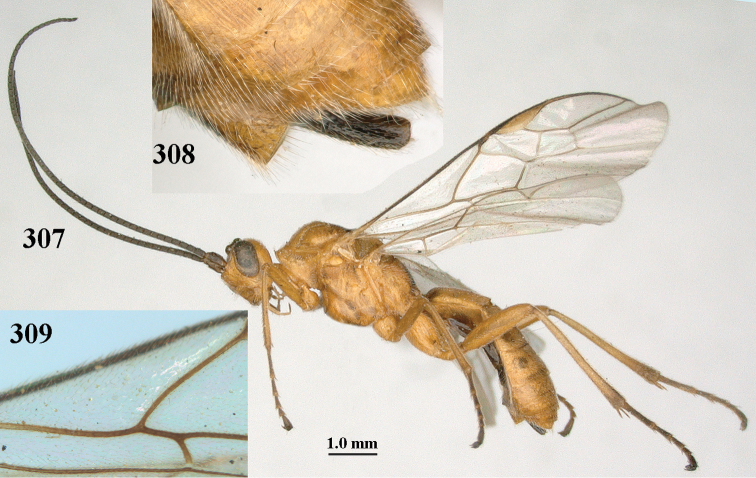
*Aleiodes fahringeri* (Telenga), ♀, Mongolia, Somon Bulgan **307** habitus lateral **308** ovipositor sheath lateral **309** detail of fore wing.

Maximum width of hypoclypeal depression 0.6–0.7 × minimum width of face; OOL 0.9 × diameter of posterior ocellus, largely smooth with spaced punctures; ventral margin of clypeus thin, anterior part shiny and distinctly protruding anteriorly (Fig. 320); mesoscutum shiny and moderately punctulate; precoxal area with only some rugulae medially; vein r of fore wing 0.5–0.6 × as long as vein 3-SR; tarsal claws rather slender and with yellowish or brown pecten, pecten remains removed from apical tooth (Fig. 321); hind tarsus fairly elongate and segment with medium-sized apical spines (Figs 307, 321); pterostigma brownish yellow; wings subhyaline; head and mesosoma laterally and dorsally (except more or less dark brown propodeum) yellowish brown; fore wing longer than 5 mm.

##### Description.

Redescribed ♀ (RMNH) from Mongolia (Somon Bulgan). Length of fore wing 6.9 mm, of body 7.7 mm.

***Head.*** Antennal segments of ♀ 58, length of antenna 1.1 × fore wing, its basal and subapical segments slender (Figs 316, 317); frons largely smooth anteriorly and rugulose posteriorly; OOL 0.9 × diameter of posterior ocellus, largely smooth with spaced punctures, (but superficially coriaceous near eye) and with satin sheen; vertex largely smooth, but superficially rugulose behind ocelli; anterior part of clypeus nearly 5 × wider than long, medially distinctly wider than laterally, largely smooth, punctulate, its ventral margin thin and protruding forwards (Fig. 320); width of hypoclypeal depression 0.7 × minimum width of face (Fig. 318); length of eye 1.6 × temple in dorsal view (Fig. 319); clypeus near lower level of eyes; length of malar space 0.2 × length of eye in lateral view.

***Mesosoma.*** Pronotum medio-dorsally flat, shiny and largely smooth; mesoscutal lobes largely smooth except for punctulation, shiny and densely setose; precoxal area of mesopleuron largely smooth medially, with only some superficial rugulae; remainder of mesopleuron finely punctate and antero-dorsally rugose; metapleuron remotely punctate and largely smooth medially; scutellum remotely punctulate; metanotum with fine complete median carina; propodeum weakly convex and densely rugose, its medio-longitudinal carina complete and fine.

**Figures 310–321.**
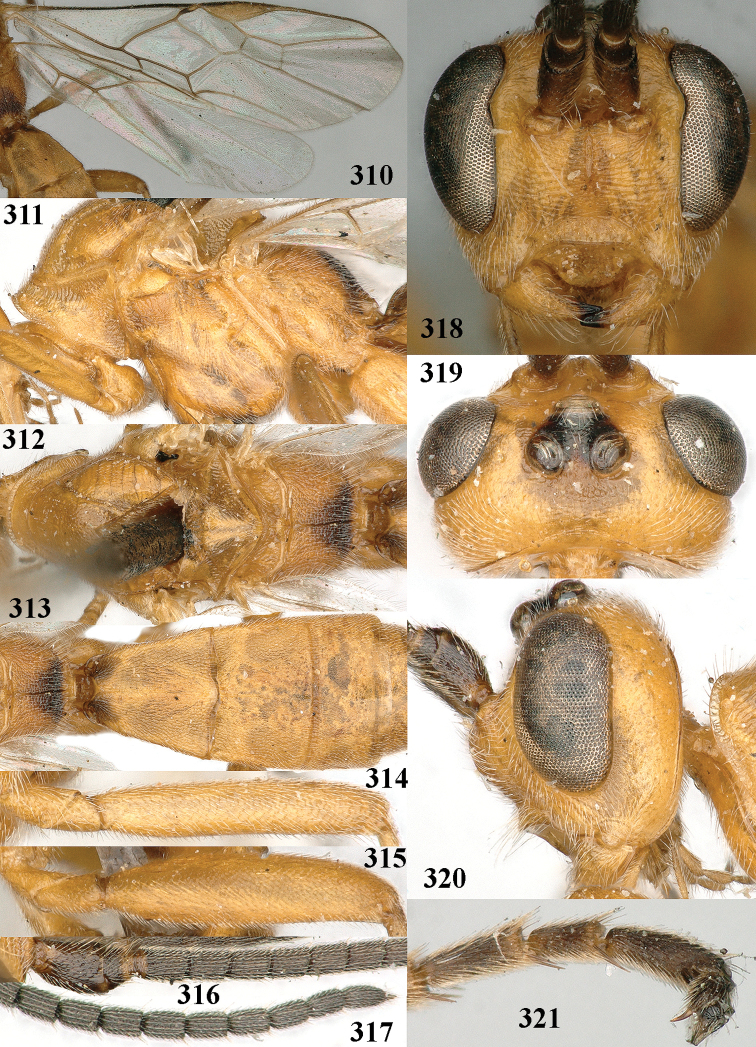
*Aleiodes fahringeri* (Telenga), ♀, Mongolia, Somon Bulgan **310** wings **311** mesosoma lateral **312** mesosoma dorsal **313** propodeum and 1^st^–3^rd^ metasomal tergites dorsal **314** fore femur lateral **315** hind femur lateral **316** base of antenna **317** apex of antenna **318** head anterior **319** head dorsal **320** head lateral **321** inner hind tarsal claw

***Wings.*** Fore wing: r 0.5 × 3-SR (Fig. 310); 1-CU1 horizontal, 0.3 × 2-CU1; r-m 0.7 × 3-SR; 2^nd^ submarginal cell medium-sized (Fig. 310); cu-a inclivous, straight; 1-M nearly straight posteriorly; 1-SR somewhat widened; surroundings of M+CU1, 1-M and 1-CU1 evenly setose but setae pale and easily overlooked. Hind wing: marginal cell linearly widened, its apical width 2.4 × width at level of hamuli (Fig. 310); 2-SC+R short and longitudinal; m-cu vaguely indicated; M+CU:1-M = 10:7; 1r-m 0.8 × 1-M.

***Legs.*** Tarsal claws with rather inconspicuous and pale brownish pecten remaining far removed from apical tooth (Fig. 321); hind coxa largely superficially finely punctate, but dorso-anteriorly densely punctate; hind trochantellus rather robust; length of hind femur and basitarsus 4.4 and 6.0 × their width, respectively; length of inner hind spur 0.5 × hind basitarsus.

***Metasoma.*** First tergite rather flat, 1.1 × as long as wide apically; 1^st^ and 2^nd^ tergites with fine medio-longitudinal carina and finely longitudinally (1^st^) or irregularly (2^nd^) densely rugose; medio-basal area of 2^nd^ tergite triangular and medium-sized (Fig. 313); 2^nd^ suture rather deep, finely crenulate and narrow; basal half of 3^rd^ tergite finely rugulose, remainder of metasoma superficially micro-sculptured or nearly smooth; 4^th^ and apical half of 3^rd^ tergite without sharp lateral crease; ovipositor sheath wide, with medium-sized setae and apically truncate (Fig. 308).

***Colour.*** Yellowish brown; antenna (except dark brown scapus and pedicellus), stemmaticum and ovipositor sheath black; tarsi, medio-posterior patch of propodeum, basal patch of 1^st^ tergite and apex of hind tibia dark brown; veins rather dark brown at medial third of fore wing, remainder of veins pale brown or yellowish; pterostigma brownish yellow; wing membrane subhyaline.

***Variation.*** Scapus entirely dark brown or largely yellowish brown; dark patches of propodeum and 1^st^ tergite sometimes absent (♀ RMNH from Ningxia). Antennal segments: ♀ 56(2), 58(2), 59(1); ♂ 57(1), 59(1). Male is very similar with apical tergites type ?1–2, setae short, sparse and hard to see, with fringe very short and negligible.

##### Distribution.

China (Ningxia), Mongolia.

##### Notes.

This Asian species is included here because it was reported from Poland (Huflejt, 1997). The record needs confirmation to rule out confusion with a similar European species.

#### Aleiodes fortipes

Taxon classificationAnimaliaHymenopteraBraconidae

(Reinhard, 1863)

543DE8ED-DC0F-5A62-8241-D34B299DFD30

Figs 322–324 , 325–338 , 339–342

Rogas fortipes Reinhard, 1863: 272; Shenefelt 1975: 1229 [examined].
Aleiodes (Neorhogas) fortipes ; Papp 1985a: 158, 1987a: 333, 1987b: 35, 1991a: 75.
Aleiodes fortipes ; Papp 2005: 176; Lozan et al. 2010: 17; Butcher et al. 2012: 14.
Rhogas freyi Hellén, 1927: 25–26; Papp 1985a: 158 (unnecessary lectotype designation and as synonym of A. fortipes), 2005: 176 [examined].
Rogas freyi ; Shenefelt 1975: 1229–1230; Tobias 1986: 75 (transl.: 121).

##### Type material.

Holotype of *A. fortipes*, ♂ (ZMB), “Gallia [**France**]”, “Type”, “Coll. H. Rhd.”, “26723”, “*fortipes* Rhd.”, “Holotypus”, “*Rogas fortipes* Reinh., 1863, ♂, Papp, 1983.”. Holotype of *A. freyi*, ♂ (ZMH), “[**Finland**], Nagu”, “R. Frey”, “*Freyi* n. sp., Hellén det.”, “Mus. Zool. H:fors, sp. typ. No. 5363, *Rhogas Freyi* Hellén”, “Lectotypus *Rogas freyi* Hellén, design. Tobias”, “*Aleiodes* % ♂ *fortipes* Rh., det. Papp J., 1983/ compared with ♂ holotype of *A. fortipes*”. The lectotype designation is superfluous because it is evident from the description that the author had only one male.

##### Additional material.

Austria, British Isles (England: V.C.s 16, 26, 28), Bulgaria, Czech Republic, Finland, France, Germany, Hungary, Netherlands (GE: ‘t Harde, Nunspeet), Poland, Spain, Sweden, Turkey. Specimens in ZJUH, BZL, CMIM, FC, MTMA, NMS, RMNH, SDEI. It has been collected in open or understory habitats, including (but not exclusively) growths dominated by *Vaccinium* and/or *Calluna* below sparse conifers. Generally, found on sandy well-drained soils in England (Breck heaths of East Anglia) and the Netherlands (Veluwe).

##### Molecular data.

MRS650 (France), MRS807 (Poland).

##### Biology.

The flight time of this univoltine species is (April)May–June, and ca 10 months of the year is spent as an exposed mummy. The only mummy seen (Fig. 324) formed in captivity firmly attached beneath a thin stem and would have been positioned low down in the vegetation, but probably aerially. It is light brown, moderately slender, and the parasitoid occupied approximately abdominal segments 3–8. The host was *Idaea* sp. (Geometridae), either *I. aversata* (Linnaeus) or *I. straminata* (Borkhausen), and the rearing arose when a few larvae of the foregoing were collected (MRS) along with an adult female of *A. fortipes* at the same site in Poland (22.v.2016) and offered to the parasitoid, which had been fed honey water, on 24.v.2016. Although two of the caterpillars were well-grown, in their final instars and at least twice as long as the parasitoid, one was accepted avidly. This host was first pricked several times, at intervals. Paralysis was rather slow to take effect and not complete until after the host was revisited for oviposition: a single insertion of ca 30 seconds duration, with no post-oviposition association (the parasitoid simply walked away after oviposition). The host mummified on 9.vi.2016 and an adult female emerged on 22.v.2017. The other host was rejected after being pricked just once, and later died. A penultimate instar caterpillar of the same host aggregate was also parasitised but died after an ecdysis. Subsequent barcoding (through the kindness of Axel Hausmann, ZSSM) of the dead caterpillars revealed one specimen each of *I. aversata* and *I. straminata*, leaving the precise determination of the successful host unclear. It is possible that the parasitised host had already been attacked before it was collected, but the rather long time before mummification occurred suggests not. In any case, at least one *Idaea* species in the *aversata*/ *straminata* group clearly serves as host. Some individuals of the long and slender, morphologically very different, larvae of *Idaea muricata* (Hufnagel) were also offered. Although possibly of less interest to the parasitoid, one penultimate instar larva (1.7 times the length of the female parasitoid) was immediately parasitised (a single prick for eventual paralysis, followed after an interval by a single insertion for oviposition lasting just more than a minute), but this larva later produced a moth. Final instars of this very elongate species of caterpillar were generally ignored, but one did elicit a downwards curl of the metasoma without, however, being stung.

There are two particularly significant aspects to the successful rearing. The first is that these *Idaea* species overwinter as quite well-grown larvae, so during the flight period of the parasitoid they are in late instars, and attacking hosts at this stage is an unusual strategy for *Aleiodes* (but see *A. aterrimus* and *A. sibiricus*). The second is that we know of no other *Aleiodes* species apart from *A. sibiricus* (q. v.) among those whose host overwinters as a larva that fails to take advantage of that to overwinter as an early instar larva within it. The apparently riskier strategy taken by *A. fortipes*, in both respects, may be plesiomorphic.

*Aleiodes fortipes* is the only known West Palaearctic species in which males have small, subapical setose pore (probably associated with pheromone release) situated mid-dorsally on each of the 4^th^–6^th^ metasomal tergites (Fig. 340). We also expect these pores to be present in *A. caucasicus*, which is only doubtfully distinct from *A. fortipes*, but we have not seen the male of *A. caucasicus*. Similar, probably homologous, pores are also a feature of males of Aleiodes (Hemigyroneuron) species which are found in the near East, Oriental and Afrotropical regions (Butcher & Quicke, 2015). Outside of *Hemigyroneuron*, metasomal pores are also found the New World *Aleiodes cameronii* (Dalla Torre) species complex and in a number of undescribed Madagascan *Aleiodes*. In *Hemigyroneuron* the pores have been shown to connect with large sub-tergal glands (Butcher & Quicke, 2011). Collectively these taxa form a basal clade in our molecular phylogeny (Fig. 1).

**Figures 322–324.**
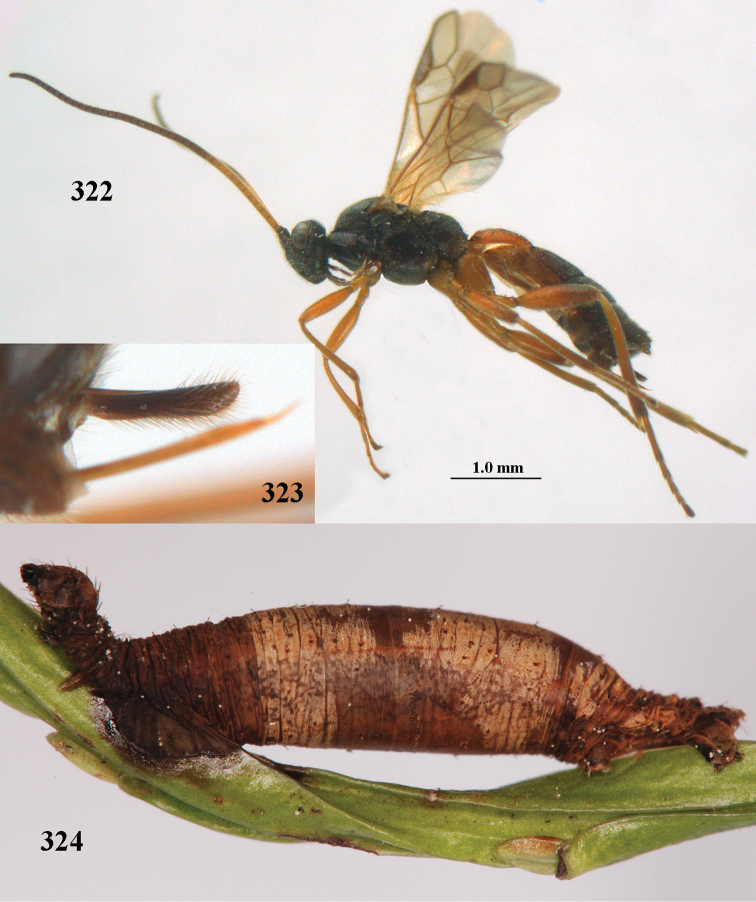
*Aleiodes fortipes* (Reinhard), ♀, England, Santon Downham **322** habitus lateral **323** ovipositor sheath lateral **324** mummy of *Idaea* sp. (either *I. aversata* (Linnaeus) or *I. straminata* (Borkhausen)), Poland, Dybki.

*Aleiodes fortipes* is the only species among those treated in this part of our revision with known hosts outside the Noctuidae and, although no host is known for rather a lot of these species, the apparently basal position of *A. fortipes* in the group is noteworthy and using geometrid hosts may also be plesiomorphic. The rather slender ovipositor sheath (Fig. 322) is another indication for its basal position. It is interesting that the known hosts of both *A. fortipes* and of the subgenus Hemigyroneuron are all Geometridae (two species of *Hemigyroneuron* with examined mummies, India and S. Africa, cited by Butcher & Quicke, 2011 [a label record indicating a pierid host of a 3^rd^ species is also cited in that paper but is discounted here because no mummy was present]). An Australian species described under *Hemigyroneuron* with examined mummy reported to be that of a geometrid by Butcher & Quicke (2016) is probably (a) actually not a member of A. (Hemigyroneuron) and (b) may be from a lasiocampid (W. Moore in litt.). The hosts of members of the *Aleiodes cameronii* complex, based on multiple rearings in both North America and Costa Rica include both Geometridae and Erebidae (Eiseman & Charney, 2010; http://v4.boldsystems.org).

##### Diagnosis.

Maximum width of hypoclypeal depression approx. 0.3 × minimum width of face (Fig. 332); 2^nd^–10^th^ antennal segments yellowish, contrasting with remaining darker segments; clypeus obtuse apically and not protruding in lateral view (Fig. 334); precoxal area largely smooth, at most with some aciculae or punctures medially (Fig. 327); tegulae brown; lobes of mesoscutum finely coriaceous-granulate and rather dull, with satin sheen; vein 1-CU1 of fore wing 0.4–0.6 × vein 2-CU1 (Fig. 332); length of hind femur 3.5–3.8 × its maximum width (Fig. 331); hind tarsal claws small and only yellowish or brownish setose (Fig. 338); body of ♂ completely black, antenna completely blackish, dark brown or with some segments yellowish subbasally and 4^th^–6^th^ tergites with a setose medio-dorsal depression; length of fore wing 3.7–5.0 mm.

##### Description.

Holotype of *A. freyi*, ♂, length of fore wing 4.5 mm, of body 5.3 mm.

***Head.*** Antenna incomplete, (length of antenna of ♀ from Santon Downham 1.2 × fore wing, its subapical segments rather robust: Fig. 336); frons smooth anteriorly and with coarse curved striae posteriorly; OOL 2.7 × diameter of posterior ocellus, and rather regularly and rather coarsely striate; vertex transversely striate, rather shiny; clypeus narrow, strongly curved dorsal margin, rugulose; ventral margin of clypeus thick and not protruding forwards (Fig. 334); width of hypoclypeal depression 0.3 × minimum width of face (Fig. 332); length of eye 1.4 × temple in dorsal view (Fig. 333); vertex behind stemmaticum transversely rugose; clypeus near lower level of eyes; face coarsely transversely rugose; length of malar space 0.4 × length of eye in lateral view.

***Mesosoma.*** Mesoscutal lobes very densely coriaceous-granulate, with vague micro-reticulate sculpture, matt; precoxal area of mesopleuron largely smooth (except some micro-sculpture) medially, rather depressed; remainder of mesopleuron largely smooth, except some punctures and antero-dorsally coarsely rugose; scutellum superficially granulate and with some punctures; propodeum coarsely rugose-reticulate and medio-longitudinal carina nearly complete.

**Figures 325–338.**
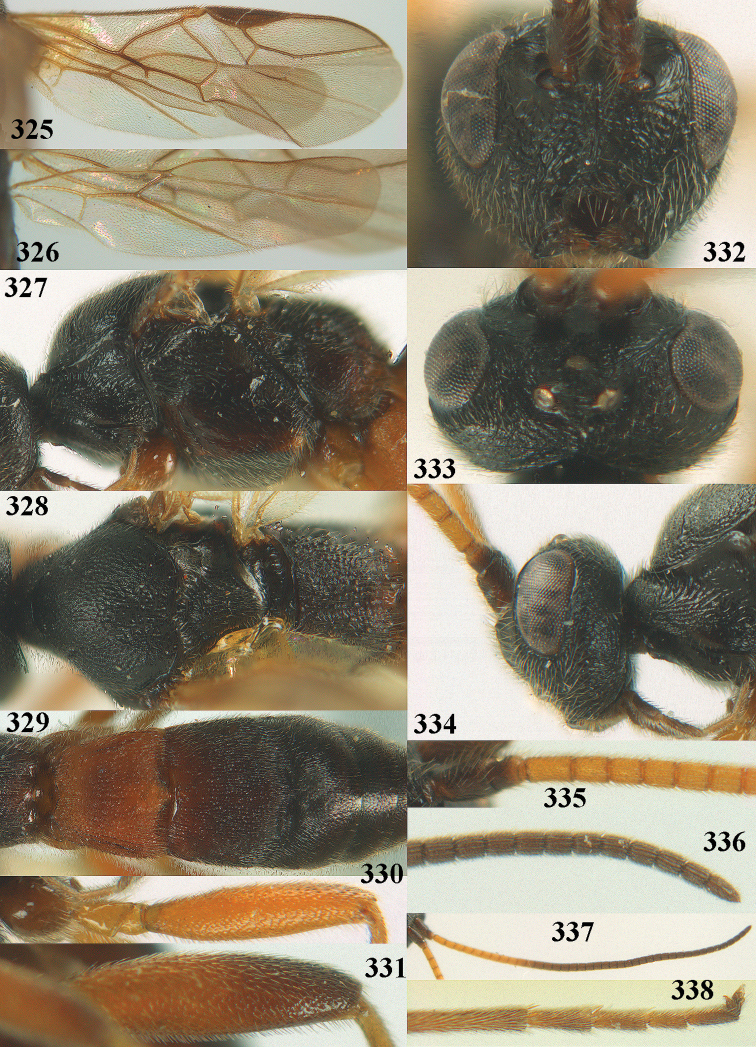
*Aleiodes fortipes* (Reinhard), ♀, England, Santon Downham **325** fore wing **326** hind wing **327** mesosoma lateral **328** mesosoma dorsal **329** 1^st^ –3^rd^ metasomal tergites dorsal **330** fore femur lateral **331** hind femur lateral **332** head anterior **333** head dorsal **334** head lateral **335** base of antenna **336** apex of antenna **337** antenna **338** inner hind tarsal claw.

***Wings.*** Fore wing: r 0.6 × 3-SR (Fig. 325); 1-CU1 horizontal and somewhat widened, 0.45 × 2-CU1; r-m 0.65 × 3-SR; 2^nd^ submarginal cell rather short (Fig. 325); cu-a vertical, straight; 1-M rather curved posteriorly; 1-SR short and narrow; surroundings of M+CU1, 1-M and 1-CU1 evenly setose; m-cu subvertical, slightly diverging from 1-M posteriorly. Hind wing: marginal cell linearly widened, its apical width 2.2 × width at level of hamuli (Fig. 326); 2-SC+R subquadrate; m-cu absent; M+CU:1-M = 80:57; 1r-m 0.6 × 1-M; 1-M straight.

***Legs.*** Tarsal claws small but robust and only yellowish setose (Fig. 338); hind coxa largely rugulose-granulate; hind trochantellus medium-sized; length of hind femur and basitarsus 3.8 and 7.6 × their width, respectively; length of inner hind spur 0.4 × hind basitarsus; fore femur 4.8 × as long as wide.

***Metasoma.*** First tergite evenly convex, 1.3 × as long as wide apically; 1^st^ and 2^nd^ tergites with weak medio-longitudinal carina and together with basal half of 3^rd^ tergite densely and finely longitudinally rugose; medio-basal area of 2^nd^ tergite narrow but rather distinct (Fig. 329); 2^nd^ suture rather shallow; remainder of metasoma superficially micro-sculptured; 4^th^ and apical half of 3^rd^ tergite without sharp lateral crease; 4^th^–6^th^ tergites with a setose medio-dorsal depression.

***Colour.*** Dark brown or blackish; palpi dark brown; mesopleuron with reddish brown streak; legs yellowish brown but tarsi, apex of hind femur (and indistinctly apices of fore and middle femora, and of tibiae) and base of hind coxa infuscate; tegulae and pterostigma brown; wing membrane slightly infuscate.

***Variation.*** Maximum width of marginal cell of hind wing 2.0–2.6 × its width near hamuli (Fig. 326); vein 1-CU1 of fore wing 0.4–0.6 × vein 2-CU1; length of hind femur 3.5–3.8 × its maximum width; length of 1^st^ tergite 1.0 (♀)–1.3 (♂) × its apical width. Propodeum and metapleuron posteriorly, 1^st^ and 2^nd^ tergites and base of 3^rd^ tergite of ♀ more or less brown, basal third of antenna (except scapus and base of pedicellus) brownish yellow or yellowish brown and ovipositor sheath rather slender, with long setae and apically narrowed (Fig. 323). Antennal segments: ♀ 38(1), 39(1), 41(1), 43(1), 44(1), 45(1); ♂ 36(1), 39(3), 40(6), 41(3), 42(7), 43(2), 44(2), 45(1). The number of antennal segments appear to be comparable between the sexes. Males have 1^st^–3^rd^ metasomal tergites completely black, and basal third of antenna completely blackish, dark brown or with some subbasal segments yellowish. The male apical tergites (besides pores, see above) are type 1, setae rather dense, no fringe observed and probably absent (Fig. 341).

##### Distribution.

*Austria, *British Isles (England), Bulgaria, Czech Republic, Finland, France, *Germany, Hungary, *Netherlands, *Poland, *Spain, *Sweden, *Turkey.

**Figures 339–342.**
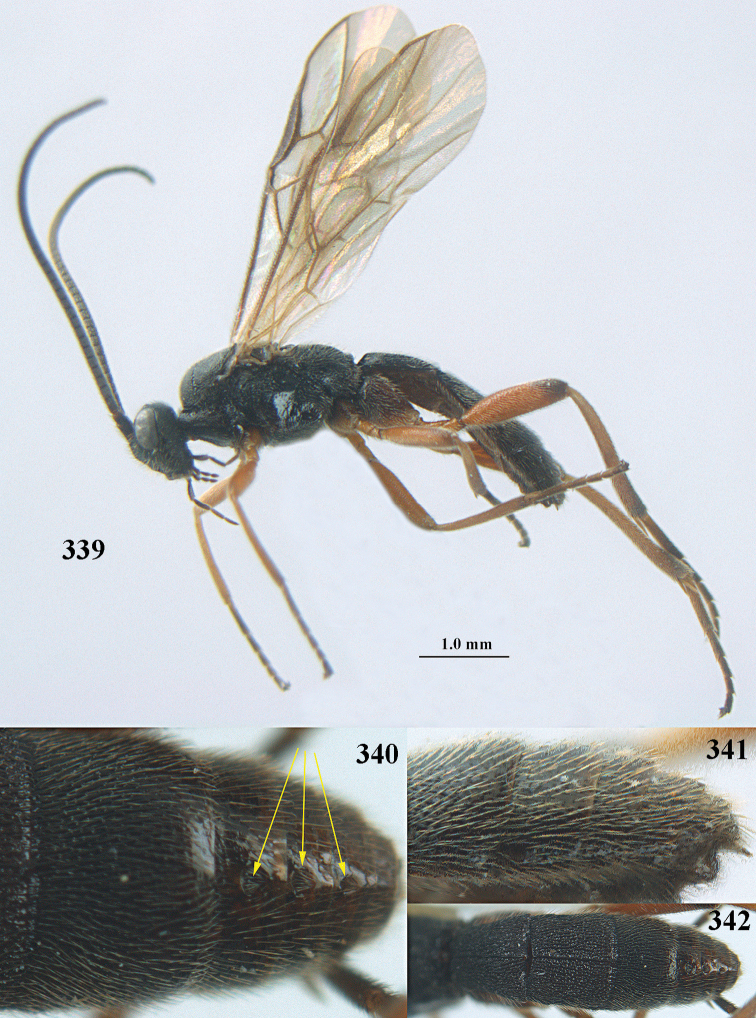
*Aleiodes fortipes* (Reinhard), ♂, England, Santon Downham **339** habitus lateral **340** 3^rd^–7^th^ metasomal tergites dorsal **341** id. lateral **342** metasoma dorsal. Arrows indicating setose depressions or pores of 4^th^–6^th^ tergites.

#### Aleiodes gasterator

Taxon classificationAnimaliaHymenopteraBraconidae

(Jurine, 1807)

0E8476B0-62DA-5838-80F9-B17814F7587A

Figs 343–346 , 347–359 , 360–364

Bracon gasterator Jurine, 1807: 118, pl. 8. [examined].
Rogas gasterator ; Shenefelt 1975: 1230–1231; Zaykov 1980a: 112.
Rogas (Rogas) gasterator ; Tobias 1976: 86, 1986: 81 (transl.: 133) p.p.
Aleiodes (Neorhogas) gasterator ; Papp 1991a: 91 p.p.
Aleiodes (Chelonorhogas) gasterator ; Falco et al. 1997: 60; Ghahari et al. 2011: 267; Rastegar et al. 2012: 3; Farahani et al. 2015: 243.
Aleiodes gasterator ; Bergamasco et al. 1995: 5; Zaldívar-Riverón et al. 2004: 234; Papp 2005: 176.
Bracon dimidiatus Spinola 1808: 123–124. Syn. nov.
Aleiodes dimidiatus ; Bergamasco et al. 1995: 5.
Rogas (Rogas) dimidiatus : Tobias 1976: 86; 1986: 81 (transl.: 134) p.p.
Rogas dimidiatus ; Zaykov 1980a: 112.
Aleiodes (Neorhogas) dimidiatus ; Papp 1991a: 90 p.p.
Aleiodes (Chelonorhogas) dimidiatus ; Samartsev and Belokobylskij 2013: 765; Farahani et al. 2015: 242.
Rhogas (Rhogas) dimidiatus var. turkestanicus Telenga, 1941: 184, 409; Shenefelt 1975: 1225 [examined]. Syn. nov.

##### Type material.

Holotype of *A. gasterator*, ♀ (Museum Genève), “[? **Switzerland**], *gasterator* J.”, “Typus”, “*Bracon gasterator* Jur., Type”, “Type du g. *Rogas* [= incorrect]”, “vu par [R.D.] Shenefelt, U.S.A., 1967” (metasoma on separate card and pin). Lectotype of *A. turkestanicus* here designated, ♀ (ZISP), “[**Turkmenistan**], Transcaspia, Bajram-aly, 17.viii.1930, T. Boguj/311, i.s.”, “*Rhogas dimidiatus* Spin. var. *turkestanica* [sic!] nov., N. Telenga det.”.

##### Additional material.

Albania, Cyprus, France (including Corsica), Greece (including Crete), Italy (including Sardinia, Sicily), North Macedonia, Portugal (including Madeira), Spain (including Mallorca, Menorca, Tenerife), Tunisia, Turkey, [Iraq, Jordan, Syria]. Specimens in ZJUH, BZL, CNC, MSC, MTMA, NMS, RMNH, ZSSM. Widespread in the Mediterranean region, where it tends to replace *A. ruficornis*.

##### Molecular data.

MRS046 (France), MRS048 (France), MRS892 (Spain).

##### Biology.

Collected chiefly in May–July and September–November, but specimens have occurred in every month of the year. Plurivoltine; there is no indication of a unique overwintering mode in the material seen. Reared from low-feeding Noctuidae: *Agrotis segetum* (Denis & Schiffermüller) (6 [6 ZJUH], Spain), *Agrotis* sp. (1 [ZJUH], Cyprus; W.R. Ingram), mixed *Agrotis* and *Spodoptera littoralis* (Boisduval) (1 [ZJUH], Cyprus; W.R. Ingram). The two mummies seen are rather different (Figs 345, 346), though it may be that neither overwintered; a small, pale and relatively slender one from *Agrotis* sp. produced a small male, while a more normal sized individual emerged from the larger, dark and stout mummy whose host was (even) less certain. Even in the latter case, the mummy is less keeled, less lined with silk and much more in relation to the size of the emerging adult than is the situation with the overwintering mummies of the *A. grassator*/ *carbonarius*/ *carbonaroides*/ *ruficornis* complex. The appearance of both mummies suggest that they would normally form below ground.

##### Diagnosis.

Maximum width of hypoclypeal depression 0.4–0.5 × minimum width of face (Fig. 355); OOL of ♀ 1.2–1.6 × as long as diameter of posterior ocellus (Fig. 356) and distinctly rugose or rugulose; antennal segments of ♀ 29–39, of ♂ 36–46(–51) (usually 39–43); antenna of ♀ 0.8–0.9 × fore wing; length of malar space of ♀ 0.5–0.6 × height of eye in lateral view (Fig. 357); clypeus thick apically and not protruding anteriorly (Fig. 357); lobes of mesoscutum densely punctate, interspaces largely smooth and shiny; posterior half of notauli deep; precoxal area coarsely vermiculate-rugose medially; marginal cell of fore wing of ♀ usually ending rather removed from wing apex (Fig. 347); vein 1-CU1 of fore wing 0.4–0.5 × as long as vein 2-CU1 (Fig. 347); hind tarsal claws yellowish or brownish bristly setose; third tergite with (faint) curved or antero-medially transverse rugulae or striae (Fig. 364) or largely smooth (sometimes with only longitudinal striae baso-laterally), often with distinct punctures laterally; hind femur at least apico-dorsally dark brown or black; inner and/or dorsal side of hind tibia (largely) yellowish or red; tegulae usually (partly) dark brown; pale males have nearly always frons medially and stemmaticum black; palpi usually brownish or yellowish, sometimes dark brown; 3^rd^ metasomal tergite frequently partly or completely reddish or yellowish; 4^th^ and 5^th^ tergites black.

##### Description.

Redescribed ♀ (RMNH) from France (Isle sur le Sorque). Length of fore wing 4.9 mm, of body 6.1 mm.

***Head.*** Antennal segments of ♀ 35, length of antenna 0.9 × fore wing, its subapical segments robust (Fig. 359), 4^th^ segment 1.2 × longer than wide; frons with coarse curved rugae; OOL 1.6 × diameter of posterior ocellus, and densely rugose or rugulose; vertex densely rugose, shiny; clypeus densely punctate; ventral margin of clypeus thick and not protruding forwards (Fig. 357); width of hypoclypeal depression 0.5 × minimum width of face (Fig. 355); length of eye 1.3 × temple in dorsal view (Fig. 356); vertex behind stemmaticum punctate-rugose; clypeus just below lower level of eyes; length of malar space 0.5 × length of eye in lateral view.

***Mesosoma.*** Mesoscutal lobes densely punctate, interspaces largely smooth with superficial granulation, shiny; precoxal area of mesopleuron evenly vermiculate-rugose medially, but only sparsely punctate posteriorly; metanotum without median carina; scutellum rather flat, sparsely punctate, but rugose laterally; propodeum coarsely vermiculate-rugose, medio-longitudinal carina nearly complete, and angulate latero-posteriorly.

***Wings.*** Fore wing: r 0.35 × 3-SR (Fig. 347); marginal cell distinctly ending basad of level of apex of vein 3-M; 1-CU1 horizontal, slender, 0.5 × 2-CU1; r-m 0.6 × 3-SR; 2^nd^ submarginal cell medium-sized (Fig. 347); cu-a inclivous, posteriorly curved; 1-M rather curved posteriorly; 1-SR wide; surroundings of M+CU1, 1-M and 1-CU1 largely glabrous. Hind wing: marginal cell gradually widened, but slightly basally, its apical width 2.3 × width at level of hamuli (Fig. 348); 2-SC+R subquadrate; m-cu largely absent; M+CU:1-M = 28:19; 1r-m 0.6 × 1-M.

***Legs.*** Tarsal claws robust and with only bristly brownish setae (Fig. 358); hind coxa largely densely punctate; hind trochantellus robust; length of hind femur and basitarsus 4.2 and 5.8 × their width, respectively; length of inner hind spur 0.5 × hind basitarsus.

***Metasoma.*** First tergite convex medially and 0.9 × as long as wide apically; 1^st^ and 2^nd^ tergites with medio-longitudinal carina and coarsely longitudinally rugose; medio-basal area of 2^nd^ tergite short and rather distinct (Fig. 351); 2^nd^ suture deep; subbasally 3^rd^ tergite with faint curved striae and medially transverse (Fig. 364); remainder of metasoma superficially micro-sculptured; 4^th^ and apical half of 3^rd^ tergite without sharp lateral crease; ovipositor sheath wide, with medium-sized setae and apically oblique, dorsally longer than ventrally (Fig. 344).

***Colour.*** Black; face (except medio-dorsally), malar space, dorsal half of temple, frons largely laterally, notauli, mesoscutum laterally, scutellum, pronotum postero-dorsally, mesopleuron dorsally and posteriorly, metapleuron largely, 1^st^ and 2^nd^ metasomal tergites and base of 3^rd^ tergite orange brown; palpi and humeral plate and veins of hind wing yellowish brown; tegula rather dark brownish; ventral half of temple largely, dorso-apical patch of hind femur, pterostigma and veins of fore wing dark brown; fore wing membrane slightly infuscate, of hind wing subhyaline.

***Variation.*** A very colour-variable species; head and mesoscutum of female may be largely black (nominate form) or reddish (= “*A. dimidiatus* / var. *turkestanicus*”, but especially the mesoscutum may be intermediate). Especially males may have the hind coxa black and most of hind tibia dark brown, sometimes the entire leg is nearly completely black or dark brown. Antennal segments: ♀ 29(1), 31(2), 32(3), 33(13), 34(13), 35(15), 36(9), 37(9), 38(3), 39(2); ♂ 36(2), 37(3), 38(4), 39(8), 40(13), 41(9), 42(5), 43(11), 44(7), 45(2), 46(2). Additionally, an exceptionally large male with 50 segmented antennae from Cyprus (ZJUH) appears to belong to this species, as does a female from Spain (RMNH) with 41 antennal segments, basal half of 3^rd^ tergite largely obliquely rugose and blackish scapus. On average males have ca 7 more antennal segments than females. Males are very similar with apical tergites type 1–2, setae rather sparse and with evident but short fringe (Fig. 360), hind tibial spurs often blunt apically and 3^rd^ tergite remotely punctate basally.

##### Distribution.

*Albania, *Cyprus, France (including Corsica), Greece (including Crete), *Iraq, Italy (including Sardinia, Sicily), *Jordan, *North Macedonia, *Portugal (including Madeira), Spain (including Mallorca, Menorca and Tenerife), *Syria, *Tunisia, *Turkmenistan, Turkey.

##### New synonymy.

The new synonymy of Rhogas dimidiatus var. turkestanicus Telenga, 1941, is based on direct comparison of the types of both taxa. The identity of *Bracon dimidiatus* Spinola, 1808, is problematic because the holotype from Italy (Genoa) is lost and the original description is far too incomplete for an easy identification. Its colour pattern (head completely yellowish, hind tibia and 3^rd^ tergite reddish) does not fit with *A. ruficornis* (Herrich-Schäffer); if the head is largely reddish brown then the temple ventrally and malar space remain blackish. This pattern agrees better with that of pale specimens of *A. gasterator* (named as A. dimidiatus var. turkestanicus Telenga, 1941). *Aleiodes ruficornis* occurs also in Italy, but its females have the head partly black ventrally, the apex of the hind tibia dark brown and most of the 3^rd^ metasomal tergite black. Therefore, we synonymise *Bracon dimidiatus* with *A. gasterator* (syn. nov.). The holotype of *Bracon gasterator* Jurine, 1807, has the 3^rd^ metasomal tergite finely curved (nearly circular) aciculate or striate basally, palpi (as far as present) pale brownish, maximum with of hypoclypeal depression 0.45 × minimum width of face, vein 1-CU1 of fore wing half as long as vein 2-CU1 and 4^th^ antennal segment 1.3 × as long as wide. *Aleiodes arnoldiisensu*Farahani et al. (2015) concerns a species closely related to *A. gasterator* (Jurine) having basal half of 3^rd^ tergite coarsely longitudinally rugose, antenna of ♀ with 30–35 segments (of ♂ 36), head linearly narrowed ventrally in anterior view and subbasal antennal segments of ♀ slightly slenderer.

**Figures 343–346.**
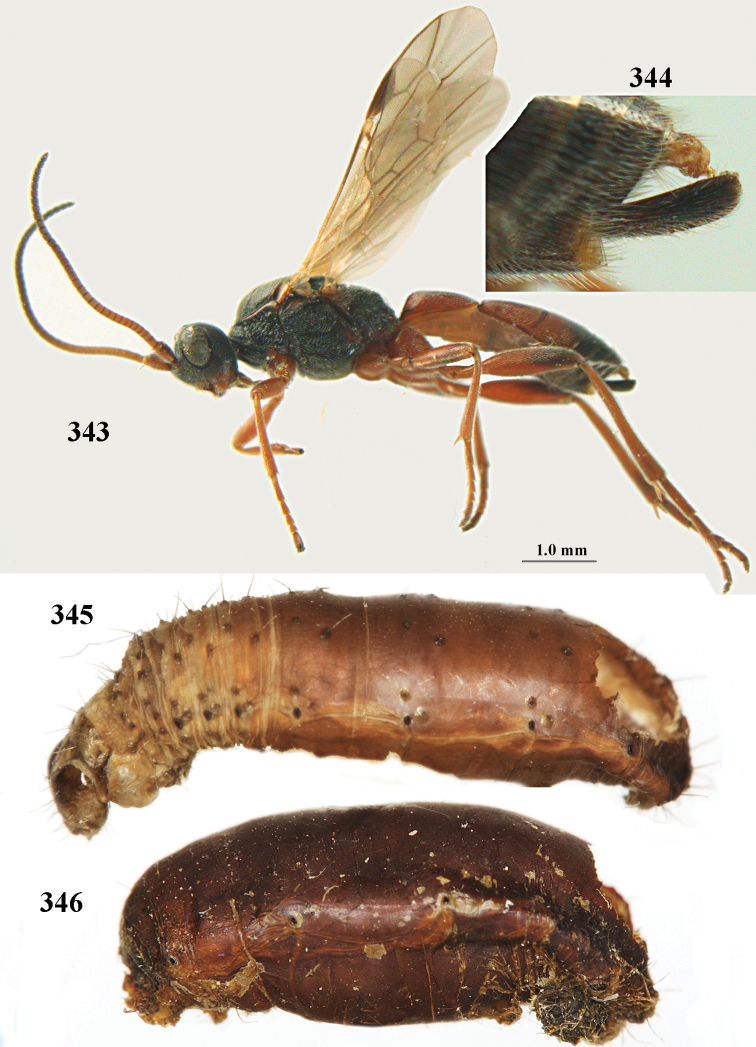
*Aleiodes gasterator* (Jurine), ♀, France, Les Constants, but 345 and 346 from Cyprus **343** habitus lateral **344** ovipositor sheath lateral **345** mummy of *Agrotis* sp. **346** mummy of *Agrotis* sp. or *Spodoptera littoralis* (Boisduval).

**Figures 347–359.**
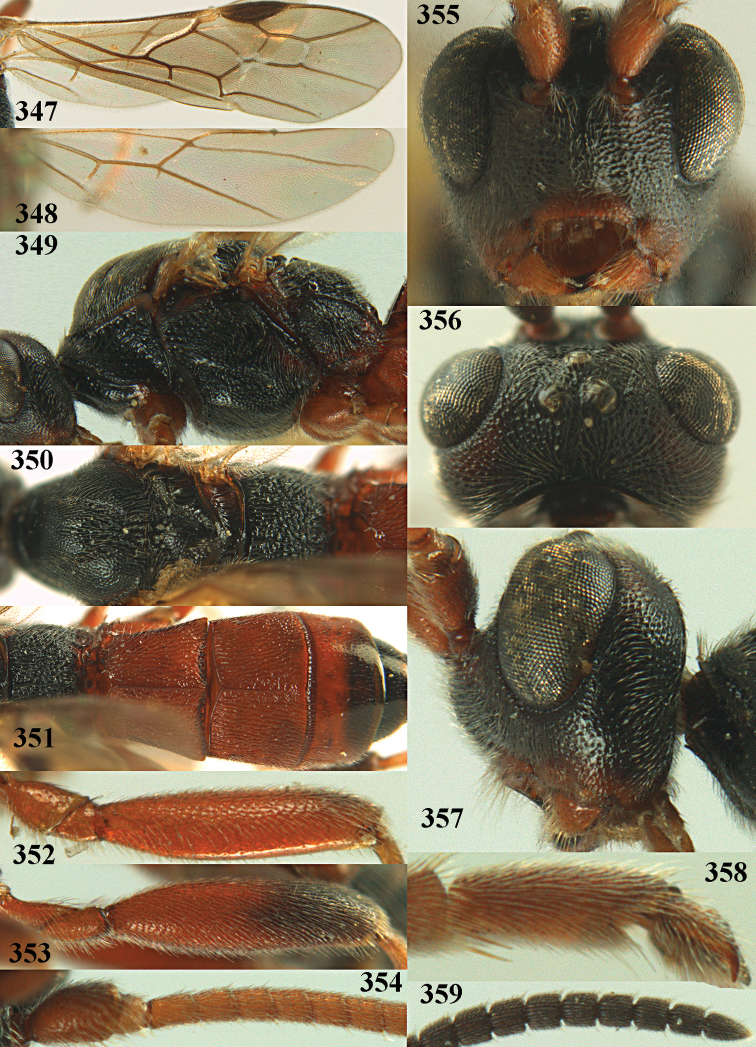
*Aleiodes gasterator* (Jurine), ♀, France, Les Constants **347** fore wing **348** hind wing **349** mesosoma lateral **350** mesosoma dorsal **351** metasoma dorsal **352** fore femur lateral **353** hind femur lateral **354** base of antenna **355** head anterior **356** head dorsal **357** head lateral **358** outer hind tarsal claw **359** apex of antenna.

**Figures 360–364.**
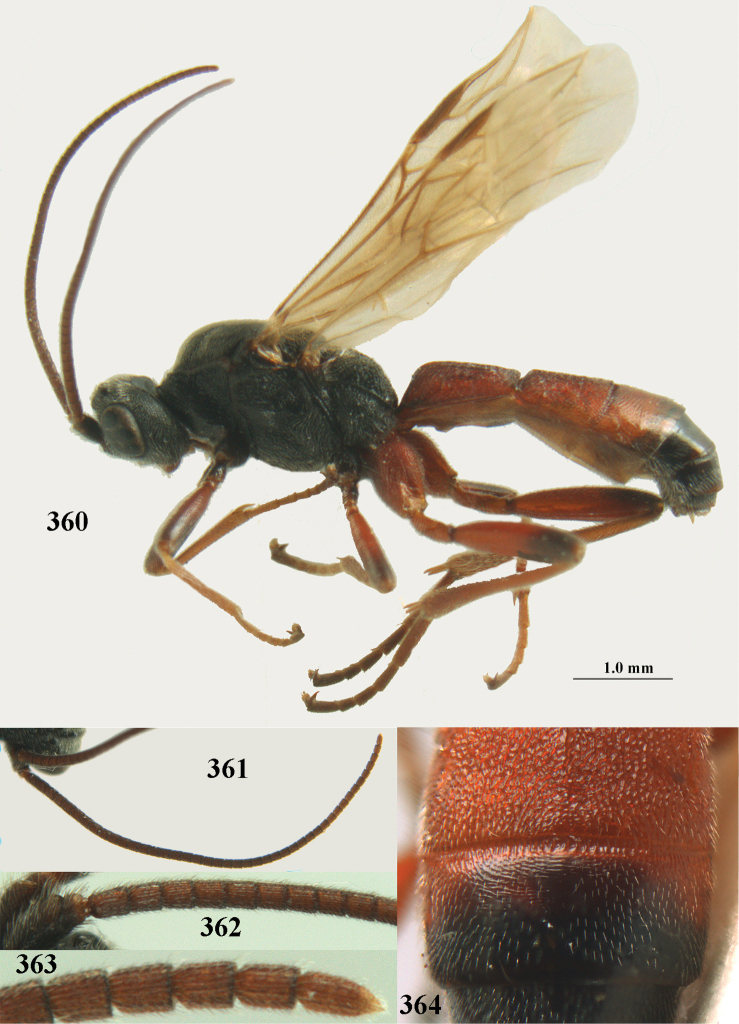
*Aleiodes gasterator* (Jurine), ♂, Italy, Livorno, but 364 of ♀, France **360** habitus lateral **361** antenna **362** base of antenna **363** apex of antenna **364** 3^rd^ metasomal tergite dorsal.

#### Aleiodes grassator

Taxon classificationAnimaliaHymenopteraBraconidae

(Thunberg, 1822)

B81F3B1B-63AE-54A5-B6FB-EFC49244C7EC

Figs 365–367 , 368–380 , 381–384

Ichneumon grassator Thunberg, 1822: 256 [examined].
Rogas grassator ; Shenefelt 1975: 1232.
Aleiodes (Neorhogas) grassator ; Papp 1991a: 86.
Aleiodes grassator ; Zaldívar-Riverón et al. 2004: 234; Quicke et al. 2014: 240; Butcher et al. 2014: 458.
Rhogas grassator ab. thoracicus Hellén, 1927: 24; Shenefelt 1975: 1232 (unavailable name).
Rogas (Rogas) flavipalpis Thomson, 1892: 1672 [examined].
Aleiodes flavipalpis ; Papp 1991a: 86 (as synonym of A. grassator).
Rogas alpinus Thomson, 1892: 1671; Shenefelt 1975: 1217 [examined]. Syn. nov.
Aleiodes alpinus ; Papp 1991a: 90 (as synonym of A. dimidiatus).

##### Type material.

Holotype of *A. grassator*, ♀ (ZMUU), unlabelled. Lectotype of *A. flavipalpis*, ♀ (ZIL), “åre”, “**Sverige**, Ǻreskutan I Jemtland/teste Papp, 1983”, “Lectotypus *Rogas flavipalpis* Thomson, 1899 [sic!], Papp, 1983”, “*Aleiodes grassator* Thb., det. Papp J., 1983”. Lectotype of *A. alpinus*, ♀ (ZIL), “[**Norway**:] Dovre”, “*alpinus* m.”, “Lectotypus *Rogas alpinus* Thoms., 1891, ♀. Papp, 1983”, “*Aleiodes dimidiatus* var. *alpinus* Th., ♀, det. Papp J., 1983”.

##### Additional material.

Austria, British Isles (England: V.C. 70; Scotland: V.C.s 83, 85, 88, 89, 97, 103; Finland, France (both Alps and Pyrenees), Italy, Germany, Norway, Romania, Sweden, Switzerland. Specimens in ZJUH, BZL, MRC, MTMA, NMS, RMNH, SDEI, ZIL, ZMUU, ZSSM. This is essentially a montane grassland species, though occurring at low altitudes in northern Europe.

##### Molecular data.

MRS215 (UK), MRS721 (UK), MRS725 (UK).

##### Biology.

Collected in (April)May–July. Univoltine, overwintering in the mummy. Reared from the noctuid *Cerapteryx graminis* (Linnaeus) (9: K.P. Bland, M.J.W. Cock, M.R. Shaw) and from mummies compatible with that (3), and it may be strictly monophagous. The known host overwinters in the egg stage, and feeds on Poaceae near ground level. The tough dark brown mummy is formed on or below the soil surface and seems spectacularly too large for the adult that will emerge from it (Fig. 384). It is more or less cylindrical, though with a pronounced lateral keel, and well-lined with silk. The cocoon chamber occupies most of the abdominal segments.

##### Diagnosis.

Maximum width of hypoclypeal depression 0.4–0.5 × minimum width of face (Fig. 375); OOL of ♀ ca twice as long as diameter of posterior ocellus (Fig. 376) and distinctly rugose or rugulose; length of 4^th^ antennal segment of ♀ 0.7–0.9 × its width (Fig. 378; in ♂ 0.9–1.0 ×); clypeus thick apically and not protruding anteriorly (Fig. 377); lobes of mesoscutum densely punctate, interspaces largely smooth and shiny; precoxal area coarsely vermiculate-rugose medially; marginal cell of fore wing of ♀ usually ending rather removed from wing apex (Fig. 368); vein 1-CU1 of fore wing 0.5–0.6 × as long as vein 2-CU1 (Fig. 368); hind tarsal claws robust (Fig. 380) and yellowish or brownish bristly setose; hind femur at least apico-dorsally dark brown or black; inner side of hind tibia of ♀ yellowish; pale males have whole frons and stemmaticum yellowish; palpi dark brown or blackish, rarely brown; 3^rd^ metasomal tergite only antero-laterally reddish or yellowish; 4^th^ and 5^th^ tergites black.

##### Description.

Redescribed ♀ (RMNH) from Finland (Sb: Leppävirta). Length of fore wing 4.6 mm, of body 5.7 mm.

***Head.*** Antennal segments of ♀ 39, 4^th^ segment 0.8 × longer than wide (Fig. 378); antenna as long as fore wing, its subapical segments robust (Fig. 379); frons with coarse curved rugae and shiny; OOL 1.9 × diameter of posterior ocellus and rugose; vertex rugose and shiny; face rugose-punctate; clypeus rugose; ventral margin of clypeus rather thick and not protruding forwards (Fig. 377); width of hypoclypeal depression 0.5 × minimum width of face (Fig. 375); length of eye 1.1 × temple in dorsal view (Fig. 376); vertex behind stemmaticum rugose; clypeus below lower level of eyes; length of malar space 0.6 × length of eye in lateral view.

***Mesosoma.*** Mesoscutal lobes moderately punctate, laterally interspaces mainly smooth, medially superficially granulate and rather shiny; precoxal area of mesopleuron coarsely rugose medially and largely smooth posteriorly; remainder of mesopleuron mainly punctate, but dorsally coarsely rugose; scutellum flat, sparsely finely punctate and only anteriorly with lateral carina; propodeum coarsely rugose, medio-longitudinal carina present on anterior half, rounded posteriorly and dorsal part approx. as long as posterior part.

***Wings.*** Fore wing: r 0.3 × 3-SR (Fig. 368); marginal cell ends basad of level of apex of 3-M; 1-CU1 horizontal, 0.5 × 2-CU1; r-m 0.6 × 3-SR; 2^nd^ submarginal cell robust (Fig. 368), 3-SR 1.3 × as long as 2-SR; cu-a vertical, straight; 1-M slightly curved posteriorly; 1-SR similar to 1-M and medium-sized; surroundings of M+CU1, 1-M and 1-CU1 setose. Hind wing: marginal cell linearly widened, its apical width twice width at level of hamuli (Fig. 369); 2-SC+R subquadrate; m-cu short; M+CU:1-M = 27:18; 1r-m 0.7 × 1-M.

***Legs.*** Tarsal claws robust and with only brownish bristly setae (Fig. 380); hind coxa largely densely punctate, but dorsally with some rugae; hind trochantellus robust; length of hind femur and basitarsus 3.1 and 3.9 × their width, respectively; length of inner hind spur 0.45 × hind basitarsus.

***Metasoma.*** First tergite rather flattened, 0.8 × as long as wide apically; 1^st^ and 2^nd^ tergites with medio-longitudinal carina and coarsely longitudinally rugose; medio-basal area of 2^nd^ tergite wide triangular and short (Fig. 372); 2^nd^ suture deep and crenulate; basal half of 3^rd^ tergite finely longitudinally rugose, remainder of metasoma superficially micro-sculptured; 4^th^ tergite without sharp lateral crease; ovipositor sheath wide, with long setae and apically truncate (Fig. 367).

***Colour.*** Orange brown; apical two thirds of antenna, labial palp, patch on hind femur dorso-apically, posterior patch of 2^nd^ tergite and telotarsi, dark brown; head, mesosoma (except side of pronotum postero-dorsally and pair of latero-posterior patches of propodeum), 3^rd^–7^th^ tergites (except antero-lateral corners of 3^rd^ tergite) black; maxillary palp, basal third of antenna, tegulae and remainder of legs rather pale yellowish brown; veins and pterostigma dark brown; wings distinctly infuscate but hind wing less than fore wing.

***Variation.*** Basal third or half of antenna of ♀ pale yellowish brown; head partly and mesosoma anteriorly of ♀ dark orange brown or both entirely black; 3^rd^ tergite longitudinally striate or rugulose basally (sometimes narrowly so), without curved sculptural elements (Fig. 372), except sometimes some weak transverse striae occasionally present at extreme apex. Males are always darker than females; mainly black with legs mainly dark brown or blackish (Fig. 381). Antennal segments: ♀ 36(2), 37(4), 38(6), 39(10), 40(6), 41(1); ♂ 47(1), 48(2), 49(2), 50(3), 51(2), 52(4), 53(1), 60(1). On average males have ca 12 more antennal segments than females. Males have 2^nd^ submarginal cell slightly shorter than of females, temple and face long setose, malar space 0.5–0.7 × length of eye in lateral view, apical tergite type 1, rarely type 2, setae rather dense, fringe not observed and probably absent (Fig. 381). The superficial granulosity of 3^rd^ tergite and mesoscutum may be absent.

##### Distribution.

*Austria, British Isles (England, Scotland), Finland, *France, *Ireland, *Italy, *Germany, Norway, *Romania, Sweden, *Switzerland.

##### New synonymy.

The synonymy of *Rogas alpinus* Thomson, 1892, with *Aleiodes grassator* (Thunberg, 1822) is based on direct comparison of the types listed above.

##### Notes.

Although males of *A. carbonaroides* are generally easily distinguished from *A. grassator* through being black, it is possible that lighter forms occur which would be difficult to recognise. Also, females of *A. carbonaroides* are similar in colour to those of *A. grassator*. Therefore, specimens collected at low altitude away from northern areas that appear, on other characters, to be *A. grassator* might well really be *A. carbonariodes*. See also remarks under *A. carbonarius* and *A. ruficornis*.

**Figures 365–367.**
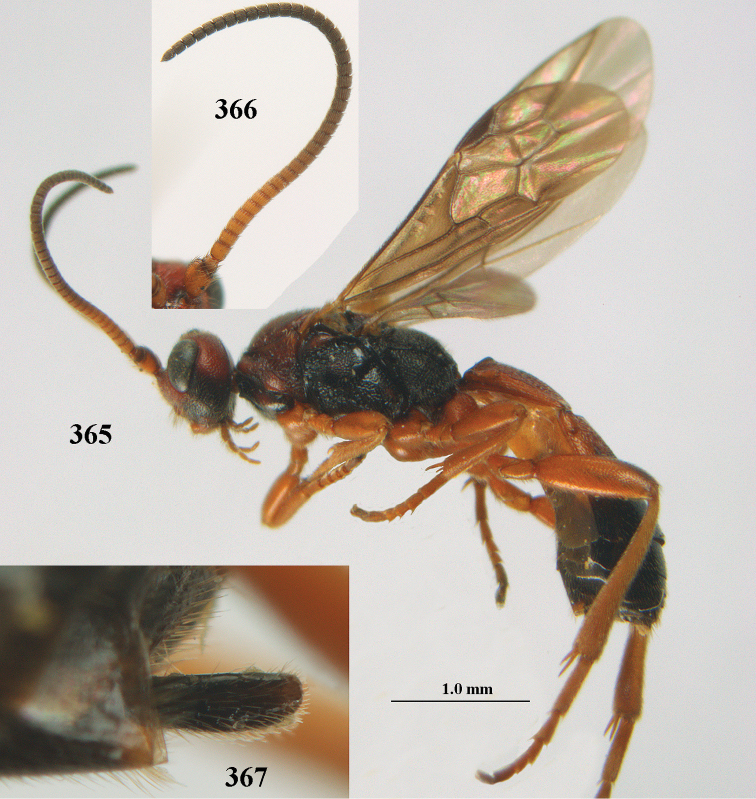
*Aleiodes grassator* (Thunberg), ♀, Scotland, Beinn Ghlas **365** habitus lateral **366** antenna **367** ovipositor sheath lateral.

**Figures 368–380.**
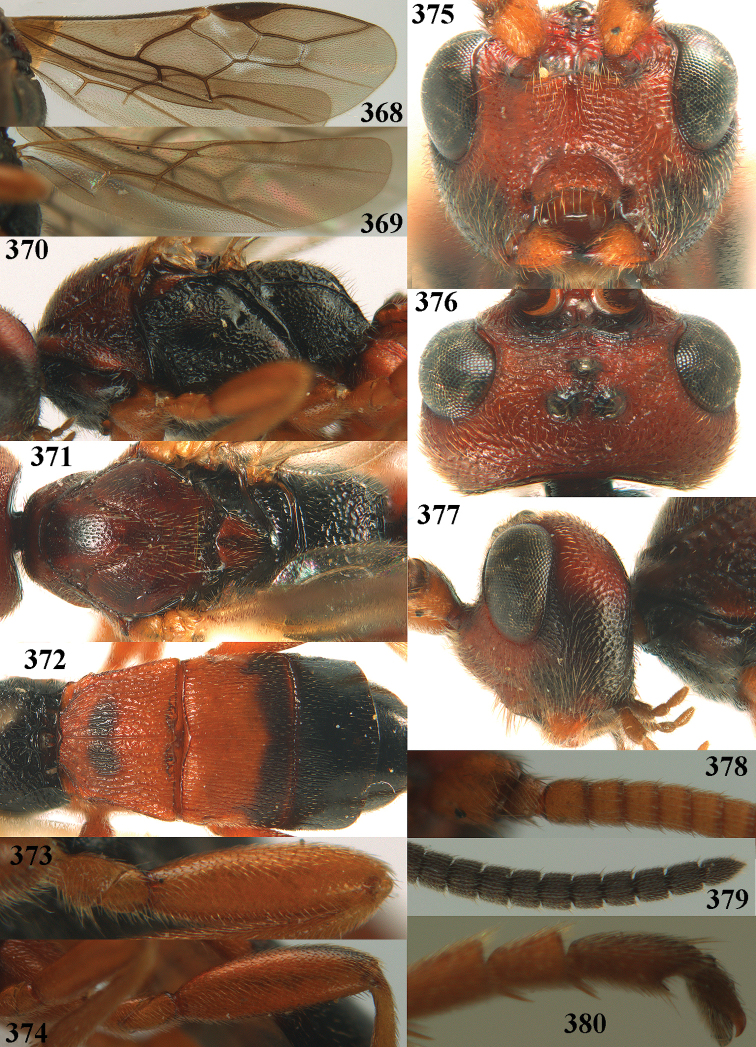
*Aleiodes grassator* (Thunberg), ♀, Scotland, Beinn Ghlas **368** fore wing **369** hind wing **370** mesosoma lateral **371** mesosoma dorsal **372** metasoma dorsal **373** fore femur lateral **374** hind femur lateral **375** head anterior **376** head dorsal **377** head lateral **378** base of antenna **379** apex of antenna **380** outer hind tarsal claw.

**Figures 381–384.**
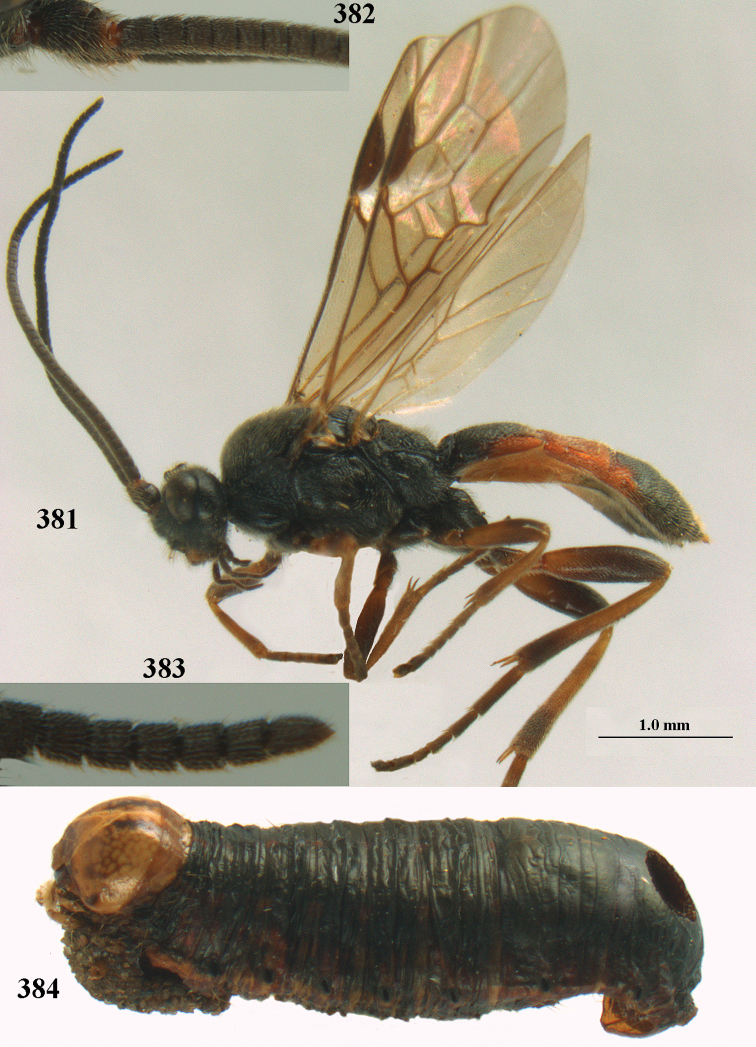
*Aleiodes grassator* (Thunberg), ♂, Scotland, Isle of Coll **381** habitus lateral **382** base of antenna **383** apex of antenna **384** mummy of ?*Cerapteryx graminis* (Linnaeus).

#### Aleiodes hemipterus

Taxon classificationAnimaliaHymenopteraBraconidae

(Marshall, 1897)

72D68C85-7514-5203-B410-C4FFFB93F0DA

Figs 385–389 , 390–399 , 400–408

Rhogas hemipterus Marshall, 1897: 137.
Rogas hemipterus ; Shenefelt 1975: 1233.
Aleiodes hemipterus ; Papp 1990: 90, 2003: 138 (lectotype listed).
Aleiodes (Chelonorhogas) hemipterus ; Belokobylskij and Kula 2012: 35–38.

##### Type material.

Lectotype here designated, ♀ (ZJUH), “**Tunisie**, Sicard”, “Type, H.T.”, “B.M. Type Hym. 3.c.243”, “B.M. Type Hym., *Rhogas hemipterus* Marshall, 1896”, “*hemipterus* Marsh.”, “Marshall coll. 1904-120”. Paralectotype: 1 ♀ (MTMA), “Tunisie”, “*hemipterus* M. coll. Marshall”, “Paratypus *Rhogas hemipterus*Marshall 1897 sp. n. % des Papp J. 1986”, “Hym. Type No 10582 Museum Budapest”, “*Aleiodes* ♀ *hemipterus* Mshl. Det. Papp J. 1991”.

##### Additional material.

1 ♀ + 1 ♂ (MNHN), “[**N. Tunisia**:] Teboursouk”, “*Rhogas hemipterus* Mrsh.”, “Muséum Paris, Coll. J. de Gaulle, 1919” [figured specimens]; 1 ♂ [but metasoma missing] (ZJUH) “Rabat, Maroc [= **Morocco**], coll. Thery”.

##### Molecular data.

None.

**Biology.** Unknown. The specimens seen do not have phenological data, and we have not seen reared material. As the female is brachypterous it is likely that the host will be found near the ground.

##### Diagnosis.

Maximum width of hypoclypeal depression approx. 0.6 × minimum width of face (Fig. 396); OOL of ♀ 1.2–1.3 × as long as diameter of posterior ocellus, of ♂ 0.9 × (Figs 397, 408) and finely reticulate-rugose; stemmaticum protruding dorsally; antennal segments of ♀ 46–50 (of ♂ unknown), penultimate segments approx. as long as wide and antenna 0.8 × as long as body; length of malar space 0.3–0.4 × height of eye; mesoscutal lobes densely rugose or rugulose and rather matt, middle lobe with medio-longitudinal ridge or carina, of ♀ surrounded by shallow grooves (Fig. 392); propodeum angulate posteriorly (Fig. 392); ♀ brachypterous and ♂ macropterous; marginal cell of hind wing of ♀ hardly widened (Fig. 390) and of ♂ distinctly widened apically (Fig. 403); hind tarsal claws with rather conspicuous pale brown pecten (Fig. 399); 1^st^–3^rd^ metasomal tergites very densely and finely longitudinally rugose; 1^st^ tergite of ♀ 1.0–1.1 × its apical width, of ♂ approx. 1.4 ×; 2^nd^ metasomal suture of ♀ hardly impressed but densely costate, of ♂ medium-sized.

##### Description.

Lectotype, ♀, length of hind wing 1.7 mm (fore wing missing, but in other specimens ca one-third longer than hind wing and 2.2 mm, brachypterous), of body 7.8 mm.

***Head.*** Antenna incomplete, segments robust; frons largely striate-rugose (but transversely costate in figured ♀); OOL 1.2 × diameter of posterior ocellus, (as vertex) rather finely and densely reticulate-rugose and rather dull; clypeus rugose; ventral margin of clypeus rather thick ventrally and rather forward protruding (Fig. 398); width of hypoclypeal depression 0.6 × minimum width of face and long (Fig. 396); length of eye 1.2 × temple in dorsal view (Fig. 397); vertex behind stemmaticum rather coarsely reticulate-rugose; clypeus near lower level of eyes; length of malar space 0.35 × length of eye in lateral view.

***Mesosoma.*** Antescutal depression distinct; mesoscutal lobes coarsely rugose-punctate (but superficial in figured ♀) and rather matt, middle lobe of pair of submedian grooves (Fig. 392); nearly entire mesopleuron (except minute smooth speculum) densely and coarsely reticulate-rugose; scutellum coarsely rugose and without lateral carinae; propodeum coarsely vermiculate-rugose, dorsal face long and rectangularly angulate postero-laterally (Fig. 392).

***Wings.*** Fore wing brachypterous, hardly surpassing propodeum (Marshall, 1897): (of ♀ from Tunisia r 0.2 × 3-SR; 1-CU1 distinctly widened and oblique, 0.4 × 2-CU1; r-m 0.8 × 3-SR; 2^nd^ submarginal cell medium-sized (Fig. 390); cu-a short, vertical, straight; 1-M straight posteriorly; 1-SR widened; 1^st^ subdiscal cell open apically and posteriorly; surroundings of M+CU1, 1-M and 1-CU1 setose; 2m-cu present as curved and only pigmented vein). Hind wing brachypterous: marginal cell reduced, sinuate and apically narrowed (Fig. 390); 2-SC+R quadrate and widened; m-cu absent; M+CU:1-M = 27:13; 1r-m 0.6 × 1-M.

***Legs.*** Tarsal claws with rather conspicuous pale brown pecten, remaining far from apical tooth and much shorter (Fig. 399); hind coxa finely and densely reticulate-rugose; hind trochantellus robust; length of hind femur and basitarsus 3.6 and 6.0 × their width, respectively; length of inner hind spur 0.4 × hind basitarsus.

***Metasoma.*** First tergite evenly convex, as long as wide apically; 1^st^–3^rd^ tergites regularly finely and very densely longitudinally rugose, rather matt and medio-longitudinal carina rather weak; medio-basal area of 2^nd^ tergite triangular and short (Fig. 393); 2^nd^ suture shallow; 4^th^ and subsequent tergites superficially punctulate; 4^th^ and apical half of 3^rd^ tergite without sharp lateral crease; ovipositor sheath wide, with medium-sized setae and apically truncate (Fig. 389).

***Colour.*** Brown; stemmaticum and ovipositor sheath black; frons, vertex medially, occiput, femora, propodeum, 1^st^ and 2^nd^ tergites somewhat infuscate; wing membrane subhyaline.

***Variation.*** Length of body 7.8–8.8 mm. Antennal segments: ♀ 46(1), 50(1); ♂ unknown. Male is normally winged (vein 3-SR of fore wing 1.5 × vein 2-SR, vein r 0.3 × 3-SR, vein cu-a oblique, vein 1-CU1 narrow and 0.3 × vein 2-CU1) and has marginal cell of hind wing 2.2 × wider than width at level of hamuli (with vein m-cu present anteriorly, 2-SC+R quadrate and M+CU:1-M:1r-m = 40:30:26). Apical metasomal segments of ♂ type 1 and sparsely setose.

##### Distribution.

Morocco, Tunisia.

##### Notes.

Marshall (1897) based his description on three females from Tunisia. Papp (2003) listed a female in ZJUH as lectotype, but this was not accepted as a designation by Belokobylskij & Kula (2012). Therefore, the redescribed female above is here designated formally as lectotype and is the same specimen intended to become lectotype by Papp (2003).

**Figures 385–389.**
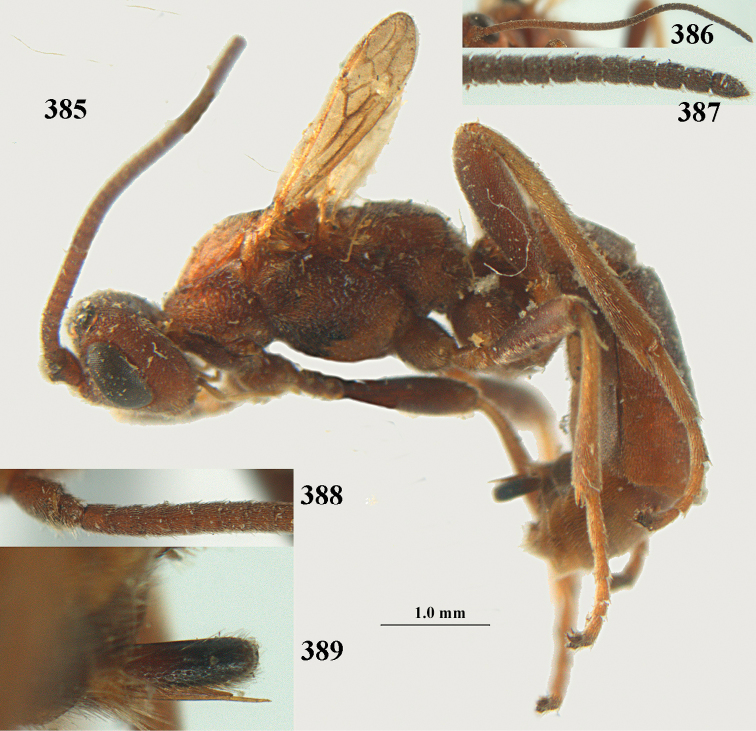
*Aleiodes hemipterus* (Marshall), ♀, Tunisia, Teboursouk **385** habitus lateral **386** antenna **387** apex of antenna **388** base of antenna **389** ovipositor sheath lateral.

**Figures 390–399.**
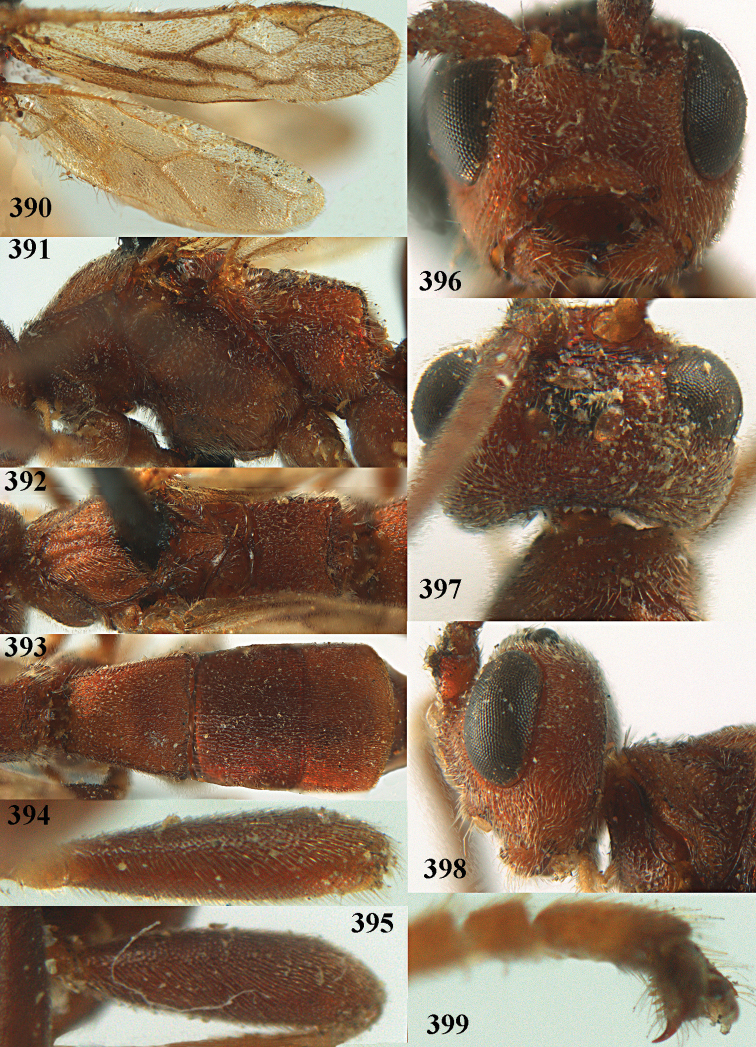
*Aleiodes hemipterus* (Marshall), ♀, Tunisia, Teboursouk **390** wings **391** mesosoma lateral **392** mesosoma dorsal **393** metasoma dorsal **394** fore femur lateral **395** hind femur lateral **396** head anterior **397** head dorsal **398** head lateral **399** outer hind tarsal claw.

**Figures 400–408.**
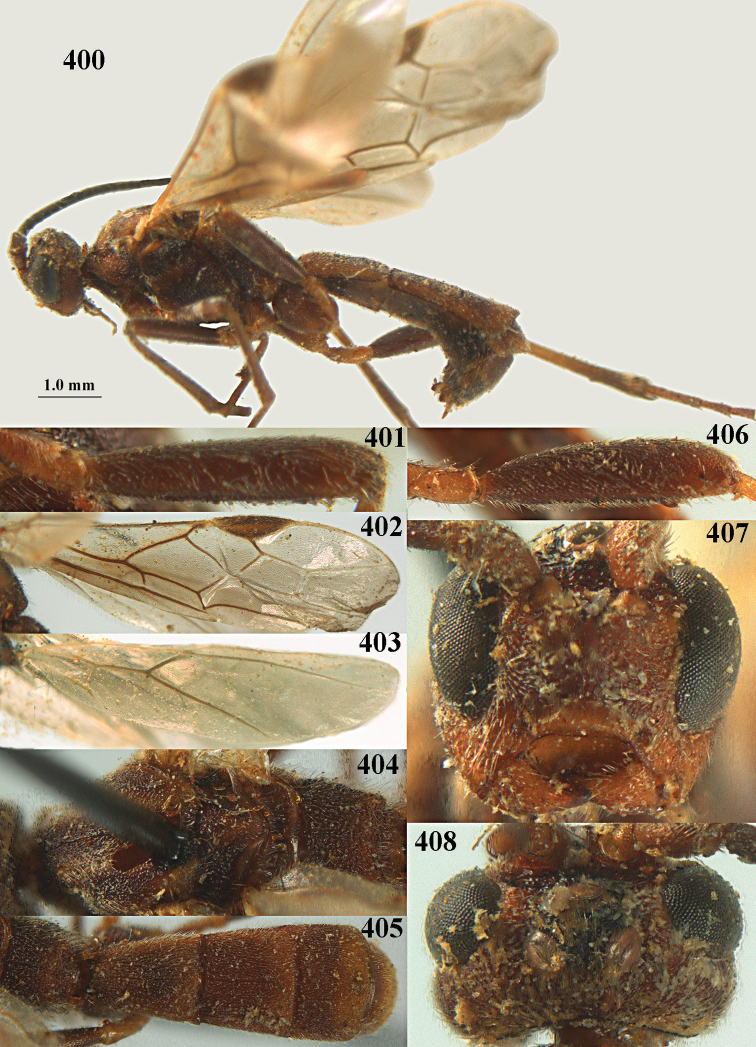
*Aleiodes hemipterus* (Marshall), ♂, Tunisia, Teboursouk **400** habitus lateral **401** fore femur lateral **402** fore wing **403** hind wing **404** mesosoma dorsal **405** metasoma dorsal **406** hind femur lateral **407** head anterior **408** head dorsal.

#### Aleiodes hirtus

Taxon classificationAnimaliaHymenopteraBraconidae

(Thomson, 1892)

5B1094D6-D8D0-5EC9-9D98-A133001D9CD2

Figs 409–410 , 411–424 , 425–427

Rogas hirtus Thomson, 1892: 1672; Shenefelt 1975: 1233.
Aleiodes (Neorhogas) hirtus ; Papp 1985a: 153, 155, 161 (lectotype designation and as synonym of A. pallidicornis), 1991a: 75 (id.).
Aleiodes hirtus ; Papp 2005: 177 (as synonym of A. pallidicornis).
Rhogas hirtus ab. coloratus Hellén, 1927: 23; Shenefelt 1975: 1233; Papp 2005: 177 (as synonym of A. pallidicornis) (unavailable name; not Rogas coloratus Motschulsky, 1863).

##### Type material.

Lectotype of *A. hirtus*, ♂ (ZIL), “[**Norway**], Norl.”, “*hirtus* m.”, “Funne I Norrland, teste Papp J., 1983”, “Lectotypus”, “*Rogas hirtus* Thms. 1891, ♂, Papp J., 1983”, “*Aleiodes pallidicornis* HS ♂, det. Papp J., 1983”.

##### Additional material.

Austria, Belgium, British Isles (England: V.C.s 26, 29, 32, 62; Scotland: V.C. 78; Ireland: V.C. H12), Bulgaria, Czech Republic, Finland, France, Germany, Hungary, Netherlands (DR: Borger), Norway, Romania, Russia, Serbia, Slovakia, Switzerland, Ukraine, [? Mongolia]. Specimens in ZJUH, BZL, CMIM, CNC, FMNH, MRC, MSC, MTMA, NMS, RMNH, SDEI, UNS, USNM, UWIM, ZIL, ZSSM.

##### Molecular data.

MRS619 (UK), MRS882 (Romania), MRS883 (Romania).

##### Biology.

Unknown. Collected in June–August, presumably univoltine but the mode of overwintering is unclear. Most British sites are more or less damp and calcareous grasslands, approaching fens. We have not seen reared material, but the clypeus suggests that the mummy will form in the soil.

##### Diagnosis.

Maximum width of hypoclypeal depression 0.5–0.6 × minimum width of face (Fig. 419); OOL of ♀ approx. 1.3 × as long as diameter of posterior ocellus (Fig. 420; in ♂ approx.1.6 ×) and punctate-rugose; ventral margin of clypeus rather thick but rather strongly protruding forwards (Fig. 421; stronger in in ♂: Fig. 426); mesoscutal lobes largely smooth, only indistinctly punctulate and shiny; precoxal area finely punctate and often with some rugulae (Fig. 413); vein 1-CU1 0.3–0.6 × vein 2-CU1 of fore wing (Fig. 411); hind tarsal claws with rather conspicuous brownish pecten (Fig. 424); length of inner spur of hind tibia 0.5–0.7 × hind basitarsus; palpi dark brown; basal half of metasoma at least partly reddish or orange and 1^st^ tergite rather coarsely sculptured; setae of body of ♂ (but of ♀ mainly its head) conspicuous and dense (Fig. 426); hind coxa black; hind femur largely or completely reddish or brownish; basal half of hind tibia usually (pale) yellowish or yellowish brown, but sometimes uniformly reddish and of ♂ ivory. In the past this species has been frequently misidentified as “*Rogas dimidiator*” or “*Rogas gasterator*”.

##### Description.

Redescribed ♂ (RMNH) from Germany (Graswang). Length of fore wing 6.2 mm, of body 8.0 mm. Entire body with long whitish setae.

***Head.*** Antennal segments of ♂ 60, length of antenna 1.3 × fore wing, its subapical segments somewhat longer than wide; frons medially largely smooth, laterally with some fine curved rugae; OOL 1.6 × diameter of posterior ocellus, and punctate-rugose, POL approx. half as long as diameter of ocellus; vertex spaced rugose, shiny; clypeus punctate; ventral margin of clypeus rather thick but distinctly protruding forwards (Fig. 421); width of hypoclypeal depression 0.55 × minimum width of face (Fig. 419); length of eye 1.1 × temple in dorsal view (Fig. 420), temples conspicuously setose (Figs 419, 426); vertex behind stemmaticum rugose; clypeus near lower level of eyes; length of malar space 0.3 × length of eye in lateral view.

***Mesosoma.*** Pronotum rugose and anteriorly oblique, without antescutal depression; mesoscutal lobes large smooth and shiny, only indistinctly punctulate and densely setose; precoxal area of mesopleuron punctulate and medially with some superficial rugulae; remainder of mesopleuron sparsely punctate; scutellum sparsely punctate and largely smooth, posteriorly with lateral rugae; propodeum rather convex and coarsely rugose, its medio-longitudinal carina only in anterior half of propodeum.

***Wings.*** Fore wing: r 0.3 × 3-SR (Fig. 411); 1-CU1 horizontal, 0.45 × 2-CU1; r-m 0.7 × 3-SR and as long as 2-SR; 2^nd^ submarginal cell medium-sized (Fig. 411); cu-a vertical, straight; 1-M straight posteriorly; 1-SR wide; surroundings of M+CU1, 1-M and 1-CU1 largely glabrous. Hind wing: marginal cell gradually widened (but less so basally: Fig. 412), its apical width 2.5 × width at level of hamuli; 2-SC+R short longitudinal; m-cu narrowly indicated; M+CU:1-M = 9:7; 1r-m 0.7 × 1-M.

***Legs.*** Tarsal claws with rather conspicuous and medium-sized brownish pecten (Fig. 424); hind coxa pimply punctate and shiny; hind trochantellus robust; length of hind femur and basitarsus 3.8 and 6.0 × their width, respectively; length of inner hind spur 0.65 × hind basitarsus (0.6 × in ♀).

***Metasoma.*** First tergite evenly convex, approx. as long as wide apically; 1^st^ and 2^nd^ tergites with medio-longitudinal carina and densely longitudinally rugose, but posterior quarter of 2^nd^ tergite irregularly rugose and no median carina; medio-basal area of 2^nd^ tergite minute (Fig. 415); 2^nd^ suture deep and moderately crenulate; basal half of 3^rd^ tergite finely rugose, remainder of metasoma largely smooth; 4^th^ and apical half of 3^rd^ tergite without sharp lateral crease; 4^th^ – 6^th^ tergites with long setae and flat.

***Colour.*** Black; legs (except black coxae, trochanters and trochantelli, 1^st^ and 2^nd^ metasomal tergites (but base of 1^st^ tergite partly infuscate) and base of 3^rd^ tergite orange brown; vaguely near base of femora, telotarsi, apex of hind femur, apical half of hind tibia, hind tarsus largely black or blackish; basal half of hind tibia pale yellow; palpi, tegulae, veins and pterostigma dark brown; wing membrane slightly infuscate.

***Variation.*** Hind femur varies from apically black to entirely orange. Propodeum can be partly orangish in posterior part. Usually both sexes have hind trochanter (often also trochantellus) more or less infuscate and darker than the orange part of the hind femur, but this is scarcely evident in a series from S. Russia (MRC, NMS). Hind coxa varies from orange to black. Female is similar to the more distinctive male but is less conspicuously setose (Figs 419–421) and its ovipositor sheath is wide, with long setae and apically truncate (Fig. 410). Precoxal sulcus smooth to superficially rugulose medially. A female and a male from Romania (NMS) are slightly different from British ones; ocelli approx. 1/5 larger and frons coarsely rugose posteriorly. This appears to be reflected by a small divergence in CO1 (2.75 %), but for the moment we treat them as belonging to *A. hirtus*. Antennal segments of ♀ 54(1), 55(2), 56(6), 57(6), 59(2), 60(2), 61(1), of ♂ 56(1), 58(1), 59(3), 60(3), 61(1). Apical tergites of male type (1–)2, setae rather sparse but long and glabrous stripe consequently not always evident and fringe present but poorly differentiated. A female from Mongolia (BZL) with completely black hind femur and base of hind tibia pale yellowish and with dark basal ring may be another very similar species.

##### Distribution.

*Austria, *Belgium, *British Isles (England, Scotland, Ireland), *Bulgaria, *Czech Republic, Finland, *France, *Germany, *Hungary, *Netherlands, Norway, *Romania, *Russia, *Serbia, *Slovakia, *Switzerland, *Ukraine.

**Figures 409, 410.**
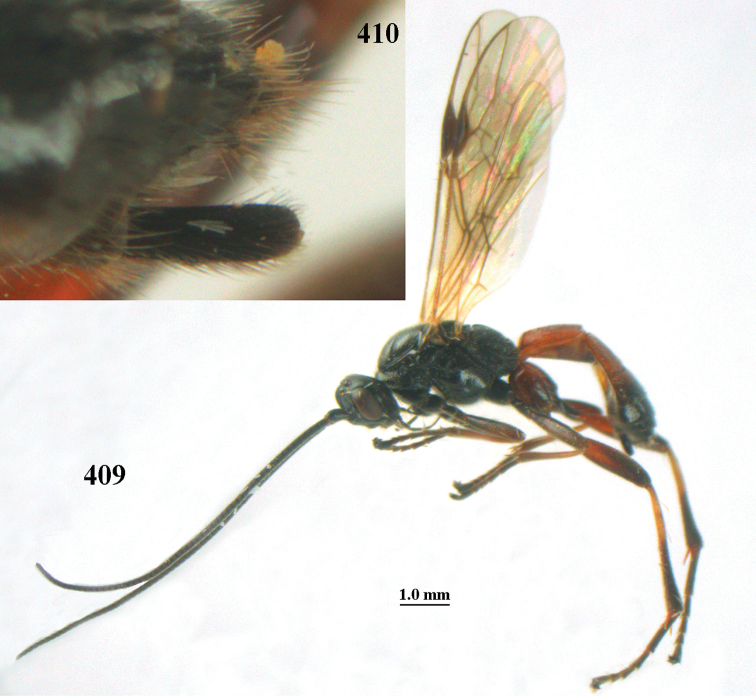
*Aleiodes hirtus* (Thomson), ♀, Scotland, Peebles **409** habitus lateral **410** ovipositor sheath lateral.

**Figures 411–424.**
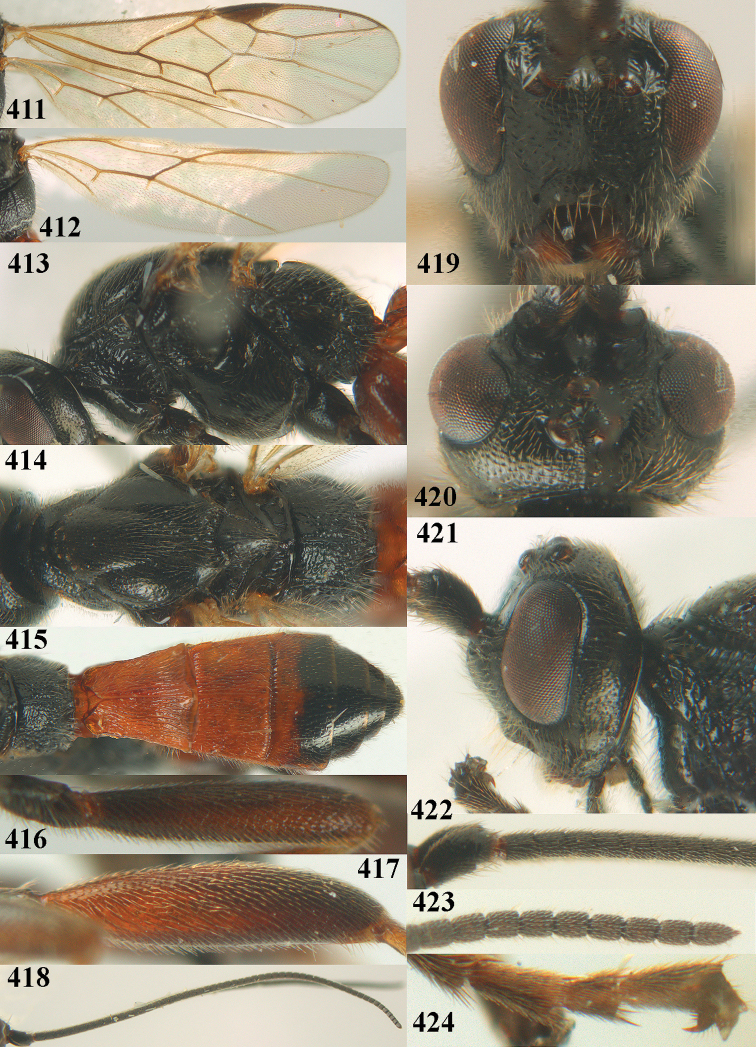
*Aleiodes hirtus* (Thomson), ♀, Scotland, Peebles **411** fore wing **412** hind wing **413** mesosoma lateral **414** mesosoma dorsal **415** metasoma dorsal **416** fore femur lateral **417** hind femur lateral **418** antenna **419** head anterior **420** head dorsal **421** head lateral **422** base of antenna **423** apex of antenna **424** inner hind tarsal claw.

**Figures 425–427.**
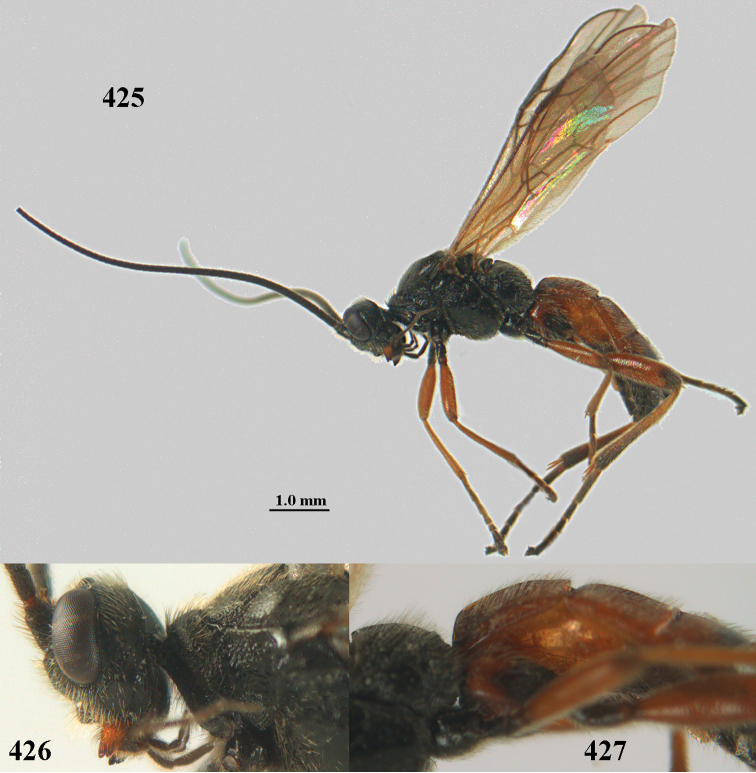
*Aleiodes hirtus* (Thomson), ♂, England, Chippenham Fen **425** habitus lateral **426** head and anterior part of mesosoma lateral **427** propodeum and 1^st^ –3^rd^ metasomal segments lateral.

#### Aleiodes improvisus

Taxon classificationAnimaliaHymenopteraBraconidae

van Achterberg & Shaw sp. nov.

3EAD392B-2586-5259-9C5B-B2EEDA721EB6

http://zoobank.org/BA464FC8-D95C-4CF8-A27F-788BB7D2D3C4

Figs 428–431 , 432–444 , 445–452

##### Type material.

Holotype, ♀ (ZJUH), “**Austria**: Tirol, Obergurgl, 2000 m, vii.[19]81, Day & Fitton”. Paratypes: 1 ♂ (NMS), “**Switzerland**: Valais, Aletschwald, 6000–7000 ft, 7–17.vi.1959, J.E. & R.B. Benson”; 1 ♂ (ZJUH), “Switzerland: Valais, J.E. & R.B. Benson, B.M. 1935-581”, “Arolla, 6500 ft, 12.vi.1935”; 3 ♂ (ZJUH, NMS), “Austria: Tirol Vent., 1860 m, vii.1981, Fitton & Day”; 1 ♂ (ZJUH), “Austria: Tirol Vent (Winterstallen), 1750 m. vii.1981, Fitton & Day”.

##### Molecular data.

None.

##### Biology.

Unknown. Collected above the tree line in the Alps in June–July, and presumably univoltine.

##### Diagnosis.

Maximum width of hypoclypeal depression 0.4–0.5 × minimum width of face (Fig. 439); OOL of ♀ 1.8 × as long as diameter of posterior ocellus and densely rugose (Fig. 440); antenna of ♀ as long as fore wing; clypeus thick apically and not protruding anteriorly in lateral view (Fig. 441); lobes of mesoscutum densely punctate, interspaces largely finely coriaceous and with satin sheen; precoxal area coarsely vermiculate-rugose medially; marginal cell of fore wing of ♀ ending rather removed from wing apex (Fig. 432); vein 1-CU1 of fore wing 0.3–0.6 × as long as vein 2-CU1; fore femur subparallel-sided (Fig. 437); hind tarsal claws yellowish or brownish bristly setose and with few dark brown or brown pectinal teeth submedially (Fig. 442); 3^rd^ tergite longitudinally rugulose basally, without curved sculptural elements (Fig. 436); head of ♀ black; inner side of hind tibia of ♀ dark brown ventrally; palpi dark brown or blackish; hind trochanter and trochantellus largely dark brown; 2^nd^ metasomal tergite of both sexes orange or dark reddish brown; 4^th^ and 5^th^ tergites black.

##### Description.

Holotype, ♀, length of fore wing 5.5 mm, of body 7.7 mm.

***Head.*** Antennal segments of ♀ 41, antenna as long as fore wing, its subbasal and subapical segments rather robust (Fig. 444); frons with curved rugae; OOL 1.8 × diameter of posterior ocellus, densely rugose and with satin sheen; vertex densely rugose (also behind stemmaticum), with satin sheen; clypeus transversely rugulose; ventral margin of clypeus thick and not protruding forwards (Fig. 441); width of hypoclypeal depression 0.4 × minimum width of face (Fig. 439); eye as long as temple in dorsal view (Fig. 440); clypeus below lower level of eyes; length of malar space 0.55 × length of eye in lateral view; temple striate near eye, and remainder rugose; head with long setae.

***Mesosoma.*** Mesoscutal lobes densely punctate, interspaces largely finely coriaceous and with satin sheen; precoxal area of mesopleuron coarsely vermiculate-rugose medially, but posteriorly rugose; mesopleuron remotely punctate and shiny medially; metapleuron densely rugose and rather dull; scutellum largely smooth (except for spaced punctures), shiny and nearly flat, with lateral carina; propodeum coarsely rugose but antero-laterally rugulose, laterally dorsal face longer than posterior one, somewhat angulate laterally but without tubercles, and with complete medio-longitudinal carina.

***Wings.*** Fore wing: r 0.4 × 3-SR; marginal cell fairly short (Fig. 432); 1-CU1 horizontal, 0.3 × 2-CU1; r-m 0.8 × 3-SR; 2^nd^ submarginal cell medium-sized (Fig. 432); cu-a vertical, straight; 1-M slightly curved posteriorly; 1-SR wider than 1-M; surroundings of M+CU1, 1-M and 1-CU1 largely setose. Hind wing: marginal cell linearly widened, its apical width 2.1 × width at level of hamuli (Fig. 433); 2-SC+R quadrate; m-cu narrowly pigmented; M+CU:1-M = 30:21; 1r-m 0.7 × 1-M.

***Legs.*** Tarsal claws mainly setose but submedially with four rather short and dark brown pectinal teeth (Fig. 442); fore femur largely parallel-sided and rather slender (Fig. 437); hind coxa punctate and shiny, but dorsally mainly rugose; hind trochantellus rather robust; length of hind femur and basitarsus 3.3 and 5.7 × their width, respectively; length of inner hind spur 0.4 × hind basitarsus.

***Metasoma.*** First tergite distinctly convex medially, its length 0.8 × apical width, robust and irregularly longitudinally rugose as 2^nd^ tergite; both tergites with medio-longitudinal carina; medio-basal area of 2^nd^ tergite triangular and small (Fig. 436); 2^nd^ suture moderately deep and crenulate; basal half of 3^rd^ tergite largely longitudinally striate, remainder of metasoma superficially micro-sculptured or smooth; 4^th^ and apical half of 3^rd^ tergite without sharp lateral crease; ovipositor sheath wide medially, with long setae and apically truncate (Fig. 429).

***Colour.*** Black; antenna (but only scapus partly yellowish), right fore coxa, trochanter, trochantellus, and femur (but left all yellowish brown except dark base of coxa and infuscated apex of femur), middle femur dorso-apically, middle coxa basally, hind trochanter, trochantellus and femur (but dorso-basally yellowish and left femur also ventrally), apical third of hind tibia (but left tibia yellowish ventrally), tegulae, pterostigma, veins largely, and metasoma ventrally largely dark brown; dorsal part of scutellum, 1^st^ tergite laterally and narrowly medially and posteriorly, 2^nd^ tergite and antero-laterally 3^rd^ tergite orange brown; right fore tibia (except basally and left one yellowish brown) and tarsi more or less infuscate (but left fore tarsus only telotarsus dark brown); fore wing membrane somewhat infuscate, but hind wing nearly subhyaline.

***Variation.*** Eye of ♀ as long as temple in dorsal view (of ♂ 1.0–1.4 ×); length of malar space 0.5–0.6 × length of eye in lateral view; palpi black or largely dark brown; 1-CU1 0.3–0.6 × 2-CU1; length of fore wing 4.0–6.5 mm. Antennal segments: ♀ 41(1); ♂ 44(2), 49(2), 51(2). Male often has much darker legs (largely dark brown with coxae black as right legs of holotype, but legs are more extensively orange, including basal half of hind femur, in two paratypes) than female and scutellum black; metasoma similarly sculptured and coloured or also basal half of 3^rd^ tergite orange brown or 1^st^ tergite only posteriorly orange or only 2^nd^ and 3^rd^ tergites (except posteriorly) dark reddish brown; in the largest male paratype (Winterstallen) traces of inwardly curved sculpture are discernible posteriorly on the almost completely longitudinally rugose 3^rd^ tergite; marginal cell of fore wing similar to ♀, with apical tergites type 1 and fringe not observed (Figs 450, 452).

##### Distribution.

Austria, Switzerland.

##### Etymology.

*Improvisus* is Latin for unexpected, unforeseen, because at first sight the specimens were expected to belong to *A. gasterator* or *A. ruficornis*.

##### Notes.

As suggested by its name this species can be easily confused with *A. gasterator* or *A. ruficornis*. It differs from *A. gasterator* mainly by being darker (subbasal antennal segments of ♀, hind trochanter and trochantellus, inner and dorsal side of hind tibia, parastigma) and somewhat higher number of antennal segments of ♀ (41 *vs* 29–39). *Aleiodes ruficornis* has an inflated fore femur (hardly or not inflated in *A. improvisus*), antenna of ♀ medium-sized (1.0–1.2 × fore wing *vs* 0.8–0.9 ×) and head of ♀ at least partly reddish brown.

**Figures 428–431.**
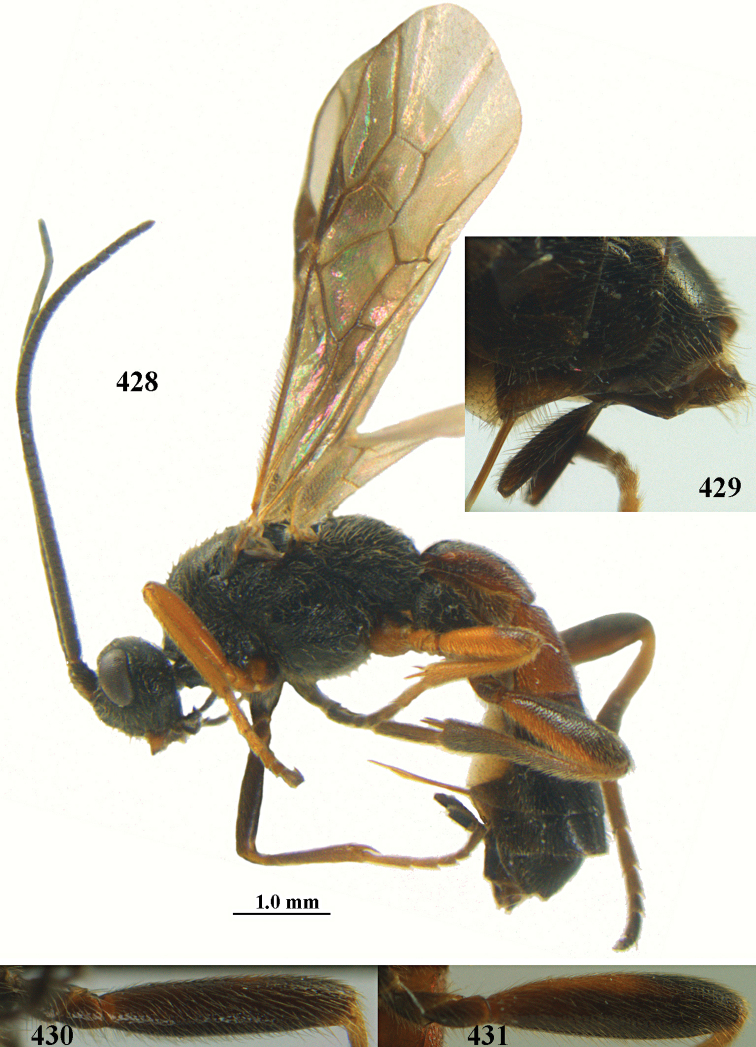
*Aleiodes improvisus* sp. nov., ♀, holotype **428** habitus lateral **429** ovipositor sheath lateral **430** right fore femur lateral **431** right hind femur lateral.

**Figures 432–444.**
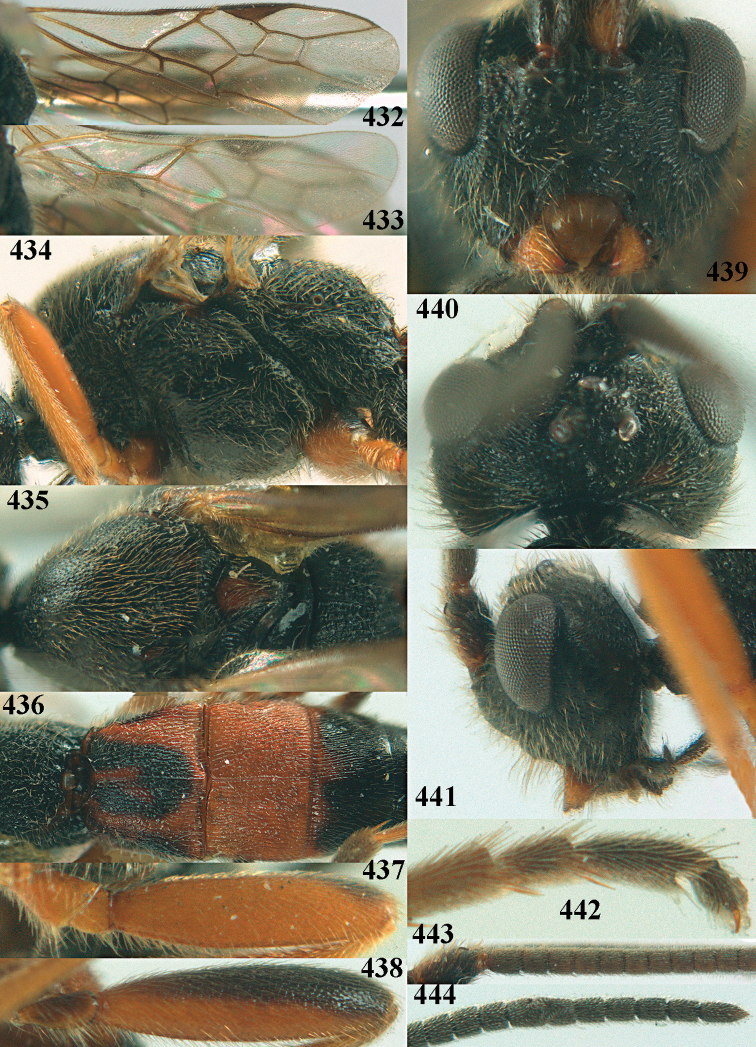
*Aleiodes improvisus* sp. nov., ♀, holotype **432** fore wing **433** hind wing **434** mesosoma lateral **435** mesosoma dorsal **436** propodeum and 1^st^ –3^rd^ metasomal tergites dorsal **437** left fore femur lateral **438** left hind femur lateral **439** head anterior **440** head dorsal **441** head lateral **442** inner hind tarsal claw **443** base of antenna **444** apex of antenna.

**Figures 445–452.**
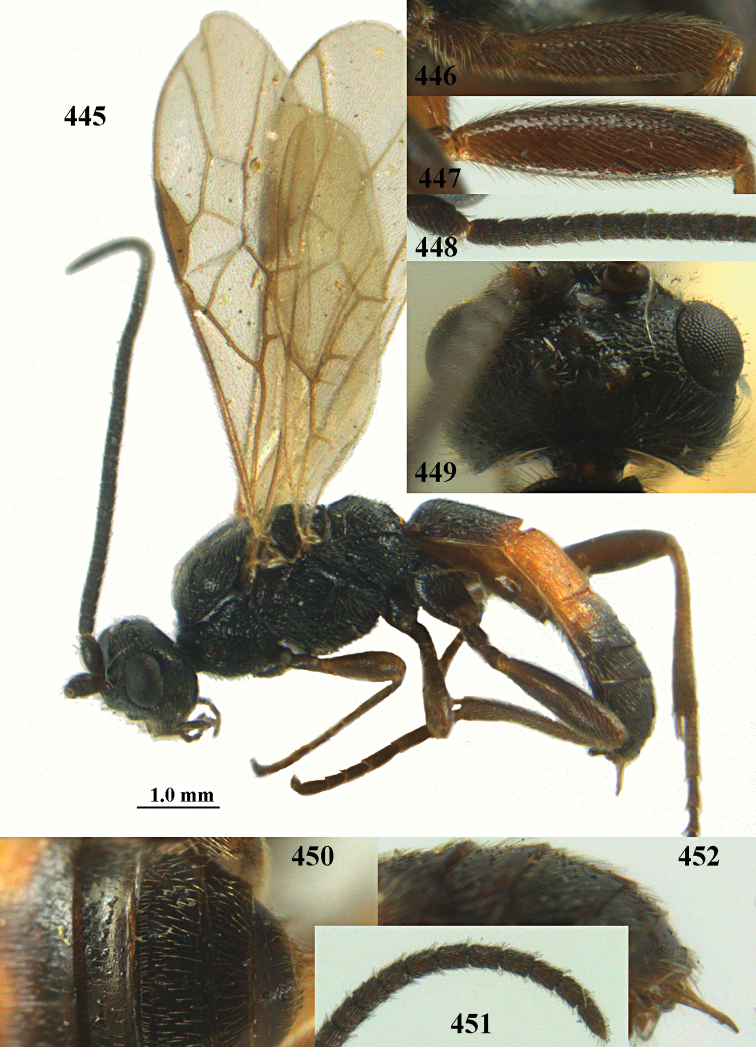
*Aleiodes improvisus* sp. nov., ♂, paratype, Switzerland (Arolla) **445** habitus lateral **446** fore femur lateral **447** hind femur lateral **448** base of antenna **449** head dorsal **450** 4^th^–7^th^ metasomal tergites dorsal **451** apex of antenna **452** 4^th^–7^th^ metasomal tergites lateral.

#### Aleiodes krulikowskii

Taxon classificationAnimaliaHymenopteraBraconidae

(Kokujev, 1898)

795E8253-12E4-5D60-9CBB-CA9878D5443B

Figs 453–454 , 455–466

Rhogas (Rhogas) krulikowskii Kokujev, 1898: 302; Telenga 1941: 157–158.
Rogas krulikowskii ; Papp 1971: 360; Shenefelt 1975: 1235.
Rogas (Rogas) krulikovskii ; Tobias 1976: 83.
Rogas (Rogas) krulikowskii ; Tobias 1986: 77 (transl. 125) (lectotype designation).
Aleiodes (Neorhogas) krulikowskii ; Papp 1985a: 153, 1991a: 84; Belokobylskij 1996: 9.
Aleiodes (Chelonorhogas) krulikowskii ; Belokobylskij 1996: 2000: 31; Chen and He, 1997: 40; He et al. 2000: 665.
Aleiodes krulikowskii ; Fortier and Shaw 1999: 230; Papp 2004: 153.
Rhogas csikii Szépligeti, 1901 150.
Rogas csikii ; Shenefelt 1975: 1223.
Aleiodes csikii ; Papp 1991: 84 (as synonym of A. jaroslawensis); 2004: 216 (as synonym of A. krulikowskii).

##### Type material.

Lectotype of *A. krulikowskii*, ♀ (ZISP), “[**Russia**:] Kirovsk ts., Malmyzh, L.K. Krulikovsk, N. 1906”, “*Rh. Krulikowskii* Kokw., No. 1906”, “Lectotypus *Rogas krulikowskii* Kok., design. [V.I.] Tobias, 1980”. Holotype of *A. csikii*, ♀ (MTMA), “[Russia:] Siberia, Minusinsk, 30.vii.[18]98, Exp. Zichy, leg. Csiki”, “Holotypus ♀ *Rhogas csikii* sp. n. Szépl., 1901/ des. Papp J, 1967”, “Hym. Typ. No. 403, Museum Budsapest”, “*Aleiodes* ♀ *krulikowskii* Kok., det. Papp J., 1983”.

##### Additional material.

1 ♀ (MTMA), “[**Romania**: N Siebenburgen,] Radnai havas, Páváy V.F/ 26.vii.1906, 1400”; 1 ♂ (MTMA), “[**Hungary**:] Jaruer, 20.vi.”, “*Rhogas carbonarius* Gir. var. det. Szépligeti”; 1 ♀ (MTMA), “[Russia:] Ussuri, Kasakewitsch, 1907, Korb”; 1 ♀ (MTMA), “**Mongolia**: Suchebaator aimak, 44 km SSW von Baruun urt, 1050 m, Exp. Dr. Z. Kaszab, 1965, nr. 349, 2–3.viii.1965”; 1 ♂ (MTMA), “Mongolia: Cojbalsan aimak, Somon Chalchingol, 600 m, Exp. Dr. Z. Kaszab, 1965, nr. 409, 13.viii.1965”.

##### Molecular data.

None.

##### Biology.

Unknown. The collection dates (June–August) suggest that it is univoltine, but there is nothing to suggest how it overwinters.

##### Diagnosis.

**Figures 453, 454.**
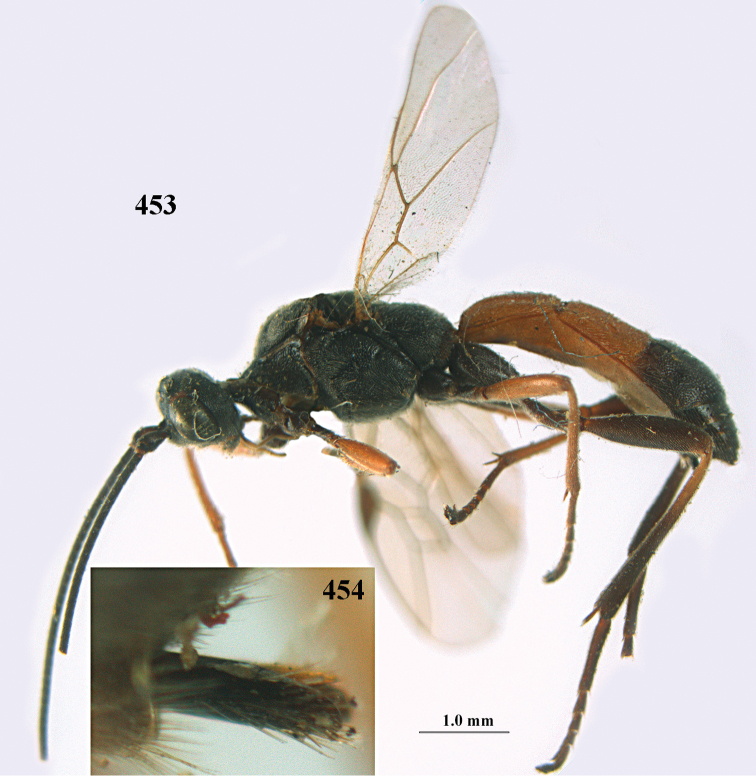
*Aleiodes krulikowskii* (Kokujev), ♀, lectotype **453** habitus lateral **454** ovipositor sheath lateral.

Maximum width of hypoclypeal depression 0.7–0.8 × minimum width of face (Fig. 463); OOL of ♀ approx. 1.3 × longer than diameter of posterior ocellus and coarsely rugose (Fig. 464); ventral margin of anterior part of clypeus thin, clypeus approx. 5 × wider than long medially (Fig. 463) and more or less protruding in lateral view (Fig. 465); head robust in anterior view (Fig. 463); lateral mesoscutal lobes densely and coarsely punctate, with interspaces narrower than punctures but interspaces becoming wider posteriorly, middle lobe coriaceous, but punctate near narrow and distinctly impressed notauli; mesopleuron densely and coarsely punctate, interspaces approx. equal to diameter of punctures or narrower; vein 1-CU1 of fore wing 0.8 × vein 2-CU1, widened and 1.1 × longer than vein m-cu; hind tarsal claws robust and with inconspicuous fine subbasal brownish pecten (Fig. 466); 1^st^ and 2^nd^ metasomal tergites comparatively slender and 1^st^ tergite moderately widened (Fig. 459); 2^nd^ tergite basally and 3^rd^ tergite apically distinctly convex in lateral view (Fig. 453); 3^rd^ tergite coarsely punctate, with complete lamelliform lateral margin (Fig. 453); hind coxa and femur completely dark brown or blackish; hind tibia usually ivory or pale yellowish basally; first and 2^nd^ metasomal tergites reddish or orange.

##### Description.

Lectotype, ♀, length of fore wing 6.9 mm, of body 9.6 mm.

***Head.*** Antenna incomplete, 32 segments remaining; frons mainly with curved or oblique rugae; OOL 1.3 × diameter of posterior ocellus, coarsely rugose and rather matt; vertex densely and rather finely rugose, hardly shiny; anterior part of clypeus densely punctate and flat; ventral margin of clypeus thin and rather forward protruding (Fig. 465); clypeus approx. 5 × wider than long medially; width of hypoclypeal depression 0.8 × minimum width of face (Fig. 463); length of eye 1.4 × temple in dorsal view (Fig. 464); vertex behind stemmaticum coarsely rugose; clypeus largely above lower level of eyes; length of malar space 0.2 × length of eye in lateral view; mandible punctate-rugose and with long setae (Fig. 465).

***Mesosoma.*** Lateral mesoscutal lobes densely and coarsely punctate, with interspaces narrower than punctures but interspaces becoming wider posteriorly, middle lobe coriaceous, but punctate near narrow and distinctly impressed notauli; precoxal area of mesopleuron and metapleuron coarsely and densely rugose punctate; remainder of mesopleuron densely punctate; metanotum with incomplete median carina; scutellum coarsely punctate; axilla largely densely rugose; propodeum rather flat and coarsely reticulate-rugose, medio-longitudinal carina on only anterior half.

**Figures 455–466.**
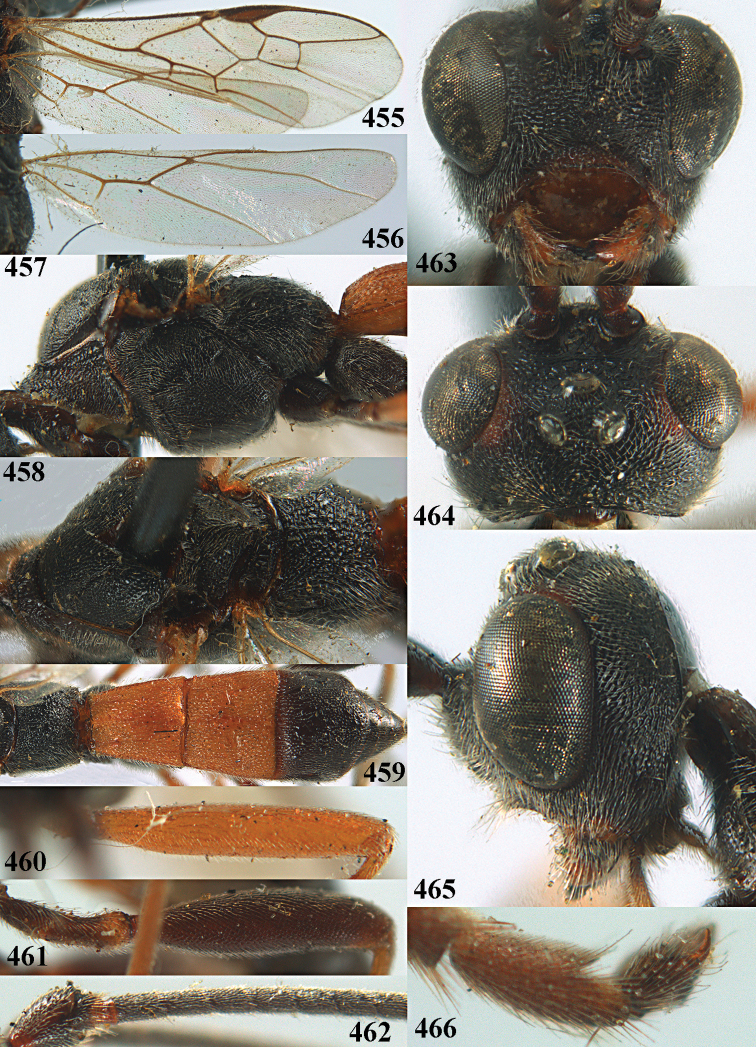
*Aleiodes krulikowskii* (Kokujev), ♀, lectotype **455** fore wing **456** hind wing **457** mesosoma lateral **458** mesosoma dorsal **459** metasoma dorsal **460** fore femur lateral **461** hind femur lateral **462** base of antenna **463** head anterior **464** head dorsal **465** head lateral **466** outer middle tarsal claw.

***Wings.*** Fore wing: r 0.6 × 3-SR (Fig. 455); 1-CU1 horizontal, 0.8 × 2-CU1 and widened; r-m 0.6 × 3-SR; 2^nd^ submarginal cell medium-sized (Fig. 455); cu-a vertical, straight; 1-M nearly straight posteriorly; 1-SR widened; surroundings of M+CU1, 1-M and 1-CU1 evenly setose; M+CU1 curved distally. Hind wing: marginal cell linearly widened, its apical width 2.8 × width at level of hamuli (Fig. 456); 2-SC+R quadrate; m-cu absent; M+CU:1-M = 40:21; 1r-m 1.1 × 1-M.

***Legs.*** Hind tarsal claws robust and with inconspicuous fine subbasal brownish pecten (Fig. 466); hind coxa largely densely finely punctate, dorso-posteriorly with oblique rugae; hind trochantellus slender; length of hind femur and basitarsus 4.3 and 5.6 × their width, respectively; length of inner hind spur 0.4 × hind basitarsus.

***Metasoma.*** First tergite rather convex and moderately widened (Fig. 459), 1.1 × longer than wide apically; 1^st^ and 2^nd^ tergites with medio-longitudinal carina and coarsely longitudinally rugose-punctate; medio-basal area of 2^nd^ tergite triangular and minute (Fig. 459); 2^nd^ suture deep and rather wide; 1^st^ tergite; 2^nd^ tergite basally and 3^rd^ tergite apically distinctly convex in lateral view (Fig. 453); 3^rd^ tergite coarsely punctate, interspaces approx. equal to diameter of punctures, with complete lamelliform lateral margin; remainder of metasoma smooth and shiny; ovipositor sheath rather slender, with long setae and apically truncate (Fig. 454).

***Colour.*** Black; orbit near ocelli reddish brown; fore and middle legs (except blackish or dark brown coxae, trochanters and trochantelli), apex of hind trochantellus and basal third of hind tibia brownish yellow; tarsi darkened and remainder of legs dark brown; palpi (except basally) pale yellowish; mandible yellowish but basally largely dark brown; propleuron and tegula anteriorly dark brown and tegula posteriorly brown; 1^st^ and 2^nd^ metasomal tergites and metasoma ventrally (except apically) orange brown; pterostigma dark brown; veins brown; wing membrane subhyaline.

***Variation.*** Orbit near hind ocellus sometimes only very slightly lighter in colour. Antennal segments: ♂ 68(1); according to the original descriptions of *A. krulikowskii* and *A. csikii*, the female types have 60 and 62 antennal segments, respectively. Apical tergites of ♂ type 2 and no fringe observed.

##### Distribution.

*Hungary, Mongolia, *Romania, Russia (Central and Far East).

#### Aleiodes miniatus

Taxon classificationAnimaliaHymenopteraBraconidae

(Herrich-Schäffer, 1838)

6706941D-AB04-5B9C-89F1-1F6D2D90A343

Figs 467–468 , 469–481

Rogas miniatus Herrich-Schäffer, 1838: 156; Shenefelt 1975: 1238–1239 (type series lost).
Rogas (Rogas) miniatus ; Tobias 1976: 81, 1986: 75–76 (transl.: 122).
Aleiodes (Neorhogas) miniatus ; Papp 1987b: 36, 1991a: 88.
Aleiodes (Chelonorhogas) miniatus ; Belokobylskij et al. 2003: 398.
Aleiodes miniatus ; Bergamasco et al. 1995: 5; Papp 2005: 177.
Rogas bicoloratus Boheman, 1853: 180; Shenefelt 1975: 1239 (as synonym of A. miniatus); Papp 2005: 177 (id.).
Aleiodes formosus Giraud, 1857: 177; Shenefelt 1975: 1239; (as synonym of A. miniatus); Papp 1985a: 159 (lectotype designation and as synonym of A. miniatus), 2005: 177 [examined].

##### Type material.

Lectotype of *A. formosus*, ♀ (MNHN), “[**Austria**, Wien,] Prata 16 juin”, “Austria, Vienne, Prater, 16 juin/Papp 1979”, “Lectotypus *Aleiodes formosus* Gir., 1857, ♀, Papp, 1979”, “*Rogas miniatus* HS ♀, det. Papp J., 1979”.

##### Additional material.

Austria, Czech Republic, France, Finland, Germany, Hungary, Romania, Russia, Sweden, Ukraine, [Kazakhstan, Kyrgyzstan]. Specimens in ZJUH, BZL, SDEI, MNHN, MTMA, NMS, OUM, RMNH, ZSSM. The OUM specimen is labelled “Litchfield L.A. Carr 23” but there are very evidently numerous non-British specimens in the (now somewhat dispersed) Carr collection labelled Litchfield, and good reasons for discounting them as British are given by Perkins (1953). Such labelling may have been a means of identifying ownership of specimens at a time of considerable exchange and identification by others, and there is no evidence that this species has ever been collected in the British Isles. Material examined from central Europe (often labelled “Germany” or “Bohemia”) is mostly much more than 100 years old, when it seems to have been quite readily collected. Three recent specimens (NMS) from different sites in Sweden (Öland: Halltorp, 2015, 2017 and Skåne: Ravlunda, 2018, all N. Johansson) were swept from herb-rich sandy or gravelly grasslands overlying calcareous bedrock, with outstanding biodiversity partly maintained by grazing (Niklas Johansson, pers. comm.). The evident decline of *A. miniatus* in central Europe, as evidenced by specimen data showing a declining number of specimens collected in that region through time, probably reflects the loss of similar steppe habitat and, although a fairly recent (1994) specimen from Romania is in MTMA, it may now be extinct in large parts of central Europe.

##### Molecular data.

MRS950 (Sweden), MRS951 (Sweden).

##### Biology.

Unknown, but it seems to inhabit herb-rich calcareous steppe grasslands. Collected in (May)June–August; presumably univoltine, but we have not examined reared material of this large and distinctive species and there is no indication of how it may overwinter. A series in BZL (one now in NMS) is labelled “Wien D. Au” which can be interpreted as [? wet] woodland near the Danube (M. Schwarz, pers. comm.), which would probably be well under 200 m a.s.l. In contrast, a recent specimen (also in BZL) from Kyrgyzstan was collected higher at 2550 m.

##### Diagnosis.

Maximum width of hypoclypeal depression approx. 0.5 × minimum width of face (Fig. 469); OOL of ♀ approx. twice as long as diameter of posterior ocellus and punctate (Fig. 477); ventral margin of clypeus thin and distinctly protruding in lateral view; length of malar space approx. equal to height of eye in lateral view (Fig. 478); mesoscutal lobes densely punctate; area of precoxal sulcus wide and coarsely rugose; length of vein 1-CU1 of fore wing 0.4 × vein 2-CU1; 2^nd^ submarginal cell of fore wing short and square (Fig. 469); vein 1r-m of hind wing longer than vein 1-M; vein 2-SC+R of hind wing subquadrate; 3^rd^ tergite densely punctate (Fig. 473); head and mesoscutum orange or brownish yellow; basal half of hind tibia (largely) pale yellowish; metasoma (except part of 1^st^ tergite) orange or brownish yellow.

##### Description.

Redescribed ♀ (RMNH) from Russia (Yaaseni). Length of fore wing 6.5 mm, of body 7.9 mm.

***Head.*** Antennal segments of ♀ 65, length of antenna 1.1 × fore wing, its subapical segments somewhat longer than wide; frons with coarse curved rugae; OOL 2.3 × diameter of posterior ocellus, and punctate; vertex densely punctate and shiny; clypeus densely punctate; ventral margin of clypeus thin and distinctly protruding forwards (Fig. 478); width of hypoclypeal depression 0.5 × minimum width of face (Fig. 476); length of eye as long as temple in dorsal view (Fig. 477); vertex behind stemmaticum densely punctate; clypeus just below lower level of eyes; malar space 0.5 × length of eye in lateral view.

***Mesosoma.*** Mesoscutal lobes densely punctate, with minute interspaces and rather shiny; precoxal area of mesopleuron wide and coarsely rugose medially, mesopleuron above it coarsely and densely punctate, even speculum with some punctures; scutellum convex and punctate; propodeum evenly convex and coarsely reticulate-rugose, medio-longitudinal carina incomplete.

***Wings.*** Fore wing: r 0.7 × 3-SR (Fig. 469); 1-CU1 horizontal, 0.4 × 2-CU1; r-m 1.2 × 3-SR; 2^nd^ submarginal cell short (Fig. 469); cu-a inclivous, straight and rather short; 1-M rather curved posteriorly; 1-SR slender and short; surroundings of M+CU1, 1-M and 1-CU1 largely setose. Hind wing: marginal cell evenly widened, its apical width 2.7 × width at level of hamuli (Fig. 470); 2-SC+R subquadrate; m-cu absent; M+CU:1-M = 35:16; 1r-m 1.5 × 1-M.

***Legs.*** Tarsal claws with only three conspicuous brownish and widened bristles basally (Fig. 481); hind coxa densely and rather finely punctate; hind trochantellus medium-sized; length of hind femur and basitarsus 4.0 and 4.2 × their width, respectively; length of inner hind spur 0.5 × hind basitarsus.

***Metasoma.*** First tergite evenly convex, 0.9 × as long as wide apically; 1^st^ tergite coarsely reticulate-rugose, 2^nd^ tergite coarsely and densely rugose-punctate, without median carina; medio-basal area of 2^nd^ tergite short triangular (Fig. 473); 2^nd^ suture deep and finely crenulate; basal half of 3^rd^ tergite densely punctate, remainder of metasoma superficially micro-sculptured; apical half of 3^rd^ tergite with sharp lateral crease; ovipositor sheath moderately wide, with long setae and apically rounded (Fig. 468).

***Colour.*** Brownish yellow; antenna, mesosternum, mesopleuron (except antero-dorsally), metapleuron, propodeum, 1^st^ tergite, and ovipositor sheath black; propleuron, small patch on middle mesoscutal lobe anteriorly, apices of femora, fore and middle tibiae, tarsi, apical half of hind tibia, veins, and pterostigma dark brown; wing membrane subhyaline; basal half of hind tibia pale yellowish.

***Variation.*** Second submarginal cell square or somewhat narrower; propleuron dark brown or yellowish; mesopleuron black or yellowish anteriorly and dorsally; medio-longitudinal carina of posterior half of propodeum absent, obsolescent or incomplete. Antennal segments: ♀ 64(5), 65(3), 66(2), 67(3), 68(2), 70(1); ♂ 61(1), 64(2), 66(1), 67(2), 68(1), 69(1), 70(1). On this limited evidence there seems to be little, if any, difference in the number of antennal segments between the sexes. Males are very similar but have the metasoma infuscated apically and the apical tergites are type 3, setae short and dense, glabrous stripe rather narrow and fringe not observed.

##### Distribution.

Austria, Czech Republic, Finland, *France, Germany, Hungary, Kazakhstan, *Kyrgyzstan, *Romania, Russia, Sweden, *Ukraine.

**Figures 467, 468.**
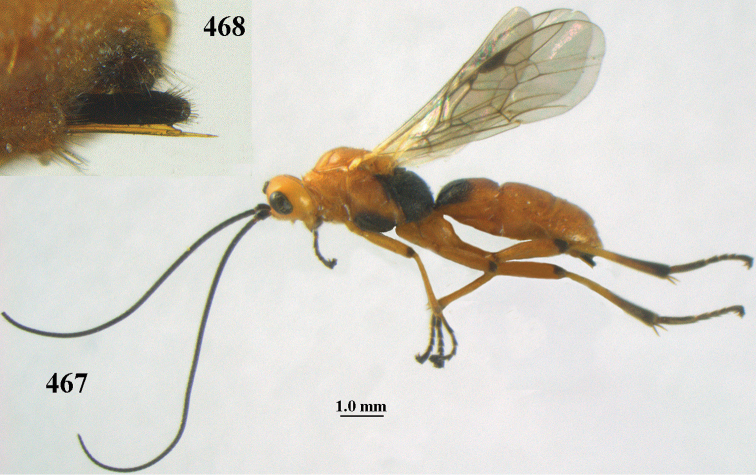
*Aleiodes miniatus* (Herrich-Schäffer)), ♀, Russia, Stavropolskij kraj **467** habitus lateral **468** ovipositor sheath lateral.

**Figures 469–481.**
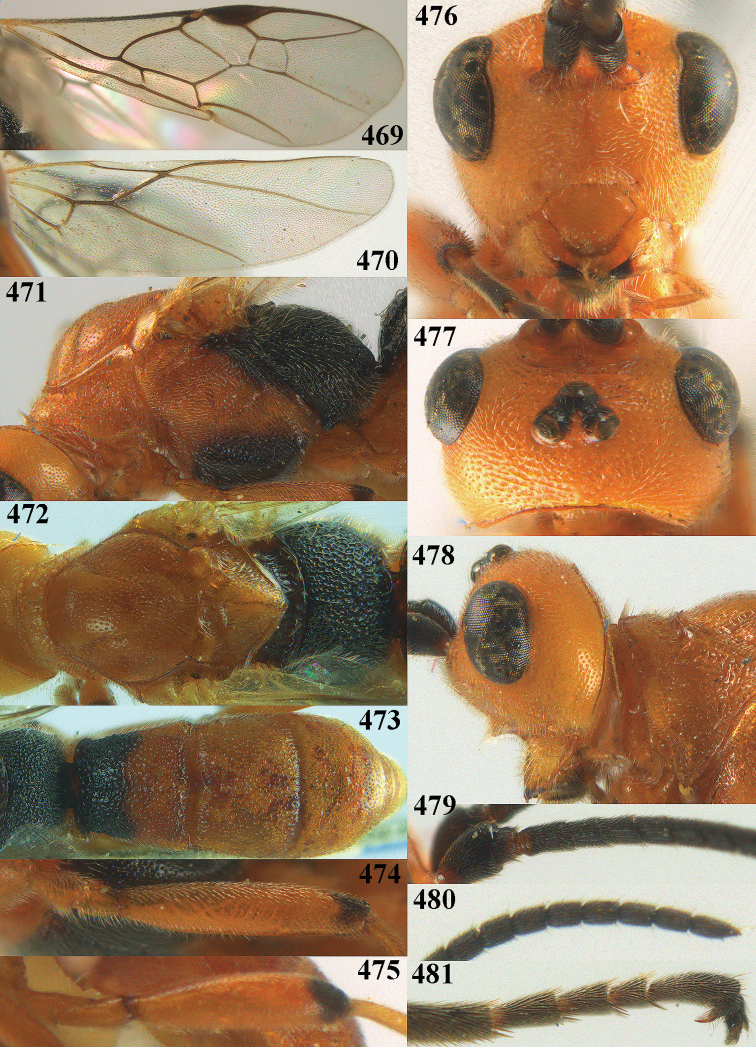
*Aleiodes miniatus* (Herrich-Schäffer)), ♀, Russia, Stavropolskij kraj **469** fore wing **470** hind wing **471** mesosoma lateral **472** mesosoma dorsal **473** metasoma dorsal **474** fore femur lateral **475** hind femur lateral **476** head anterior **477** head dorsal **478** head lateral **479** base of antenna **480** apex of antenna **481** inner hind tarsal claw.

#### Aleiodes morio

Taxon classificationAnimaliaHymenopteraBraconidae

(Reinhard, 1863)

13CC2154-7801-57A1-BC88-A29E700F37D9

Figs 482–483 , 484–495 , 496 , 497–503

Rogas morio Reinhard, 1863: 255; Shenefelt 1975: 1239.
Rogas (Rogas) morio ; Tobias 1976: 83, 1986: 76 (transl.: 124).
Aleiodes (Neorhogas) morio ; Papp 1985a: 160 (♀ type lost, designation as lectotype), 1991a: 92.
Aleiodes (Chelonorhogas) morio ; Belokobylskij et al. 2003: 398.
Aleiodes morio ; Papp 2005: 177.

##### Type material.

Lectotype ♀ from southern Germany most probably lost (Papp 1985a); the only paralectotype (♂, ZMB) is a melanistic male of *A. coxalis* (Spinola, 1808) which does not fit the original description. The paralectotype was listed as *A. tristis* Wesmael by Papp (1985a).

##### Additional material.

1 ♀ (MTMA), “[**Hungary**:] Budapest, Rákospalita, 4.iv.%”, “*Rhogas morio* Reinh. ♀, det. Szépligeti”, “*Aleiodes morio* Reinh., ♀, det. Papp J., 1984”; 1 ♂ (MTMA), “[Hungary:] Nadap, 19–21.iv.1951, Móczár Bajári”; 1 ♂ (ZSSM), “[? **Germany**:] *Rogas* n. sp.?, /: Ruthe, Berlin[?]/”; 1 ♀ (FMNH), “Suomi [= **Finland**:] U: Nurmijärvi, 6712:373, 13.v.1984, M. Koponen”; 2 ♀ + 1 ♂ (FMNH, RMNH), id., but 15.v.1984.

##### Molecular data.

None.

##### Biology.

Unknown. Specimens of both sexes have been collected in April and May, from which from which it is safe to surmise that the winter is passed in the mummy as the male would not hibernate. But we have seen no reared material nor any indication of habitat for this central European species. Its early flight time might be one reason why it is seldom collected and apparently rare.

##### Diagnosis.

Maximum width of hypoclypeal depression 0.5–0.6 × minimum width of face; OOL of ♀ 0.8 × (of ♂ 0.9 ×) diameter of posterior ocellus and rugose; ventral margin of anterior part of clypeus comparatively sharp and more or less protruding in lateral view (Fig. 493); head rather transverse (Fig. 492); mesoscutal lobes coriaceous; precoxal area of mesopleuron rugose medially; vein 1-CU1 0.3–0.4 × vein 2-CU1 and 0.3 × vein m-cu; hind tarsal claws with conspicuous and robust brownish pecten (Figs 494, 503); posterior orbit black; pterostigma of ♀ pale brown medially, of ♂ dark brown; coxae and femora completely black or dark brown; hind tibia usually ivory or pale yellowish basally; 1^st^ and 2^nd^ metasomal tergites of both sexes black. According to Papp (1985) most closely related to *A. sibiricus* (Kokujev), but that species does not have all black females and has the shape of the clypeus different. According to the original description the pterostigma is yellowish and laterally darkened, 1^st^ subdiscal cell of the fore wing rather short, because vein cu-a distinctly more postfurcal than its own length (ca twice its own length) and meaning vein 1-CU1 of intermediate [approx. 0.6 ×] length of 2-CU1 and 0.8 × vein m-cu] [= “discoidali posterior brevior” as indicated for *A. pallidicornis*], precoxal sulcus area rugose medially, 1^st^ tergite twice wider posteriorly than basally, hind leg black, except pale yellowish dorso-basal area of hind tibia and palpi dark brown. Here we accept the interpretation of the first reviser (Szépligeti 1906) despite the difference in the shape of the 1^st^ subdiscal cell, because it may be part of intraspecific variation.

##### Description.

Redescribed ♀ (MTMA) from Hungary (Budapest). Length of fore wing 7.5 mm, of body 9.4 mm.

***Head.*** Antenna incomplete, 47 segments remaining (54 in lectotype), length of complete antenna approx. 0.9 × fore wing, its subbasal and subapical segments short; frons largely rugose; OOL 0.8 × diameter of posterior ocellus, and mainly rugulose and with satin sheen; depression near posterior ocellus smooth; vertex densely rugulose and with satin sheen; clypeus with some punctures; ventral margin of clypeus rather thin and protruding forwards (Fig. 493); width of hypoclypeal depression 0.6 × minimum width of face (Fig. 491); length of eye 1.7 × temple in dorsal view (Fig. 492); vertex behind stemmaticum punctate-rugulose; clypeus largely above lower level of eyes; length of malar space 0.3 × length of eye in lateral view.

***Mesosoma.*** Mesoscutal lobes densely and finely punctate, with satin sheen; precoxal area of mesopleuron widely and densely rugose, but densely punctate posteriorly; middle of mesopleuron densely rugulose and dorsally coarsely rugose; metapleuron largely rugose; scutellum punctate-coriaceous; propodeum rather flat and densely rugose or rugulose, medio-longitudinal carina complete, and without protruding carinae laterally.

***Wings.*** Fore wing: r 0.5 × 3-SR (Fig. 484); 1-CU1 slightly oblique, 0.3 × 2-CU1; r-m 0.7 × 3-SR; 2^nd^ submarginal cell robust and posteriorly somewhat diverging (Fig. 484); cu-a inclivous, straight; 1-M slightly curved posteriorly; 1-SR wide; surroundings of M+CU1, 1-M and 1-CU1 densely but inconspicuously setose. Hind wing: marginal cell linearly widened, its apical width 2.4 × width at level of hamuli (Fig. 485); 2-SC+R short and subquadrate; m-cu present anteriorly; M+CU:1-M = 4:3; 1r-m 0.6 × 1-M.

***Legs.*** Tarsal claws bristly setose, medium-sized, and with robust pecten basally (cf. Fig. 494); hind leg missing in redescribed specimen.

***Metasoma.*** First tergite evenly convex, 0.9 × as long as wide apically; 1^st^ tergite with medio-longitudinal carina; 1^st^ and 2^nd^ tergites and basal half of 3^rd^ tergite finely and densely longitudinally rugulose; medio-basal area of 2^nd^ tergite short triangular (Fig. 488); 2^nd^ suture distinct and finely crenulate; remainder of metasoma superficially micro-sculptured; 4^th^ tergite without sharp lateral crease; ovipositor sheath wide, with rather long setae and apically truncate (Fig. 483).

***Colour.*** Black; palpi brownish yellow, but basally dark brown; tegulae pale yellowish; legs (except pale base of tibiae), metasoma ventrally and veins dark brown; pterostigma brown and medially yellowish brown; wing membrane subhyaline.

***Variation.*** Clypeus distinctly to moderately protruding and ventrally rather thin to thick. Antennal segments of ♀ 51(1), 52(1), 54(1), of ♂ 55(1). Males are very similar to the redescribed female (including the wing venation: Fig. 497), apical tergites type 1 with fringe not observed and probably absent (Fig. 498), mesopleuron rugulose or punctate medially and pterostigma entirely dark brown.

##### Distribution.

*Finland, Germany, Hungary.

##### Notes.

The lost lectotype from Germany had hyaline wings (which separates it from the *A. carbonarius*/ *grassator*/ *carbonaroides* complex), the pterostigma paler medially than laterally (entirely dark brown), base of the hind tibia pale yellow (black in ♂) and the body of ♀ entirely black (more or less yellowish or reddish).

**Figures 482, 483.**
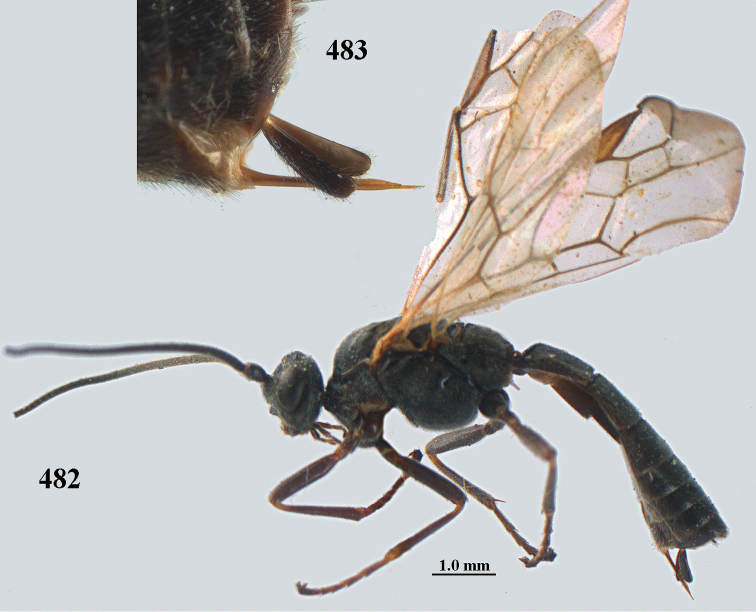
*Aleiodes morio* (Reinhard), ♀, Hungary, Budapest **482** habitus lateral **483** ovipositor sheath lateral.

**Figures 484–495.**
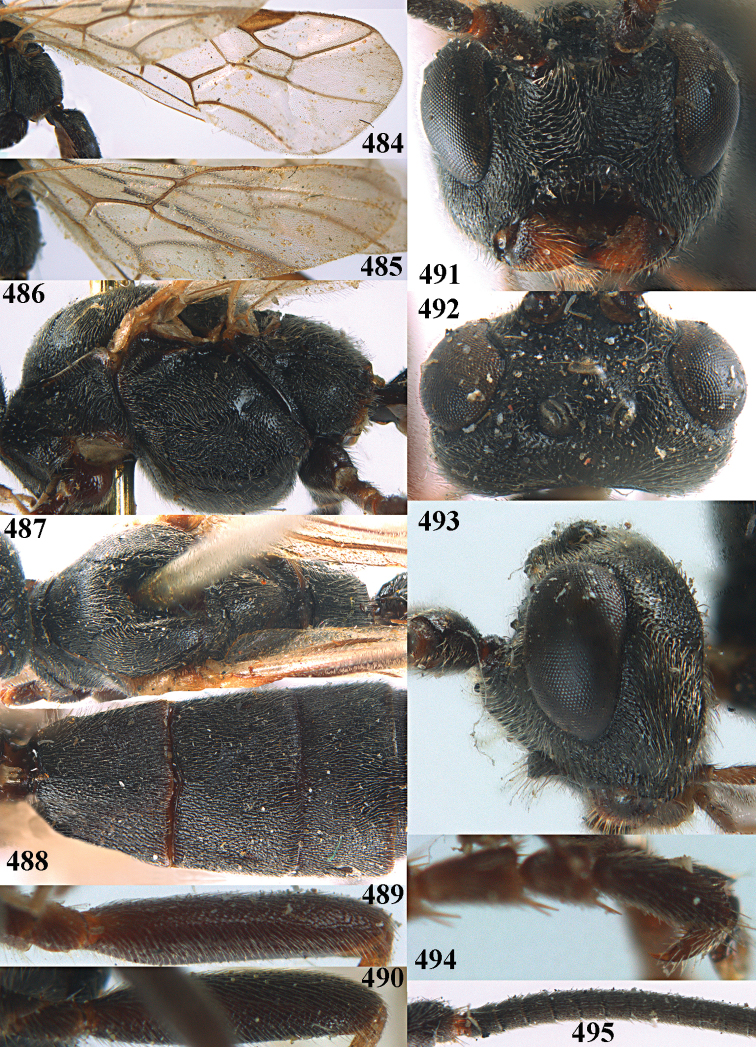
*Aleiodes morio* (Reinhard), ♀, Hungary, Budapest, but 490 of ♂ from Nadap **484** fore wing **485** hind wing **486** mesosoma lateral **487** mesosoma dorsal **488** 1^st^–3^rd^ metasomal tergites dorsal **489** fore femur lateral **490** hind femur lateral **491** head anterior **492** head dorsal **493** head lateral **494** outer middle tarsal claw **495** base of antenna.

**Figure 496.**
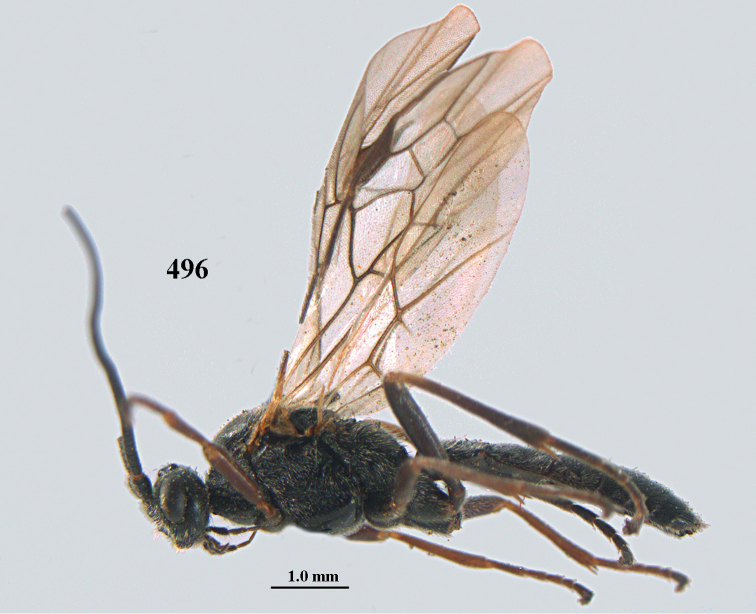
*Aleiodes morio* (Reinhard), ♂, Hungary, Nadap, habitus lateral.

**Figures 497–503.**
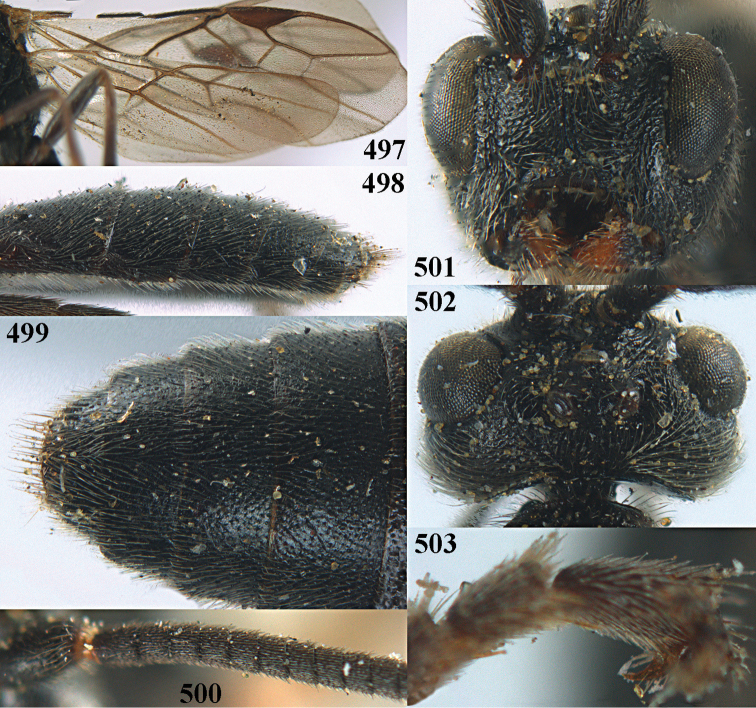
*Aleiodes morio* (Reinhard), ♂, Hungary, Nadap **497** wings **498** apex of metasoma lateral **499** apex of metasoma dorsal **500** base of antenna **501** head anterior **502** head dorsal **503** inner hind tarsal claw.

#### Aleiodes nigrifemur

Taxon classificationAnimaliaHymenopteraBraconidae

van Achterberg & Shaw sp. nov.

BC5C8E9C-EA96-55BC-B052-089A49510904

http://zoobank.org/2535423C-36E7-4ECC-9FC3-362039FC4928

Figs 504–505 , 506–518

##### Type material.

Holotype, ♀, (RMNH), “**Greece**, Peloponn[esus], Chelmos, 1700 m, 29.v.1987, H. Teunissen”.

##### Molecular data.

None.

##### Biology.

Unknown; the only known specimen was collected at the end of May which gives no clue of voltinism or how the winter is passed.

##### Diagnosis.

Maximum width of hypoclypeal depression 0.6 × minimum width of face (Fig. 513); OOL of ♀ 1.6 × longer than diameter of posterior ocellus and rugulose (Fig. 514); width of clypeus intermediate apically, but strongly protruding in lateral view (Fig. 515); lobes of mesoscutum densely punctate, coriaceous between punctures; precoxal area widely rugose, and posteriorly punctate; vein 1-CU1 of fore wing 0.2 × vein 2-CU1 (Fig. 506); hind tarsal claws rather robust and with few yellowish pectinal teeth (Fig. 517); 3^rd^ antennal segment of ♀ and basal third of hind femur black; basal third of hind tibia pale yellowish, contrasting with black basal half of hind femur. Similar to *A. morio* (Reinhard), but has pterostigma black (pale brown in *A. morio*), fore wing darkened apically (subhyaline), vein 1-M of hind wing linear with M+CU (angled); metasoma largely yellowish brown (entirely blackish) and eye in lateral view comparatively small (eye larger).

##### Description.

Holotype, ♀, length of fore wing 7.2 mm, of body 8.2 mm.

***Head.*** Antennal segments of ♀ 62, length of antenna 1.1 × fore wing, its subapical segments medium-sized (Fig. 516); frons largely rugose; OOL 1.6 × diameter of posterior ocellus, rugulose and shiny; depression near posterior ocellus rugose; vertex largely rugose, rather shiny; clypeus rugulose; ventral margin of clypeus intermediate and distinctly protruding forwards (Fig. 515; as face dorsally); width of hypoclypeal depression 0.6 × minimum width of face (Fig. 513); length of eye 1.3 × temple in dorsal view (Fig. 514); vertex behind stemmaticum rugulose; clypeus largely above lower level of eyes; length of malar space 0.4 × length of eye in lateral view.

***Mesosoma.*** Mesoscutal lobes densely punctate, rather shiny and interspaces coriaceous; precoxal area of mesopleuron widely rugose but posteriorly punctate, and area above it densely punctate or rugulose; metapleuron densely punctate dorsally and rugose ventrally; metanotum with short median carina anteriorly; scutellum remotely punctate, with some lateral rugae; propodeum rather short and flat, coarsely reticulate-rugose, medio-longitudinal carina complete, and without protruding carinae laterally.

***Wings.*** Fore wing: r 0.5 × 3-SR (Fig. 506); 1-CU1 slightly oblique, 0.2 × 2-CU1; r-m 0.7 × 3-SR; 2^nd^ submarginal cell medium-sized (Fig. 506); cu-a inclivous, straight; 1-M curved posteriorly; 1-SR wider than 1-M; surroundings of M+CU1, 1-M and 1-CU1 largely glabrous. Hind wing: marginal cell linearly widened, its apical width 1.9 × width at level of hamuli (Fig. 506); 2-SC+R short and longitudinal; m-cu present anteriorly; vein 2-1A comparatively long (Fig. 506); M+CU:1-M = 24:37; 1r-m 0.65 × 1-M.

***Legs.*** Tarsal claws rather robust, bristly setose and few small yellowish teeth (Fig. 517); hind coxa largely punctate and with some oblique striae dorsally; hind trochantellus rather robust; length of hind femur and basitarsus 4.5 and 5.1 × their width, respectively; length of inner hind spur 0.45 × hind basitarsus.

***Metasoma.*** First tergite rather flat medially, 0.8 × as long as wide apically; 1^st^ tergite and anterior half of 2^nd^ tergite with medio-longitudinal carina; 1^st^–2^nd^ tergites densely longitudinally rugose; 3^rd^ tergite (except posterior third) mainly rugulose; medio-basal area of 2^nd^ tergite triangular and rather distinct (Fig. 509); 2^nd^ suture rather deep and crenulate; remainder of metasoma superficially micro-sculptured or smooth; 4^th^ and apical half of 3^rd^ tergite without sharp lateral crease; ovipositor sheath wide, with long setae and apically truncate (Fig. 505).

***Colour.*** Black; maxillary palp apically, basal 0.4 of hind tibia and tegulae pale yellowish; mandible (but with dark brown patch), side of pronotum dorso-posteriorly, fore and middle tibiae, hind basitarsus basally, 1^st^ tergite apically, 2^nd^–5^th^ tergites orange brown; remainder of legs dark brown; remainder of palp, veins and pterostigma dark brown; lateral lobes of mesoscutum (except anteriorly and medially) dark reddish brown; wing membrane subhyaline, but apically infuscated (Fig. 506).

##### Distribution.

Greece (main).

##### Etymology.

The species is named after its black femur; *niger* is Latin for black, dark, dusky.

**Figures 504, 505.**
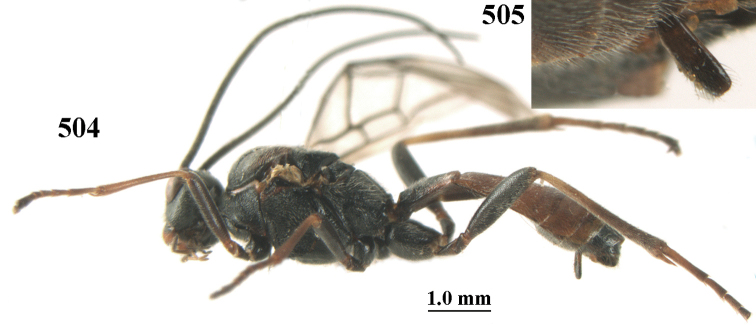
*Aleiodes nigrifemur* sp. nov. ♀, holotype **504** habitus lateral **505** ovipositor sheath lateral.

**Figures 506–518.**
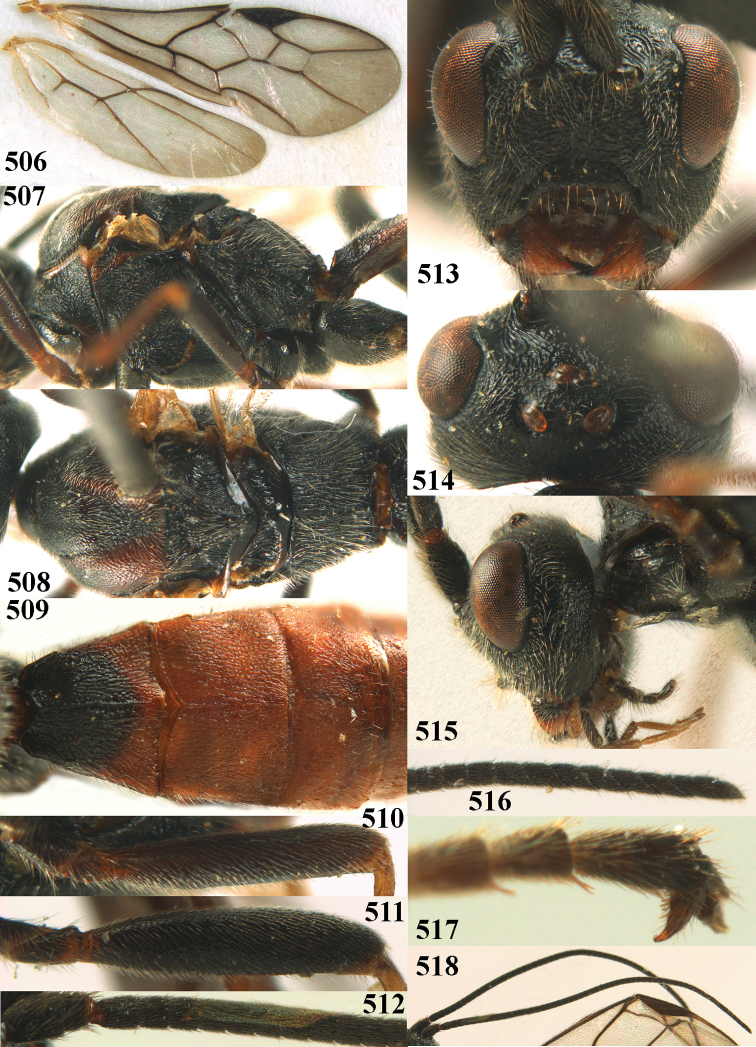
*Aleiodes nigrifemur* sp. nov. ♀, holotype **506** wings **507** mesosoma lateral **508** mesosoma dorsal **509** 1^st^–3^rd^ metasomal tergites dorsal **510** fore femur lateral **511** hind femur lateral **512** base of antenna **513** head anterior **514** head dorsal **515** head lateral **516** apex of antenna **517** outer hind tarsal claw **518** antennae.

#### Aleiodes nobilis

Taxon classificationAnimaliaHymenopteraBraconidae

(Haliday [in Curtis], 1834)

1EFAF609-9B50-5562-9833-8120CCCF5138

Figs 519–521 , 522–535 , 536 , 537–542

Rogas nobilis Haliday [in Curtis], 1834: 512; Papp 2005: 176 (as syn. of A. ductor).
Rogas ductor var. nobilis ; Shenefelt 1975: 1227.
Aleiodes (Neorhogas) nobilis ; Papp 1991a: 70 (as synonym of A. ductor).
Aleiodes (Chelonorhogas) nobilis ; van Achterberg 1997: 62 (both syntypes lost); Belokobylskij et al. 2003: 398.
Aleiodes nobilis ; Bergamasco et al. 1995: 5; O’Connor et al. 1999: 91–92; Papp 2005: 177.
Rogas medianus Thomson, 1892: 1668; Shenefelt 1975: 1237; van Achterberg 1997: 62 (as synonym of A. nobilis); Belokobylskij et al. 2003: 398 (id.); Papp 2005: 177 (id.) [examined].
Rogas (Rogas) medianus ; Tobias 1976: 85, 1986: 80 (transl.: 133).
Aleiodes (Neorhogas) medianus ; Papp 1991a: 69; Belokobylskij 1996: 13.
Aleiodes medianus ; Papp and Vas 2016: 152.
Aleiodes ductor ; auct. p.p.

##### Type material.

Neotype of *A. nobilis* here designated: ♀ (NMS), “[**Scotland**:] W. Ross, Coppachy, Letterewe Estate, ix.2007, Mal. trap, P. Tinsley-Marshall”, “BCLDQR _00123”. Lectotype of *A. medianus*, ♀ (ZIL), “[**Sweden**:] Scan”, “*medianus* m.”, “Funnen vid Esperöd I Skåne, teste Papp J., 1983”, “Lectotypus *Rogas medianus* Thoms., 1891, ♀, Papp, 1983”, “*Aleiodes medianus* Th., ♀, det. Papp J., 1983”. The lectotype designation for *A. nobilis* is necessary for nomenclatural stability, because the type series is lost (van Achterberg, 1997) and the species has been confused with similar species in the past. The specimen from Scotland is selected neotype because it fits well the original description, Scotland is relatively close to both type localities (near Holywood in Ireland and Monk’s Wood in England) and it is in good condition.

##### Additional material.

Austria, British Isles (Scotland: V.C.s 72, 88, 105; Ireland: V.C. H29), Bulgaria, Croatia, Czech Republic, Finland, Germany, Hungary, Italy, Moldova, Netherlands (LI: Gulpen; St. Pietersberg; Geulle (Bunderbos); NB: Udenhout (“de Brand”), OV: Voorst (Twello), ZH: Lexmond), Poland, Romania, Russia, Serbia, Slovakia, Sweden. Specimens in ALC, ZJUH, BZL, HSC, MTMA, NMS, NRS, RMNH, SDEI, Tullie House Museum Carlisle, USNM, ZSSM.

##### Molecular data.

MRS401 (Finland), MRS880 (Russia), MRS881 (UK).

##### Biology.

Collected predominantly in grassy places, June–October. Reared from the noctuid *Autographa gamma* (Linnaeus) (4 [1 NRS, 2 HSC], Germany, Sweden; H. Schnee) but, in view of its moderately northern areas of occurrence, it seems very likely that other plusiine noctuids would play an important part in its host range. The rearing data indicate that it is plurivoltine, and adult emergence in November from mummies forming in October suggests that it overwinters in the host larva.

##### Diagnosis.

**Figures 519–521.**
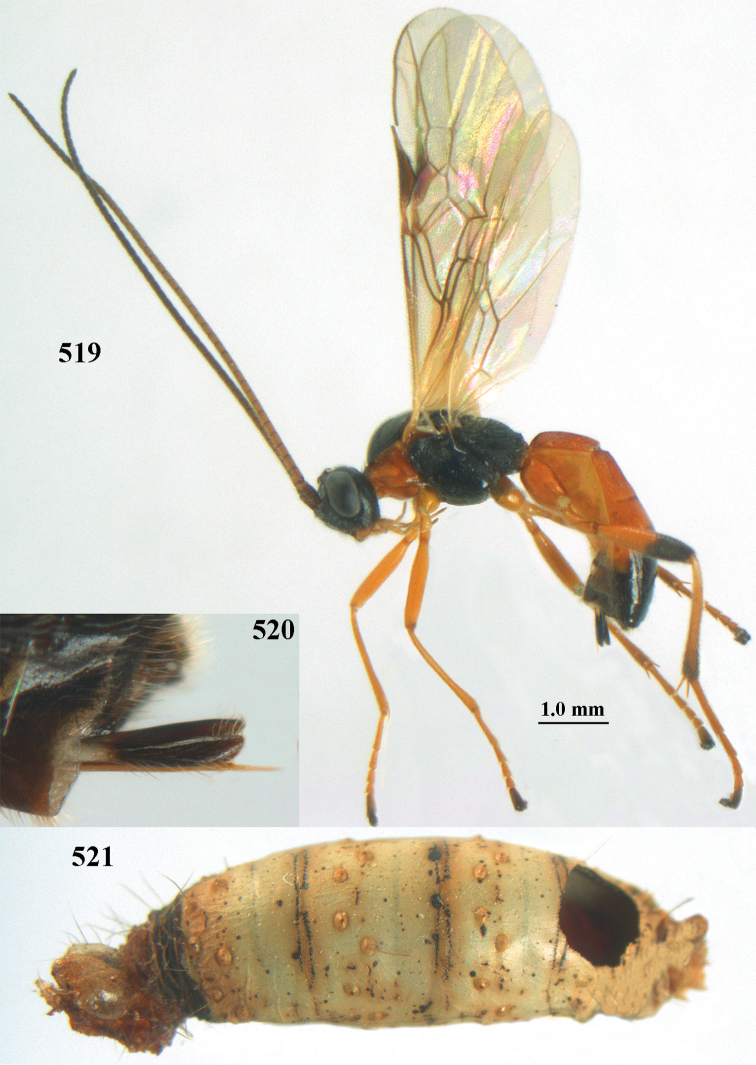
*Aleiodes nobilis* (Haliday), ♀, neotype **519** habitus lateral **520** ovipositor sheath lateral **521** mummy of *Autographa gamma* Linnaeus (Germany, Lindenhayn).

**Figures 522–535.**
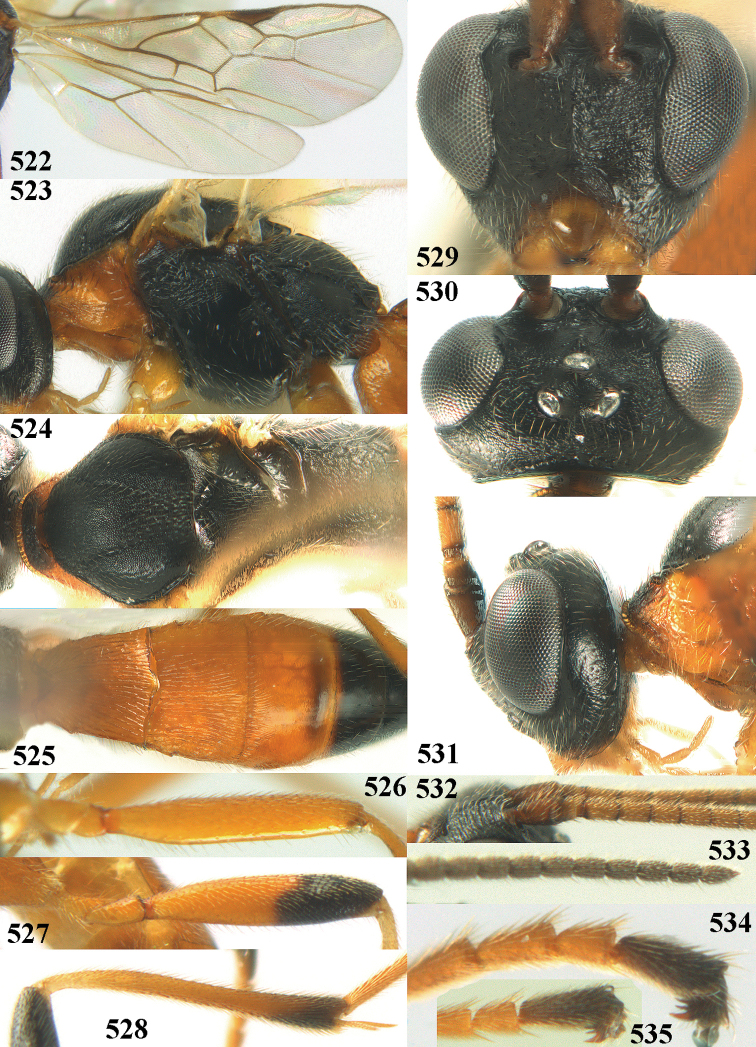
*Aleiodes nobilis* (Haliday), ♀, neotype **522** wings **523** mesosoma lateral **524** mesosoma dorsal **525** 1^st^–4^th^ metasomal tergites dorsal **526** fore femur lateral **527** hind femur lateral **528** hind tibia lateral **529** head anterior **530** head dorsal **531** head lateral **532** base of antenna **533** apex of antenna **534** outer hind tarsal claw **535** outer fore tarsal claw.

Maximum width of hypoclypeal depression approx. 0.3 × minimum width of face (Fig. 529); OOL of ♀ approx. as long as diameter of posterior ocellus and granulate (Fig. 530); ventral margin of clypeus thick and not protruding in lateral view (Fig. 531); mesoscutal lobes (as vertex) very finely and densely granulate, with satin sheen; precoxal area smooth; vein 1-CU1 0.7–1.3 × vein 2-CU1 and vein 1-CU1 wider than 2-CU1 (Fig. 522); tarsal claws with distinct dark brown pecten (Figs 534, 535); hind femur and basitarsus slender (Figs 519, 527); 1^st^ metasomal tergite comparatively slender (Fig. 525); at least basal half of 4^th^–6^th^ tergites of ♂ usually with long dense setosity (Figs 537, 538); head black; pronotum usually (partly) orange brown; both tegula and humeral plate equally yellowish; base of hind tibia pale yellowish; hind basitarsus brownish yellow, strongly contrasting with dark brown telotarsus; 2^nd^ tergite yellowish or reddish.

##### Description.

Redescribed ♀ (RMNH) from Slovakia (Kubrica). Length of fore wing 5.1 mm, of body 5.9 mm.

***Head.*** Antennal segments of ♀ 48, length of antenna 1.25 × fore wing, its subapical segments slender (Fig. 533); frons matt and granulate; OOL equal to diameter of posterior ocellus, and coriaceous-granulate; vertex coriaceous-granulate and rather dull; clypeus punctate-coriaceous; ventral margin of clypeus thick and not protruding forwards (Fig. 531); width of hypoclypeal depression 0.3 × minimum width of face (Fig. 529); length of eye 2.5 × temple in dorsal view (Fig. 530); vertex behind stemmaticum granulate; clypeus near lower level of eyes; length of malar space 0.3 × length of eye in lateral view.

***Mesosoma.*** Mesoscutal lobes densely and finely granulate, rather shiny near tegulae; precoxal area of mesopleuron smooth, surroundings sparsely punctulate; metapleuron mostly granulate; metanotum without median carina; scutellum granulate and with lateral carina; propodeum slightly convex, granulate with spaced rugosity, medio-longitudinal carina only anteriorly present, and no protruding carinae laterally.

***Wings.*** Fore wing: r 0.6 × 3-SR (Fig. 522); 1-CU1 straight, 1.2 × 2-CU1; r-m 0.7 × 3-SR; 2^nd^ submarginal cell short (Fig. 522); cu-a vertical, nearly straight; 1-M slightly curved posteriorly; 1-SR rather narrow; surroundings of M+CU1, 1-M and 1-CU1 evenly setose. Hind wing: marginal cell linearly widened, its apical width 2.4 × width at level of hamuli (Fig. 522); 2-SC+R subquadrate; m-cu absent; M+CU:1-M = 14:13; 1r-m 0.6 × 1-M.

***Legs.*** Tarsal claws with conspicuous and robust dark brown pecten (Figs 534, 535); hind coxa sparsely finely punctate; hind trochantellus robust; length of hind femur and basitarsus 4.7 and 8.0 × their width, respectively; length of inner hind spur 0.5 × hind basitarsus.

***Metasoma.*** First tergite rather flattened, as long as wide apically; 1^st^ and 2^nd^ tergites rather regularly sublongitudinally striate, without medio-longitudinal carina on 2^nd^ tergite; medio-basal area of 2^nd^ tergite wide triangular and rather distinct (Fig. 525); 2^nd^ suture rather deep and narrow; basal quarter of 3^rd^ tergite finely striate, remainder of metasoma smooth; 4^th^ and apical half of 3^rd^ tergite without sharp lateral crease; ovipositor sheath rather long and slender, with long setae and apically rounded (Fig. 520).

***Colour.*** Black; pterostigma (except yellowish extreme base and apex), veins (except brown vein C+SC+R), clypeus, apical third of hind tibia and telotarsus dark brown; palpi, tegulae, remainder of tibiae and tarsi, pale yellowish; apex of middle femur and apical half hind femur, black; remainder of legs, antenna (but apical segments and to some degree scapus infuscate) yellowish brown; 1^st^–3^rd^ metasomal tergites (except black medial patch of 1^st^ tergite), propleuron and pronotum orange; wing membrane subhyaline.

***Variation.*** 1-CU1 0.7–1.2 × 2-CU1; striae of 2^nd^ tergite regularly sublongitudinal or somewhat diverging posteriorly (Fig. 525), but in male sometimes only granulate; basal third or half of 3^rd^ tergite finely striate, rarely completely smooth; fore and middle femora black or dark brown apically or brownish yellow; pronotal side orange to dark brown dorsally; dark patch of 1^st^ tergite absent (e.g., lectotype of *A. medianus*), small, large or occupying most of tergite; posterior half of 3^rd^ tergite orange or black. Antennal segments: ♀ 46(3), 47(8), 48(3), 49(5), 50(5); ♂ 45(2), 46(2), 47(6), 48(5), 49(6); with little difference in the number of antennal segments between the sexes. Males are very similar, but apical tergites type 4, dense setae (making the tergites appear concave) and fringe strong (Figs 537, 538).

##### Distribution.

*Austria, British Isles (Scotland, Ireland), Bulgaria, *Croatia, Czech Republic, Finland, Germany, Hungary, Italy, Moldova, Netherlands, *Poland, *Romania, Russia, *Serbia, *Slovakia, Sweden.

**Figure 536.**
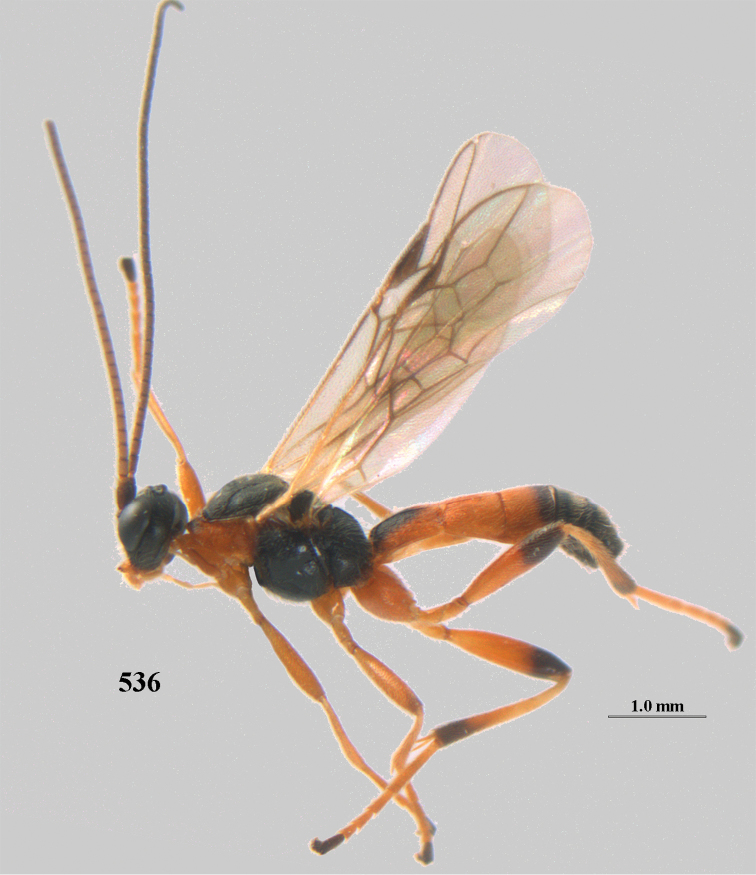
*Aleiodes nobilis* (Haliday), ♂, Netherlands, Gulpen, habitus lateral.

**Figures 537–542.**
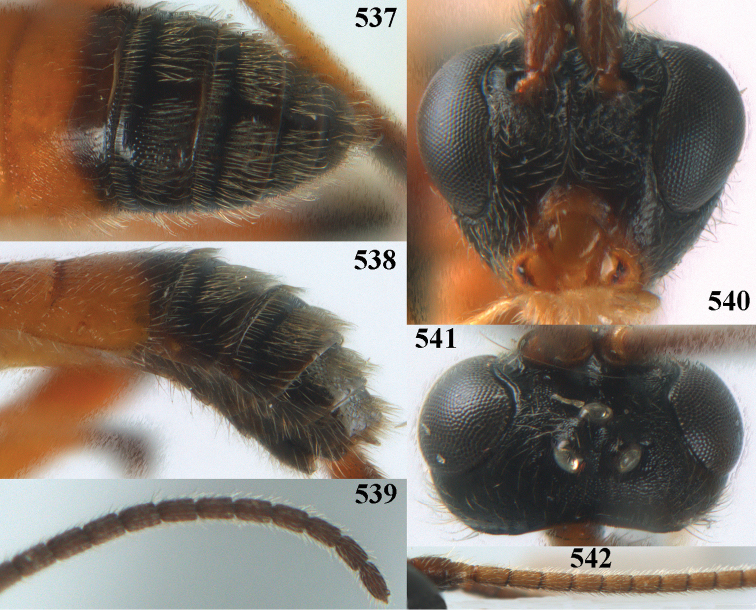
*Aleiodes nobilis* (Haliday), ♂, Netherlands, Gulpen **537** apex of metasoma dorsal **538** apex of metasoma lateral **539** apex of antenna **540** head anterior **541** head dorsal **542** base of antenna.

#### Aleiodes pallidicornis

Taxon classificationAnimaliaHymenopteraBraconidae

(Herrich-Schäffer, 1838)

46C8126E-8FD3-54D4-B32E-95E448D882E1

Figs 543–544 , 545–557

Rogas pallidicornis Herrich-Schäffer, 1838: 156; Shenefelt 1975: 1241; Zaykov 1980b: 87.
Rhogas pallidicornis ; Fahringer 1932: 266.
Rogas (Rogas) pallidicornis ; Tobias 1976: 84, 1986: 80 (transl.: 130).
Aleiodes (Neorhogas) pallidicornis ; Papp 1987b: 36, 1991a: 70 (as senior synonym of A. hirtus).
Aleiodes (Chelonorhogas) pallidicornis ; Belokobylskij 2000: 42; Ku et al. 2001: 236; Belokobylskij et al. 2003: 398.
Aleiodes pallidicornis ; Papp 2005: 177.
Rhogas pallidipennis Dalla Torre, 1898: 221 [invalid emendation].
Rogas ductor auctt. p.p. [North & Central Europe, e.g., Lozan et al. 2010: 17].

##### Type material.

Neotype of *A. pallidicornis* here designated, ♀ (RMNH), “[**Netherlands**], [Zuid-]Holland, Asperen, 6.viii.1972, C.J. Zwakhals”. The neotype designation is necessary for nomenclatorial stability, because the types of Braconidae described by Herrich-Schäffer are lost (Horn and Kahle 1935–37; no specimens could be found by the first author in ZMB), and the species has been confused with similar species in the past. The specimen is selected because it fits well the original description, Netherlands is relatively close to the probable type location in Germany and it is in excellent condition.

##### Additional material.

Austria, Belarus, British Isles (Scotland: V.C. ?92), Bulgaria, Germany, Hungary, Italy, Montenegro, Netherlands (ZH: Asperen; Schoonrewoerd; Waarder), Romania, Russia, Slovakia, Switzerland, Turkey [Iran, North Korea]. Specimens in ZJUH, BZL, MRC, MSC, MTMA, NMS, RMNH, UNS, ZSSM.

##### Molecular data.

MRS885 (Russia).

##### Biology.

Very little is known. Specimens collected in (May) June–August (September), the great majority in June-July strongly suggesting that it is at least largely univoltine. The Dutch specimens were collected in fairly humid coppice woods. The single British specimen (ZJUH; G.T. Lyle) was reared (emergence 20.vi.1926) from a “noctua” caterpillar collected by E.A. Cockayne in Aberdeenshire. The mummy is lost. At that time, the term “noctua” was used generally for Noctuidae rather than in the restricted sense of the genus of that name, and it would appear (as Cockayne was by then a distinguished amateur lepidopterist) that the host larva did not belong to an obviously identifiable species. Otherwise we have not seen reared material, and there is no indication of how the winter is passed.

##### Diagnosis.

**Figures 543, 544.**
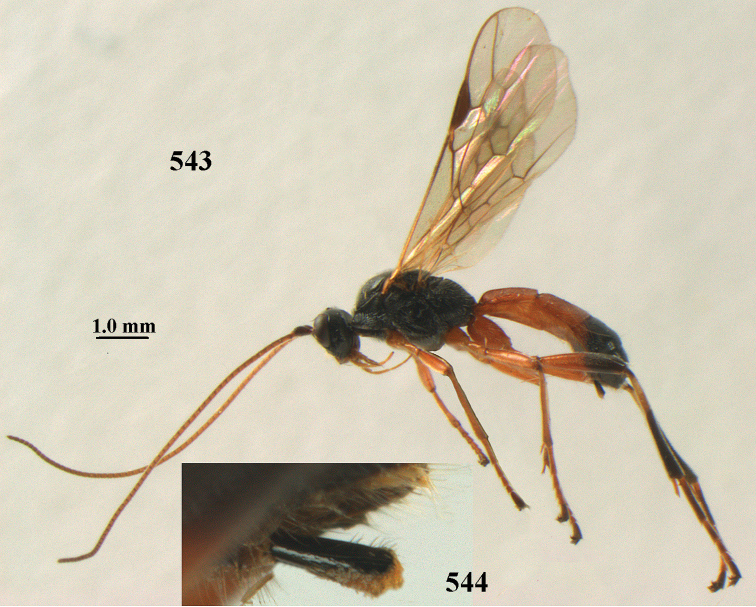
*Aleiodes pallidicornis* (Herrich-Schäffer), ♀, neotype **543** habitus lateral **544** ovipositor sheath lateral.

**Figures 545–557.**
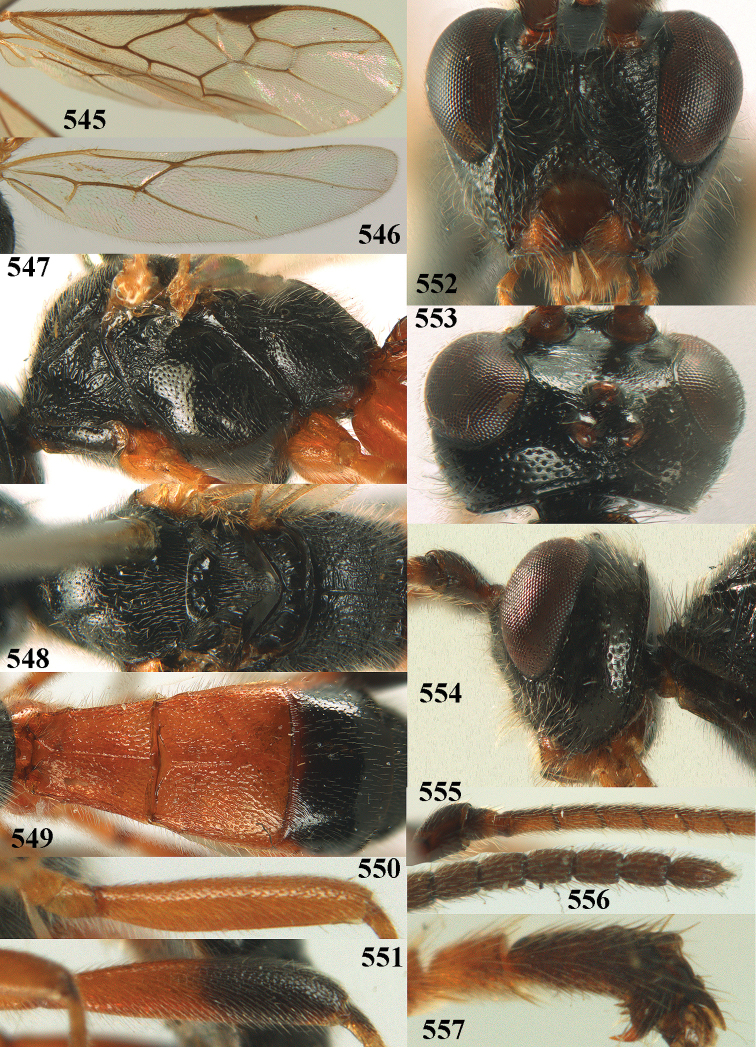
*Aleiodes pallidicornis* (Herrich-Schäffer), ♀, neotype **545** fore wing **546** hind wing **547** mesosoma lateral **548** mesosoma dorsal **549** 1^st^–3^rd^ metasomal tergites dorsal **550** fore femur lateral **551** hind femur lateral **552** head anterior **553** head dorsal **554** head lateral **555** base of antenna **556** apex of antenna **557** inner hind tarsal claw.

Maximum width of hypoclypeal depression approx. 0.3 × minimum width of face (Fig. 552); OOL of ♀ approx. as long as diameter of posterior ocellus and remotely punctate with interspaces superficially granulate (Fig. 553); ventral margin of clypeus thick and not protruding in lateral view (Fig. 554); mesoscutal lobes and vertex very finely and densely granulate, with satin sheen; precoxal area smooth medially, but sometimes some rugae below it; vein 1-CU1 0.4–0.6 × vein 2-CU1 and equally slender (Fig. 545); tarsal claws with distinct dark brown pecten (Fig. 557); hind femur and basitarsus slender (Fig. 543); basal quarter of 3^rd^ tergite largely finely striate; at least basal half of 4^th^–6^th^ tergites of ♂ usually with long dense setosity; head and pronotum black; both tegula and humeral plate equally yellowish; base of hind tibia with narrow dark brown band; hind femur and tibia at least partly black or dark brown; 2^nd^ tergite yellowish or reddish.

**Description**. Neotype, ♀, length of fore wing 5.9 mm, of body 6.6 mm.

***Head.*** Antennal segments of ♀ 54, length of antenna 1.3 × fore wing, its subapical segments rather robust (Fig. 556); frons largely superficially granulate, anteriorly with some weak striae; OOL 1.4 × diameter of posterior ocellus, and punctate, interspaces granulate; vertex spaced punctate, shiny; clypeus densely and coarsely punctate, with granulate interspaces; ventral margin of clypeus thick and not protruding forwards (Fig. 554); width of hypoclypeal depression 0.5 × minimum width of face (Fig. 552); length of eye 2.1 × temple in dorsal view (Fig. 553); vertex behind stemmaticum granulate with some transverse rugae; clypeus near lower level of eyes; length of malar space 0.4 × length of eye in lateral view.

***Mesosoma.*** Mesoscutal lobes finely punctate with largely granulate interspaces, with satin sheen; precoxal area of mesopleuron distinctly remotely punctate, interspaces larger than punctures; metapleuron densely punctate-granulate; metanotum with median carina; scutellum punctate-granulate; propodeum evenly convex and coarsely rugose, its medio-longitudinal carina complete.

***Wings.*** Fore wing: r 0.4 × 3-SR (Fig. 545); 1-CU1 horizontal, 0.5 × 2-CU1; r-m 0.6 × 3-SR; 2^nd^ submarginal cell medium-sized (Fig. 545); cu-a vertical, straight; 1-M nearly straight posteriorly; 1-SR wide; surroundings of M+CU1, 1-M and 1-CU1 evenly setose. Hind wing: marginal cell gradually widened, its apical width 2.6 × width at level of hamuli (Fig. 546); 2-SC+R short and longitudinal; m-cu present basally; M+CU:1-M = 15:14; 1r-m 0.6 × 1-M.

***Legs.*** Tarsal claws with rather small dark brownish pecten, absent near apical tooth (Fig. 557); hind coxa largely densely and finely punctate; hind trochantellus rather robust; length of hind femur and basitarsus 5.0 and 7.5 × their width, respectively; length of inner hind spur 0.4 × hind basitarsus.

***Metasoma.*** First tergite rather flattened, as long as wide apically; 1^st^ and 2^nd^ tergites with medio-longitudinal carina and coarsely irregularly rugose, but posteriorly 2^nd^ tergite largely smooth and no median carina; medio-basal area of 2^nd^ tergite triangular and rather large (Fig. 549); 2^nd^ suture rather deep and finely crenulate; basal half of 3^rd^ tergite smooth (except for punctuation) and shiny as remainder of metasoma; 4^th^ and apical half of 3^rd^ tergite without sharp lateral crease; ovipositor sheath moderately wide, with long setae and apically truncate (Fig. 544).

***Colour.*** Black; hind tarsus largely infuscate, but 3^rd^ and 4^th^ segments paler than other segments; apices of fore and middle tibiae slightly infuscate, base of middle and hind tibiae and telotarsi dark brown; apical two-fifths of hind femur and hind tibia (except a pale yellowish band subbasally) black; remainder of legs, 1^st^ and 2^nd^ tergites, and 3^rd^ tergite antero-laterally orange brown; palpi and tegulae brownish yellow; most of veins and pterostigma dark brown; wing membrane subhyaline.

***Variation.*** Antennal segments: ♀ 49(2), 50(2), 51(2), 52(6), 53(6), 54(1), 56(1); ♂ 50(1), 51(3), 52(1), 53(2), 54(2), 55(1), 56(3). On average males have ca two more antennal segments than females. Males are similar but have a large dark brown patch on 1^st^ tergite, hind tarsus largely dark brown and apical tergites type 3, positioned rather posteriorly, setae long and fringe not observed.

##### Distribution.

Austria, *Belarus, *British Isles (Scotland), Bulgaria, Hungary, *Iran, *Italy, *Montenegro, *Netherlands, North Korea, *Romania, Russia, *Slovakia, Switzerland, *Turkey.

##### Notes.

The type of *Rogas pallidicornis* Herrich-Schäffer, 1838, has been lost. Traditionally, it has been considered to belong to *Aleiodes ductor* (Thunberg, 1822), but the latter species is a synonym (see under *A. unipunctator*). The inadequate original description indicates that the 2^nd^ tergite has diverging rugae, which excludes part of *A. ductor* auctt. Female specimens with yellowish or brownish palpi, basal half of the antenna yellowish and blackish hind tibia (except its pale yellowish base) fit well the original description of *A. pallidicornis*.

#### Aleiodes pallidistigmus

Taxon classificationAnimaliaHymenopteraBraconidae

(Telenga, 1941)

2DDCBC74-9D5C-5AA5-9C5B-4B625E197404

Figs 558–559 , 560–572

Rhogas (Rhogas) pallidistigmus Telenga, 1941: xii, 143, 177 (but also as *palidistigma* (p. 409) and pallidistigma (p. 420)) [examined].
Rogas pallidistigmus ; Shenefelt 1975: 1241.
Aleiodes (Neorhogas) pallidistigmus ; Papp 1985b: 348.
Aleiodes (Chelonorhogas) pallidistigmus ; Chen and He 1997: 41; Fortier and Shaw 1999: 228; Belokobylskij 2000: 47; Ku et al. 2001: 237.
Aleiodes pallidistigma ; He et al., 2000: 667.
Rogas heterostigma Stelfox, 1953: 149; Shenefelt 1975: 1233 [examined]. Syn. nov.
Aleiodes (Neorhogas) heterostigma ; Papp 1985a: 143, 146–147, 153, 1991a: 95.
Aleiodes heterostigma ; O’Connor et al. 1999: 91; Papp 2005: 177.

##### Type material.

Paratypes of *A. heterostigma*, 4 ♀ + 1 ♂ (ZJUH, USNM), “[**Ireland**], Rye Water, Co. KD, 5/9.vii.[19]42, AWS[telfox]”; 1 ♀ (RMNH), id., but 5.vii.1942; 1 ♀ (NMI), id., but 8.vii.1948; 1 ♂ (NMI), “[Ireland], Woodbrook, OC, 26.vi.[19]38”. Holotype of *A. pallidistigmus* (♀, ZISP) from Far East Russia (Primorsky Krai, Ussuri area, Vinogradovka, 10.viii.1929, Kiritshenko).

##### Additional material.

1 ♀ (RMNH), “**Belgium**: Liège, Mt. Rigi, 650 m, 2.viii.1986, at light, C. v. Achterberg, RMNH”; 1 ♀ (MTMA), “Dania [= **Denmark**]: S-Jutland, Kragelund Mose, near Baekke, 11.viii.1973, [T.] Munk”; 1 ♀ (NMS), “**Wales**: Cereigion, Rhôs Rhydd, SN572738, Molinia bog, 30.vii.1987, NCC Welsh Peatland Survey, P. Holmes, NMSZ 1996.023”; 1 ♂ (NMS) “Wales; Ceredigion, Comin Esgair Maen, SN652649, *Equisetum* bog, 23.vii.1987, NCC Welsh Peatland Survey, P. Holmes, NMSZ 1996.023”; 1 ♂ (FMNH), “Fennia [= **Finland**]: Helsinki, 27.vii.1978, O. Ranin”; 1 ♀ (NMS) Far East Russia, Anismovka v. Shkotova/S Primorje reg., 11–13.viii.2003, leg. Osipov”; 2 ♀ (MRC) “Far East Russia, S. Primorje reg., Lazo distr. Valentin 17–18.vii.2003 leg. Osipov”: 1 ♀ (NMS) [Russian, Far East]; 2 ♀ (RMNH), “**China**: Jilin, Gomngzhuling, 43°5'N, 124°8'E, viii–ix.1983, Wang Chenghun”.

##### Molecular data.

None.

##### Biology.

Unknown for West Palaearctic populations. Specimens have been collected in (June)July-August in open boggy areas, certainly at least sometimes over limestone. Presumably univoltine, but we have not seen reared material from West Palaearctic and the overwintering mode is unclear.

##### Diagnosis.

Maximum width of hypoclypeal depression 0.4–0.5 × minimum width of face (Fig. 568); OOL of ♀ approx. 1.3 × as long as diameter of posterior ocellus and densely granulate (Fig. 569); penultimate segments rather slender and antenna 1.5 × as long as fore wing; ventral margin of clypeus thick, not protruding in lateral view; mesoscutal lobes finely granulate-punctulate and matt; precoxal area coarsely rugose; marginal cell of fore wing of ♀ ending rather close to wing apex (Fig. 560); vein 1-CU1 of fore wing 0.4–0.5 × as long as vein 2-CU1 (Fig. 560); hind tarsal claws rather robust and only brownish setose (Fig. 571); 3^rd^ tergite and basal half of 4^th^ tergite coriaceous and dull; labial palp yellowish brown or brown; basal half of hind tibia reddish or yellowish, slightly paler than basal half of hind femur, and its apex reddish or yellowish; 4^th^ and 5^th^ tergites black.

##### Description.

Redescribed ♀ paratype of *A. heterostigma* (RMNH) from Ireland (Rye Water). Length of fore wing 4.9 mm, of body 6.3 mm.

***Head.*** Antennal segments of ♀ 58, length of antenna 1.1 × fore wing, its subapical segments rather robust (Fig. 567); frons largely superficially granulate; OOL 1.8 × diameter of posterior ocellus, and superficially rugulose-granulate and shiny; vertex superficially rugulose-granulate, rather shiny; clypeus with some punctures; ventral margin of clypeus thick and not protruding forwards (Fig. 570); width of hypoclypeal depression 0.4 × minimum width of face (Fig. 568); length of eye 1.3 × temple in dorsal view (Fig. 569); vertex behind stemmaticum superficially granulate-rugulose; clypeus near lower level of eyes; length of malar space 0.4 × length of eye in lateral view.

***Mesosoma.*** Mesoscutal lobes densely and finely punctate-granulate, matt; precoxal area of mesopleuron coarsely rugose medially; remainder of mesopleuron coarsely punctate, with some rugae near speculum and interspaces superficially granulate; scutellum rather flat, punctulate-granulate and with weak lateral carinae; propodeum rather convex, shiny and coarsely rugose, medio-longitudinal carina distinct only on its anterior half.

***Wings.*** Fore wing: r 0.3 × 3-SR (Fig. 560); 1-CU1 horizontal, 0.45 × 2-CU1; r-m 0.7 × 3-SR; 2^nd^ submarginal cell short (Fig. 560); cu-a inclivous, straight; 1-M slightly curved posteriorly; surroundings of M+CU1, 1-M and 1-CU1 setose. Hind wing: marginal cell gradually widened with basal half rather narrow (Fig. 561), its apical width 2.6 × width at level of hamuli; 2-SC+R short longitudinal; m-cu weakly developed; M+CU:1-M = 35:26; 1r-m 0.5 × 1-M.

***Legs.*** Tarsal claws rather robust and only brownish setose (Fig. 572); hind coxa coarsely punctate, dorsally with oblique striae; hind trochantellus robust; length of hind femur and basitarsus 4.1 and 4.6 × their width, respectively; length of inner hind spur 0.5 × hind basitarsus.

***Metasoma.*** First tergite rather flattened, as long as wide apically; 1^st^ and 2^nd^ tergites and base of 3^rd^ tergite finely and irregularly longitudinally rugose, with medio-longitudinal carina weak; medio-basal area of 2^nd^ tergite triangular and short (Fig. 564); 2^nd^ suture rather deep and crenulate; apical half of 3^rd^ tergite punctate-granulate, remainder of metasoma smooth except for some superficial micro-sculpture; 4^th^ and apical half of 3^rd^ tergite without sharp lateral crease; ovipositor sheath wide, with long setae and apically truncate (Fig. 559).

***Colour.*** Black; telotarsi largely and basal quarter of antenna dark brown; palpi, tegulae and pterostigma pale yellow; remainder of legs, 1^st^ and 2^nd^ tergites, basal half of 3^rd^ tergite largely and pronotum orange brown; veins brown; wing membrane subhyaline.

***Variation.*** Pronotum anteriorly and basal half of antenna orange brown to dark brown, pterostigma is yellowish to largely (except base) rather dark brown; length of malar space 1.0–1.4 × basal width of mandible; OOL 0.7–1.8 × diameter of ocellus and metapleuron medially more or less punctate, rugulose-coriaceous or rugose. Antennal segments: ♀ 54(1), 58(1), 59(4), 60(2), 62(1), 64(1); ♂ 59(1), 60(1), 62(1). Apical tergites of ♂ type 1, fringe absent.

##### Distribution.

*Belgium, British Isles (Ireland, Wales), China, *Denmark, *Finland, Russia (Far East).

##### New synonymy.

We tried to separate the East Palaearctic *A. pallidistigmus* from the West Palaearctic *A. heterostigma*, but efforts were in vain. The differences such as the colour of the basal half of the antenna (dark brown in *A. heterostigma* and usually yellowish or brown in *A. pallidistigmus*), the eyes and ocelli often smaller, OOL 1.1–1.8 × diameter of ocellus (0.7–1.4 ×), malar space 1.2–1.3 × basal width of mandible (1.0–1.4 ×) and metapleuron with a shiny and more or less punctate area (less shiny and rugulose-coriaceous or rugose) are too variable to justify separation of *A. heterostigma*. Therefore, we synonymise *A. heterostigma* with *A. pallidistigmus* (syn. nov.).

**Figures 558, 559.**
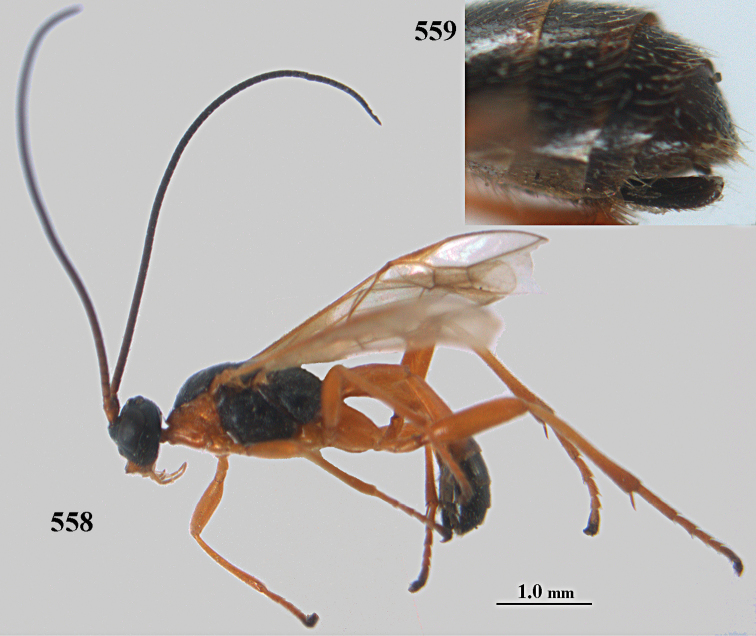
*Aleiodes pallidistigmus* (Telenga), ♀, Denmark, Kragelund Mose **558** habitus lateral **559** ovipositor sheath lateral.

**Figures 560–572.**
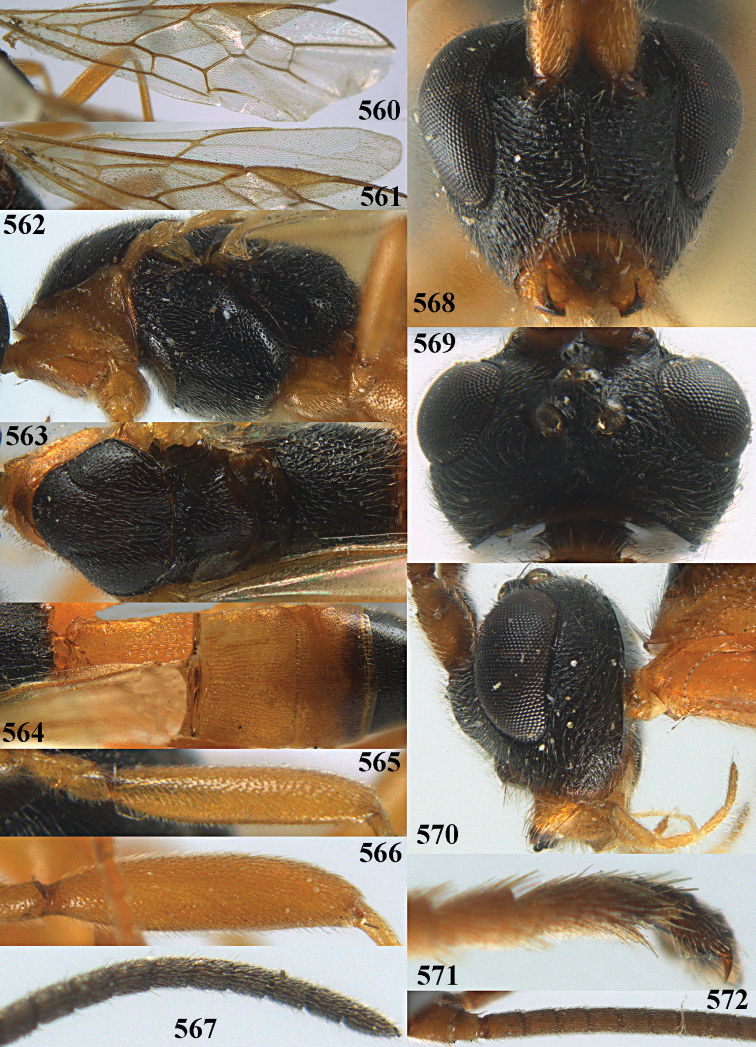
*Aleiodes pallidistigmus* (Telenga), ♀, Denmark, Kragelund Mose **560** fore wing **561** hind wing **562** mesosoma lateral **563** mesosoma dorsal **564** 1^st^–3^rd^ metasomal tergites dorsal **565** fore femur lateral **566** hind femur lateral **567** apex of antenna **568** head anterior **569** head dorsal **570** head lateral **571** outer hind tarsal claw **572** base of antenna.

#### Aleiodes periscelis

Taxon classificationAnimaliaHymenopteraBraconidae

(Reinhard, 1863)

1A94464D-518E-5653-8810-0820C4462E09

Figs 573–576 , 577–589 , 590 , 591–603

Rogas periscelis Reinhard, 1863: 254; Shenefelt 1975: 1242; Kotenko 1992: 96 [examined].
Rhogas (Rhogas) periscelis var. charkowensis Kokujev, 1898: 297.
Rhogas (Rhogas) periscelis var. charkoviensis [sic!]; Telenga 1941: 163, 164.
Rogas (Rogas) periscelis ; Tobias 1976: 86, 1986: 81 (transl. 133); Papp 1983: 330.
Aleiodes (Neorhogas) periscelis ; Papp 1985a: 161 (lectotype designation), 1985b: 348; 1991a: 79, 1991c: 641, 1994: 307.
Aleiodes (Chelonorhogas) periscelis ; Belokobylskij 2000: 36.
Aleiodes periscelis ; Fortier and Shaw 1999: 230; Belokobylskij and Taeger 2001: 115; Belokobylskij et al. 2003: 400 (excluded from German checklist); Papp, 2005: 177.
Rhogas jaroslawensis Kokujev, 1898: 302. Syn. nov.
Rhogas (Rhogas) jaroslavensis ; Telenga 1941: 176, 408 (invalid emendation).
Rogas jaroslawensis ; Shenefelt 1975: 1235.
Rogas (Rogas) jaroslavensis ; Tobias 1976: 85.
Rogas (Rogas) jaroslawensis ; Tobias 1986: 81 (transl.: 133; as synonym of A. rufipes (Thomson)).
Aleiodes (Neorhogas) jaroslawensis ; Papp 1985a: 153, 1991a: 80.
Aleiodes jaroslawensis ; Papp 2005: 177 (as valid species).

##### Type material.

Lectotype of *R. periscelis*, ♂ (ZMB), “[**Austria**:] Neusiedler See”, “Type”, “Coll. H. Rhd.”, “26696”, “*periscelis* Gir. [= from Giraud]”, “Lectotypus *Rogas periscelis* Rhd., 1863, ♂, Papp, 1982”, “*Aleiodes periscelis* Rhd., ♂, det. Papp J., 1983”; 2 ♂ paralectotypes (MNHN), one with “[Austria:] Prater, Mai”, “ex coll. Giraud”. Holotype of *R. jaroslawensis*, ♀ (ZISP; examined photos made by K. Samartsev), “[S. **Russia**:] Berditsino [Yaroslavskiy rayon, 57.454N, 40.108E], 22.vi.1892, A.M. Yakovlev, 1909”, “*Rh. jaroslawensis* Kokw., No. 1909” and with a round golden label.

##### Additional material.

Czech Republic, Germany, Hungary, Russia. Specimens in ALC, BZL, MTMA, NMS, SDEI, ZISP, ZMB, ZSSM.

##### Molecular data.

None.

##### Biology.

Unknown but presumably univoltine. Specimens of both sexes collected in April and May suggest that the winter is passed in the mummy. We have not seen reared material, but several Hungarian specimens appear to have been collected in *Quercus*-dominated woodland, but without indication of any association with *Quercus* as such.

##### Diagnosis.

**Figures 573–576.**
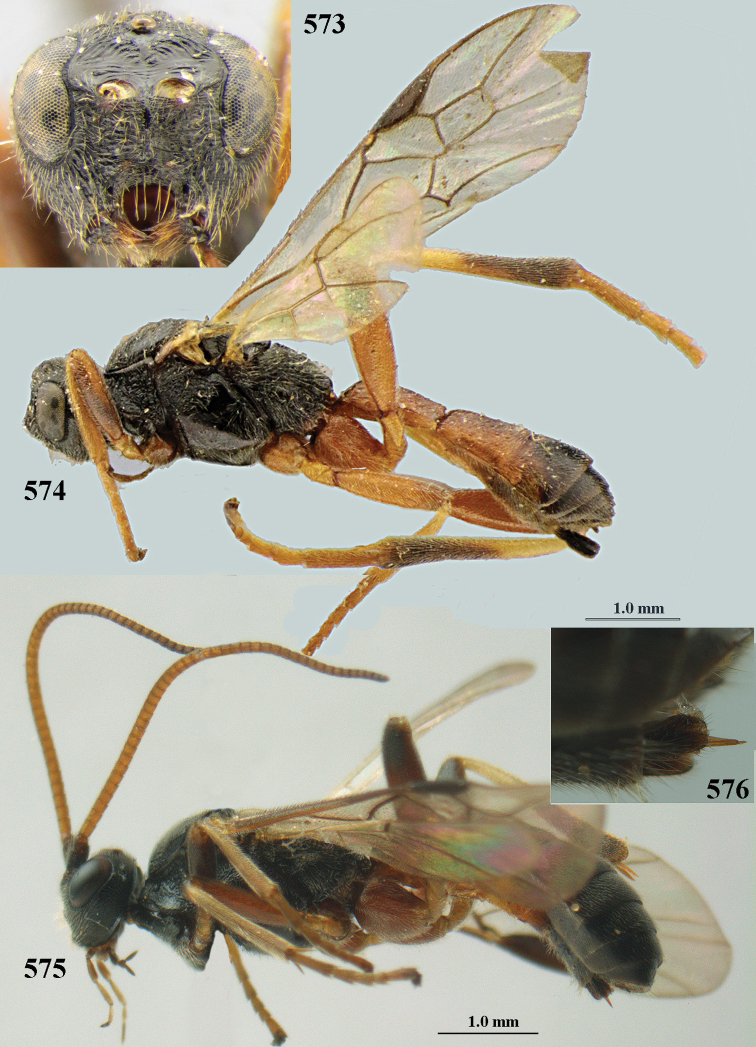
*Aleiodes periscelis* (Reinhard), ♀, 573, 574 Russia (holotype *R. jaroslawensis* Kokujev) and 575, 576 Czech Republic, Pisek **573** head anterior **574, 575** habitus lateral **576** ovipositor sheath lateral. Photographs 573, 574 by K. Samartsev.

Maximum width of hypoclypeal depression approx. 0.4 × minimum width of face (Figs 573, 584); OOL of ♀ 1.5 × as long as diameter of posterior ocellus (Fig. 585; of ♂ 1.4 ×; Fig. 599), rugulose or rugose and with satin sheen; ventral margin of clypeus thick and not protruding anteriorly (Fig. 586); mesoscutum remotely punctulate and with satin sheen, interspaces of lateral lobes largely smooth, of middle lobe superficially coriaceous; area of precoxal sulcus smooth and shiny; length of vein 1-CU1 of fore wing 0.3–0.4 × vein 2-CU1 and 0.5 × vein m-cu; vein 2-SC+R of hind wing subquadrate; hind basitarsus robust; head (including basal half of mandible) black; antenna of ♀ (except scapus and pedicellus, and apically darkened) brownish yellow; apex of hind femur usually largely black dorsally; basal half of hind tibia (largely) pale yellowish; fore coxa dark brown; 2^nd^ tergite of ♀ orange or dark reddish brown, of ♂ largely black; 4^th^–6^th^ tergites of males flat, and with long dense setosity (Fig. 590).

##### Description.

Redescribed ♀ (BZL), Czech Republic (Pisek); length of fore wing 5.8 mm, of body 8.1 mm.

***Head.*** Antennal segments 45 (holotype ♀ of *A. jaroslawensis*: 42), length of antenna approx. as long as fore wing, its subbasal and subapical segments robust (Figs 587, 588); frons largely smooth anteriorly and with curved rugae posteriorly; OOL 1.5 × diameter of posterior ocellus, rugulose and shiny; vertex finely rugose and with satin sheen; clypeus slightly convex and mainly transversely aciculate; ventral margin of clypeus thick and not protruding anteriorly (Fig. 586); width of hypoclypeal depression 0.4 × minimum width of face (Fig. 584); length of eye 1.5 × temple in dorsal view (Fig. 585); vertex behind stemmaticum rugulose; clypeus below lower level of eyes; length of malar space 0.45 × length of eye in lateral view and 1.3 × basal width of mandible.

***Mesosoma.*** Mesoscutum remotely punctulate and with satin sheen, interspaces of lateral lobes largely smooth, and of middle lobe superficially coriaceous; scutellum superficially punctate, laterally rugose; precoxal area of mesopleuron smooth and shiny; metapleuron largely densely punctate, but ventrally coarsely rugose; metanotum with distinct median carina anteriorly; propodeum rather flat and coarsely vermiculate rugose, medio-longitudinal carina complete, and slightly tuberculate laterally.

**Figures 577–589.**
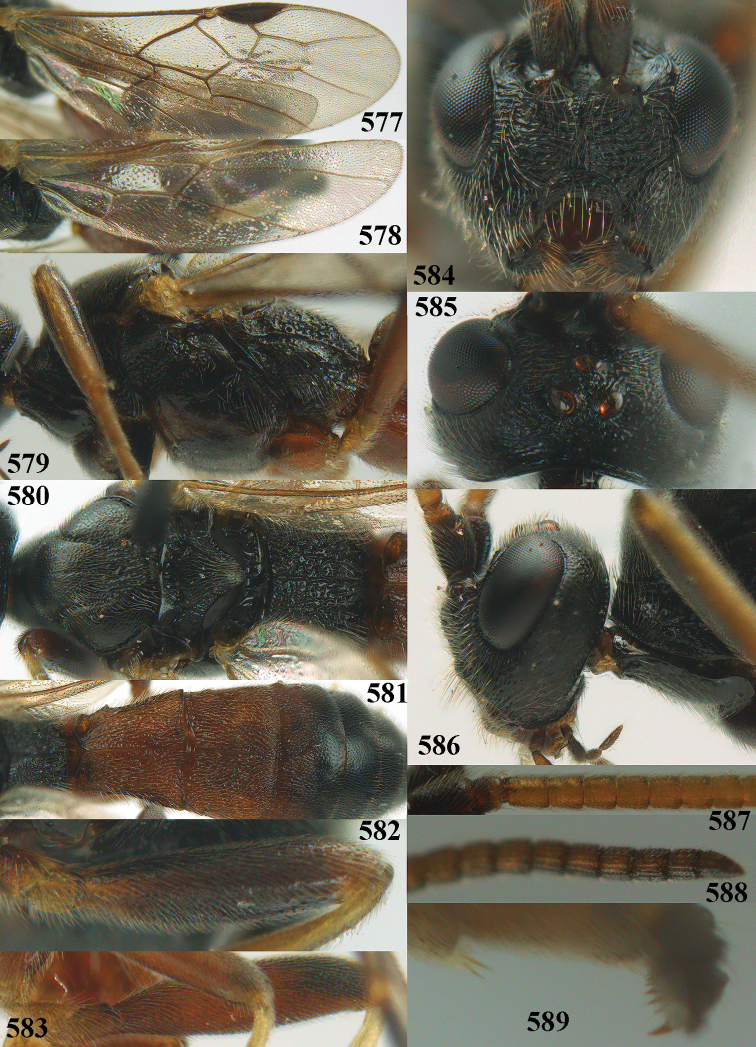
*Aleiodes periscelis* (Reinhard), ♀, Czech Republic, Pisek **577** fore wing **578** hind wing **579** mesosoma lateral **580** mesosoma dorsal **581** metasoma dorsal **582** fore femur lateral **583** hind femur lateral **584** head anterior **585** head dorsal **586** head lateral **587** base of antenna **588** apex of antenna **589** outer middle tarsal claw.

***Wings.*** Fore wing: r 0.3 × 3-SR (Fig. 577); 1-CU1 horizontal, 0.3 × as long as 2-CU1; r-m 0.6 × 3-SR; 2^nd^ submarginal cell medium-sized (Fig. 577); cu-a vertical, straight; 1-M nearly straight posteriorly and subparallel; 1-SR slender; surroundings of M+CU1, 1-M and 1-CU1 densely setose. Hind wing: marginal cell gradually widened (but less so in its basal third) and apical width 2.2 × width at level of hamuli (Fig. 578); 2-SC+R quadrate; m-cu short; M+CU:1-M = 40:33; 1r-m 0.5 × 1-M.

***Legs.*** Tarsal claws with conspicuous and medium-sized brownish pecten, remaining removed from tarsal tooth (Fig. 589); hind coxa largely coriaceous-punctate, but dorsal besides smooth depression rugose; hind trochantellus rather robust; length of hind femur 3.7 × its width.

***Metasoma.*** First tergite rather flat posteriorly, wide subbasally and 0.9 × longer than wide apically; 1^st^ and 2^nd^ tergites with coarse medio-longitudinal carina and coarsely longitudinally rugose, but posterior quarter of 2^nd^ tergite rather finely rugose; medio-basal area of 2^nd^ tergite triangular and wide (Fig. 581); 2^nd^ suture moderately deep, finely crenulate and narrow; basal two-thirds of 3^rd^ tergite finely longitudinally rugose, remainder of metasoma superficially micro-sculptured and with satin sheen; 4^th^ and apical half of 3^rd^ tergites without sharp lateral crease; ovipositor sheath wide, with rather long setae and apically truncate (Figs 574, 576).

***Colour.*** Black; palpi dark brown basally and remainder pale brown; antenna (except dark brown scapus and pedicellus), tegulae (but anteriorly dark brown), middle and hind trochanters and trochantelli brownish yellow; fore coxa, trochanter and femur dark brown; basal 0.4 of hind tibia ivory and remainder black; remainder of legs (but hind femur with a blackish patch dorso-apically), 1^st^ and 2^nd^ tergites and basal two thirds of 3^rd^ tergite, largely dark reddish brown; pterostigma dark brown; veins mainly yellowish brown, but medially brown (Figs 577, 578); wing membrane subhyaline.

**Figure 590.**
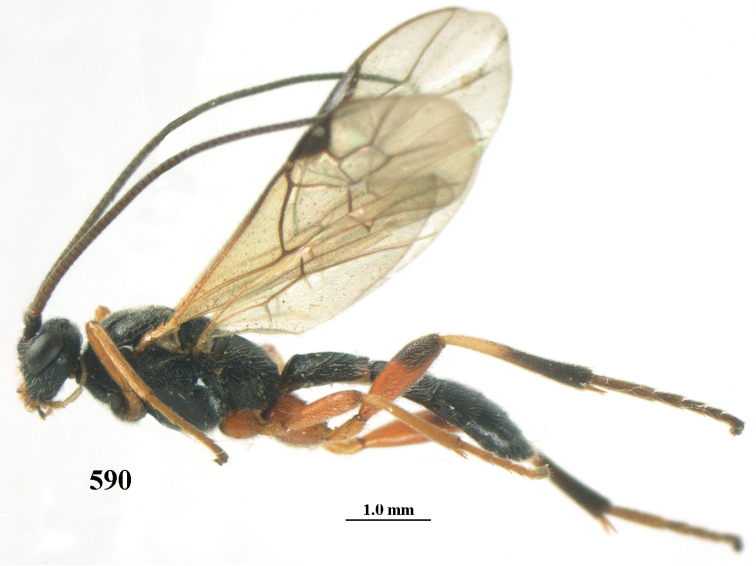
*Aleiodes periscelis* (Reinhard), ♂, Russia, Serpukhov, habitus lateral.

***Variation.*** Holotype of *A. jaroslawensis* has apex of hind femur yellowish brown (Fig. 574). Antennal segments: ♀ 42(1), 45(1); ♂ 50(1), 53(1), 54(2), 56(1). Males appear to have ca ten more antennal segments than females. Male has apical tergites type 1, setae moderately long and fringe not observed, probably absent (Fig. 590); antenna rather dark brown, but scapus largely blackish brown and antennal segments slightly slenderer than of female and 1.1–1.2 × as long as fore wing; metasoma black, but extreme apex of 1^st^ tergite yellowish brown; OOL approx. 1.4 × width of posterior ocellus.

##### Distribution.

Austria, Czech Republic, Germany, Hungary, Russia.

##### New synonymy.

The ♀ holotype of *Rhogas jaroslawensis* lacks the antennae, but according to the original description the antenna was 42-segmented, distinctly shorter than the body, reddish brown, except for the darkened apex and the black scapus. This and the other characters still visible agree well with our interpretation of *A. periscelis* (except that the hind femur is yellowish brown apically); therefore, we synonymise *R. jaroslawensis* with *A. periscelis* (syn. nov.).

**Figures 591–603.**
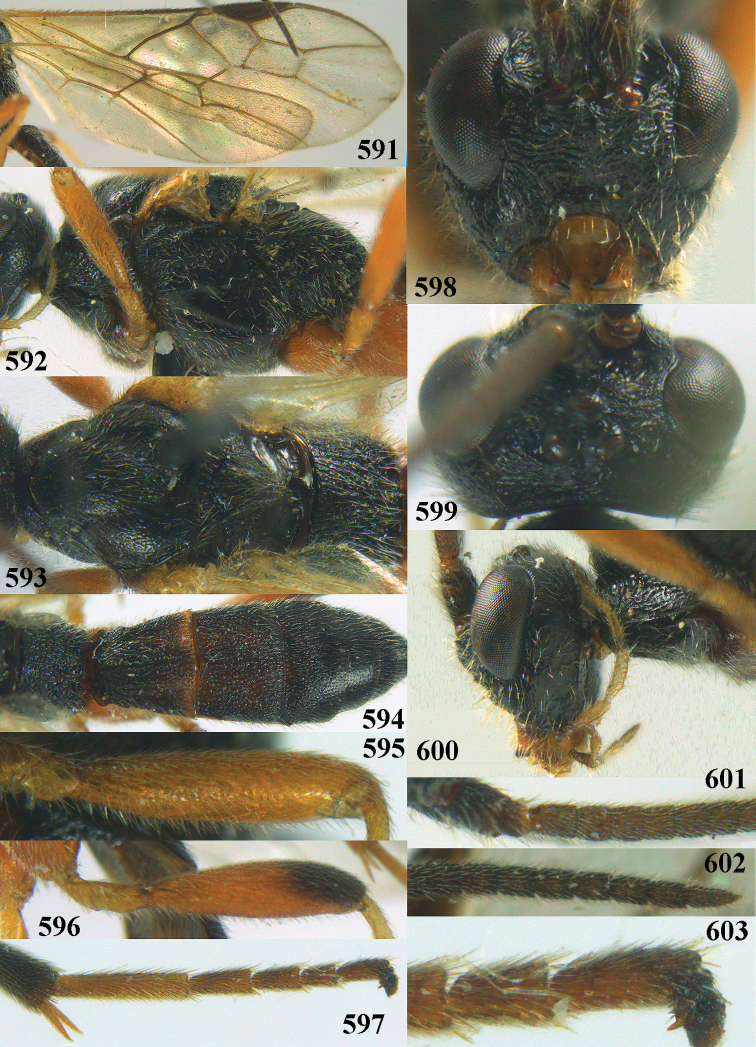
*Aleiodes periscelis* (Reinhard), ♂, Russia, Serpukhov **591** wings **592** mesosoma lateral **593** mesosoma dorsal **594** 1^st^ –5^th^ metasomal tergites dorsal **595** fore femur lateral **596** hind femur lateral **597** hind tarsus lateral **598** head anterior **599** head dorsal **600** head lateral **601** base of antenna **602** apex of antenna **603** inner hind tarsal claw.

#### Aleiodes pulchripes

Taxon classificationAnimaliaHymenopteraBraconidae

Wesmael, 1838

252D41F9-7D7C-5A97-BB69-6CD6F339C89E

Figs 604–607 , 608–621 , 622–626

Aleiodes pulchripes Wesmael. 1838: 102; Čapek and Lukás 1989: 31; Papp 1991a: 73, 2005: 177; Fortier and Shaw 1999: 224; O’Connor et al. 1999: 92; Belokobylskij et al. 2003: 398 [examined].
Rogas pulchripes ; Shenefelt 1975: 1245.
Rogas (Rogas) pulchripes ; Tobias 1976: 83, 1986: 78 (transl.: 128).
Aleiodes (Neorhogas) pulchripes ; Papp 1985a: 149, 153, 161, 1991a: 73.
Aleiodes pulchricornis Kolubajiv, 1962: 27; Shenefelt 1975: 1245 (invalid emendation; not A. pulchricornis (Szépligeti, 1902)); Papp 2005: 177 (as synonym of A. pulchripes).

##### Type material.

Holotype of *A. pulchripes*, ♂ (KBIN), “*A. pulchripes* ♂ mihi”, “*A. pulchripes* mihi, dét. C. Wesmael”, “Coll. Wesmael”, “**Belgique**, Charleroi/ teste Papp J., 1983”, “Holotypus”, “*Aleiodes pulchripes* Wesm., 1838, ♂, Papp, 1983”.

##### Additional material.

Austria, British Isles (England: V.C. 59; Isle of Man: V.C. 71: Ireland: V.C. H21), Czech Republic, Finland, Germany, Hungary, Netherlands (GE: Vierhouten; ZH: Leiden; NH: Amsterdam; Sloten), Russia, Sweden. Specimens in ZJUH, CNC, IKC, KBIN, MSC, MTMA, NMS, NRS, RMNH, USNM, UWIM, ZISP, ZSSM.

##### Molecular data.

MRS847 (Sweden), MRS873 (Sweden).

##### Biology.

Collected in (June)July and August. Univoltine, overwintering in an exposed mummy. Reared from the following arboreal acronictine Noctuidae: *Acronicta aceris* (Linnaeus) (2 [CNC, MSC], Austria, Germany; J. Schwarz), *Acronicta psi* (Linnaeus) (22 [1 NRS, 2 ZISP]; M.R. Shaw), *Acronicta leporina* (Linnaeus) (1 [IKC], Finland; M.J. Pellinen), *Acronicta tridens* (Dennis & Schiffermüller) (4:2; M.R. Shaw), *Acronicta psi* or *tridens* (2), indet. Acronictinae (1). A quantitative account of rearing this species at its only known English site, comprising old hedges rich in *Sorbus aucuparia* bordering a largely reclaimed peat bog, is given by Shaw (1979). Experimental rearings were unfortunately limited to unobserved exposures of multiple hosts in closed boxes; extremely hot weather marred the results, but from one box containing 15 of each of *Subacronicta megacephala* (Dennis & Schiffermüller) and *A. tridens*, the surviving 13 *S. megacephala* were dissected after three days of exposure and contained no hosts, while at least eight of the *A. tridens* contained parasitoids (two found by dissection + six mummies formed; of the other seven, one contained no parasitoid on dissection + six resulted in moths). This suggests that *S. megacephala* is outside the host range. Similar but less well quantified experiments also excluded the low-feeding *Acronicta rumicis* (Linnaeus) and the arboreal lymantriine Erebidae*Euproctis similis* (Fuessly). It is worth adding that the rather frequent citation of lymantriine hosts in the literature can undoubtedly be explained by misidentification of the setose and rather colourful larvae of most species of arboreal acronictine noctuids. The mummy is dark grey in colour, leaving only little evidence of the patches of bright colour that had been a feature of the host larva. It forms in the caudal part of the host, the anterior segments of which strongly contract towards the extensive point of attachment, and the cocoon occupies approx. 4^th^–7^th^ abdominal segments. As mummification approaches, the host aligns itself on a narrow aerial twig to which the mummy becomes ventrally adpressed, thus leaving a weakly arched dorsal profile bearing a strong resemblance to an overwintering lateral bud (e.g., of *Sorbus aucuparia*: Fig. 605).

##### Diagnosis.

Maximum width of hypoclypeal depression 0.3–0.4 × minimum width of face (Fig. 616); OOL distinctly less than diameter of posterior ocellus, largely smooth but micro-sculptured near eyes; ventral margin of clypeus thick and not protruding in lateral view (Fig. 618); mesoscutal lobes coriaceous; mesopleuron (including precoxal sulcus area) nearly or completely smooth; propodeum with pair of crest-like protuberances laterally; vein 1-CU1 of fore wing much shorter than vein 2-CU1; basal half of marginal cell of hind wing parallel-sided (Fig. 609); tarsal claws with large dark brown pecten up to apical tooth of claw (Fig. 621); hind spurs dark brown; hind tibial spurs of ♂ obtuse apically (Fig. 624); head black; pterostigma pale yellowish or light brown; mesopleuron, mesosternum and scutellum brownish yellow; apex of hind femur yellowish or reddish; basal half of hind tibia pale yellowish.

##### Description.

Redescribed ♀ (RMNH) from England (Chat Moss). Length of fore wing 5.3 mm, of body 6.5 mm.

***Head.*** Antennal segments of ♀ 56, length of antenna 1.3 × fore wing, its subapical segments slender (Fig. 620); frons largely smooth; OOL 0.3 × diameter of posterior ocellus, and smooth near ocelli, but micro-sculptured near eye, shiny; vertex largely smooth, with few punctures, shiny; face crest-like protruding medio-dorsally; clypeus densely punctate; ventral margin of clypeus thick and not protruding forwards (Fig. 618); width of hypoclypeal depression 0.35 × minimum width of face (Fig. 616); length of eye 5.6 × temple in dorsal view (Fig. 617); vertex behind stemmaticum mainly smooth but partly rugulose; clypeus above lower level of eyes; length of malar space 0.15 × length of eye in lateral view.

***Mesosoma.*** Mesoscutal lobes coriaceous, rather shiny; precoxal area of mesopleuron smooth as most of mesopleuron; metanotum with medio-longitudinal carina anteriorly; scutellum finely punctate, interspaces smooth, but posteriorly coriaceous; propodeum rather flat medially and rather remote rugose, medio-longitudinal carina nearly complete, and with slightly protruding carinae laterally.

***Wings.*** Fore wing: r 0.4 × 3-SR (Fig. 608); 1-CU1 slightly oblique, 0.4 × 2-CU1; r-m 0.6 × 3-SR; 2^nd^ submarginal cell rather robust (Fig. 608); cu-a inclivous, straight; 1-M nearly straight posteriorly; 1-SR wide; surroundings of M+CU1, 1-M and 1-CU1 sparsely setose. Hind wing: basal half of marginal cell parallel-sided, apical half linearly widened, its apical width twice width at level of hamuli (Fig. 609); 2-SC+R subquadrate; m-cu absent; M+CU:1-M = 31:26; 1r-m 0.7 × 1-M and 1-M oblique.

***Legs.*** Tarsal claws with conspicuous and robust dark brown pecten up to apical tooth of claw (Fig. 621); hind coxa dorsally largely smooth and remainder remotely punctate; hind trochantellus robust; length of hind femur and basitarsus 4.3 and 5.0 × their width, respectively; length of inner hind spur 0.45 × hind basitarsus.

***Metasoma.*** First tergite evenly convex medially, 0.9 × longer than wide apically, wider than base of 2^nd^ tergite; 1^st^ and 2^nd^ tergites with medio-longitudinal carina and coarsely irregularly sublongitudinally rugose; medio-basal area of 2^nd^ tergite triangular and rather large (Fig. 612); 2^nd^ suture deep and coarsely crenulate; basal half of 3^rd^ tergite rugulose, remainder of metasoma largely smooth; 4^th^ and apical half of 3^rd^ tergite without sharp lateral crease; ovipositor sheath wide, with long setae and apically truncate (Fig. 606).

***Colour.*** Blackish or dark brown; telotarsi, apical 0.4 of hind tibia, hind tibial spurs and hind tarsus dark brown; remainder of hind tibia and palpi yellowish; remainder of legs, pterostigma and tegulae pale brownish yellow; veins brown; mesoscutum medio-dorsally, scutellum, metanotum, mesopleuron (except partly antero-dorsally), mesosternum and metapleuron orange yellow; wing membrane subhyaline.

***Variation.*** Scutellum largely finely punctate, coriaceous medio-posteriorly, but may be striate. Specimens from Sweden are appreciably darker than those from Britain. Vein m-cu of hind wing absent or faintly indicated. Antennal segments: ♀ 51(1), 52(2), 53(2), 54(3), 55(4), 56(6), 57(7), 58(7), 59(2), 60(1), 62(1); ♂ 49(1), 51(1), 53(2), 54(9), 55(6), 56(6), 57(2). Females have on average ca three more antennal segments than males. Males have obtuse hind tibial spurs and the tarsal pecten less developed than in females, propleuron and pronotum yellowish or blackish posteriorly; posterior half of mesoscutum largely yellowish or blackish; apical tergites type 2, somewhat sparse setose, glabrous stripe broad but with some setae directed into it and fringe rather weak (Figs 625, 626).

##### Distribution.

*Austria, British Isles (England, Isle of Man, Ireland), Czech Republic, Finland, Germany, Hungary, *Netherlands, Russia, Sweden.

**Figures 604–607.**
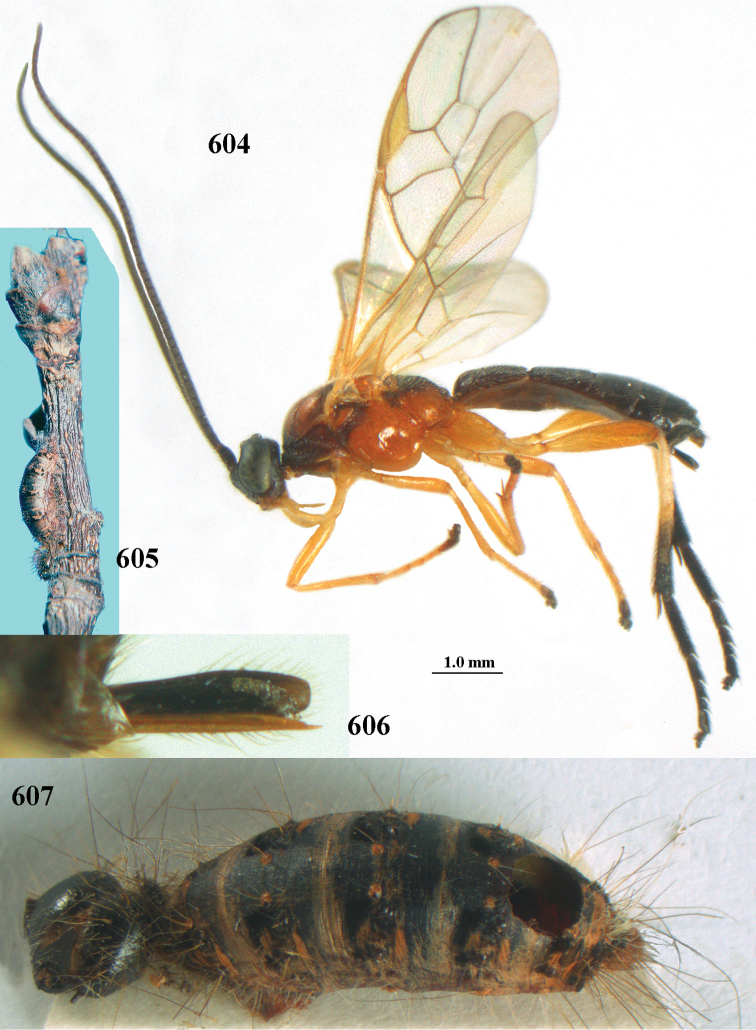
*Aleiodes pulchripes* Wesmael, ♀, England, Chat Moss **604** habitus lateral **605** mummy of *Acronicta psi* (Linnaeus) in winter **606** ovipositor sheath lateral, **607** mummy of *Acronicta psi* (Linnaeus) after emergence of parasitoid.

**Figures 608–621.**
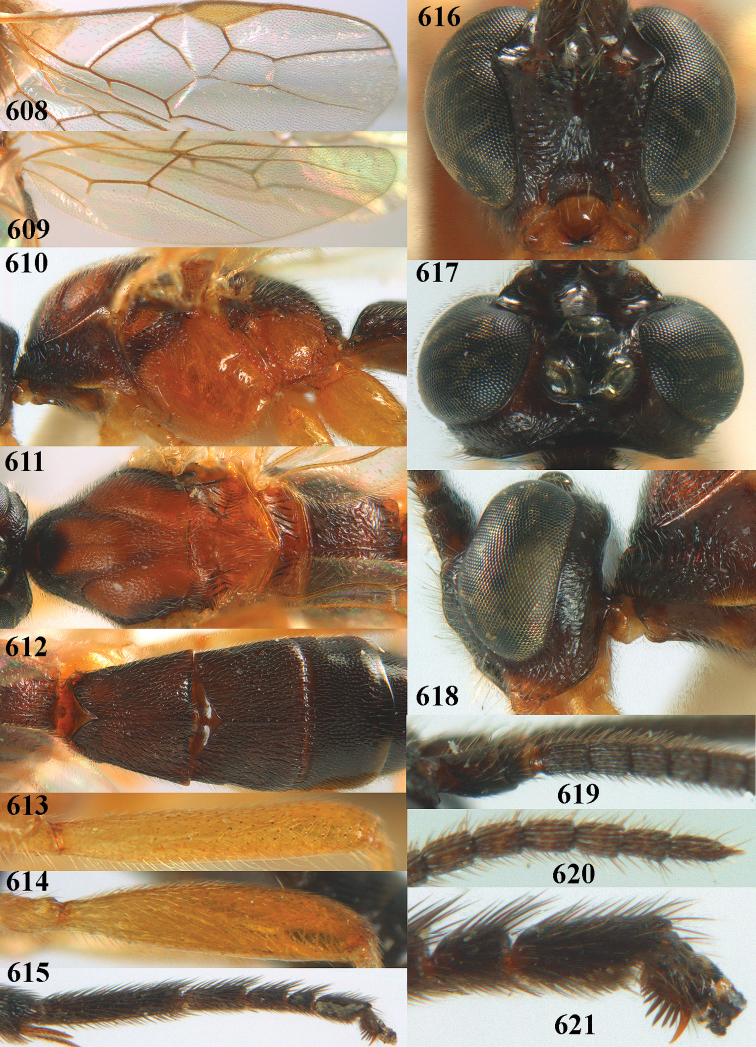
*Aleiodes pulchripes* Wesmael, ♀, England, Chat Moss **608** fore wing **609** hind wing **610** mesosoma lateral **611** mesosoma dorsal **612** 1^st^–3^rd^ metasomal tergites dorsal **613** fore femur lateral **614** hind femur lateral **615** hind tarsus lateral **616** head anterior **617** head dorsal **618** head lateral **619** base of antenna **620** apex of antenna **621** outer hind tarsal claw.

**Figures 622–626.**
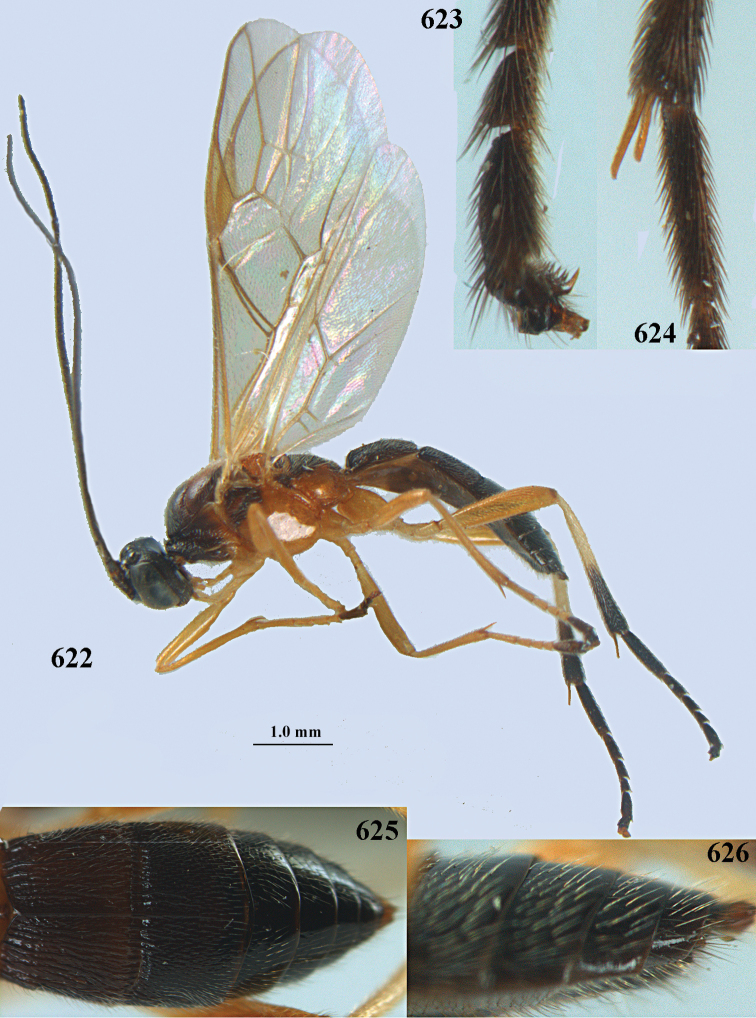
*Aleiodes pulchripes* Wesmael, ♂, England (ex *Acronicta* culture) **622** habitus lateral **623** outer hind claw lateral **624** hind tibial spurs and basitarsus lateral **625** metasoma dorsal **626** 4^th^–7^th^ metasomal tergites lateral.

#### Aleiodes quadrum

Taxon classificationAnimaliaHymenopteraBraconidae

(Tobias, 1976)

33A49D24-B0A2-5511-B3FE-383C9E346F4C

Figs 627–628 , 629–641 , 642 , 643–651

Rogas (Rogas) quadrum Tobias, 1976: 83, 221, 1986: 76 (transl.: 125).
Aleiodes (Neorhogas) quadrum ; Papp 1985a: 162, 1991a: 83.
Aleiodes quadrum ; Papp 2005: 177.
Rogas (Rogas) illustris Papp, 1977a: 112, 1985a: 162 (as synonym of A. quadrum), 1991a: 83 (id.), 2005: 176 (id.) [examined].

##### Type material.

Holotype of *A. illustris*, ♀ (MTMA), “Yugoslavia, [**Croatia**:] Kostrena, Rijeka, 12.viii.1966, Uremović”, “Holotypus ♀ *Rogas illustris* sp. n., Papp, J., 1977”, “Hym. Typ. No. 2378, Mus. Budapest”; paratype, ♀ (MTMA), “[**Hungary**], Hársbokorhegy, Nagykovacsi”, “1.viii.1952, Bajári”, “Paratypus ♀ *Rogas illustris* sp. n., Papp, J., 1977”, “Hym. Typ. No. 2380, Mus. Budapest”.

##### Additional material.

1 ♀ (NMS), “**France**: Ardèche, Accons, UV light, 24.vi.2013, M.R. Shaw”, “MRS Aleiodes DNA 796”; 1 ♀ (NMS), “France: Savoie, Queige, Le Villaret, 700m., 19.vi. 2019, C.W. Plant”; 1 ♀ (BZL), “**Turkey**, 15 km W Refahye, W of Erzincan, 1600 m, 7.vii.2000, M. Halada”; 1 ♀ (BZL), “**GRC [= Greece**], Westmakadonien, Florina, Aussichtsplatz SE Karies, 40°45'2"N, 21°10'39"E, 1080 m msl, 27.vi.2016, 2016/31, LF, H. u. R. Rausch”; 1 ♀ (MTMA), “[**North**] **Macedonia**, Skopje Prov., Mt. Vodno, 16.vii.1997, Gy. Rozner”; 1 ♀ (NMS), “N. Macedonia, Vardar river valley, above Demir Kapiya, N41°22'58", E22°11'45", 244m, 13.vii.2019 S. Beshkov & A. Nahirnic”; 1 ♂ (NMS), “**Bulgaria**: Haskovo, E. Rhodopes, SW Mezek, 450 m, MV light, 17.vii.2015, C.W. Plant”.

##### Molecular data.

MRS796 (France), additionally MRS824 (Bulgaria) likely to be a male of this species.

##### Biology.

Unknown. Collected in June–July, likely to be univoltine, but there is nothing to suggest how the winter is passed. We have not seen reared material, but the elongate and strongly apically compressed metasoma suggests that the host would be concealed, perhaps between spun leaves, in a leaf sheath, in a seed capsule or in some similar situation.

##### Diagnosis.

Maximum width of hypoclypeal depression approx. 0.6 × minimum width of face (Fig. 636); OOL of ♀ 0.6–0.7 × diameter of posterior ocellus and rugose; ventral margin of clypeus obtuse apically and clypeus hardly protruding anteriorly (Fig. 638); lobes of mesoscutum densely finely punctate, with interspaces approx. equal to diameter of punctures, smooth and shiny; precoxal area distinctly rugose, but posteriorly only punctate; vein cu-a of fore wing vertical; surroundings of veins M+CU1 and 1-+2-CU1 largely glabrous; vein 1-CU1 of fore wing approx. 0.8 × vein 2-CU1 and as long as m-cu (Fig. 629); surroundings of veins M+CU and 1-M of hind wing largely glabrous; hind tarsal claws with conspicuous dark brown pecten close to apical tooth (Fig. 635); 1^st^ tergite parallel-sided and longer than wide apically (Fig. 632); 2^nd^ tergite of ♀1.0–1.2 × as long as wide basally and black; head black; vein 1-M of fore wing dark brown; wing membrane slightly infuscate.

##### Redescription.

♀ (NMS) from France (Accons). Length of fore wing 6.9 mm, of body 9.6 mm.

***Head.*** Antennal segments of ♀ 53, antenna as long as fore wing, its subapical segments rather robust (Fig. 640); frons with curved striae but medially largely smooth; OOL 0.7 × diameter of posterior ocellus, finely rugose and shiny; vertex coarsely punctate but behind ocelli rugose, rather shiny; clypeus nearly flat and coarsely rugose-punctate; ventral margin of clypeus thick and hardly protruding anteriorly (Fig. 638); width of hypoclypeal depression 0.6 × minimum width of face (Fig. 636); length of eye 1.6 × temple in dorsal view (Fig. 637); clypeus near lower level of eyes; length of malar space 0.2 × length of eye in lateral view.

***Mesosoma.*** Mesoscutal lobes densely and finely punctate, with interspaces approx. equal to diameter of punctures, smooth and shiny; scutellum finely and densely punctate; precoxal sulcus area of mesopleuron distinctly rugose but posteriorly only punctate, remainder of mesopleuron distinctly but remotely punctate; metapleuron remotely punctate, but ventrally rugose; propodeum evenly convex and coarsely vermiculate-rugose and medio-longitudinal carina nearly complete.

***Wings.*** Fore wing: r 0.4 × 3-SR (Fig. 629); 1-CU1 horizontal, 0.8 × 2-CU1; r-m 0.6 × 3-SR; 2^nd^ submarginal cell medium-sized (Fig. 629); cu-a nearly vertical and straight; 1-M curved posteriorly; 1-SR rather slender; surroundings of M+CU1, 1-M and 1-CU1 largely glabrous. Hind wing: marginal cell linearly widened, its apical width 2.2 × width at level of hamuli (Fig. 629); 2-SC+R short longitudinal; m-cu narrowly present; M+CU:1-M = 50:33; 1r-m 0.8 × 1-M.

***Legs.*** Tarsal claws with conspicuous and robust blackish pecten, close to level of apical tooth (Fig. 635); hind coxa largely densely punctate; hind trochantellus rather robust; length of hind femur and basitarsus 4.3 and 5.6 × their width, respectively; length of inner hind spur 0.4 × hind basitarsus.

***Metasoma.*** First tergite evenly convex, 1.3 × longer than wide apically; 1^st^ and 2^nd^ tergites with medio-longitudinal carina and finely longitudinally rugose, but posterior quarter of 2^nd^ tergite smooth and no median carina; medio-basal area of 2^nd^ tergite triangular and wide (Fig. 632); 2^nd^ tergite as long as wide basally and with shallow transverse impression; 2^nd^ suture shallow and narrowly crenulate; 3^rd^ and subsequent tergites finely punctulate and strongly shiny; apical half of 3^rd^ and 4^th^ tergites without sharp lateral crease; ovipositor sheath widened apically, with medium-sized setae and apically truncate (Fig. 628).

***Colour.*** Black; hind tibia dark brown apically and remainder pale yellowish; middle and hind tarsi, and fore telotarsus blackish or dark brown; mandible, remainder of legs, mesoscutum, pronotum postero-dorsally, mesopleuron dorsally, scutellum, metanotum, ovipositor sheath and 1^st^ tergite (except pair of dark patches apically) orange; tegulae yellowish; palpi and pterostigma blackish; vein dark brown, but vein at base of wings yellowish; wing membrane largely slightly infuscate.

***Variation.*** OOL of ♀ 0.6–0.7 × diameter of posterior ocellus. The female from Turkey is very similar but has 1^st^ tergite 1.4 × as long as wide apically and 2^nd^ tergite 1.2 × longer than wide basally. Antennal segments: ♀ 53(1), 56(1), 57(1), 58(1). Apical tergites of male type 2 with fringe rather strong (Figs 645, 646). The figured male from Bulgaria has OOL rugose anteriorly, 1^st^ tergite 1.2 × as long as wide posteriorly, 2^nd^ tergite completely parallel-sided and 0.9 × as long as wide, 3^rd^ tergite rugose-striate in anterior half and parallel-sided 0.7 × as long as wide, fore femur 4.5 × longer than wide and hind femur 4.1 × longer than wide.

##### Distribution.

Azerbaijan, *Bulgaria, Croatia, *France, *Greece, Hungary, *North Macedonia, *Turkey.

##### Notes.

The holotype of *A. quadrum* is a male and it is less reliable to identify this species from it than from the holotype female of *A. illustris*; nevertheless, we accept the synonymy proposed by Papp (1985a). The figured male from Bulgaria (NMS; Figs 642–651) is considered to be this species (initially through its CO1 sequence); it is morphologically very similar to *A. cruentus* and there is a possibility that some similar males have been returned to depositories determined as *A. cruentus* with no recognition that they might belong to *A. quadrum*. However, the matter remains unresolved until more males of *A. quadrum* become available.

**Figures 627, 628.**
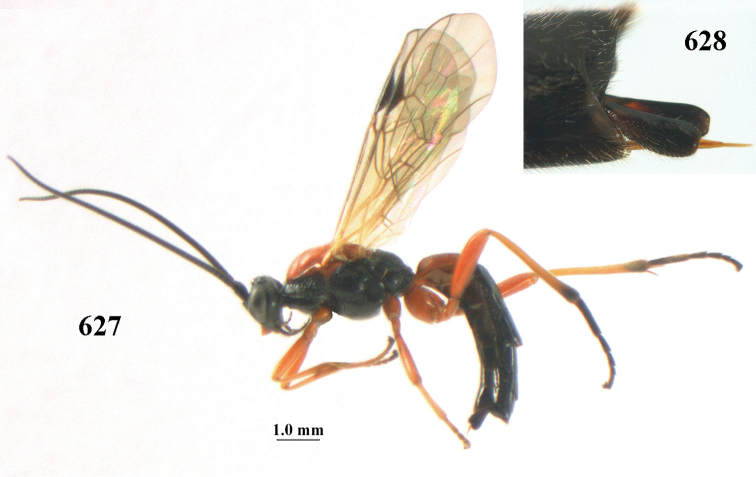
*Aleiodes quadrum* (Tobias), ♀, France, Accons **627** habitus lateral **628** ovipositor sheath lateral.

**Figures 629–641.**
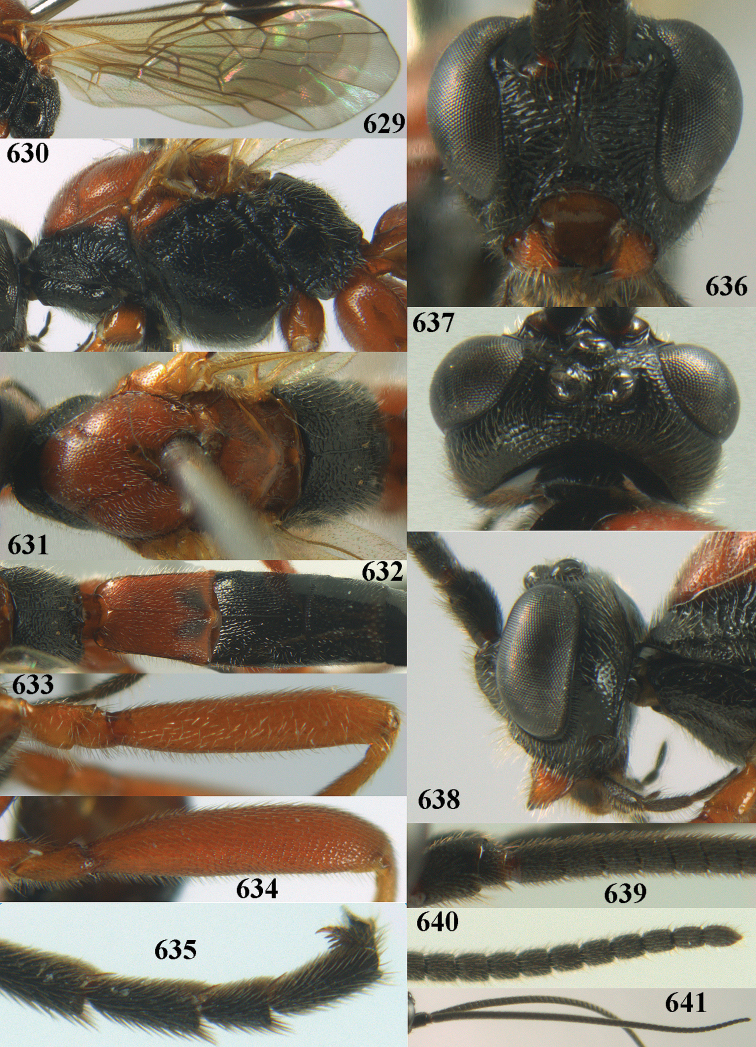
*Aleiodes quadrum* (Tobias), ♀, France, Accons **629** wings **630** mesosoma lateral **631** mesosoma dorsal **632** metasoma dorsal **633** fore femur lateral **634** hind femur lateral **635** outer hind tarsal claw **636** head anterior **637** head dorsal **638** head lateral **639** base of antenna **640** apex of antenna **641** antenna.

**Figure 642.**
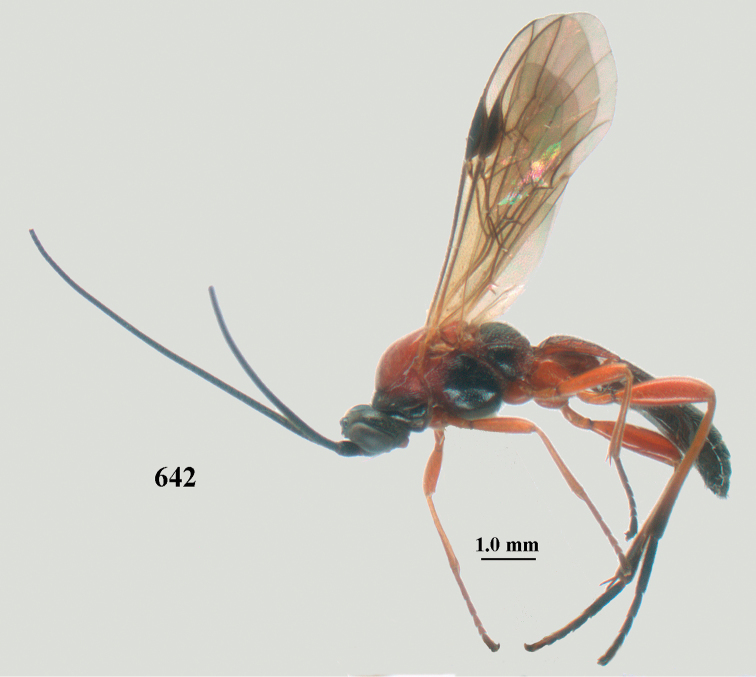
*Aleiodes quadrum* (Tobias), ♂, Bulgaria, Haskovo, habitus lateral.

**Figures 643–651.**
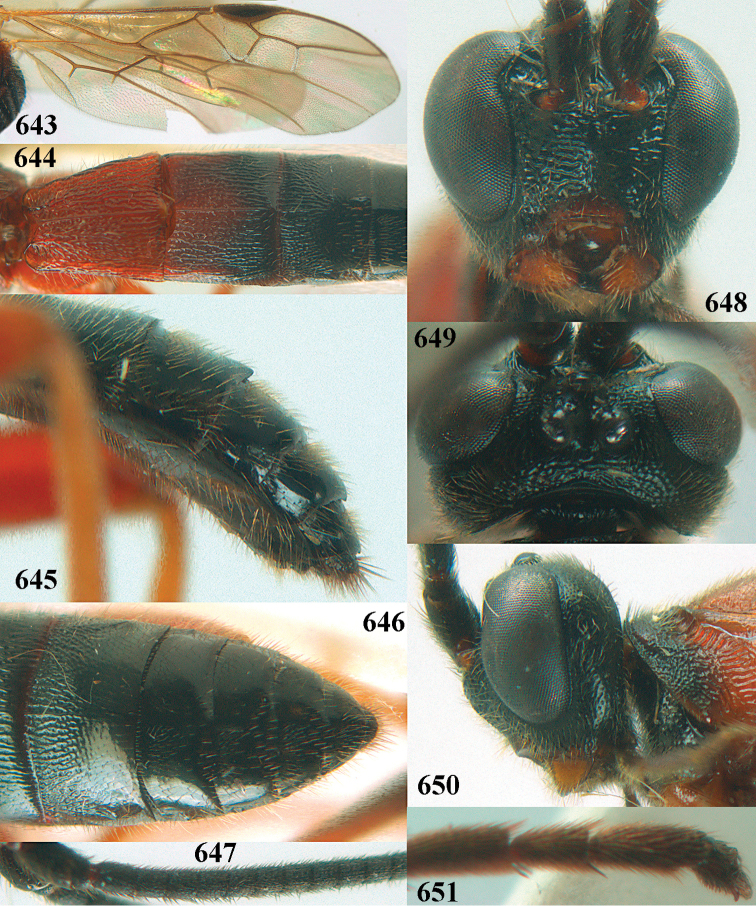
*Aleiodes quadrum* (Tobias), ♂, Bulgaria, Haskovo **643** wings **644** 1^st^–3^rd^ metasomal tergites dorsal **645** 3^rd^–7^th^ metasomal tergites lateral **646** id. dorsal **647** basal antennal segments **648** head anterior **649** head dorsal **650** head lateral **651** inner hind tarsal claw.

#### Aleiodes ruficeps

Taxon classificationAnimaliaHymenopteraBraconidae

(Telenga, 1941)

E86D9554-FF63-508C-B1D8-901E801532D8

Figs 652–653 , 654–666

Rhogas (Rhogas) ruficeps Telenga, 1941: 179, 421, Fig. [examined].
Rogas ruficeps ; Shenefelt 1975: 1146; Zaykov 1980b: 87.
Rogas (Rogas) ruficeps ; Tobias 1976: 81, 1986: 76 (transl.: 122; lectotype designation).
Aleiodes ruficeps ; Papp 1991a: 88; Fortier and Shaw 1999: 230.
Rogas gasterator auctt. p.p.

##### Type material.

Lectotype of *A. ruficeps*, ♀ (ZISP), “[**Russia**: Crimea,] Eupatoria [= Eupatoriya], Tavrits, gub, 7.v.1907, V.E. Jakovlev”, “*Rhogas ruficeps* sp. n., Telenga det.”, “Lectotype *Rogas ruficeps* Tl., design. Tobias, 1980”.

##### Additional material.

Bulgaria, Russia, Turkey, [Armenia, Iran]. Specimens in ZJUH, BZL, NMS, RMNH, ZISP, ZSSM.

##### Molecular data.

None.

##### Biology.

Unknown. Specimens collected in April-May; presumably univoltine, but there is nothing to suggest how it overwinters. We have not seen reared material.

##### Diagnosis.

**Figures 652, 653.**
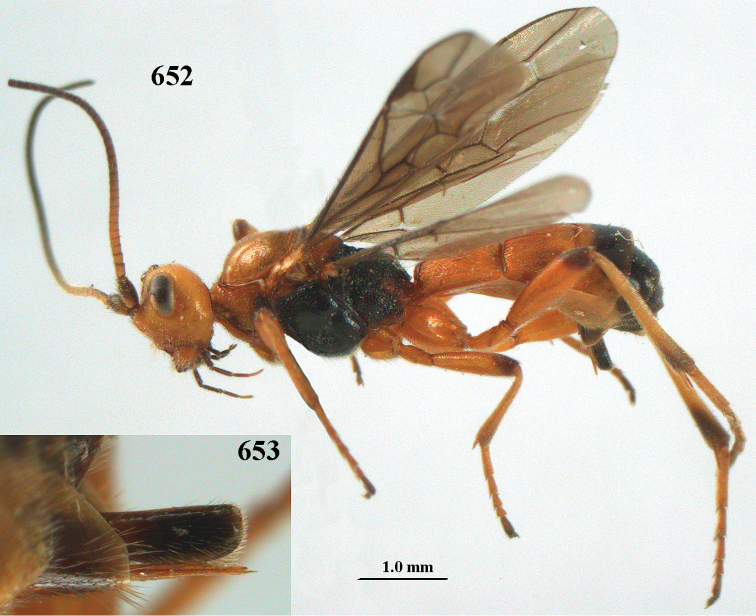
*Aleiodes ruficeps* (Telenga), ♀, Turkey, Konya **652** habitus lateral **653** ovipositor sheath lateral.

**Figures 654–666.**
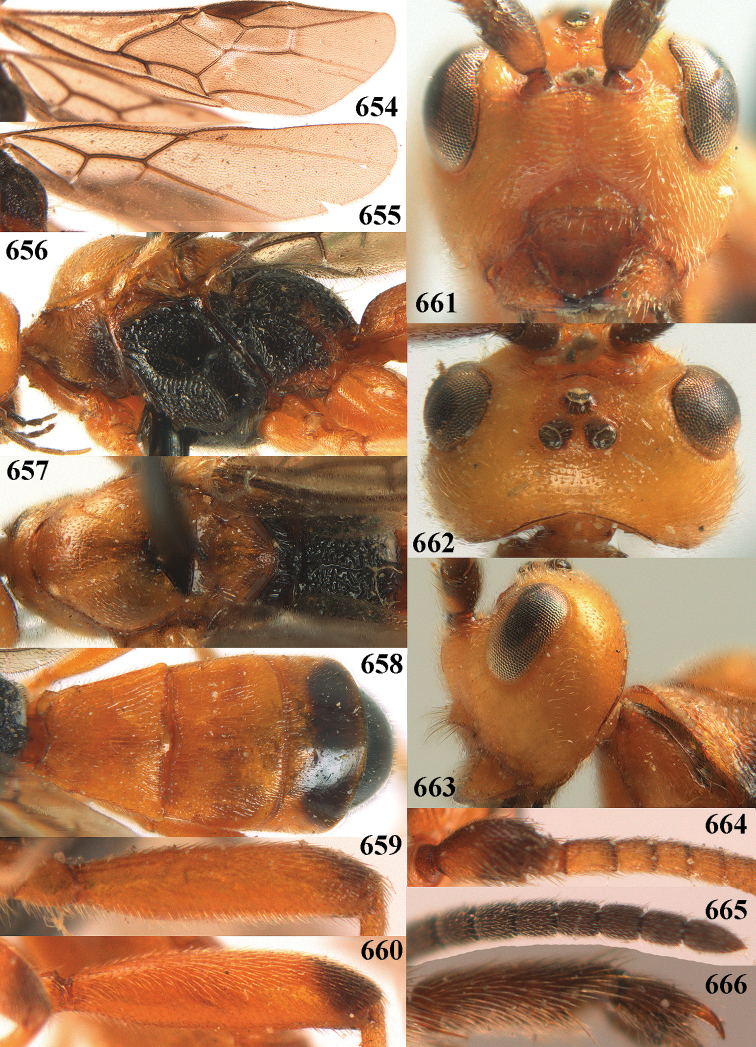
*Aleiodes ruficeps* (Telenga), ♀, Turkey, Konya **654** fore wing **655** hind wing **656** mesosoma lateral **657** mesosoma dorsal **658** 1^st^ –3^rd^ metasomal tergites dorsal **659** fore femur lateral **660** hind femur lateral **661** head anterior **662** head dorsal **663** head lateral **664** base of antenna **665** apex of antenna **666** inner hind tarsal claw.

Maximum width of hypoclypeal depression 0.5–0.6 × minimum width of face (Fig. 661); OOL ca twice diameter of posterior ocellus and moderately punctate (Fig. 662); 4^th^–10^th^ antennal segments approx.as long as wide (Figs 652, 664); ventral margin of clypeus thick to rather sharp and distinctly protruding in lateral view (Fig. 663); mesoscutal lobes punctate and interspaces largely smooth and shiny, lobes rather convex; scutellum sparsely punctate; precoxal sulcus coarsely vermiculate-rugose; marginal cell of fore wing of ♀ ending rather removed from wing apex (Fig. 654); length of vein r of fore wing 0.3 × vein 3-SR (Fig. 654); vein 1-CU1 of fore wing 0.3–0.5 × vein 2-CU1 (Fig. 654); hind tarsal claws rather slender, hardly curved and only brownish setose (Fig. 666); head completely or largely orange or yellowish; palp dark brown basally; hind femur apico-dorsally dark brown or black; hind tibia pale yellowish but apically darkened; 4^th^ and 5^th^ tergites black; wing membrane nearly entirely infuscate.

Resembles *A. grassator* because of the robust antennal segments and dark wings, but *A. ruficeps* has frons, OOL, vertex, malar space, and third tergite less sculptured, apex of hind tibia and palpi dark brown, basal antennal segments of ♀ somewhat less robust, hypoclypeal depression wider, marginal cell of fore wing slenderer and vein r of fore wing shorter. Differs from the similar *A. ruficornis* by having hypoclypeal depression wider, clypeus wider and lower, apical antennal segments of ♀ slenderer, OOL less sculptured and more antennal segments (♀: 45–47 *vs* 35–39(–41) of *A. ruficornis*).

##### Description.

Lectotype, ♀, length of fore wing 7.3 mm, of body 8.6 mm.

***Head.*** Antennal segments of ♀ 45, length of antenna 1.1 × fore wing, its subapical segments moderately robust (Fig. 665); frons with few rugae, remainder smooth; OOL 2.0 × diameter of posterior ocellus, and moderately densely punctate; vertex spaced punctate and shiny; clypeus punctate; ventral margin of clypeus thick and distinctly protruding forwards (Fig. 663); width of hypoclypeal depression 0.5 × minimum width of face (Fig. 661); length of eye 1.1 × temple in dorsal view (Fig. 662), temples subparallel-sized behind eyes; vertex behind stemmaticum punctate; clypeus distinctly below lower level of eyes; occipital carina widely reduced ventrally (Fig. 663); length of malar space 0.7 × length of eye in lateral view.

***Mesosoma.*** Mesoscutal lobes punctate and interspaces smooth, shiny; precoxal area of mesopleuron coarsely vermiculate-rugose, near precoxal area mesopleuron distinctly punctate; scutellum rather flattened, sparsely punctate; propodeum coarsely vermiculate-rugose, medio-longitudinal carina incomplete, absent posteriorly and propodeum rounded laterally.

***Wings.*** Fore wing: r 0.3 × 3-SR (Fig. 654); 1-CU1 horizontal, 0.3 × 2-CU1; r-m 0.6 × 3-SR; 2^nd^ submarginal cell rather long (Fig. 654); cu-a slightly inclivous, straight; 1-M slightly curved posteriorly; 1-SR slender; surroundings of M+CU1, 1-M and 1-CU1 largely setose. Hind wing: marginal cell linearly widened, its apical width 2.5 × width at level of hamuli (Fig. 655); 2-SC+R longitudinal; m-cu pigmented only basally; M+CU:1-M = 7:5; 1r-m 0.7 × 1-M.

***Legs.*** Tarsal claws rather slender, hardly curved and with six brownish bristles (Fig. 666); hind coxa rather weakly punctate; hind trochantellus robust; hind femur distinctly punctate; fore femur 3.3 × longer than wide; length of hind femur and basitarsus 3.2 and 5.2 × their width, respectively; length of inner hind spur 0.5 × hind basitarsus.

***Metasoma.*** First tergite rather flattened, 0.9 × as long as wide apically; 1^st^ and 2^nd^ tergites with medio-longitudinal carina and regularly longitudinally rugose; medio-basal area of 2^nd^ tergite triangular and short (Fig. 658); 2^nd^ suture rather deep and finely crenulate; basal half of 3^rd^ tergite finely striate, remainder of metasoma smooth; 4^th^ and apical half of 3^rd^ tergite without sharp lateral crease; ovipositor sheath wide, with medium-sized setae and apically truncate (Fig. 653).

***Colour.*** Orange brown; antenna, palpi, apices of femora, telotarsi, bases of fore and middle coxae, pterostigma, and veins (but 1-SR much paler than 1-M) dark brown; mesosoma (except for mesoscutum and scutellum), ovipositor sheath, 3^rd^ tergite (except basally) and subsequent tergites black or blackish; apex of hind tibia only narrowly dark brown; wing membrane nearly entirely infuscate.

***Variation.*** Vein 1-CU1 of fore wing 0.3–0.5 × vein 2-CU1. Females may have the flagellum extensively pale basally, or entirely dark. Antennal segments: ♀ 45(1), 46(1), 47(1); ♂ 56(1), 58(1). Males appear to have ca ten more antennal segments than females. Male has apical tergites type 1, setae rather long and sparse, and fringe not observed.

##### Distribution.

*Armenia, Bulgaria, *Iran, Russia, *Turkey.

#### Aleiodes ruficornis

Taxon classificationAnimaliaHymenopteraBraconidae

(Herrich-Schäffer, 1838)

F561F7BF-1CC5-5CBC-B33C-E3BA444A17B9

Figs 667–669 , 670–681 , 682–685

Rogas ruficornis Herrich-Schäffer, 1838: 156, fig.; Shenefelt 1975: 1224 (as synonym of A. dimidiatus) [neotype designated below].
Aleiodes (Neorhogas) ruficornis ; Papp 1985a: 152 (as synonym of A. dimidiatus), 1991a: 90 (id.).
Aleiodes (Chelonorhogas) ruficornis ; Belokobylskij et al. 2003: 398; van Achterberg 2014: 209; Abdolalizadeh et al. 2017: 37.
Aleiodes ruficornis ; Bergamasco et al. 1995: 5; Zaldívar-Riverón et al. 2004: 234; Papp 2005: 176 (as synonym of A. dimidiatus).
Aleiodes brevicornis Wesmael, 1838: 98; Shenefelt 1975: 1224; Papp 1985a: 152, 157 (as synonym of A. dimidiatus), 2005: 176 (id.); Belokobylskij et al. 2003 (as synonym of A. ruficornis) [examined].
Aleiodes (Neorhogas) brevicornis ; Papp 1991a: 90 (as synonym of A. dimidiatus).
Aleiodes nigripalpis Wesmael, 1838: 97; Shenefelt 1975: 1224; Papp 1985a: 152, 157 (lectotype designation; as synonym of A. dimidiatus), 1991a: 90, 2005: 176 (id.); Belokobylskij et al. 2003 (as synonym of A. ruficornis) [examined].
Rhogas dimidiatus ab. nigrobasalis Hellén, 1927: 24 (invalid name).
Rhogas dimidiatus ab. ruficollis Hellén, 1927: 24 (invalid name).
Rhogas carbonarius ab. giraudi Fahringer, 1931: 236 [unavailable name for melanistic males].
Rhogas carbonarius var. giraudi Telenga, 1941: 168. Syn. nov.
Rogas dimidiatus ab. infuscatus Hellén, 1957: 49 (invalid name).
Rogas dimidiatus ab. nigripes Hellén, 1957: 49 (invalid name).
Aleiodes (Aleiodes) arnoldii ; Farahani et al. 2015: 232–233 (but see note under A. arnoldii).
Rogas gasterator auctt. p.p.
Rogas dimidiatus auctt. p.p.

##### Type material.

Neotype of *A. ruficornis* here designated, ♀ (NMS), “[**Germany**,] Einbeck, L. 31.v.[19]85, [R. Hinz]”, “ex: *Hoplodrina blanda* Schiff. (Lep.)”. Holotype of *A. brevicornis*, ♀ (KBIN), “*A. brevicornis* ♀ mihi. 3.”, “Coll. Wesmael”, “*A. brevicornis* mihi, dét. C. Wesmael”, “**Belgique**, Liège, leg. Robert/ teste Papp J., 1983”, “Holotypus *Aleiodes brevicornis* Wesm., 1838 ♀, det. Papp, 1983”, “*Aleiodes dimidiatus* Spin. ♀, det. Papp J., 1983”. Holotype of *A. nigripalpis*, ♂ (KBIN), “*A. nigripalpis* ♂ mihi. 2.”, “Coll. Wesmael”, “*A. nigripalpis* mihi, dét. C. Wesmael”, “Belgique, Liège/ teste Papp J., 1983”, “Lectotypus *Aleiodes nigripalpis* Wesm., 1838 ♂, det. Papp, 1983”, “*Aleiodes dimidiatus* Spin. ♂, det. Papp J., 1983”. The neotype designation for *A. ruficornis* is necessary for nomenclatural stability, because the types of Braconidae described by Herrich-Schäffer are lost (Horn and Kahle 1935–37; the first author could not find any specimen in ZMB) and the species has been confused with similar species in the past. The specimen is selected because it fits well the original description, the probable type location was in Germany, it has been reared and it is in good condition.

##### Additional material.

Andorra, Austria, British Isles (England (V.C. 5, 11, 13, 15, 17, 19, 20, 22, 23, 24, 28, 29, 31, 33, 38, 39, 60, 63); Wales (V.C. 52) [no specimens seen from Scotland]), Bulgaria, Croatia, Czech Republic, Finland, France, Germany, Greece, Hungary, Italy (including Sicily), Moldova, Montenegro, Netherlands (DR: Borger; LI: Venlo, ZH: Oostkapelle), North Macedonia, Norway, Romania, Russia (including Far East), Serbia, Slovakia, Sweden, Switzerland, Turkey, Ukraine, [Afghanistan, Dagestan, Iran, Kazakhstan, Kyrgyzstan]. Specimens in ALC, ZJUH, BZL, FMNH, MMUM, MRC, MSC, MSNV, MTMA, NMS, NRS, OUM, RMNH, SDEI, UNS, UWIM, ZISP, ZSSM. This is a widespread species, generally common, but partly replaced by *A. gasterator* in the Mediterranean region.

##### Molecular data.

MRS140 (UK), MRS877 (Sweden), MRS888 (UK), MRS890 (UK), MRS891 (UK).

##### Biology.

Specimens collected from April–September; probably plurivoltine in the southern part of its range but in Britain univoltine, flying from June–August with a varied means of overwintering (see below). Reared from the noctuids *Agrotis clavis* (Hufnagel) (4 [4 ZISP]/ Russia), *Agrotis segetum* (Dennis & Schiffermüller) (1 [FMNH]/Finland), *Agrotis* sp. (3), *Euxoa nigricans* (Linnaeus) (1 [FMNH]/Finland), *Euxoa* sp. (3:1 [3 FMNH/Finland], *Hoplodrina blanda* (Dennis & Schiffermüller) (4:1, Germany; R. Hinz), *Hoplodrina octogenaria* (Goeze) (1; W.A. Watson), *Mythimna impura* (Hübner) (1 [ZSSM]/Germany; E. Haeselbarth). A further mummy from *H. blanda* failed to emerge (M.R. Shaw), but was no doubt of this species. The above hosts belong to three different subfamilies of Noctuidae, all feeding and resting close to ground level. In addition, we have seen a specimen labelled as ex the nymphalid *Brenthis ino* (Rottemburg) but accompanied by a mummy of a noctuid, probably *Hoplodrina* sp. (det. M.R. Shaw). In experiments a range of hosts recorded for this species in the literature (several arctiine and lymantriine Erebidae and the lasiocampid *Lasiocampa quercus* (Linnaeus)) that are actually hosts of superficially similar species such as *A. alternator* (Nees) were offered to the female reared ex *H. octogenaria* but, as expected, they were firmly rejected. However, this female readily accepted late 1^st^ instar larvae of *Agrotis exclamationis* (Linnaeus), from which adult progeny resulted very smoothly (1:16\13\\12\12+0. The few failures to oviposit were almost certainly due to egg depletion). Searching in the vicinity of hosts included antennal drumming (the tips curled downwards) and indeed the antennae seemed to be the only proximal means of locating and assessing the host. Once the host was found it was immediately accepted, rapidly jabbed and stood over or often withdrawn from (1.0–1.5 cm) while the venom took affect (20–40 secs), then relocated via antennal searching (when it had been withdrawn from this might take up to a minute, but it was always eventually successful) scooped in with the fore legs (the antennae only slightly involved), positioned and held between the mid legs for the duration of oviposition (20–30 secs). Frequently the host larva was kicked free of the ovipositor by the parasitoid’s hind leg(s) and the parasitoid rapidly left without any period of post-oviposition association. Recovery from the venom was rather protracted (up to 20 mins), during which time hosts were rejected if rediscovered. Towards the end of successful oviposition runs it was evident that venom depletion ran ahead of egg depletion, resulting in erratic (but nevertheless successful) oviposition sequences. No host feeding took place. In this experimental series oviposition took place in mid-July with mummification at the end of August and adult emergence in late May of the following year. However, although the winter was passed in the mummy in this entire series (and probably also the case for the other, natural, *Agrotis* hosts), it is clear that the rearings from *Hoplodrina* and probably also *Mythimna* involved overwintering in the host larva with adult emergence in the year of mummification. This host-related difference in overwintering is not inconsistent with univoltinism in Britain (where the experiments and other observations were done) but it is certainly an interesting quirk of its host range and might be of significance in suggesting one way in which a temporal isolating mechanism could potentially arise as a forerunner to speciation (cf. Shaw, 2003). The mummy is formed in the soil and is not strongly (if at all) glued to the substrate. It is predominantly dark brown in colour, very large in relation to the size of the insect that will emerge and, although basically cylindrical, somewhat flattened in appearance owing to a pronounced but blunt lateral keel (Fig. 669). It is more or less strongly contracted at the anterior end, markedly less so caudally, and the copiously silken lining typically occupies 3^rd^–8^th^ abdominal segments.

##### Diagnosis.

Maximum width of hypoclypeal depression 0.4–0.5 × minimum width of face (Fig. 676); OOL of ♀ 1.4–1.6 × as long as diameter of posterior ocellus and distinctly rugose or rugulose (Fig. 677); length of 4^th^ antennal segment of ♀ 1.0–1.4 (of ♂ 1.1–1.4) × its width (Fig. 679); ventral margin of clypeus thick and not protruding anteriorly (Fig. 678); lobes of mesoscutum densely punctate, interspaces largely smooth and shiny; precoxal area coarsely vermiculate-rugose medially; marginal cell of fore wing of ♀ usually ending rather removed from wing apex (Fig. 670); vein 1-CU1 of fore wing 0.4–0.6 × as long as vein 2-CU1; hind tarsal claws yellowish or brownish bristly setose and with few yellowish pectinal teeth (Fig. 681); hind femur at least apico-dorsally dark brown or black; inner side of hind tibia of ♀ yellowish; pale males have whole frons and stemmaticum yellowish; palpi dark brown or blackish, rarely brown; 3^rd^ metasomal tergite only antero-laterally reddish or yellowish; 4^th^ and 5^th^ tergites black. Specimens from high altitude have the head conspicuously long setose and the tarsal claws brownish pectinate basally. In this respect males are similar to *A. hirtus*, but *A. hirtus* has precoxal area and mesoscutum largely smooth and clypeus distinctly protruding in lateral view.

##### Description.

Neotype, ♀, length of fore wing 3.9 mm, of body 6.5 mm.

***Head.*** Antennal segments of ♀ 35, antenna as long as fore wing, its subbasal and subapical segments robust (Figs 679, 680); frons with curved rugae; OOL 1.2 × diameter of posterior ocellus, rugose and moderately shiny; vertex rugose, rather shiny; clypeus rugose; ventral margin of clypeus thick and not protruding forwards (Fig. 678); width of hypoclypeal depression 0.5 × minimum width of face (Fig. 676); length of eye twice temple in dorsal view (Fig. 677); vertex behind stemmaticum rugose; clypeus below lower level of eyes; length of malar space 0.5 × length of eye in lateral view; temple punctate and shiny, but rugulose near occipital carina.

***Mesosoma.*** Mesoscutal lobes distinctly punctate, interspaces of lateral lobes smooth and shiny; precoxal area of mesopleuron coarsely vermiculate-rugose medially, but posteriorly punctate; mesopleuron punctate medially; metapleuron distinctly rugose ventrally and dorsally punctate; scutellum largely smooth (except for punctulation), rather shiny and nearly flat, with lateral carina; propodeum coarsely reticulate-rugose, laterally dorsal face longer than posterior one, somewhat angulate laterally but without tubercles, and with complete medio-longitudinal carina.

***Wings.*** Fore wing: r 0.3 × 3-SR; marginal cell short (Fig. 670); 1-CU1 horizontal, 0.5 × 2-CU1; r-m 0.6 × 3-SR; 2^nd^ submarginal cell medium-sized (Fig. 670); cu-a inclivous, straight; 1-M rather curved posteriorly; 1-SR slightly wider than 1-M; surroundings of M+CU1, 1-M and 1-CU1 largely setose. Hind wing: marginal cell linearly widened, its apical width 2.2 × width at level of hamuli (Fig. 670); 2-SC+R subquadrate; m-cu narrowly pigmented; M+CU:1-M = 30:19; 1r-m 0.6 × 1-M.

***Legs.*** Tarsal claws mainly setose and medially with 4 yellowish rather short pectinal teeth (Fig. 681); hind coxa punctate and shiny; hind trochantellus robust; length of hind femur and basitarsus 3.3 and 4.0 × their width, respectively; length of inner hind spur 0.5 × hind basitarsus.

***Metasoma.*** First tergite rather convex medially, 0.9 × longer than wide apically, robust and coarsely irregularly longitudinally rugose as 2^nd^ tergite; 1^st^ tergite and basal half of 2^nd^ tergite with medio-longitudinal carina; medio-basal area of 2^nd^ tergite triangular and rather distinct (Fig. 673); 2^nd^ suture deep and crenulate; basal half of 3^rd^ tergite largely longitudinally striate, remainder of metasoma superficially micro-sculptured or smooth; 4^th^ and apical half of 3^rd^ tergite without sharp lateral crease; ovipositor sheath wide, with medium-sized setae and apically truncate (Fig. 668).

***Colour.*** Reddish or orange-brown; stemmaticum medially, malar space largely, temple and occiput ventrally, mesosternum, mesopleuron ventrally, 3^rd^ tergite (except antero-laterally), 4^th^–7^th^ tergites black; palpi (only labial palp darkened basally) brown; basal half of antenna, tegulae, parastigma, and base of pterostigma pale yellowish; apical half of antenna, pedicellus dorsally, propodeum dorsally and medially, middle and hind femora apico-dorsally, and telotarsi dark brown; remainder of pterostigma and veins dark brown or brown (Fig. 670); fore wing membrane rather infuscate, but hind wing nearly subhyaline.

***Variation.*** Female: mesosoma occasionally wholly black. Male face and mesosoma usually black but can be variably marked with red; scape and pedicel usually (partly) reddish in central and southern populations but most often entirely black in more north-western ones (e.g., British Isles, Sweden); hind coxa varies from black to red. Length of malar space 0.5–0.6 × length of eye in lateral view; head black or largely reddish brown (except temple ventrally and malar space); interspaces of mesoscutal lobes smooth to micro-sculptured; 1-CU1 0.4–0.6 × 2-CU1; 3^rd^ tergite longitudinally striate or rugulose basally (sometimes narrowly so), without curved sculptural elements (Fig. 673), except sometimes some weak transverse striae occasionally present at extreme apex; males from montane habitats are generally darker than lowland males. Antennal segments: ♀ 34(6), 35(12), 36(15), 37(22), 38(17), 39(13), 40(2), 41(3), 42(2), 43(1); ♂ 43(1), 44(1), 45(1), 46(1), 47(5), 48(13), 49(32), 50(19), 51(36), 52(36), 53(28), 54(11), 55(4), 56(4), 57(2). On average males have ca 14 more antennal segments than females. Male has marginal cell of fore wing less robust than in ♀, with apical tergites type 1–2, density of setae rather variable and fringe evident but sparse (Fig. 682).

##### Distribution.

*Afghanistan, *Andorra, Austria, British Isles (England, Wales), *Bulgaria, *Croatia, *Czech Republic, *Finland, *France, Germany, *Iran, *Kazakhstan, *Kyrgyzstan, *Montenegro, *Netherlands, *North Macedonia, *Norway, *Romania, *Russia (including Dagestan and Far East), *Serbia, *Slovakia, *Sweden, Switzerland, *Turkey, *Ukraine.

##### Notes.

An examined female (NMS) from Hungary, Borzsony Mts., 140 m altitude, 20–30.vii.2005 (unfortunately, too damaged for description) represents a very similar but new species. The 4^th^–10^th^ antennal segments are not moniliform, slenderer than in typical *A. ruficornis*, the fore femur is more robust than in *A. ruficornis*, and the COI sequence (MRS886) is different (2.1 %).

*Aleiodes ruficornis* is the commonest and most widespread of a small group of related species parasitising grassland and “cutworm” hosts, exhibiting strong sexual dimorphism with unremarkable males but the more extensively orange females having a stronger build and much shorter antennae. The least extreme in these respects is *A. gasterator*, which largely (but not completely) replaces *A. ruficornis* in the Mediterranean region. *Aleiodes grassator* is similar to *A. ruficornis*, but it appears to be restricted to montane and northern habitats where it might be thought to replace *A. ruficornis*. However, some males that morphologically agree best with *A. ruficornis* have been collected at high altitude in the Alps (up to 2550 m), where *A. improvisus* also occurs, but whether these high-altitude *A. ruficornis* males are parts of breeding populations or have simply been carried up in thermals is impossible to say. The females in this group (excluding *A. gasterator*) are scarcer in collections than males, as they fly very little and rarely enter Malaise traps.

**Figures 667–669.**
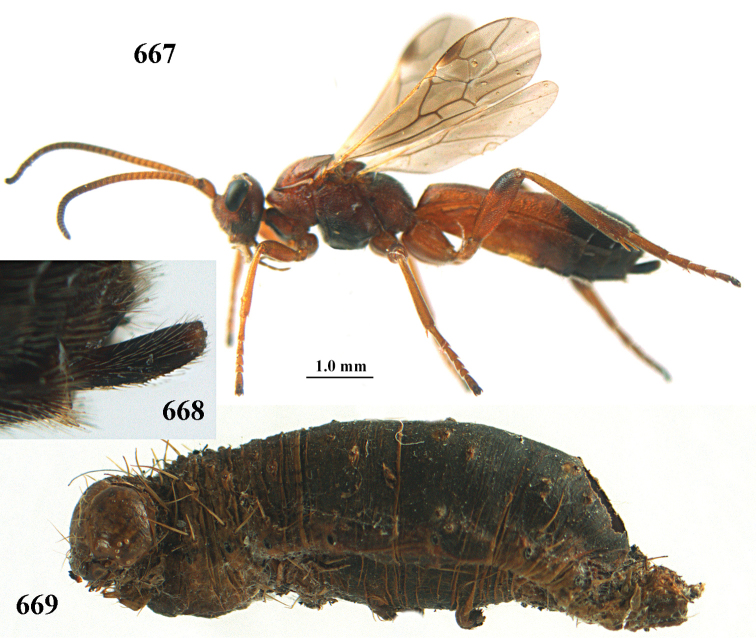
*Aleiodes ruficornis* (Herrich-Schäffer), ♀, neotype **667** habitus lateral **668** ovipositor sheath lateral **669** mummy of *Hoplodrina blanda* (Denis & Schiffermüller).

**Figures 670–681.**
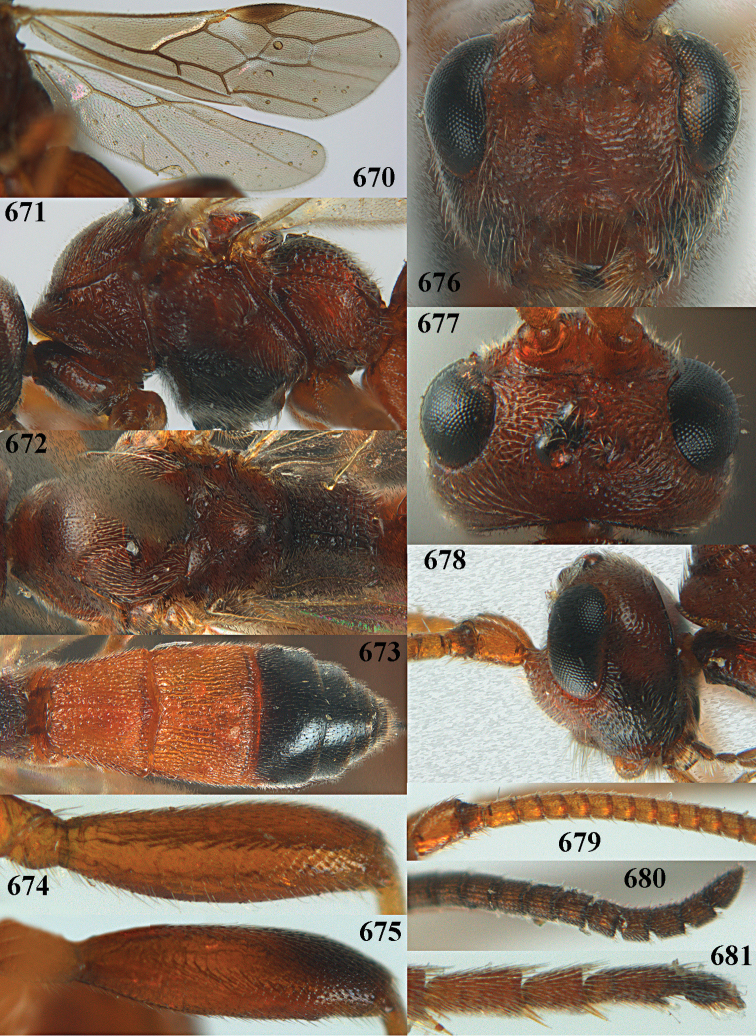
*Aleiodes ruficornis* (Herrich-Schäffer), ♀, neotype **670** wings **671** mesosoma lateral **672** mesosoma dorsal **673** metasoma dorsal **674** fore femur lateral **675** hind femur lateral **676** head anterior **677** head dorsal **678** head lateral **679** base of antenna **680** apex of antenna **681** outer hind tarsal claw.

**Figures 682–685.**
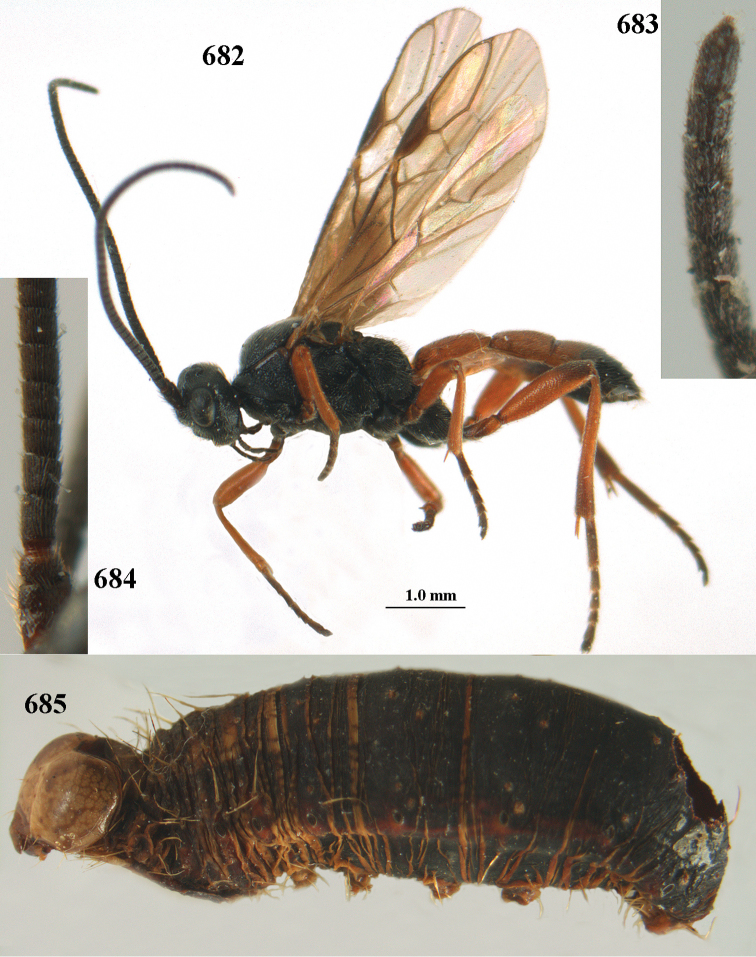
*Aleiodes ruficornis* (Herrich-Schäffer), ♂, U.K. (culture) **682** habitus lateral **683** apex of antenna **684** base of antenna **685** mummy of *Hoplodrina octogenaria* (Goeze).

#### Aleiodes rufipes

Taxon classificationAnimaliaHymenopteraBraconidae

(Thomson, 1892)

AEFA9612-4F10-5BCC-8892-DBC764BE5C09

Figs 686–687 , 688–700

Rogas rufipes Thomson, 1892: 1669; Shenefelt 1975: 1224; Kotenko 1992: 96 [examined].
Rogas (Rogas) rufipes ; Tobias 1986: 81 (transl.: 133).
Aleiodes (Neorhogas) rufipes ; Papp 1985a: 162, 1987b: 36, 1991a: 88; Belokobylskij 1996: 14; Riedel et al. 2002: 106.
Aleiodes (Chelonorhogas) rufipes ; Chen et al. 1992: 496; Belokobylskij 2000: 40; Chen and He 1997: 42; He et al. 2000: 665; Ghahari et al. 2011: 4; Farahani et al. 2015: 229, 244.
Aleiodes rufipes ; Fortier and Shaw 1999: 228; Papp 2002: 562, 2005: 177; Aydogdu and Beyarslan 2005: 191, 192.

##### Type material.

Holotype, ♀ (ZIL), “Lap”, “*rufipes* m”, “**Sverige** [= Sweden], Lappland, teste Papp J., 1983”, “Holotypus”, “*Rogas rufipes* Thoms., 1891, ♀, Papp, 1983”.

##### Additional material.

Finland, Norway, Sweden. Specimens in FMNH, NMS, MTMA, RMNH, ZIL.

##### Molecular data.

MRS294 (Sweden), MRS312 (Sweden), MRS314 (Sweden), MRS673 (Finland), MRS674 (Finland), MRS676 (Finland), MRS680 (Finland).

##### Biology.

Unknown. Collected from July–August; presumably univoltine. We have not seen reared material and there is no indication of how the winter may be passed.

##### Diagnosis.

**Figures 686, 687.**
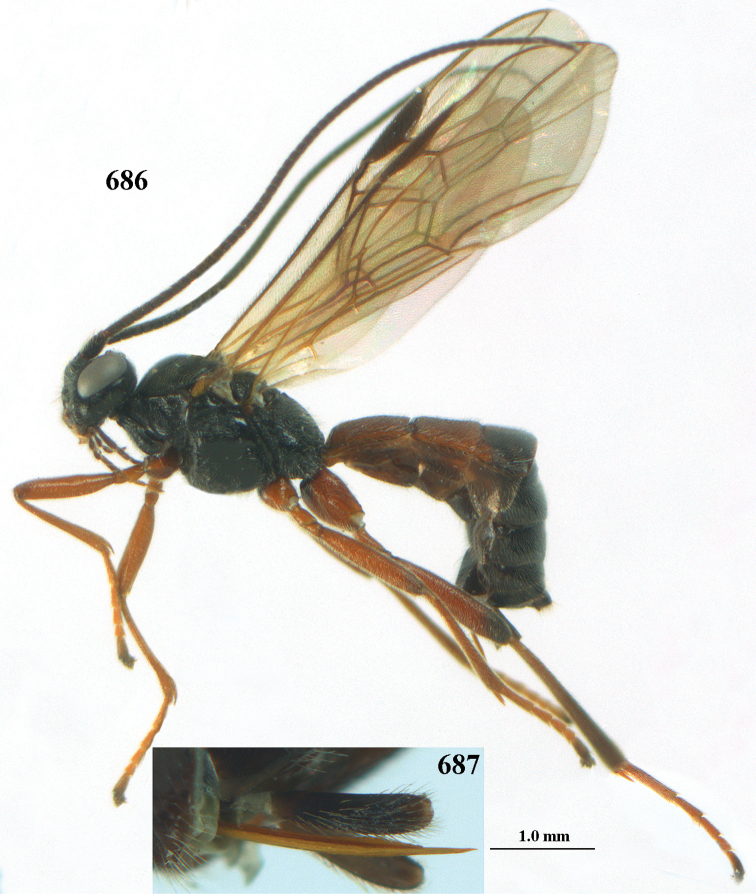
*Aleiodes rufipes* (Thomson), ♀, Sweden, Lillav **686** habitus lateral **687** ovipositor sheath lateral.

Maximum width of hypoclypeal depression approx. 0.4 × minimum width of face (Fig. 695); OOL of ♀ 1.1–1.5 × as long as diameter of posterior ocellus and punctate-rugulose to coriaceous-rugose (Fig. 696); length of antenna of ♀ 1.0–1.1 × length of fore wing; ventral margin of clypeus thin and not protruding in lateral view (Fig. 697); mesoscutal lobes remotely punctulate and with satin sheen; area of precoxal sulcus smooth; length of vein 1-CU1 of fore wing 0.2–0.3 × vein 2-CU1 and 0.4 × vein m-cu; vein 2-SC+R of hind wing subquadrate; tarsal claws with robust apical tooth and with medium-sized yellowish brown pecten (Fig. 698); hind femur and basitarsus slender (Fig. 686); 1^st^ metasomal tergite comparatively steep anteriorly (Fig. 686); head (largely) black; apex of hind femur usually largely black dorsally; basal half of hind tibia (largely) pale yellowish; 2^nd^ tergite yellowish or reddish; males usually with dense and long setosity on at least basal half of 4^th^–6^th^ tergites.

##### Description.

Redescribed ♀ (RMNH) from Finland (Enntekiö). Length of fore wing 5.3 mm, of body 5.8 mm.

***Head.*** Antennal segments of ♀ 51, antenna as long as fore wing, its subapical segments rather robust and apical segment with short spine (Fig. 700); frons largely smooth, except for some micro-sculpture; OOL 1.2 × diameter of posterior ocellus, coriaceous-rugose and slightly shiny, groove beside posterior ocellus rather shallow and crenulate; vertex coriaceous with some rugulae, rather dull; clypeus rugose; ventral margin of clypeus thin and not protruding forwards (Fig. 697); width of hypoclypeal depression 0.4 × minimum width of face (Fig. 695); length of eye 1.7 × temple in dorsal view (Fig. 696); vertex behind stemmaticum coriaceous-rugulose; clypeus near lower level of eyes; length of malar space 0.4 × length of eye in lateral view.

***Mesosoma.*** Mesoscutal lobes largely punctate-coriaceous, with satin sheen; precoxal area of mesopleuron partly remotely punctate as its surroundings; medio-longitudinal carina of metanotum distinct posteriorly; scutellum punctate; propodeum convex and coarsely rugose, medio-longitudinal carina absent posteriorly, and without protruding carinae laterally.

**Figures 688–700.**
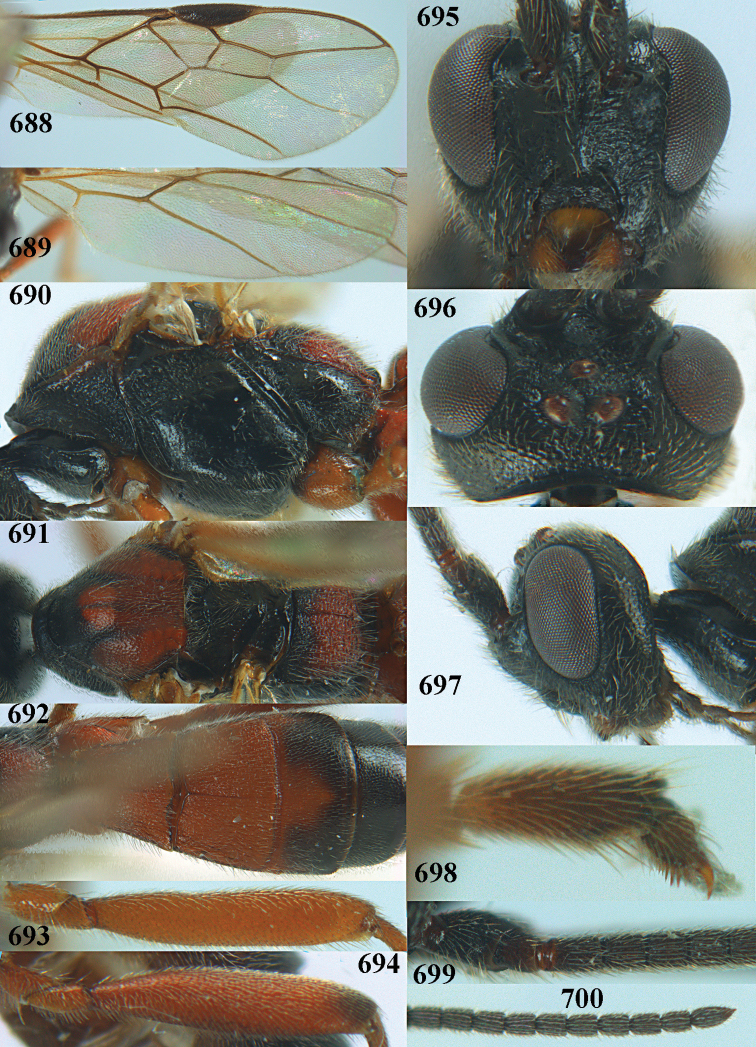
*Aleiodes rufipes* (Thomson), ♀, Sweden, Lillav **688** fore wing **689** hind wing **690** mesosoma lateral **691** mesosoma dorsal **692** 1^st^–3^rd^ metasomal tergites dorsal **693** fore femur lateral **694** hind femur lateral **695** head anterior **696** head dorsal **697** head lateral **698** outer hind tarsal claw **699** base of antenna **700** apex of antenna.

***Wings.*** Fore wing: r 0.3 × 3-SR (Fig. 688); 1-CU1 slightly oblique, 0.3 × 2-CU1; r-m 0.5 × 3-SR; 2^nd^ submarginal cell long (Fig. 688); cu-a slightly inclivous, straight but posteriorly slightly curved; 1-M nearly straight posteriorly; 1-SR wide; surroundings of M+CU1, 1-M and 1-CU1 densely setose. Hind wing: marginal cell linearly widened, its apical width 1.7 × width at level of hamuli (Fig. 689); 2-SC+R subquadrate; m-cu absent; M+CU:1-M = 26:25; 1r-m 0.6 × 1-M.

***Legs.*** Tarsal claws with rather conspicuous and medium-sized brownish pecten (Fig. 698); hind coxa densely punctate; hind trochantellus rather robust; length of hind femur and basitarsus 4.8 and 6.4 × their width, respectively; length of inner hind spur 0.5 × hind basitarsus.

***Metasoma.*** First tergite convex and basally rather steep, 0.9 × longer than wide apically; 1^st^ and 2^nd^ tergites with medio-longitudinal carina and longitudinally rugose; maximum width of 2^nd^ tergite 1.6 × its median length; medio-basal area of 2^nd^ tergite medium-sized triangular and rather short (Fig. 692); 2^nd^ suture deep and finely crenulate; basal half of 3^rd^ tergite rugulose, remainder of metasoma superficially micro-sculptured; 4^th^ and apical half of 3^rd^ tergite without sharp lateral crease; ovipositor sheath wide, with long setae and apically truncate (Fig. 687).

***Colour.*** Black; mesoscutum largely, legs, and 1^st^–3^rd^ metasomal tergites (but 3^rd^ tergite narrowly infuscate posteriorly) reddish brown; tegulae brownish yellow; ovipositor sheath, palpi, pterostigma and veins dark brown; telotarsi partly infuscate; wing membrane subhyaline.

***Variation.*** Legs usually largely reddish, but telotarsi, apices of hind femur and tibia frequently dark brown and sometimes most of hind tibia and apical half of hind femur black; clypeus blunt to rather acute ventrally; depression near posterior ocelli smooth or finely crenulate; mesoscutum of ♀ usually partly reddish brown, but sometimes largely or entirely black; ventral third of mesopleuron regularly and finely punctate. Antennal segments: ♀ 57(2), 59(1); ♂ 57(1), 59(1), 60(5), 61(1), 62(1). Male is very similar with mesoscutum black (rarely partly reddish) and at least 2^nd^ tergite orange brown (sometimes with pair of dark brown patches), apical tergites type 1–2 with fringe rather strong when visible, and also often evident on tergites following the third.

##### Distribution.

Finland, Norway, Sweden.

#### Aleiodes rugulosus

Taxon classificationAnimaliaHymenopteraBraconidae

(Nees, 1811)

BC048A17-A771-52A8-AE01-FD716951E65F

Figs 701–703 , 704–716 , 717, 718 , 719 , 720–727

Bracon rugulosus Nees, 1811: 32; Papp 1985a: 162 (neotype designation).
Rogas rugulosus ; Shenefelt 1975: 1247–1248.
Rogas (Rogas) rugulosus ; Tobias 1976: 84, 1986: 78 (transl.: 128).
Aleiodes (Neorhogas) rugulosus; Papp 1987b: 36, 1991a: 79; Riedel et al. 2002: 106.
Aleiodes (Chelonorhogas) rugulosus ; Belokobylskij et al. 2003: 398.
Aleiodes rugulosus ; Bergamasco et al. 1995: 5; Zaldívar-Riverón et al. 2004: 234; Papp 2005: 177.
Rhogas rugulosus var. pictus Kokujev, 1898: 296; Shenefelt 1975: 1247–1248 (not Herrich-Schäffer 1838).

##### Type material.

Neotype, ♀ (KBIN), “*A. rugulosus*”, “dét. C. Wesmael”, “Coll. Wesmael”, “Belgique, Bruxelles”/ teste Papp J., 1983”, “Neotypus, *Bracon rugulosus* Nees, 1812 [sic!], ♀, Papp 1983”, “*Aleiodes rugulosus* Ns. ♀, det. Papp J., 1983”.

##### Additional material.

Albania, Austria, Belgium, Bulgaria, Czech Republic, British Isles (England: V.C.s 1, 3, 4, 11, 17, 25, 27, 29, 69; Wales: V.C.s 41, 48; Scotland: V.C.s 78, 81, 88, 98; Ireland: V.C. H30), Finland, France, Germany, Hungary, Moldova, Netherlands (DR: Borger; GE: ‘t Harde; Heerde; Otterlo, NB: Kampina; NH: Crailo; OV: Buurserzand), North Macedonia, Norway, Poland, Romania, Russia, Slovakia, Spain, Sweden. Specimens in ALC, ZJUH, BZL, HHC, IKC, MMUM, MRC, MSC, MSNV, MTMA, NMS, NRS, OUM, RMNH, SDEI, UWIM, ZSSM.

##### Molecular data.

MRS191 (Hungary), MRS217 (UK), MRS398 (France), MRS884 (Poland).

##### Biology.

Collected in (May)July–August(September), usually in open habitats such as mosses, heaths, herb-rich grasslands and fens. Partly plurivoltine, at least in the southern part of its range, but largely univoltine in the north (in a UK culture only one female out of 20 reared, and two males out of 69, emerged in the same year as mummy formation). Reared from Noctuidae, Acronictinae: *Acronicta auricoma* (Denis & Schiffermüller) (7 [1 BZL, 1 MSC, 1 ZMUO]; J. Voogd/Netherlands, M & J. Schwarz/Austria, Finland), *Acronicta euphorbiae* (Dennis & Schiffermüller)/*cinerea* (Hufnagel) (5 [2 IKC, 1 BZL, 1 FMNH]; M.J. Pellinen, D. & J. Steedan), *Acronicta menyanthidis* (11; R.P. Knill-Jones, W.A. Watson), *Acronicta rumicis* (Linnaeus) (5 [1 ZJUH, 1 IKC, 1 MTMA]; R.J. Heckford, M.J. Pellinen, M.R. Shaw), *Oxicestra geographica* (Fabricius) (17 [12 BZL, 5 MTMA]; Hungary), *Acronicta* sp. on low plants (3), *Simyra albovenosa* (Goeze) (14 [5 ZJUH, 4 FMNH, 2 UMZC, 1 NRS]; M.R. Shaw); A. Lozan, Romania), *Acronicta euphorbiae* (Dennis & Schiffermüller) on *Euphorbia sanguinea* Hochst (5 [2 RMNH, 3 ALC]). These species all feed on low plants. The mummy is moderately arched, very strongly glued down (usually to a narrow twig or stem low in the vegetation), and persists through the winter. The pupation chamber, occupying approximately abdominal segments 3–7 of the host, is rather densely lined with silk which is laid down after the mummy has hardened suggesting that the larva within can turn easily. Rearing experiments, undertaken using stock originally reared from *A. menyanthidis*, suggested that this host and *A. rumicis* were equally suitable, but most experiments were not conducted in a way to provide clear data in this respect. The behaviour of the adult females towards these hosts indicated some adaptation to use of highly aggregated species (i.e., that lay large batches of eggs) as, firstly there was a habituation process whereby repeated contacts with hosts generally preceded oviposition, and secondly there was only weak displacement following oviposition (resulting in rather frequent super-parasitism). The antennae were used to locate hosts with wide sweeping motions, and usually the host curled up and was manipulated backwards against the hind tarsi before the ovipositor was inserted and the egg was laid. Generally, there was no pre-oviposition sting and post-oviposition association with the only slightly subdued host was minimal, but the oviposition process was variable and occasionally there was a brief jab, but no subsequent waiting period, before oviposition. Less enthusiasm for sub-active hosts, such as those oviposited into a few seconds or minutes earlier, provided a short-lived impediment to super-parasitism, although sometimes two (on one occasion four, confirmed by dissection) eggs were laid into a single host in separate consecutive bouts without the parasitoid really relinquishing the host. First instar hosts were less easy than 2^nd^ or 3^rd^ instars for the parasitoid to deal with, and although oviposition into 2^nd^ instar hosts was somewhat more successful than into 3^rd^ instars, occasionally successful oviposition into early 4^th^ instar hosts occurred. Mean development times from oviposition to mummification in different instar hosts (*A. rumicis*) under the same ambient conditions (Reading, S. England, July) varied as might be expected given that mummification was always at essentially the same (penultimate instar) stage of the host’s larval life: for 1^st^ (*N* = 23), 2^nd^ (*N* = 40) and 3^rd^ (*N* = 7) instars, 27.0, 25.5 and 20.7 days, respectively. There is no venom effect to influence successful host development. Opportunities to offer other hosts were limited but it was clear that, although oviposition into larvae of the closely related arboreal species *Subacronicta megacephala* (Dennis & Schiffermüller) was fairly readily obtained (*N* = 15), though slightly inhibited by the host’s adherence to its silken pad rather than curling up, the parasitoid was always encapsulated (as a 1^st^ instar larva in observed cases) and no progeny resulted. No rearings of *A. rugulosus* from arboreal Acronictinae have been seen, although these conspicuous larvae are often collected and reared.

##### Diagnosis.

Maximum width of hypoclypeal depression 0.3–0.4 × minimum width of face (Fig. 711); OOL approx. equal to diameter of posterior ocellus and coarsely punctate (Fig. 712); vertex flattened behind ocelli; ventral margin of clypeus thick and not protruding in lateral view (Fig. 713); mesoscutal lobes coriaceous; mesopleuron (including precoxal sulcus area) nearly or completely smooth; propodeum with pair of crest-like protuberances laterally; vein 1-CU1 of fore wing much shorter than vein 2-CU1; basal half of marginal cell of hind wing parallel-sided and subapically widened (Fig. 704); tarsal claws with large dark brown pecten up to apical tooth of claw (Fig. 716); hind spurs (dark) reddish brown; hind tibial spurs of ♂ acute apically (Fig. 722); head black; dorsal 0.4 of mesopleuron, mesosternum and scutellum black; metasoma entirely black (typical) or 1^st^ and 2^nd^ tergites orange or yellowish brown; apex of hind femur yellowish or reddish; basal half of hind tibia pale yellowish.

##### Description.

Redescribed ♀ (RMNH) from Netherlands (Buurserzand). Length of fore wing 6.3 mm, of body 7.7 mm.

***Head.*** Antennal segments of ♀ 65, length of antenna 1.3 × fore wing, its subapical segments rather slender (Fig. 716); frons largely smooth except few striae; OOL equal to diameter of posterior ocellus, coarsely punctate and shiny; vertex coarsely punctate with some rugae, shiny; clypeus coarsely punctate; ventral margin of clypeus thick and not protruding forwards (Fig. 713); width of hypoclypeal depression 0.4 × minimum width of face (Fig. 711); length of eye twice temple in dorsal view (Fig. 712); vertex behind stemmaticum flattened and punctate-rugose; clypeus between eyes; length of malar space 0.3 × length of eye in lateral view.

***Mesosoma.*** Mesoscutal lobes finely punctate with very finely granulate interspaces, rather matt; precoxal area of mesopleuron smooth, mesopleuron densely punctate posteriorly; scutellum coarsely punctate and rather flat; propodeum rather flattened medially, very coarsely reticulate-rugose, medio-longitudinal carina nearly complete, and with small crest-like protuberances laterally.

***Wings.*** Fore wing: r 0.6 × 3-SR (Fig. 704); 1-CU1 horizontal, 0.4 × 2-CU1; r-m 0.9 × 3-SR; 2^nd^ submarginal cell short (Fig. 704); cu-a inclivous, straight; 1-M nearly straight posteriorly; 1-SR wide; surroundings of M+CU1, 1-M and 1-CU1 largely setose. Hind wing: basal 0.6 of marginal cell subparallel-sided and remainder linearly widened, its apical width 1.8 × width at level of hamuli (Fig. 704); 2-SC+R subquadrate; m-cu absent; M+CU:1-M = 37:28; 1r-m 0.8 × 1-M.

***Legs.*** Tarsal claws with conspicuous and robust blackish pecten (Fig. 716); hind coxa largely densely and coarsely punctate; hind trochantellus rather robust; length of hind femur and basitarsus 3.9 and 5.0 × their width, respectively; length of inner hind spur 0.5 × hind basitarsus.

***Metasoma.*** First tergite moderately convex, 0.9 × longer than wide apically; 1^st^ and 2^nd^ tergites with coarse medio-longitudinal carina and very coarsely and irregularly longitudinally rugose; medio-basal area of 2^nd^ tergite large and distinct (Fig. 707); 2^nd^ suture deep, rather wide and coarsely crenulate; basal half of 3^rd^ tergite punctate-rugose, remainder of metasoma finely punctate; 4^th^ and apical half of 3^rd^ tergite without sharp lateral crease; ovipositor sheath wide, with rather long setae and apically narrowed and rounded (Fig. 702).

***Colour.*** Black (including fore and middle telotarsi, apical half of hind tibia and hind tarsus); basal half of hind tibia pale yellowish; palpi (but basally somewhat infuscate) and remainder of legs reddish brown; tegulae yellowish brown; hind tibial spurs and pterostigma dark brown; veins brown; mesopleuron with broad dark reddish longitudinal band; wing membrane slightly infuscate.

***Variation.*** Micro-sculpture of lateral lobes of mesoscutum very finely granulate or absent and resulting in a largely smooth surface; maximum width of marginal cell of hind wing 1.5–2.3 × its width near hamuli; body entirely black (both sexes) or 1^st^ and 2^nd^ metasomal tergites and mesosoma partly dark reddish (both sexes, but 2^nd^ and 3^rd^ tergites of males more or less darkened); hind tibial spurs dark brown or reddish brown. Antennal segments: ♀ 60(4), 61(7), 62(10), 63(18), 64(14), 65(2), 66(8), 67(4), 68(3), 69(1); ♂ 53(1), 56(2), 57(1), 58(7), 59(6), 60(15), 61(15), 62(19), 63(28), 64(12), 65(10), 66(4), 67(2), 68(1). Females have on average ca one more antennal segment than males. Male is similar to the dark female form, with acute hind tibial spurs (Fig. 722), 3^rd^ tergite convex in lateral view (Fig. 720), with dense and long setosity and apical tergites type 1 and no fringe observed (Figs 721, 724).

**Figures 701–703.**
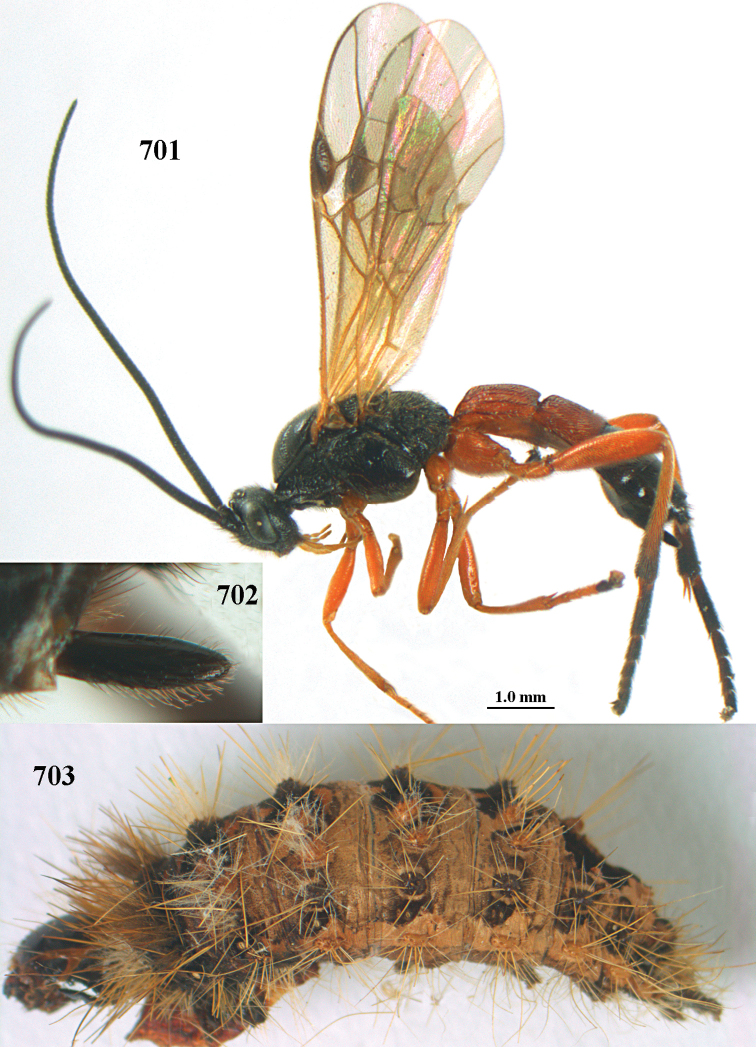
*Aleiodes rugulosus* (Nees), ♀, U.K., culture **701** habitus lateral **702** ovipositor sheath lateral **703** mummy of *Acronicta rumicis* (Linnaeus).

*Aleiodes rugulosus* is a very colour-variable species; the 1^st^ and 2^nd^ metasomal tergites are quite frequently mostly or entirely dark red or orange brown (Fig. 701) in populations in which entirely black females (Fig. 717) also often occur. The variation is not geographical, since most populations definitely have both forms. In rearing experiments, a strong genetic basis for this feature became evident: from a virgin female with completely black metasoma, all five males reared were black, and a cross between one of them and a (wild-reared) black female produced eleven female progeny, all black. In contrast, a lineage from a pairing between a weakly red male and a red female comprised three red males and eight red females, separate individuals of which produced a single red and a single black male as progeny. When sufficient material from single sites is available it is usual to see a clear predominance of one form or the other. Sometimes the lower part of mesopleuron is pale (often looking unpigmented there, but narrowly), and in extreme reddish specimens the scutellum, much of the mesopleuron (but usually the mesosternum remains darkish), the metanotum dorsally, the metapleuron in part and much of the propodeum are also reddish.

##### Distribution.

Albania, Austria, Belgium, Bulgaria, Czech Republic, British Isles (England, Wales, Scotland, Ireland), Finland, France, Germany, Hungary, *Netherlands, *North Macedonia, *Moldova. Norway, Poland, *Romania, Russia, *Slovakia, *Spain, Sweden.

**Figures 704–716.**
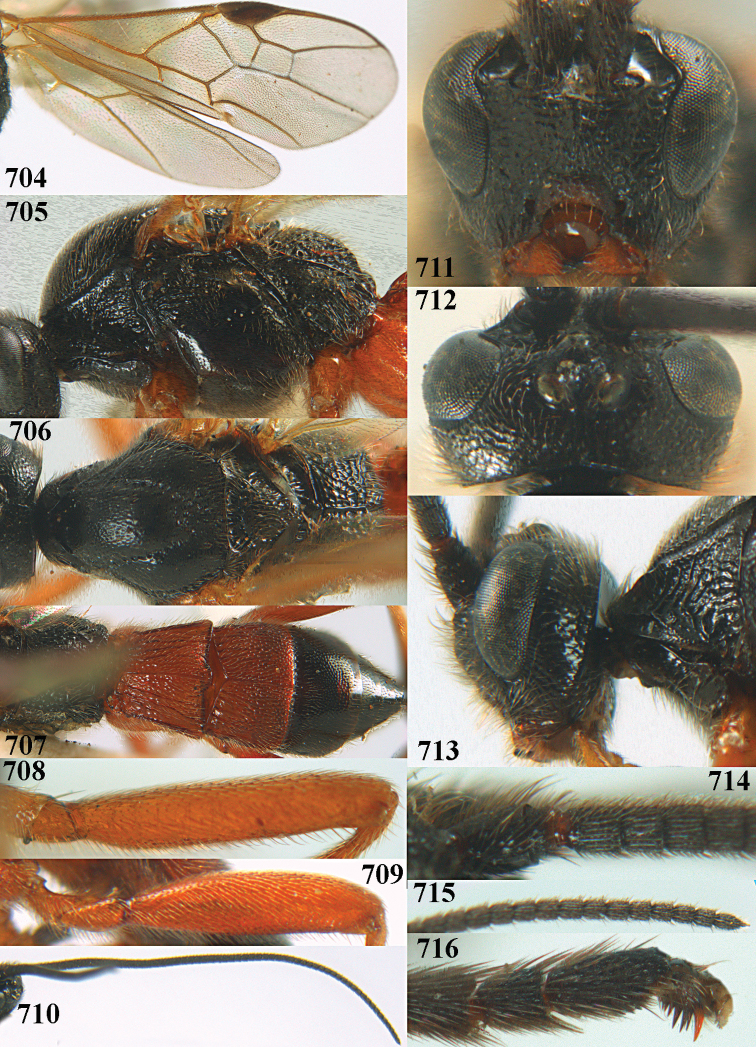
*Aleiodes rugulosus* (Nees), ♀, U.K., culture **704** wings **705** mesosoma lateral **706** mesosoma dorsal **707** propodeum and 1^st^–3^rd^ metasomal tergites dorsal **708** fore femur lateral **709** hind femur lateral **710** antenna **711** head anterior **712** head dorsal **713** head lateral **714** base of antenna **715** apex of antenna **716** outer hind tarsal claw.

**Figures 717, 718.**
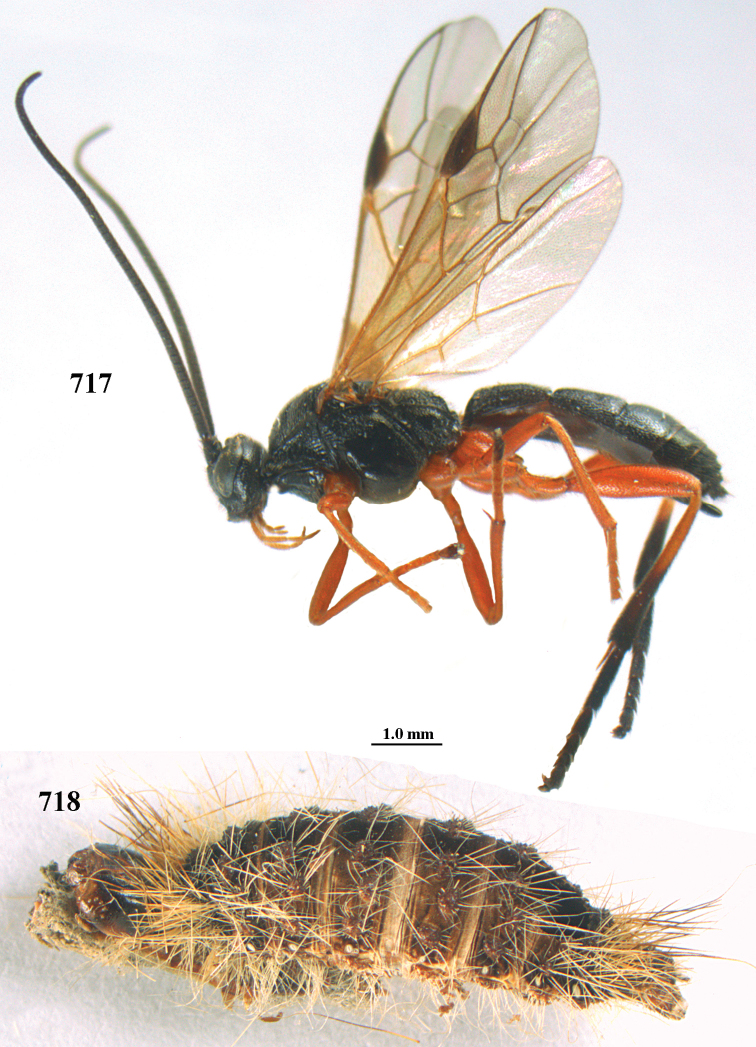
*Aleiodes rugulosus* (Nees), dark form, ♀, U.K., Meathop Moss **717** habitus lateral **718** mummy of *Acronicta menyanthidis* (Esper).

**Figure 719.**
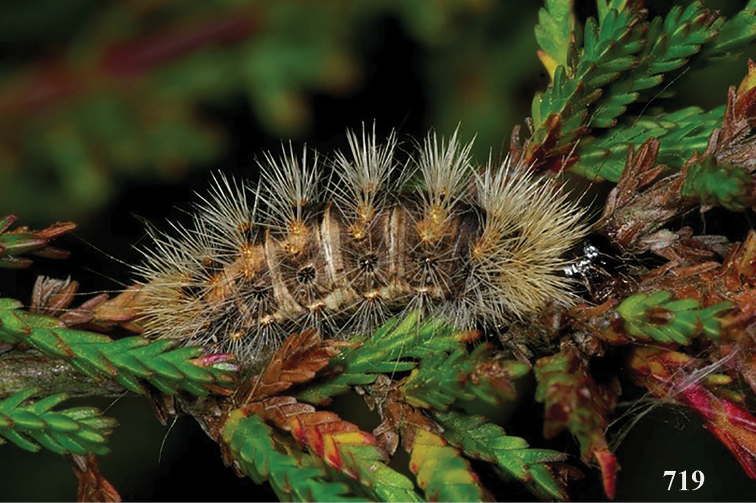
*Aleiodes rugulosus* (Nees), Netherlands, Ede, mummy of *Acronicta auricoma* (Denis & Schiffermüller). Photograph: J. Voogd.

**Figures 720–727.**
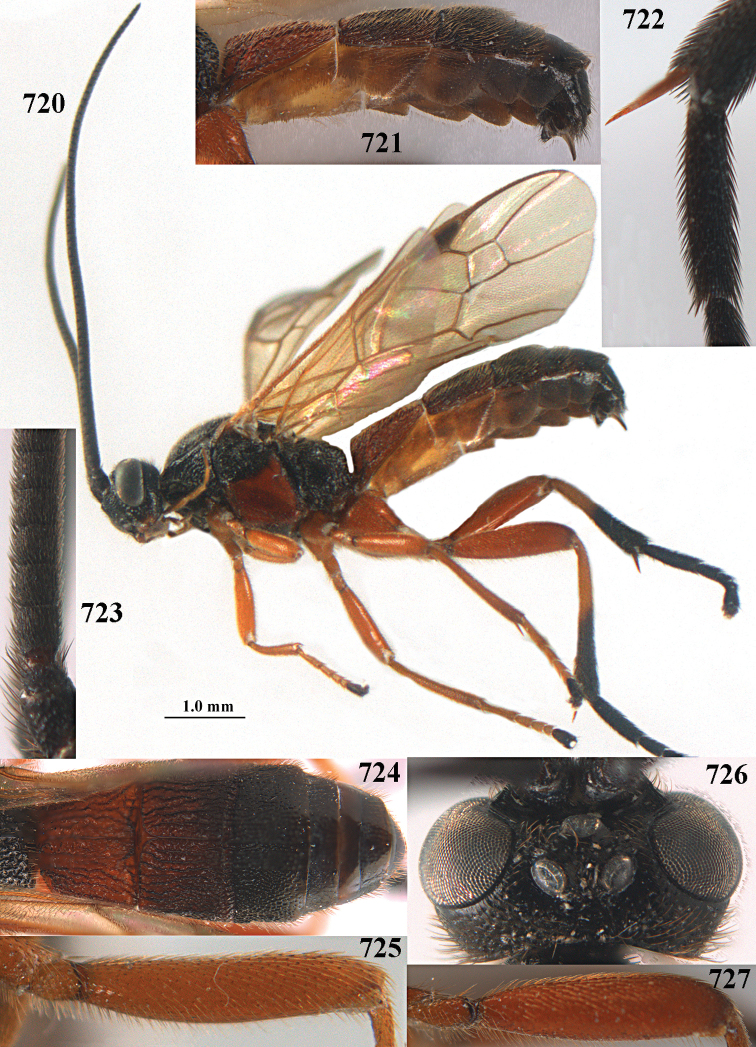
*Aleiodes rugulosus* (Nees), ♂, Hungary (Halastó) **720** habitus lateral **721** metasoma lateral **722** hind tibial spurs and basitarsus lateral **723** base of antenna **724** metasoma dorsal **725** fore femur lateral **726** head dorsal **727** hind femur lateral.

#### Aleiodes schewyrewi

Taxon classificationAnimaliaHymenopteraBraconidae

(Kokujev, 1898)

D9F506D0-8EC9-55FD-89A7-EA36438530AA

Figs 728–729 , 730–742

Rhogas (Rhogas) schewyrewi Kokujev, 1898: 304.
Rhogas (Rhogas) schevyrevi [sic!]; Telenga 1941: 186.
Rogas schewyrewi ; Shenefelt 1975: 1248
Rogas (Rogas) schewyrewi ; Papp 1977b: 116.
Aleiodes (Chelonorhogas) schewyrewi ; Belokobylskij 2000: 46.
Rhogas (Rhogas) schewyrewi var. zaydamensis Kokujev, 1898: 305; Telenga 1941: 186 [examined].
Rogas schewyrewi var. zaydamensis ; Shenefelt 1975: 1248–1249.
Rogas (Aleiodes) schewyrewi var. zaydamensis ; Papp 1977b: 116, 117.

##### Type material.

Holotype of *A. schewyrewi zaydamensis*, ♀ (ZISP), “[**Mongolia**], Kerijsk Kr., Ruio Zaydam, Przewalski”, “1910a”, [illegible handwritten label], “*Rh. Schewyrewi* Kokw. var. *zaydamensis* Kokw., No.1910a”, “♀ *Rhogas schewyrewi* var. *zaydamensis* Kok., C. van Achterberg, 1992, holotype”. Holotype of *A. s. schewyrewi* not found, according to the original description with same label data and with a larger part of the body blackish.

##### Additional material.

1 ♀ (BZL), “S. **Russia**, [Volgograd obl.], Elton Lake env., 20.v.2001, J. Miatleuski”; 1 ♂ (MTMA), “Mongolia, Gobi Altay aimak, Mongol els, 10 km SO von Somon Chechmort, 1600 m, Exp. Dr. Z. Kaszab, 1966”, “Nr. 684, 13.vii.1966”, “*Rogas schewyrewi* Kok., det. Papp J., 1977/ compared with ♀ det. Kokujev, Papp, 1983, 57”.

##### Molecular data.

None.

##### Biology.

Unknown. A female collected in May and a male in July may suggest that it is plurivoltine (or, less probably, that the female overwinters as an adult).

##### Diagnosis.

**Figures 728, 729.**
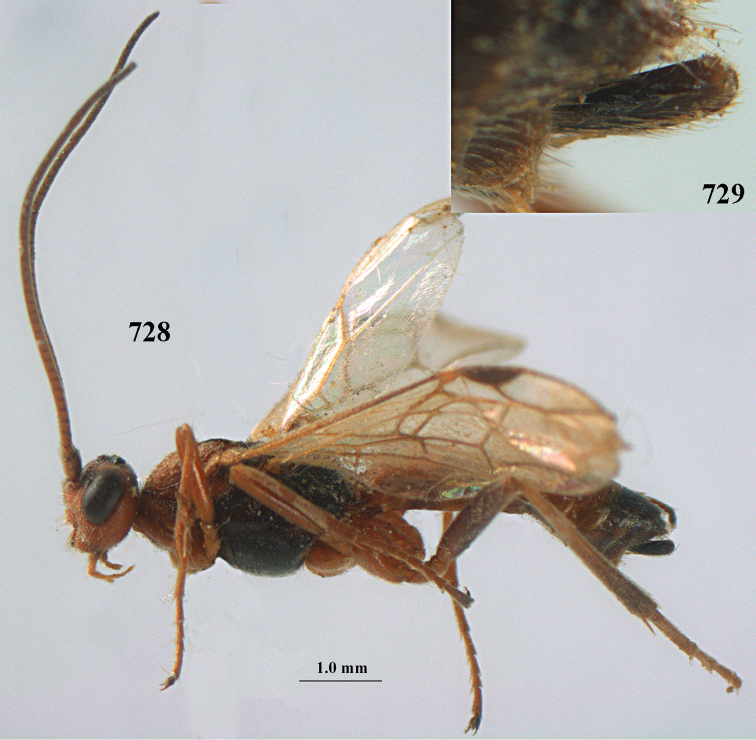
Aleiodes schewyrewi (Kokujev) , ♀, holotype var. zaydamensis (Kokujev) **728** habitus lateral **729** ovipositor sheath lateral.

**Figures 730–742.**
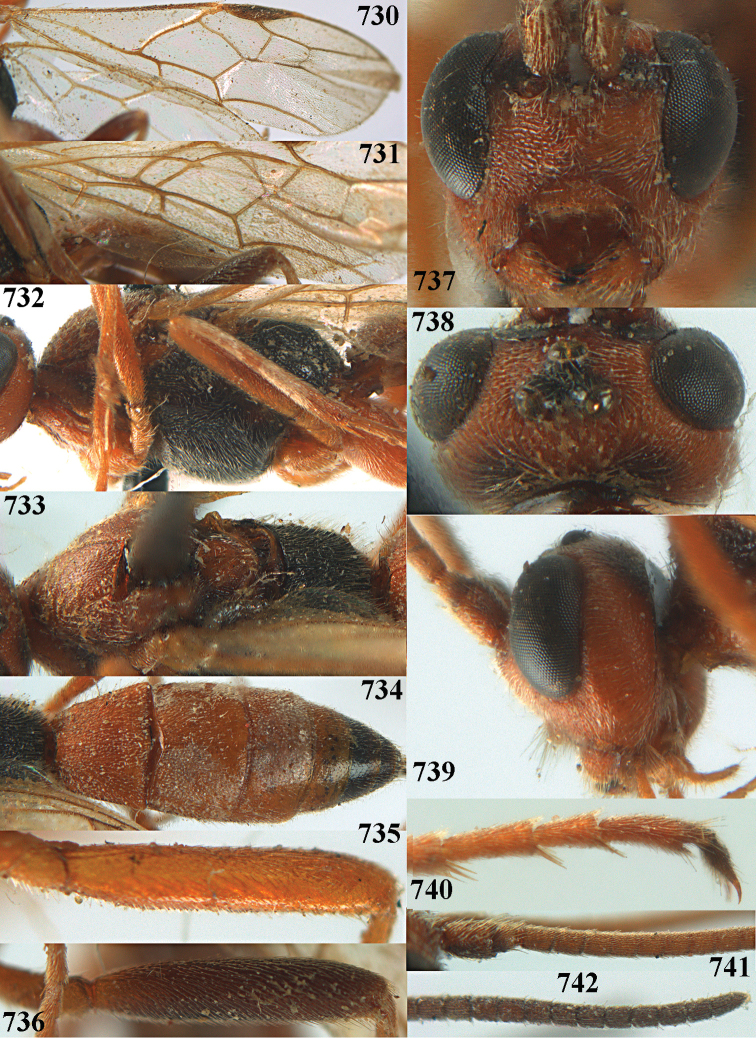
Aleiodes schewyrewi (Kokujev) , ♀, holotype var. zaydamensis (Kokujev) **730** fore wing **731** hind wing **732** mesosoma lateral **733** mesosoma dorsal **734** metasoma dorsal **735** fore femur lateral **736** hind femur lateral **737** head anterior **738** head dorsal **739** head lateral **740** outer hind tarsal claw **741** base of antenna **742** apex of antenna.

Maximum width of hypoclypeal depression 0.6–0.7 × minimum width of face (Fig. 737); OOL of ♀ approx. as long as diameter of posterior ocellus and densely rugose (Fig. 738); head in anterior view rather robust (Fig. 737); clypeus distinctly protruding anteriorly in lateral view, thick apically and with long setae on medium-sized anterior part (Fig. 739); lobes of mesoscutum largely superficially punctate, interspaces finely granulate or smooth and with satin sheen; precoxal area densely rugose, but posterior third only finely punctate; vein 1-CU1 of fore wing 0.3 × vein 2-CU1 and 0.5 × vein m-cu (Fig. 730); hind tarsal claws long and slender, nearly straight and only brownish bristly setose (Fig. 740); tarsal segments (except telotarsus) with long apical spiny bristles (Fig. 728); basal half of hind tibia pale yellowish, contrasting with dark brown colour of basal half of hind femur.

##### Description.

Holotype, ♀, length of fore wing 5.8 mm, of body 7.5 mm.

***Head.*** Antennal segments of ♀ 48, length of antenna 1.1 × fore wing, its subapical segments moderately slender (Fig. 742); frons largely with fine curved rugae; OOL equal to diameter of posterior ocellus, and densely rugose; vertex superficially rugose-punctate, rather shiny; clypeus convex and densely punctate; ventral margin of clypeus thick and protruding forwards (Fig. 740); width of hypoclypeal depression 0.6 × minimum width of face (Fig. 737); length of eye 1.3 × temple in dorsal view (Fig. 738); vertex behind stemmaticum finely rugose-punctate and with long setae; clypeus largely above lower level of eyes; length of malar space 0.3 × height of eye in lateral view.

***Mesosoma.*** Mesoscutal lobes largely superficially punctate, interspaces finely granulate and with satin sheen; precoxal area of mesopleuron largely smooth medially, densely punctate anteriorly and posteriorly densely rugose, but posterior 0.3 only finely punctate; metapleuron spaced coarsely punctate; metanotum with fine and nearly complete median carina; scutellum sparsely punctate, shiny; propodeum rather convex and rather coarsely rugose, medio-longitudinal carina on anterior 0.4 of propodeum.

***Wings.*** Fore wing: r curved and 0.4 × 3-SR (Fig. 730); 1-CU1 horizontal and slightly widened, 0.3 × 2-CU1; r-m 0.7 × 3-SR; 2^nd^ submarginal cell medium-sized (Fig. 730); cu-a slightly inclivous, straight; 1-M slightly curved; 1-SR slender; surroundings of M+CU1, 1-M and 1-CU1 setose. Hind wing: basal third of marginal cell subparallel-sided and remainder linearly widened; 2-SC+R short and longitudinal; m-cu slightly indicated; M+CU:1-M = 10:7; 1r-m 0.6 × 1-M.

***Legs.*** Tarsal claws long and slender, nearly straight and only brownish bristly setose (Fig. 740); tarsal segments (except telotarsus) with long apical spiny bristles (Figs 728, 740); hind coxa largely punctate, but dorsally punctate-rugose; hind trochantellus rather robust; length of hind femur and basitarsus 4.7 and 6.6 × their width, respectively; length of inner hind spur 0.4 × hind basitarsus.

***Metasoma.*** First tergite evenly convex and strongly widened posteriorly, 0.9 × longer than wide apically; 1^st^ and 2^nd^ tergites with weak medio-longitudinal carina (absent posteriorly) and finely longitudinally rugose, but 2^nd^ tergite smooth medio-posteriorly; medio-basal area of 2^nd^ tergite wide triangular and rather distinct (Fig. 734); 2^nd^ suture deep and narrow; basal half of 3^rd^ tergite aciculate, remainder of metasoma smooth and shiny; 2^nd^ and 3^rd^ tergites with sharp lateral crease; ovipositor sheath moderately widened, with medium-sized setae and apically truncate (Fig. 729).

***Colour.*** Yellowish brown (including basal half of antenna); apical half of antenna, frons largely, stemmaticum, occiput dorso-laterally, pronotal side medially, axilla, mesopleuron (except antero-dorsally), mesosternum, metapleuron, propodeum, hind femur and apical third of hind tibia, 5^th^–7^th^ tergites, last two posterior sternites, ovipositor sheath and pterostigma dark brown or blackish brown; veins brown; wing membrane subhyaline.

***Variation.*** Length of 4^th^ hind tarsal segment 1.8–2.0 × longer than wide; malar space and temple ventrally largely dark brown or yellowish brown. Antennal segments: ♀ 45(1); ♂ 54(1); according to original description ♀ type has 58 segments. Male has clypeus yellowish and contrasting with black face, apical tergites type 1, and no fringe observed.

##### Distribution.

*Iran, Mongolia, *Russia (European part).

#### Aleiodes schirjajewi

Taxon classificationAnimaliaHymenopteraBraconidae

(Kokujev, 1898)

ACE6E3C7-C81F-5A63-AE5A-C438590A3E56

Figs 743 , 744–757

Rhogas reticulator var. schirjajewi Kokujev, 1898: 299 [examined].
Rogas schirjajewi ; Shenefelt 1975: 1249.
Rogas (Rogas) schirjaevi [sic!]; Tobias 1976: 85.
Rogas (Rogas) schirjaewi [sic!]; Tobias 1986: 80 (transl.: 132).
Aleiodes (Neorhogas) schirjajewi ; Papp 1991a: 71, 2002: 562.
Aleiodes (Chelonorhogas) schirjajewi ; Samartsev and Belokobylskij 2013: 766.
Aleiodes schirjajewi ; Shaw et al. 1998: 63; Papp 2005: 177.

##### Type material.

Holotype, ♂ (ZISP), “[**Kazakhstan**], Kemropavl., Akmolin, 908a”, “K. Kokujeva”, “908a, *Rh. reticulator* Nees v. *schirjajewi* Kokw.”, “Holotypus”.

##### Additional material.

Bulgaria, Hungary, Italy, Moldova, Russia, Serbia, Ukraine [Dagestan, Kazakhstan]. Specimens in BZL, ZJUH, MRC, MSC, MTMA, NMS, RMNH, SDEI, ZISP.

##### Molecular data.

None.

##### Biology.

Unknown. Specimens collected throughout April–September, presumably plurivoltine. We have not seen reared material and it is unclear how the winter is passed.

**Diagnosis.** Maximum width of hypoclypeal depression 0.3–0.4 × minimum width of face (Fig. 751); length of antenna of ♀ 1.1–1.4 × fore wing; ventral margin of clypeus thick and obtuse apically and clypeus not protruding in lateral view (Fig. 753); vertex and frons with strong striae or rugae; mesoscutum, metapleuron and scutellum normally shiny and without dense granulation, at most with some superficial micro-sculpture; precoxal area of mesopleuron smooth; vein 2-CU1 of fore wing approx. as long as vein 1-CU1 or shorter (Fig. 744); vein M+CU of hind wing distinctly longer than vein 1-M (Fig. 745); hind tarsal claws with medium-sized dark brown pecten (Fig. 756); head black; mesoscutum and scutellum orange brown; fore and middle femora distinctly black or dark brown apically; basal half of hind tibia dark brown; anterior half of mesosoma, 1^st^ and 2^nd^ metasomal tergites yellowish or orange brown; at least basal half of 4^th^–6^th^ tergites of ♂ with long and dense setosity.

##### Description.

Holotype, ♂, length of fore wing 5.0 mm, of body 5.8 mm.

***Head.*** Antennal segments of ♂ 50, length of antenna 1.3 × fore wing, its subapical segments rather robust; 4^th^ segment of maxillary palp slender and cylindrical; frons with rather coarse curved rugae and interspaces smooth; OOL 1.1 × diameter of posterior ocellus, coarsely rugose and shiny; coarsely transversely rugose and shiny; clypeus punctate-rugulose; ventral margin of clypeus thick and not protruding forwards (Fig. 753); width of hypoclypeal depression 0.4 × minimum width of face (Fig. 751); length of eye 3.2 × temple in dorsal view (Fig. 752); vertex behind stemmaticum coarsely rugose; clypeus below lower level of eyes; occipital carina complete; length of malar space 0.4 × length of eye in lateral view.

***Mesosoma.*** Mesoscutal lobes finely punctate, shiny, interspaces micro-sculptured; precoxal area of mesopleuron smooth medially except for some crenulations and punctures, its surroundings smooth; scutellum moderately punctate; propodeum rather convex and coarsely reticulate, coarse medio-longitudinal carina present anteriorly, carinae not protruding laterally.

***Wings.*** Fore wing: r 0.7 × 3-SR (Fig. 744); 1-CU1 horizontal, as long as 2-CU1; r-m 0.9 × 3-SR; 2^nd^ submarginal cell short (Fig. 744); cu-a vertical, largely straight; 1-M nearly straight posteriorly; 1-SR slender; surroundings of M+CU1, 1-M and 1-CU1 setose. Hind wing: marginal cell linearly widened, its apical width 2.2 × width at level of hamuli (Fig. 745); 2-SC+R subquadrate; m-cu absent; M+CU:1-M = 15:11; 1r-m 0.7 × 1-M.

***Legs.*** Tarsal claws with medium-sized dark brown pecten (Fig. 756); hind coxa largely densely punctate; hind trochantellus medium-sized; length of hind femur and basitarsus 4.0 and 7.0 × their width, respectively; length of inner hind spur 0.5 × hind basitarsus.

***Metasoma.*** First tergite moderately flattened, 0.9 × longer than wide apically; 1^st^ and 2^nd^ tergites with medio-longitudinal carina and densely vermiculate-rugose; medio-basal area of 2^nd^ tergite wide triangular and distinct (Fig. 748); 2^nd^ suture deep and moderately crenulate; 3^rd^ tergite finely striate basally, remainder of metasoma largely smooth; 4^th^ without sharp lateral crease; basal half of 4^th^–6^th^ tergites of ♂ with long and dense setosity.

***Colour.*** Orange brownish; basal half of antenna, palpi largely and parastigma (except base) yellowish brown; head, mesosternum (except anteriorly), mesopleuron (except anteriorly and antero-dorsally), apical 0.4 of hind femur, 3^rd^ tergite (except antero-laterally) and subsequent tergites black; scapus, pedicellus basally, apical half of antenna, apex of fore and middle femora, apex of middle and hind tibiae, hind basitarsus, 2^nd^ hind tarsal segment apically, telotarsi, pterostigma, parastigma basally and veins dark brown; wing membrane subhyaline.

***Variation.*** Vein 1-CU1 of fore wing 1.0–2.3 × vein 2-CU1; maximum width of marginal cell of hind wing 1.6–2.6 × its width near hamuli (Fig. 745). Antennal segments: ♀ 46(1), 48(3), 49(1), 50(2), 51(1); ♂ 47(1), 48(2), 49(2), 50(1), 51(1). The sexes have comparable numbers of antennal segments. Apical tergites of ♂ type 4, dense, making the tergites look concave and fringe not observed. Female is very similar to the redescribed male; ovipositor sheath wide, with long setae and apically truncate.

##### Distribution.

*Bulgaria, Hungary, *Italy, Kazakhstan, Moldova, Russia (including Dagestan and Far East), Serbia, Ukraine.

**Figure 743.**
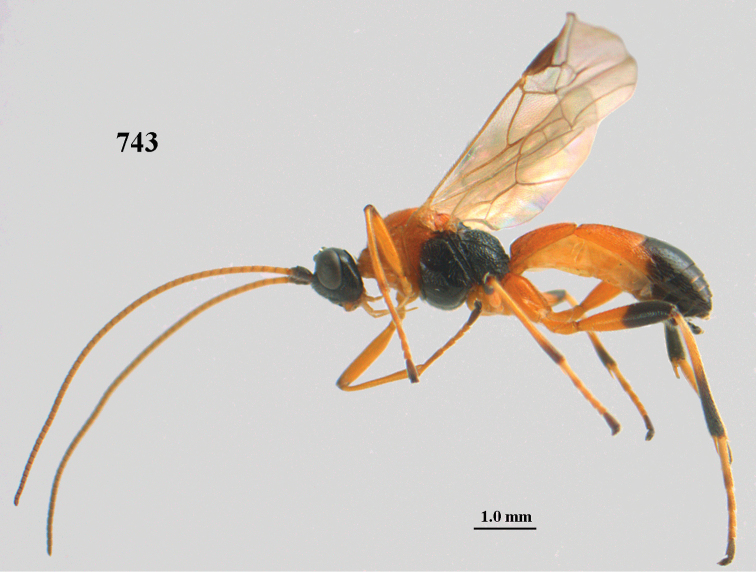
*Aleiodes schirjajewi* (Kokujev), ♀, Hungary, Budapest, habitus lateral.

**Figures 744–757.**
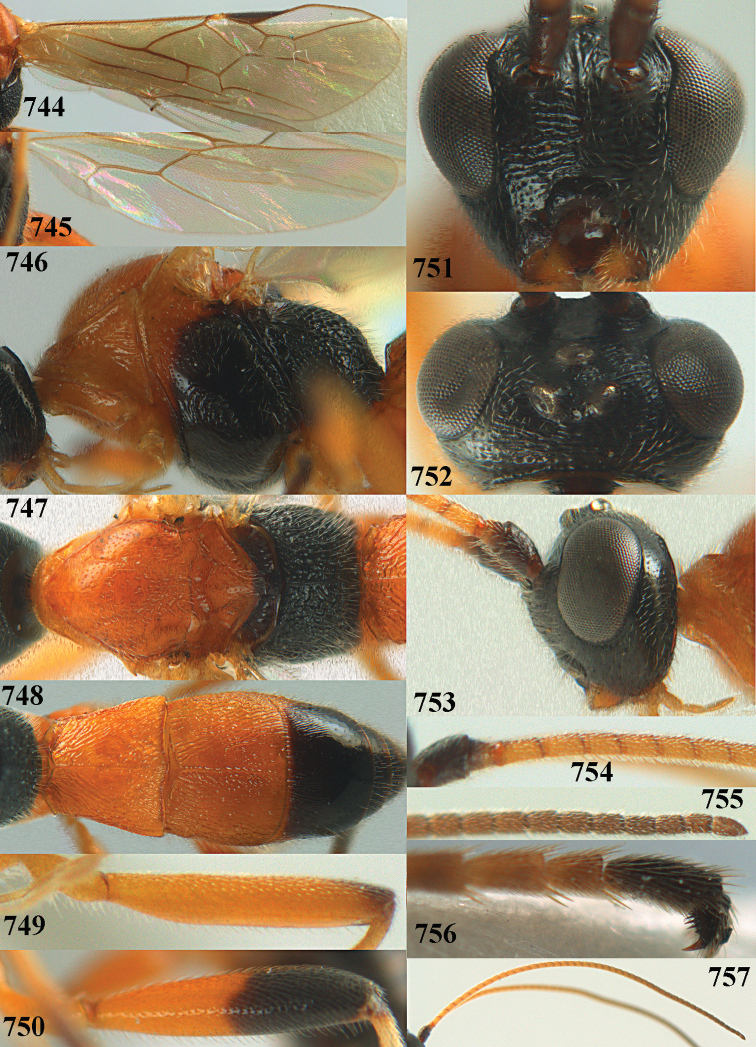
*Aleiodes schirjajewi* (Kokujev), ♀, Hungary, Budapest, but 756 ♀, Ukraine **744** fore wing **745** hind wing **746** mesosoma lateral **747** mesosoma dorsal **748** metasoma dorsal **749** fore femur lateral **750** hind femur lateral **751** head anterior **752** head dorsal **753** head lateral **754** base of antenna **755** apex of antenna **756** outer hind tarsal claw **757** antenna.

#### Aleiodes sibiricus

Taxon classificationAnimaliaHymenopteraBraconidae

(Kokujev, 1903)

CDFDDC7E-C46C-5695-881E-D661D6998B63

Figs 758–759 , 760–762 , 763–776

Rhogas sibiricus Kokujev, 1903: 286 [examined].
Rogas sibiricus ; Shenefelt 1975: 1250.
Rogas (Rogas) sibiricus ; Tobias 1976: 83, 84, 1986: 76, 78 (transl.: 124, 128; lectotype designation).
Aleiodes (Neorhogas) sibiricus ; Papp 1985a: 150, 153, 162, 1991a: 92; Belokobylskij 1996: 15.
Aleiodes sibiricus ; Papp, 2005: 177.
Rhogas hungaricus Szépligeti, 1906: 616; Papp 1985a: 150, 153, 162 (as synonym of A. sibiricus; lectotype designation), 2005: 177 (id.); 2004: 216 (id.) [examined].
Rogas hungaricus ; Shenefelt 1975: 133.
Rhogas reinhardi Fahringer, 1931: 221 (description in key only), 1932: 275 (full description; as R. rheinhardi); Papp 1985a: 153, 162 (as synonym of A. sibiricus; holotype examined), 2005: 177 (id.).

##### Type material.

Paralectotype of *A. sibiricus*, ♀, (ZJUH), “[**Russia**], Irkutsk, v., I. Jakovlev”, “K. Kokujeva”, “Paratypus *Rogas sibiricus* Kokujev”, “Rec. in exchange [from] Academy of Science, Leningrad, BM.1963.211”, “Ant. 69”. Lectotype of *A. hungaricus*, ♀, (MTMA), “[**Hungary**], Budapest, Szépligeti”, “Lectotypus *Rhogas hungaricus* Szépl. 1906, ♀, Papp, 1966”, “Hym. Typ. No. 401, Mus. Budapest”, “*Aleiodes sibiricus* Kok., ♀, det. Papp J., 1983/compared with ♀ paralectotype”.

##### Additional material.

Albania, Austria, Bulgaria, France, Germany, Greece, Hungary, Italy, North Macedonia, Sweden, Turkey. Specimens in ZJUH, BZL, MTMA, NMS, RMNH, ZSSM.

##### Molecular data.

MRS310 (Sweden), MRS313 (Sweden), MRS805 (France).

##### Biology.

Collected in April and May, and presumably univoltine, but 2 ♀ from Sweden: Ångermanland, Lillavammasjon were collected apparently in July in window traps set on the trunks of *Betula* and *Picea*. We have examined four males collected in April which suggests spring emergence from the mummy rather than overwintering as an adult. This is corroborated by the data with the single reared specimen examined (MTMA), from the noctuid *Noctua comes* Hübner collected 7.iv.1961 and emerging on 3.iv.1962 (Germany; [R.] Hinz). The rearer was widely experienced with caterpillars, and the host determination is unlikely to be wrong (the other caterpillar species with which it might conceivably be confused all have similar biology and phenology in any case). This host initiates its overwintering as a small larva, feeding in mild weather through the winter and normally being well-grown by April, by then in its penultimate or final instar. The rearing is of great interest because it shows that *A. sibiricus*, like *A. fortipes* (q. v.), not only parasitises a host that has overwintered as a larva, but also must habitually attack late instar hosts. The reared specimen is accompanied by a stout mummy, large but not unduly so for the size of the adult that emerged, lacking its anterior portion to leave a partitioned chamber comprising abdominal segments 4 onwards, which is well lined with silk and would presumably normally form in the soil (Fig. 762).

##### Diagnosis.

Maximum width of hypoclypeal depression 0.5–0.6 × minimum width of face (Figs 759, 770); antenna of ♀ with 65–72 segments and 5^th^–10^th^ segments wider than long; anterior part of clypeus short and transverse, its height 0.2–0.3 × height of hypoclypeal depression (Fig. 770); ventral margin of clypeus rather thin and slightly protruding in lateral view (Fig. 772); mesoscutal lobes densely punctate and interspaces smooth; precoxal area (rather) coarsely vermiculate-rugose medially; length of vein r of fore wing 0.3–0.5 × vein 3-SR (Fig. 763); vein 1-CU1 horizontal and 0.2–0.3 × vein 2-CU1; hind tarsus and claws slender and claws with inconspicuous brownish teeth (Fig. 775); 4^th^ and 5^th^ metasomal tergites more or less yellowish to reddish brown; head, mesoscutum, scutellum, mesopleuron and apex of metasoma black.

##### Description.

Paralectotype of *A. sibiricus*, ♀, length of fore wing 9.1 mm, of body 10.0 mm.

***Head.*** Antennal segments of ♀ 69, antenna as long as fore wing, its subapical segments medium-sized; frons smooth; OOL 1.1 × diameter of posterior ocellus, and finely coriaceous-rugulose; vertex rugulose and rather dull; clypeus coriaceous and strongly transverse (4–6 × wider than high; Figs 759, 770); ventral margin of clypeus rather thin and slightly protruding forwards (Fig. 772); width of hypoclypeal depression 0.5 × minimum width of face (Fig. 770); length of eye 1.1 × temple in dorsal view (Fig. 771); vertex behind stemmaticum rugulose; clypeus near lower level of eyes; length of malar space 0.3 × length of eye in lateral view.

***Mesosoma.*** Mesoscutal lobes densely punctate and interspaces smooth, rather matt; precoxal area of mesopleuron rugose medially and anteriorly, its surroundings moderately punctate; scutellum sparsely punctate and no lateral carina; propodeum rather convex and densely and finely rugose, medio-longitudinal carina complete and no protruding carinae laterally.

***Wings.*** Fore wing: r 0.3 × 3-SR (Fig. 763); 1-CU1 horizontal, 0.2 × 2-CU1; r-m unsclerotized, 0.7 × 3-SR; 2^nd^ submarginal cell rather short (Fig. 763); cu-a inclivous, straight; 1-M nearly straight posteriorly; 1-SR wide; surroundings of M+CU1, 1-M and 1-CU1 setose. Hind wing: basal half of marginal cell slightly widened, but apical half distinctly linearly widened, its apical width 2.5 × width at level of hamuli (Fig. 764); 2-SC+R subquadrate; m-cu slightly indicated; M+CU:1-M = 51:38; 1r-m 0.7 × 1-M.

***Legs.*** Tarsal claws with four inconspicuous brownish pecten-teeth (Fig. 775); hind coxa punctulate; hind trochantellus robust; length of hind femur and basitarsus 4.1 and 6.0 × their width, respectively; length of inner hind spur 0.4 × hind basitarsus.

***Metasoma.*** First tergite rather flattened, 0.8 × longer than wide apically; 1^st^ and 2^nd^ tergites with weak medio-longitudinal carina and densely finely rugose, but posterior quarter of 2^nd^ tergite irregularly rugose and no median carina; medio-basal area of 2^nd^ tergite wide and short (Fig. 767); 2^nd^ suture deep, rather wide medially and finely crenulate; basal half of 3^rd^ tergite finely rugose, remainder of metasoma superficially micro-sculptured; 4^th^ and apical half of 3^rd^ tergite without sharp lateral crease; ovipositor sheath wide, with long setae and apically truncate (Fig. 761).

***Colour.*** Black; palpi and tegulae pale yellowish; legs (except black coxae; apex of hind femur dorsally, inner side of hind tibia apically (not outer side!) and telotarsi infuscated), apex of first tergite, 2^nd^–5^th^ tergites and metasoma ventrally, yellowish brown; ovipositor sheath largely, pterostigma and most veins dark brown; vein 1-R1 of fore wing yellowish brown; wing membrane subhyaline.

***Variation.*** Face, clypeus, mesoscutum, propleuron, upper part of mesopleuron, and first tergite partly, or rarely entirely, reddish brown. Usually in males and rarely in females mesoscutum wholly black; vein r of fore wing 0.3–0.5 × vein 3-SR; clypeus flattened and subparallel-sided or convex and ventrally concave; pterostigma medially dark brown or yellowish brown. Antennal segments ♀: 65(4), 66(8), 67(6), 68(4), 69(2), 70(5), 71(2), 72(2); ♂ 66(1), 71(1). Male apical tergites of type 1 and fringe not observed.

##### Distribution.

*Albania, *Austria, *Bulgaria, *France, Germany, *Greece, Hungary, Italy (main), *North Macedonia, Russia (Siberia), *Sweden, *Turkey.

##### Notes.

The holotype of *A. reinhardi* (Fahringer, 1931) from Bolzano (N Italy) was examined by Papp (1985a) and directly compared with the lectotype of *A. hungaricus*. Unfortunately, the holotype could not be located in NHMW, but there is no obvious reason not to follow the synonymy with *A. sibiricus* (Kokujev) proposed by Papp (1985a). *Aleiodes agilis* (Telenga, 1941) from China, Iran, and Caucasus is very similar to *A. sibiricus*, but *A. agilis* has antenna of ♀ with ca 48 segments (69–72 segments in *A. sibiricus*), 2^nd^ tergite narrowly smooth posteriorly (finely sculptured), pronotum largely yellow (black), clypeus not protruding in lateral view (somewhat protruding) and is often smaller (body length 7–8 mm *vs* 7–11 mm).

**Figures 758, 759.**
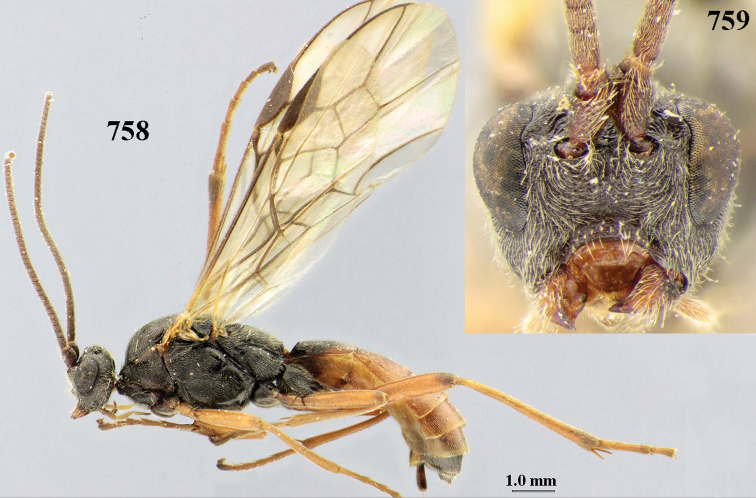
*Aleiodes sibiricus* (Kokujev), ♀, lectotype **758** habitus lateral **759** head anterior. Photographs: K. Samartsev.

**Figures 760–762.**
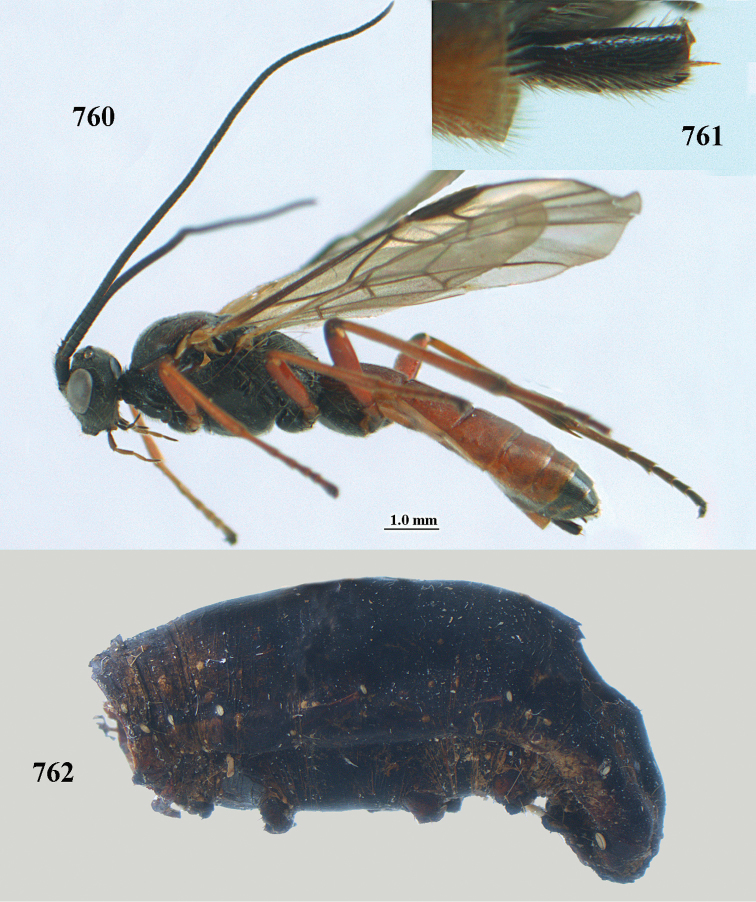
*Aleiodes sibiricus* (Kokujev), ♀, Hungary, Heves, but 762 Germany, Freiburg **760** habitus lateral **761** ovipositor sheath lateral **762** mummy of *Noctua comes* Hübner.

**Figures 763–776.**
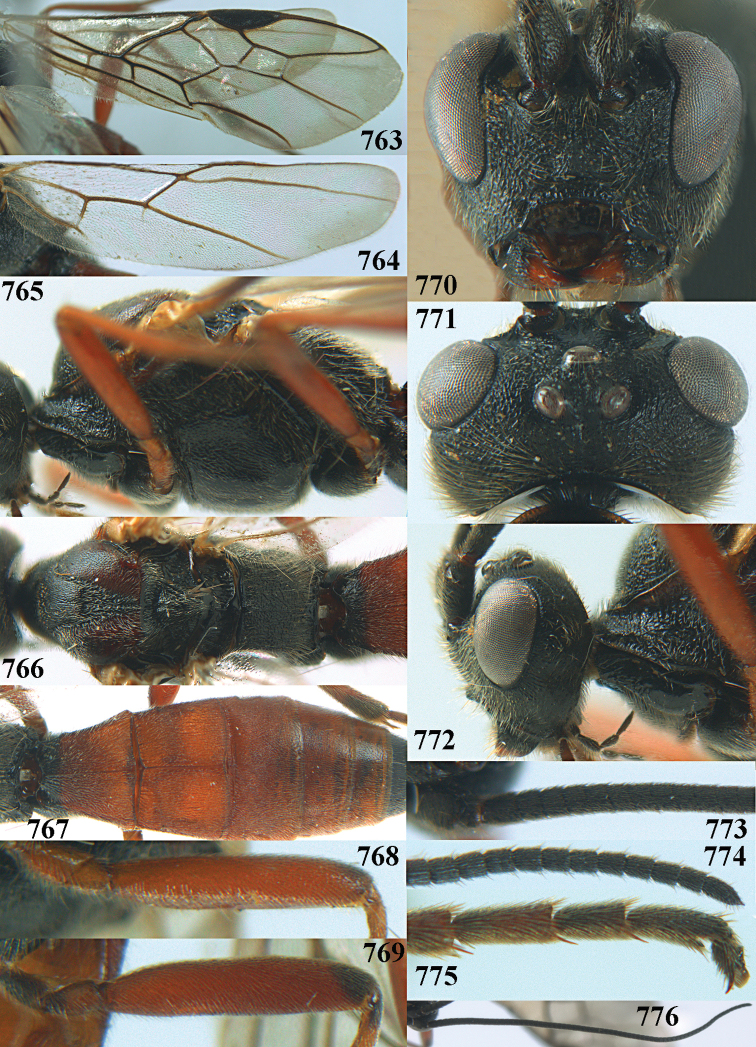
*Aleiodes sibiricus* (Kokujev), ♀, Hungary, Heves **763** fore wing **764** hind wing **765** mesosoma lateral **766** mesosoma dorsal **767** metasoma dorsal **768** fore femur lateral **769** hind femur lateral **770** head anterior **771** head dorsal **772** head lateral **773** base of antenna **774** apex of antenna **775** outer hind tarsal claw **776** antenna.

#### Aleiodes turcicus

Taxon classificationAnimaliaHymenopteraBraconidae

van Achterberg & Shaw sp. nov.

3DCD7753-2BA1-58CB-8965-223FBD52EE35

http://zoobank.org/885C0189-5A7B-4D35-B7D3-82F1094633AA

Figs 777–778 , 779–791

##### Type material.

Holotype, ♀ (NMS), “**Turkey**: Sivas, v.2001, D.L.J. Quicke”, “MRS *Aleiodes* DNA 126 [one middle leg]”. Paratype: 1 ♂ (RMNH), “Turkey, Hakkâri, [20 km S Siirt, 500 m, 23.vi.1985], C.J. Zwakhals”.

##### Molecular data.

MRS126 (Turkey).

##### Biology.

Unknown. The material examined was collected in the period May–June. It is not clear how many generations occur, or how the winter is passed.

##### Diagnosis.

Maximum width of hypoclypeal depression 0.4–0.5 × minimum width of face (Fig. 786); OOL approx. 2.3 × diameter of posterior ocellus and sparsely punctate (Fig. 787); ventral margin of clypeus obtuse and not protruding in lateral view (Fig. 788); length of antenna of ♀ 1.1–1.2 × fore wing; lateral lobes of mesoscutum largely smooth; precoxal area coarsely vermiculate-rugose medially; vein 1-CU1 of fore wing approx. 0.4 × as long as vein 2-CU1; hind tarsal claws yellowish or brownish setose (Fig. 791); head and part of mesosoma black; palpi, pterostigma and apical 0.2–0.3 of hind tibia of ♀ blackish; wing membrane distinctly infuscate.

##### Description.

Holotype, ♀, length of fore wing 5.3 mm, of body 7.9 mm.

***Head.*** Antennal segments of ♀ 47, length of antenna 1.15 × fore wing, length of 4^th^ segment 1.1 × its width, and its subapical segments 1.2 × as long as wide (Figs 789, 790); frons with regular curved rugae, shiny, and rugose behind antennal sockets; OOL 2.3 × diameter of posterior ocellus, and area mostly finely remotely punctate, interspaces much larger than diameter of punctures; vertex spaced punctate laterally, densely punctate and with transverse rugae medially, shiny; clypeus medium-sized, coarsely and densely punctate; ventral margin of clypeus thick and not protruding forwards (Fig. 788); width of hypoclypeal depression 0.5 × minimum width of face (Fig. 786); length of eye 1.3 × temple in dorsal view (Fig. 787); vertex behind stemmaticum sparsely punctate; clypeus near lower level of eyes; length of malar space 0.5 × length of eye in lateral view; eyes medium-sized, elliptical (Fig. 788).

***Mesosoma.*** Mesoscutal lobes smooth between rather remote punctures, strongly shiny, more densely punctate on middle lobe; notauli distinct but shallow, especially posterior half; mesoscutum short setose, widely and strongly rugose medio-posteriorly; scutellum slightly convex, remotely punctate and evenly rounded laterally, no carina; prepectal carina strong, reaching anterior border; precoxal area coarsely vermiculate rugose anteriorly and medially, posteriorly absent; mesopleuron above precoxal area (except speculum) remotely punctate, shiny, and antero-dorsally coarsely vermiculate-rugose; metapleuron densely rugose, but dorsally punctate, interspaces approx. equal to diameter of punctures; propodeum evenly convex and coarsely rugose, medio-longitudinal carina complete, but irregular, no tubercles.

***Wings.*** Fore wing: r 0.35 × 3-SR (Fig. 779); 1-CU1 horizontal, 0.4 × as long as 2-CU1; r-m 0.9 × 2-SR, and 0.7 × 3-SR; 2^nd^ submarginal cell medium-sized (Fig. 779); cu-a slightly oblique, approx. parallel with CU1b, straight; 1-M rather curved posteriorly. Hind wing: marginal cell gradually and evenly widened, its apical width 1.9 × width at level of hamuli (Fig. 780); 2-SC+R subquadrate; m-cu distinct, shorter than cu-a.

**Figures 777, 778.**
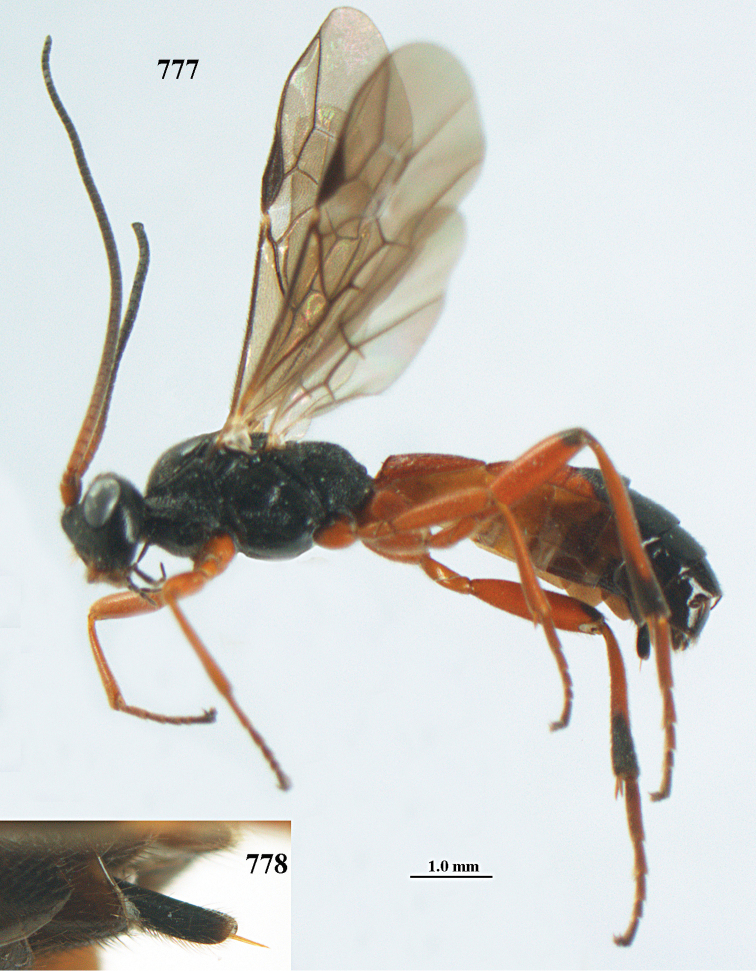
*Aleiodes turcicus* sp. nov., ♀, holotype **777** habitus lateral **778** ovipositor sheath lateral.

***Legs.*** Tarsal claws subpectinate, with four brown medium-sized pectinal bristles and some finer ones basally (Fig. 791); hind coxa moderately coarsely punctate, with several long oblique rugae, shiny; hind trochantellus robust; length of hind femur and basitarsus 4.0 and 4.9 × their width, respectively; length of inner hind spur 0.5 × hind basitarsus; hind tibia slender (Fig. 777).

***Metasoma.*** First tergite rather flattened; 1^st^ and 2^nd^ tergites coarsely and densely rugose, robust, with distinct median carina; medio-basal area of 2^nd^ tergite wide and short (Fig. 783); 2^nd^ suture deep medially and shallow laterally; basal 0.4 of 3^rd^ tergite finely striate, remainder of metasoma largely smooth, strongly shiny, punctulate; 4^th^ and apical half of 3^rd^ tergite without sharp lateral crease; ovipositor sheath rather wide, with long setae and apically rounded (Fig. 778).

***Colour.*** Black; palpi, base of middle coxa, apical 0.2 (dorsally)–0.3 (inner side) of hind tibia, apex of hind femur and telotarsi blackish; remainder of tarsi more or less darkened and base of hind tibia dark brown; basal seven segments of antenna (remainder more or less dark brown), 1^st^ and 2^nd^ tergites and antero-lateral corners of 3^rd^ tergite, and remainder of legs orange brown; humeral plate pale yellowish but partly darkened; pterostigma blackish; veins dark brown; wing membrane blackish infuscate.

***Variation.*** Apical metasomal tergites of ♂ type 2; inner hind tibial spur 0.50 × hind basitarsus; mesopleuron, metapleuron and propodeum may be largely yellowish.

##### Distribution.

Turkey.

**Figures 779–791.**
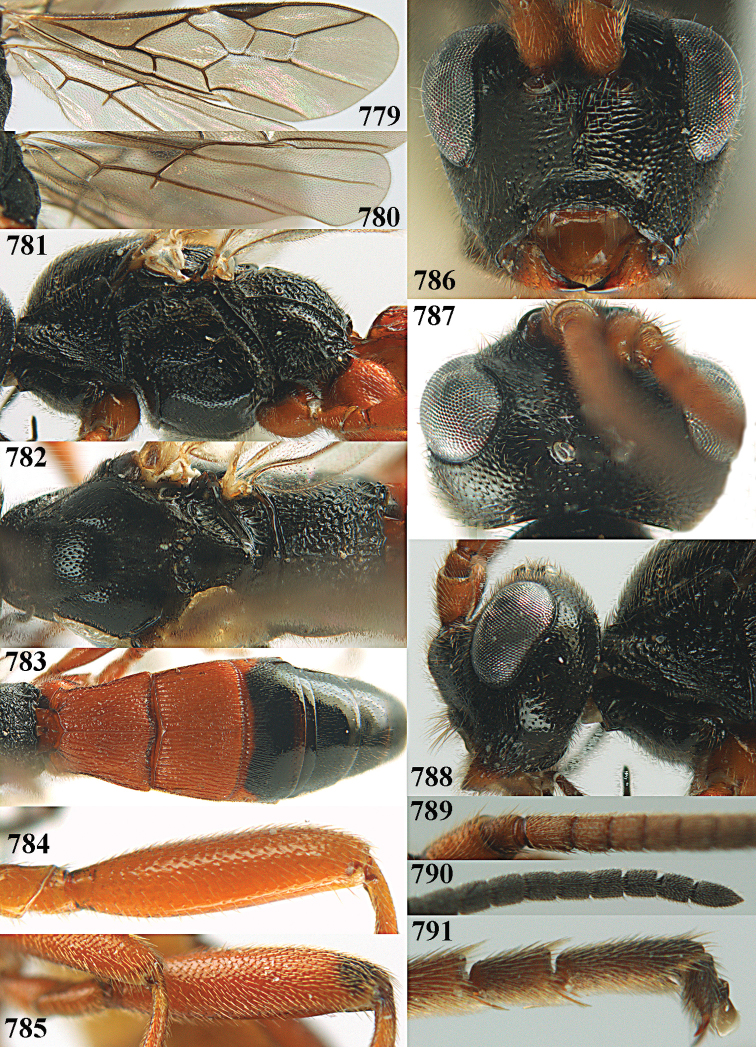
*Aleiodes turcicus* sp. nov., ♀, holotype **779** fore wing **780** hind wing **781** mesosoma lateral **782** mesosoma dorsal **783** metasoma dorsal **784** fore femur lateral **785** hind femur lateral **786** head anterior **787** head dorsal **788** head lateral **789** base of antenna **790** apex of antenna **791** outer hind tarsal claw.

#### Aleiodes unipunctator

Taxon classificationAnimaliaHymenopteraBraconidae

(Thunberg, 1822)

B0E1D2FC-6C1C-54EB-AB9C-1858C4DF4BC9

Figs 792–794 , 795–807 , 808–812

Ichneumon unipunctator Thunberg, 1822: 267 [examined].
Rogas unipunctator ; Shenefelt 1975: 1254–1255; Zaykov 1980c: 229; Jakimavicius 1974: 96.
Rogas (Rogas) unipunctator ; Tobias 1976: 84, 1986: 78 (transl.: 128).
Aleiodes (Neorhogas) unipunctator ; Papp 1985a: 151, 163, 1991a: 86, 1996: 456; Belokobylskij 1996: 18; Papp and Rezbanyai-Reser 1996: 71, 73, 95, 96; Riedel et al. 2002: 106.
Aleiodes (Chelonorhogas) unipunctator ; Belokobylskij 2000: 44, 2003: 399; Chen and He 1997: 43; He et al. 2000: 667; Rastegar et al. 2012: 3; Farahani et al. 2015: 229, 244.
Aleiodes unipunctator ; Čapek and Lukás 1989: 31; Bergamasco et al. 1995: 6; O’Connor et al. 1999: 92; Fortier and Shaw 1999: 230; Belokobylskij and Taeger 2001: 115; Marsh and Shaw 2001: 303; Belokobylskij et al. 2003: 399; Zaldívar-Riverón et al. 2004: 234; Papp 2005: 177; Lozan et al. 2010: 17.
Ichneumon ductor Thunberg, 1822: 269; Papp 1985a: 157 (not auctt.) [examined]. Syn. nov.
Aleiodes irregularis Wesmael, 1838: 101; Shenefelt 1975: 1255 (as synonym of A. unipunctator); Papp 1985a: 163 (id.) [examined].
Rhogas unipunctator ab. nigrescens Hellén, 1927: 23; Shenefelt 1975: 1255 (excluded name).

##### Type material.

Holotype of *A. unipunctator*, ♂ (ZMUU) with holotype label by CvA. Holotype of *A. ductor*, ♂ (ZMUU), “α”, “*Rhogas ductor* Thbg”, “Uppsala Univ. Zool Mus., Thunbergsaml. Nr. 25332, *Ichneumon ductor* Sv. Type”. Holotype of *A. irregularis*, ♂ (KBIN), “[**Belgium**], 11 Juin, Brig.”, “*A. irregularis* ♂ mihi 5”, “dét. C. Wesmael”, “Belgique, Bruxelles/teste Papp J., 1983”, “Holotype”, “*Aleiodes irregularis* Wesm., 1838, ♂, Papp, 1983”, “*Aleiodes unipunctator* Thb. ♂, det. Papp J., 1984”.

##### Additional material.

Austria, Belgium, British Isles (England: V.C.s 1, 3, 5, 6, 8, 15, 16, 17, 18, 20, 21, 23, 25, 26, 27, 28, 29, 32, 33, 40, 53, 55, 58, 59, 63, 64, 65, 66, 69; Wales: V.C.s 35, 52 ; Scotland: V.C.s 72, 75, 76, 77, 79, 80, 82, 83, 85, 86, 87, 88, 89, 90, 91, 95, 96, 97, 98, 99, 100, 101, 103, 105, 108, 109, 110, 111, 112; Ireland: V.C.s H5, H19, H20, H21, H22, H28, H30), Bulgaria, Czech Republic, Denmark, Finland, Germany, Greece (mainland, Corfu), Hungary, Italy, Montenegro, Netherlands (DR: Wijster; Borger; GE: Nunspeet; Kemperberg, NB: Bergen op Zoom, ZH: Arkel; Melissant; Oostvoorne; ZE: Oostkapelle), Norway, Romania, Russia, Sweden, [Kazakhstan, Tadzhikistan, W. Caucasus]. Specimens in AAC, ALC, ZJUH, BZL, CNC, FMNH, HHC, HSC, IKC, OUM, MMUM, MRC, MSC, MSNV, MTMA, NMI, NMS, RMNH, SDEI, UMZC, UNS, UWIM, ZMUU, ZSSM.

##### Molecular data.

MRS211 (UK), MRS221 (Germany), MRS354 (UK), MRS893 (UK).

##### Biology.

Univoltine, flying from May to August. Reared from the noctuids *Apamea unanimis* (Hübner) (23 [1 ZJUH, 1 FMNH, 1 NRS, 1 MTMA]; M.R. Shaw), *Apamea crenata* (Hufnagel) (2:1, ZJUH; G.T. Lyle), Apamea ? sordens (Hufnagel) (3:1; K.P. Bland), and from mummies compatible with *Apamea* spp. (8). These closely related hosts all live in the shoots or leaf sheaths of Poaceae. The large number reared from *A. unanimis* may be at least partly the result of a sampling bias, as the larvae of that species are so readily detected and collected when feeding on *Phalaris*. The winter is passed in the relatively slender brown mummy, which is nearly cylindrical and only weakly keeled laterally (Fig. 794). It probably usually forms at or below soil level and is rather weakly contracted at the head end (which is bent sideways in a high proportion of cases), with the thinly silken cocoon occupying abdominal segments (2–)3–8. This species is widespread and often abundant, especially in rank or damp grassland habitats, in the northern part of its range.

##### Diagnosis.

Maximum width of hypoclypeal depression 0.5–0.6 × minimum width of face (Fig. 802); OOL of ♀ approx. 0.8 × as long as diameter of posterior ocellus and mainly granulate mixed with some punctures (Fig. 803); ventral margin of clypeus thick, not protruding in lateral view (Fig. 804); mesoscutal lobes finely granulate-punctulate and matt; precoxal area comparatively narrow and moderately rugose medially; marginal cell of fore wing of ♀ ending rather close to wing apex (Fig. 795); vein 1-CU1 of fore wing 0.4–0.5 × as long as vein 2-CU1 (Fig. 795); hind tarsal claws rather robust and only brownish setose (Fig. 807); 1^st^ tergite rather slender basally (Fig. 798); whole 4^th^ and part of 3^rd^ metasomal tergite smooth and very glossy (Fig. 798); labial palp yellowish brown or brown; basal half of hind tibia pale yellowish or ivory, at least inner side contrasting with reddish or dark brown colour of basal half of hind femur (usually less pronounced in ♂), and its apex dark brown or black; 4^th^ and 5^th^ tergites black; wings rather slender and their membrane subhyaline.

##### Description.

Redescribed ♀ (RMNH) from Sweden (Storbacken). Length of fore wing 5.8 mm, of body 5.7 mm.

***Head.*** Antennal segments of ♀ 50, length of antenna 1.2 × fore wing, its subapical segments rather robust; frons largely smooth anteriorly (except some fine sculpture) and densely rugulose posteriorly; OOL 0.8 × diameter of posterior ocellus, granulate with some punctures and matt; vertex granulate with some rugulosity and rather dull; clypeus coarsely punctate; ventral margin of clypeus thick and not protruding forwards (Fig. 804); width of hypoclypeal depression 0.6 × minimum width of face (Fig. 802); length of eye 1.7 × temple in dorsal view (Fig. 803); vertex behind stemmaticum rugulose-granulate; clypeus near lower level of eyes; length of malar space 0.25 × length of eye in lateral view.

***Mesosoma.*** Mesoscutal lobes moderately punctate and interspaces distinctly granulate, with satin sheen; precoxal area of mesopleuron moderately rugose medially, sparsely punctulate posteriorly as surroundings of precoxal area; scutellum rather sparsely punctate, but medio-posteriorly rugulose, shiny; propodeum rather convex and moderately rugose, medio-longitudinal carina complete, and no protruding carinae laterally.

***Wings.*** Fore wing: r 0.3 × 3-SR (Fig. 795); 1-CU1 narrow and horizontal, 0.4 × 2-CU1; r-m 0.5 × 3-SR; 2^nd^ submarginal cell medium-sized and 1^st^ subdiscal cell slender (Fig. 795); cu-a nearly vertical, straight; 1-M slightly curved posteriorly; 1-SR narrow posteriorly and widened anteriorly; surroundings of M+CU1, 1-M and 1-CU1 largely setose. Hind wing: basal half of marginal cell slightly wider and its apical half distinctly gradually widened, its apical width 2.2 × width at level of hamuli (Fig. 795); 2-SC+R short longitudinal; m-cu indistinct; M+CU:1-M = 4:3; 1r-m 0.7 × 1-M.

***Legs.*** Tarsal claws yellowish bristly setose, without distinct pecten (Fig. 807); hind coxa largely distinctly punctate; hind trochantellus robust; length of hind femur and basitarsus 4.0 and 6.3 × their width, respectively; length of inner hind spur 0.5 × hind basitarsus.

***Metasoma.*** First tergite rather flattened, 1.2 × as long as wide apically; 1^st^ and 2^nd^ tergites with distinct medio-longitudinal carina and longitudinally striate; medio-basal area of 2^nd^ tergite wide triangular and short (Fig. 798); 2^nd^ suture rather deep and finely crenulate; 3^rd^ tergite nearly entirely smooth and strongly shiny, as remainder of metasoma; 4^th^ and apical half of 3^rd^ tergite without sharp lateral crease; ovipositor sheath wide, with rather long setae and apically truncate (Fig. 793).

***Colour.*** Black; pronotum dorso-posteriorly, telotarsi largely, hind tarsus, apical half of hind tibia, pterostigma (except paler extreme base), most veins and apical fifth of 2^nd^ tergite dark brown; palpi brown; tegulae and basal half of hind tibia pale yellowish; apical third of 1^st^ tergite and 2^nd^ tergite (except apically) and remainder of legs, orange brown; wing membrane subhyaline.

***Variation.*** Propodeum and pronotum sometimes weakly marked with orange. One male seen with vein r-m of fore wing absent. Antennal segments: ♀ 47(1), 48(7), 49(12), 50(21), 51(26), 52(40), 53(40), 54(15), 55(5), 56(3), 57(1); ♂ 47(1), 48(1), 49(1), 50(8), 51(7), 52(22), 53(27), 54(34), 55(35), 56(21), 57(6), 58(8), 59(1). On average males have ca one to two more antennal segments than females. Male is very similar with apical tergites type 2, setae rather sparse and fringe short (Figs 809, 810). Melanistic females and males occur, metasoma entirely blackish or with only apex of 1^st^ tergite narrowly and 2^nd^ tergite medially and antero-laterally dark orange; clypeus reddish brown or blackish; apex of hind femur often somewhat infuscate.

##### Distribution.

Austria, British Isles (England, Wales, Scotland, Ireland), Bulgaria, Czech Republic, *Denmark, Finland, Germany, *Greece (mainland and Corfu), Hungary, Italy, *Kazakhstan, *Montenegro, Netherlands, Norway, *Romania, Russia (including W. Caucasus), Sweden, *Tadzhikistan.

##### New synonymy.

Both male holotypes of *I. unipunctator* and *I. ductor* are preserved in the Thunberg collection, but are severely damaged. From the holotype of *A. ductor* only the head, fore coxa, mesoscutum and metasoma remain (Roman, 1912). The holotype of *A. unipunctator* has the head and the metasoma separately glued on a card and the mesosoma is still attached to the pin. Judging from these remnants (especially the mainly smooth and very shiny 3^rd^ tergite, the wide hypoclypeal depression (0.6 × minimum width of face) and the coriaceous vertex), it is obvious that both belong to the same species. Consequently, *A. ductor* (Thunberg) is synonymised with *A. unipunctator* (Thunberg) (syn. nov.). *Aleiodes ductor* auctt. is divided among *A. pallidicornis* (Herrich-Schäffer, 1838) (N and C European populations) and *A. apicalis* (Brullé, 1832) (Mediterranean and southern C European populations).

**Figures 792–794.**
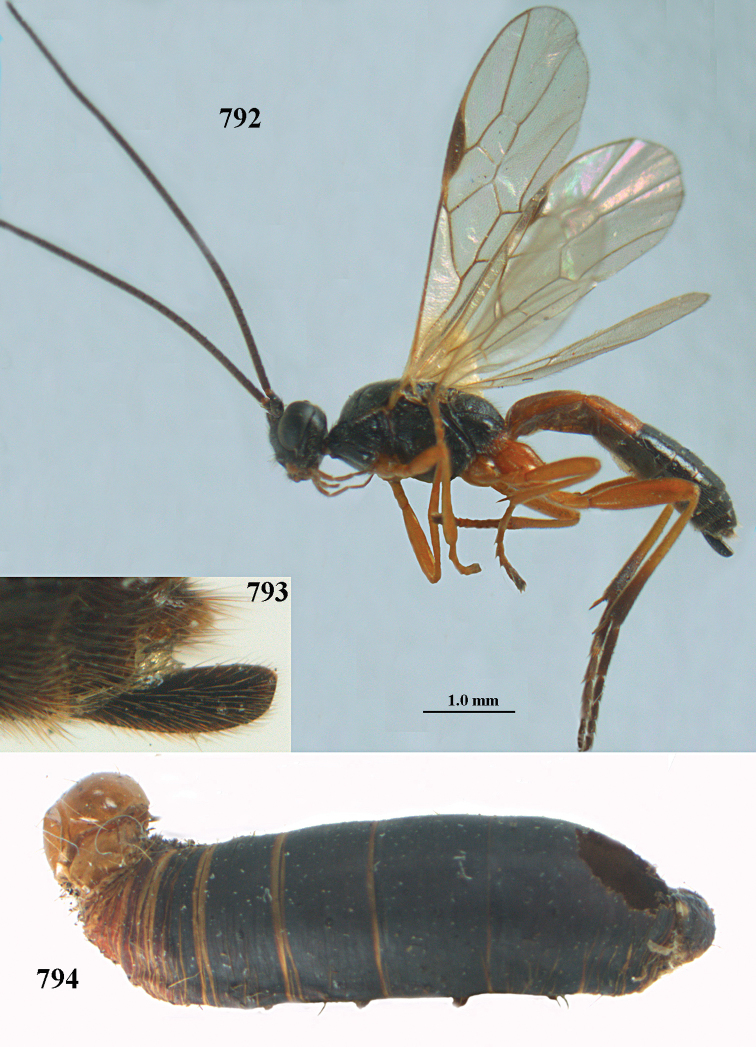
*Aleiodes unipunctator* (Thunberg), ♀, England, Fletcher Moss **792** habitus lateral **793** ovipositor sheath lateral **794** mummy of *Apamea unanimis* Hübner.

**Figures 795–807.**
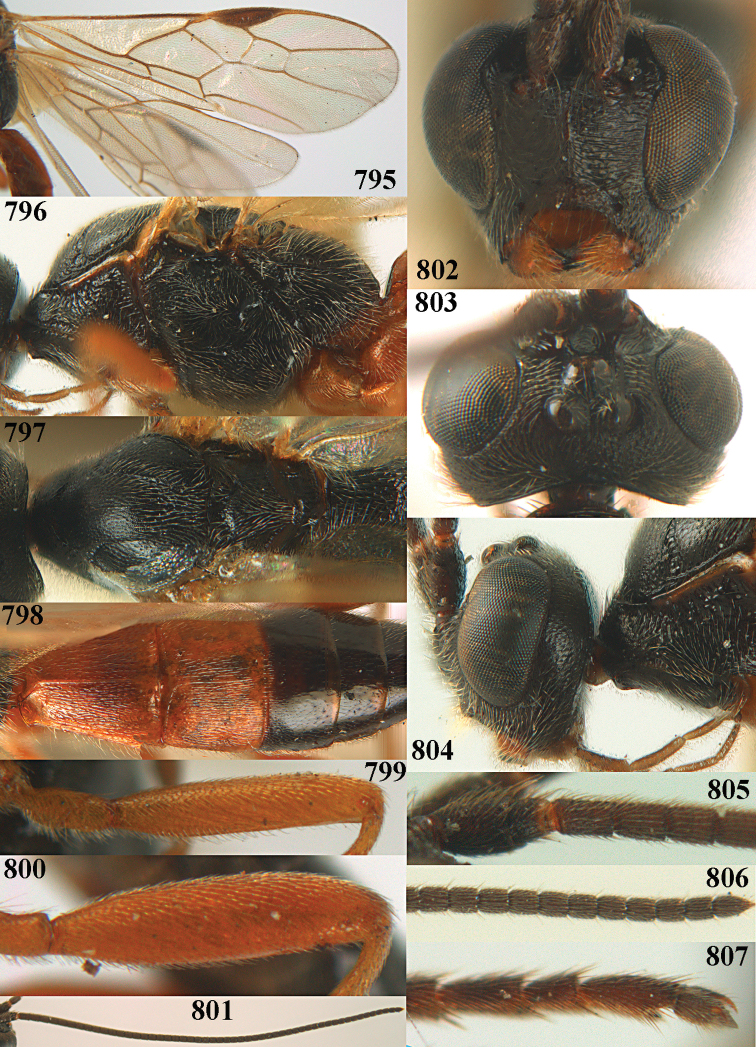
*Aleiodes unipunctator* (Thunberg), ♀, England, Fletcher Moss **795** wings **796** mesosoma lateral **797** mesosoma dorsal **798** metasoma dorsal **799** fore femur lateral **800** hind femur lateral **801** antenna **802** head anterior **803** head dorsal **804** head lateral **805** base of antenna **806** apex of antenna **807** inner hind tarsal claw lateral.

**Figures 808–812.**
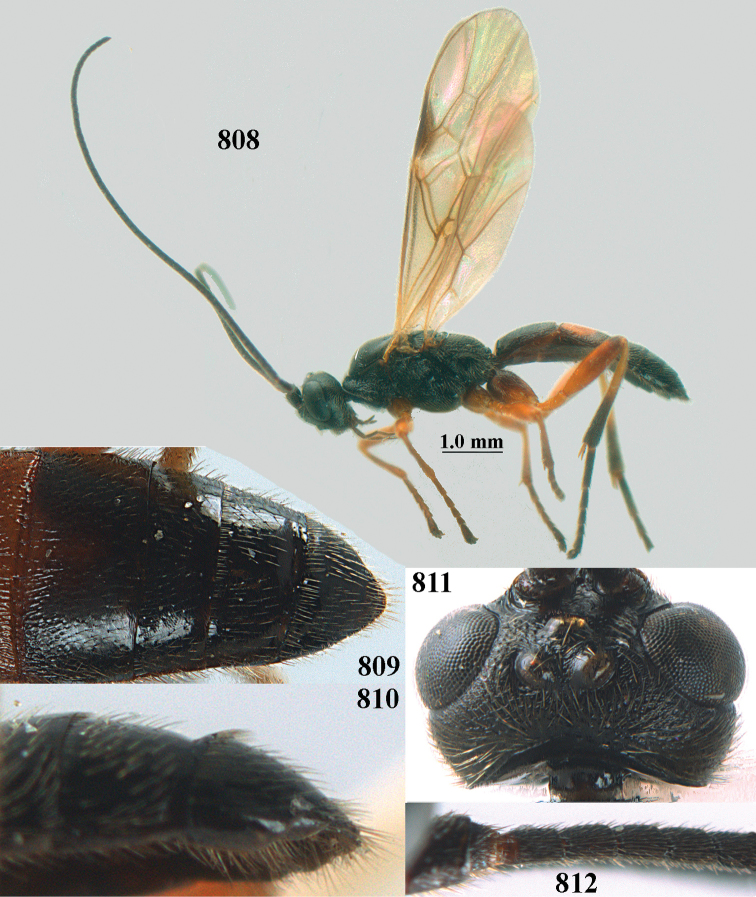
*Aleiodes unipunctator* (Thunberg), ♂, Scotland, Edinburgh, but 808 Sweden, Särö-Hamra **808** habitus lateral **809** apex of metasoma dorsal **810** apex of metasoma lateral **811** head dorsal **812** base of antenna.

#### Aleiodes venustulus

Taxon classificationAnimaliaHymenopteraBraconidae

(Kokujev, 1905)

4DEAB885-8DBC-588C-B538-E5754A94E3FC

Figs 813–814 , 815–826

Rhogas venustulus Kokujev, 1905: 15.
Rogas venustulus ; Shenefelt 1975: 1255–1256; Tobias 1986: 78 (transl.: 129).
Aleiodes venustulus ; Fortier and Shaw 1999: 230; Aydogdu and Beyarslan 2005: 192, 2006: 87.
Rhogas (Rhogas) robustus Telenga, 1941: 151, 415.
Rogas robustus ; Tobias 1986: 78 (transl.: 129; as synonym of A. venustulus).

##### Type material.

Holotype of *A. venustulus*, ♀ (ZISP), “[**Kyrgyzstan**], Ushch. Kyzyl-su[u], [= village south of Lake Issyk Kul], 7–9.000’[ft], 5.vii.[19]03, E. Pojarkov”, No. 2273, *Rh. venustulus* Kok.”, “K. Kokujeva”.

##### Additional material.

1 ♀ + 1 ♂ (MTMA), “[**Kazakhstan**:] Turkestan, Almásy, Naryn-kol, 1906, Tekkes”.

##### Molecular data.

None.

##### Biology.

Unknown.

##### Diagnosis.

Maximum width of hypoclypeal depression 0.6–0.7 × minimum width of face (Fig. 822); OOL twice as long as diameter of posterior ocellus and coarsely rugose; clypeus rather short, rather flat, weakly protruding anteriorly and its ventral margin thick (Fig. 824); mesoscutum coarsely and remotely punctate, with interspaces smooth and wider than punctures; precoxal sulcus area coarsely and densely punctate; vein 1-CU1 of fore wing approx. 0.8 × as long as vein 2-CU1 and as long as vein m-cu (Fig. 815); membrane near veins M+CU1 and 1-CU1 of fore wing sparsely setose; tarsal claws rather robust and with some fine dark brown spiny bristles subbasally (Fig. 825); head brownish yellow; vein 1-M of fore wing brown; apical half of hind tibia dark brown; metasoma of ♀ yellowish, but anterior 0.6 of first tergite black; wing membrane subhyaline.

##### Description.

Holotype of *A. venustulus*, ♀, length of fore wing 6.7 mm, of body 8.6 mm.

***Head.*** Antenna incomplete, with eight segments remaining; frons rugose and shiny; OOL twice diameter of posterior ocellus, mainly rugose and shiny; stemmaticum densely punctate; vertex remotely punctate and shiny; clypeus punctate and slightly convex; ventral margin of clypeus thick and anterior part weakly protruding (Fig. 824); width of hypoclypeal depression 0.6 × minimum width of face (Fig. 822); length of eye 1.3 × temple in dorsal view (Fig. 823); vertex behind stemmaticum convex and remotely punctate; clypeus near lower level of eyes; length of malar space 0.4 × length of eye in lateral view and temple as wide as eye.

***Mesosoma.*** Mesoscutum coarsely and remotely punctate, with interspaces smooth and wider than punctures; precoxal area coarsely and densely punctate, remainder of mesopleuron remotely punctate and antero-dorsally rugose; metapleuron densely and coarsely punctate; metanotum with coarse medio-longitudinal carina anteriorly; scutellum punctate; propodeum convex and coarsely rugose, its medio-longitudinal carina present only on anterior third of propodeum.

***Wings.*** Fore wing: r 0.4 × 3-SR (Fig. 815); 1-CU1 slightly oblique, 0.2 × 2-CU1; r-m 0.6 × 3-SR; 2^nd^ submarginal cell medium-sized (Fig. 815); cu-a inclivous, straight; 1-M nearly straight posteriorly; 1-SR wide; surroundings of M+CU1, 1-M and 1-CU1 largely glabrous. Hind wing: marginal cell linearly widened, its apical width twice width at level of hamuli (Fig. 816); 2-SC+R short and vertical; m-cu absent; M+CU:1-M = 12:11; 1r-m 0.7 × 1-M.

***Legs.*** Tarsal claws rather robust and with some fine dark brown spiny bristles subbasally (Fig. 825); hind coxa largely densely punctate; hind trochantellus rather robust; length of hind femur and basitarsus 4.7 and 6.5 × their width, respectively; length of inner hind spur 0.4 × hind basitarsus.

***Metasoma.*** First tergite evenly convex, as long as wide apically; 1^st^ and 2^nd^ tergites with medio-longitudinal carina and coarsely rugose-reticulate; medio-basal area of 2^nd^ tergite triangular and rather distinct (Fig. 819); 2^nd^ suture deep and narrow; basal half of 3^rd^ tergite punctate-rugose, remainder of metasoma superficially micro-sculptured; 4^th^ and apical half of 3^rd^ tergite without sharp lateral crease; ovipositor sheath wide, with long setae and apically truncate (Fig. 814).

***Colour.*** Black; antenna (except scapus and pedicellus), palpi, tegulae, fore and middle telotarsi, veins, and pterostigma dark brown; coxae, trochanters and trochantelli, apical third of hind femur (ventrally extended to its apical two-thirds), hind tibia (except pale yellowish basal ring), fore and middle femora apically, and hind tarsus black; remainder of legs yellowish brown; wing membrane subhyaline.

***Variation.*** No specimens with intact antennae examined; 2^nd^ tergite coarsely rugose-reticulate or coarsely longitudinally rugose. Male is very similar and with apical tergites type 3, setae quite dense, glabrous stripe narrow, and fringe very weak.

##### Distribution.

Kazakhstan, Kyrgyzstan.

##### Notes.

This Central Asian species bears a superficial resemblance to *A. miniatus* and *A. aestuosus*. It is included in this revision, because it has been reported twice from Turkey (Aydogdu and Beyarslan 2005, 2006).

**Figures 813, 814.**
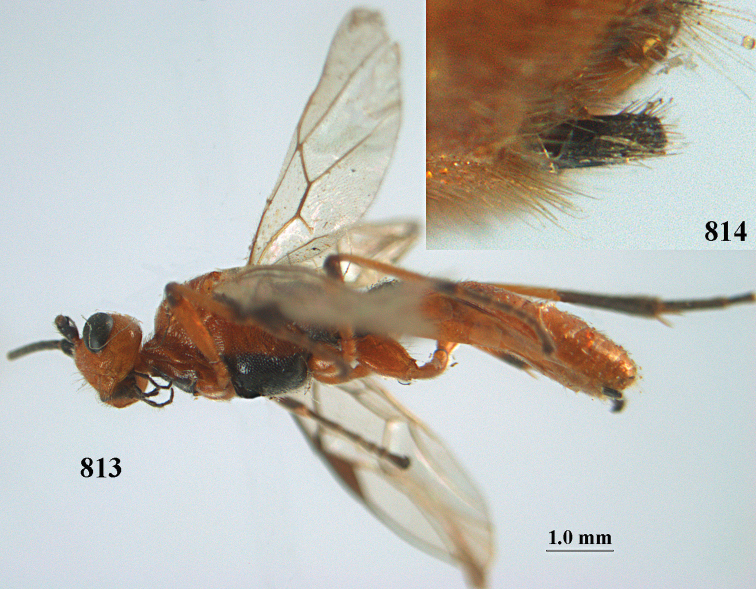
*Aleiodes venustulus* (Kokujev), ♀, holotype **813** habitus lateral **814** ovipositor sheath lateral.

**Figures 815–826.**
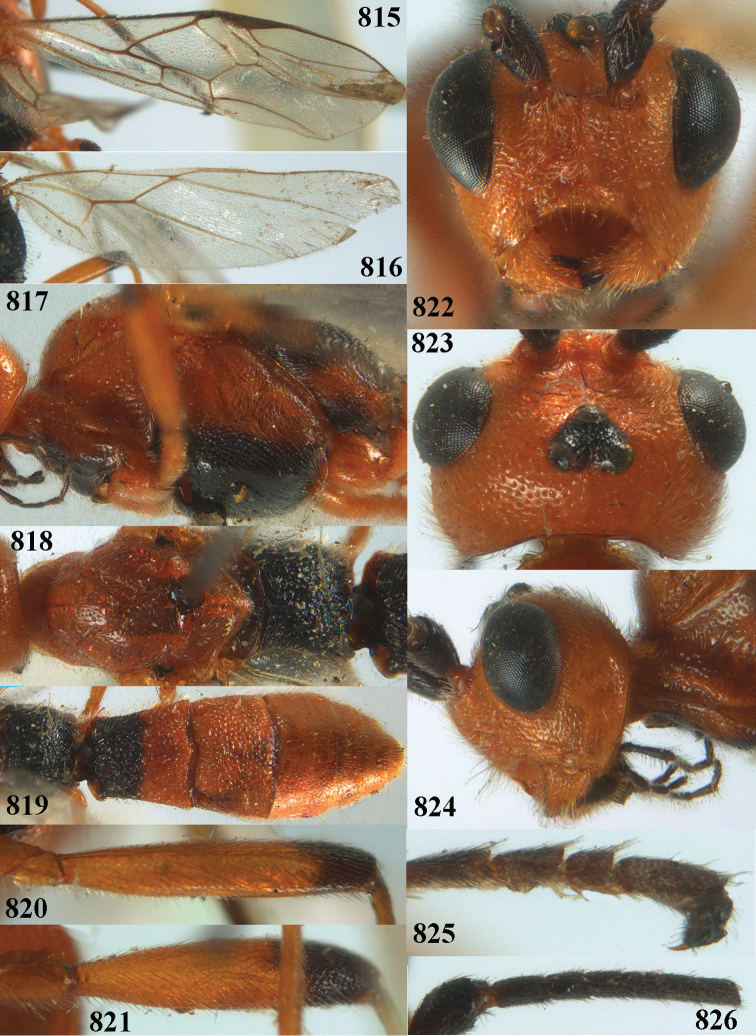
*Aleiodes venustulus* (Kokujev), ♀, holotype **815** fore wing **816** hind wing **817** mesosoma lateral **818** mesosoma dorsal **819** metasoma dorsal **820** fore femur lateral **821** hind femur lateral **822** head anterior **823** head dorsal **824** head lateral **825** inner hind tarsal claw lateral **826** base of antenna.

#### Aleiodes zwakhalsi

Taxon classificationAnimaliaHymenopteraBraconidae

van Achterberg & Shaw sp. nov.

9BE89C08-4F59-52A4-9134-46994C08F756

http://zoobank.org/3C42EDAB-B9DB-45DB-884C-B23A630B249F

Figs 827–829 , 830–840

##### Type material.

Holotype, ♀ (RMNH), “**Turkey**, Ankara, Kizilcahaman, 1100 m, 17.vi.1985, C.[J.] Zwakhals”. Paratypes: 1 ♂ (RMNH), “Turkey, Agri, Mt. Ararat, 1800 m, 3.vii.1985, C.J. Zwakhals”; 1 ♀ (RMNH), “Turkiye, Gümüshane, 40–46 km E [of] Bagburt, 1500 m, 19.vii.1989, J.A.W. Lucas”; 1 ♀ (NMS), “Turkey: Zigana Dagi, 5,000 ft., SW of Tabzon, 10.viii.1959, K.M. Guichard”.

##### Molecular data.

None.

##### Biology.

Unknown. Probably univoltine as all known adults were collected in the period June to August, but there is no indication of its means of overwintering.

##### Diagnosis.

Maximum width of hypoclypeal depression approx. 0.7 × minimum width of face (Fig. 837); OOL of ♀ finely remotely punctate and 0.8–1.0 × diameter of posterior ocellus; ventral margin of clypeus thick apically and not protruding in lateral view (Fig. 839); lobes of mesoscutum densely finely punctate, with interspaces shiny; precoxal area densely punctate and with some rugae medially; vein cu-a of fore wing oblique, parallel with vein 3-CU1; surroundings of veins M+CU1 and 1-+2-CU1 largely setose; vein r of fore wing 0.4–0.5 × vein 3-SR (Fig. 830); vein 1-CU1 of fore wing 0.3–0.6 × vein 2-CU1 (Fig. 830); hind tarsal claws with rather conspicuous pale brown pecten (Fig. 840); 1^st^ tergite gradually narrowed basally (Fig. 833); 2^nd^ tergite 0.7 × as long as wide basally and black; 3^rd^ tergite densely punctulate basally, and sparsely so apically; head black; vein 1-M of fore wing brown; wing membrane subhyaline.

This new species is similar to *A. cruentus* (Nees), but it differs by having the surroundings of veins M+CU1 and 1-+2-CU1 partly setose (Fig. 828; largely glabrous in *A. cruentus*), vein M+CU1 of fore wing with bend near its distal fifth (Fig. 828; straight or slightly curved), vein cu-a of fore wing oblique, parallel with vein 3-CU1 (vertical or nearly so, rarely oblique), vein r of fore wing 0.4–0.5 × vein 3-SR (0.3–0.4 ×), vein 1-CU1 of fore wing 0.3–0.6 × vein 2-CU1 (0.8–1.1 ×, rarely less), OOL of ♀ 0.8–1.0 × diameter of posterior ocellus (0.5–0.8 ×, rarely longer), length of eye 1.1–1.6 × temple in dorsal view (1.5–1.9 ×), ovipositor sheath comparatively slender and area between ocelli and eyes moderately punctate (coarsely punctate). Also very similar to *A. diversus* (Szépligeti), it differs from the latter by having vein 1-CU1 of fore wing distinctly shorter than vein m-cu, hind femur approx. 4 × as long as wide, vein cu-a inclivous (parallel with vein 3-CU1; vertical and vein 3-CU1 diverging posteriorly in *A. diversus*), 5^th^–10^th^ antennal segments of ♀ as long as wide (shorter than wide), vertex and OOL remotely punctate (densely punctate) and ovipositor sheath slender (robust).

##### Description.

Holotype, ♀, length of fore wing 7.0 mm, of body 9.0 mm.

***Head.*** Antennal segments of ♀ 59, length of antenna 1.1 × fore wing, its subapical segments moderately slender; frons largely smooth; OOL equal to diameter of posterior ocellus, finely remotely punctate and shiny; vertex distinctly punctate and shiny; clypeus punctate-rugose, wide and short; ventral margin of clypeus thick and not protruding forwards (Fig. 839); width of hypoclypeal depression 0.7 × minimum width of face (Fig. 837); length of eye 1.1 × temple in dorsal view (Fig. 838); vertex behind stemmaticum densely punctate; clypeus near lower level of eyes; length of malar space 0.25 × length of eye in lateral view.

***Mesosoma.*** Mesoscutal lobes largely densely and finely punctate, shiny; precoxal area of mesopleuron densely punctate, medially with few rugae; surroundings of precoxal area densely punctate; scutellum sparsely and finely punctate, rather flat, shiny and laterally rugose-punctate; propodeum evenly convex and coarsely rugose, medio-longitudinal carina complete but irregular posteriorly, and no protruding carinae laterally.

***Wings.*** Fore wing: r 0.4 × 3-SR (Fig. 830); 1-CU1 horizontal, 0.5 × 2-CU1 and 0.7 × m-cu; r-m 0.6 × 3-SR; 2^nd^ submarginal cell medium-sized (Fig. 830); cu-a inclivous, straight; 1-M slightly curved posteriorly; vein M+CU1 of fore wing with distinct bend near its distal fifth (Fig. 828); 1-SR widened; surroundings of M+CU1, 1-M and 1-CU1 largely setose. Hind wing: marginal cell gradually widened, its apical width 2.1 × width at level of hamuli (Fig. 830); 2-SC+R subquadrate; short m-cu weakly developed; M+CU:1-M = 7:4; 1r-m 0.9 × 1-M.

***Legs.*** Tarsal claws with rather conspicuous pale brownish pecten (Fig. 840); hind coxa largely densely punctulate; hind trochantellus robust; length of hind femur and basitarsus 4.0 and 5.0 × their width, respectively; length of inner hind spur 0.55 × hind basitarsus.

***Metasoma.*** First tergite rather flattened, as long as wide apically and distinctly narrowed basally (Fig. 833); 1^st^ and 2^nd^ tergites with medio-longitudinal carina and coarsely longitudinally rugose; medio-basal area of 2^nd^ tergite wide triangular and short (Fig. 833); 2^nd^ tergite 0.7 × as long as its basal width; 2^nd^ suture deep and finely reticulate; basally 3^rd^ tergite densely punctulate and apically (as remainder of metasoma) sparsely punctulate; 4^th^ and apical half of 3^rd^ tergite without sharp lateral crease; ovipositor sheath slender, with medium-sized setae and apically rounded (Fig. 829).

***Colour.*** Black (including fore coxa anteriorly and basally); apex of hind tibia, telotarsi, hind tarsus, palpi, pterostigma and veins, dark brown; hind tibia (except apex) brownish yellow; clypeus narrowly ventrally, remainder of legs, pronotum (except ventrally), mesopleuron dorsally, mesoscutum, scutellum, and metanotum, orange-brown; tegulae pale brownish yellow; wing membrane subhyaline.

***Variation.*** Antennal segments of ♀ 58(1), 59(1), 60(1), of ♂ 62(1); vein r of fore wing 0.4–0.5 × vein 3-SR; vein 1-CU1 of fore wing 0.3–0.6 × vein 2-CU1; OOL of ♀ 0.8–1.0 × diameter of posterior ocellus; length of eye 1.1–1.6 × temple in dorsal view; clypeus ventrally orange brown or black; mesopleuron dorsally or largely orange brown; basal half of third tergite rugose, punctate-rugose or punctulate; entire 1^st^ tergite orange brown, posterior half of 1^st^ tergite brownish and rest of tergite blackish (as base of middle coxa) or entirely black. Male is very similar, apical tergites type 1 and no fringe observed.

##### Distribution.

Turkey (Asian part: 1100–1800 m).

**Figures 827–829.**
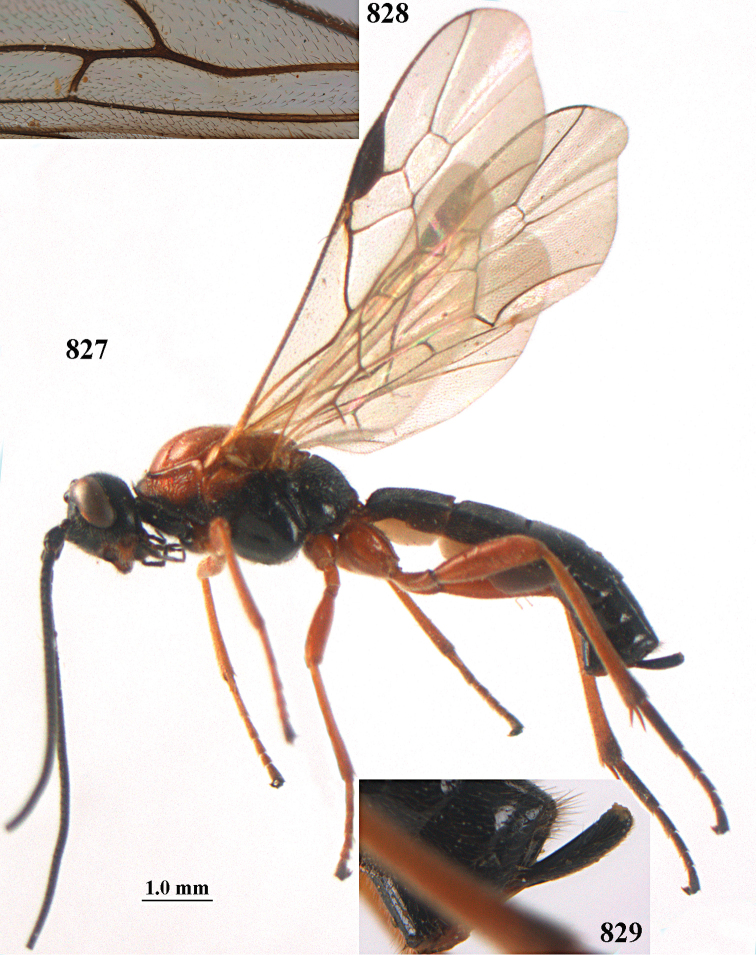
*Aleiodes zwakhalsi* sp. nov., ♀, holotype **827** habitus lateral **828** detail of fore wing **829** ovipositor sheath lateral.

**Figures 830–840.**
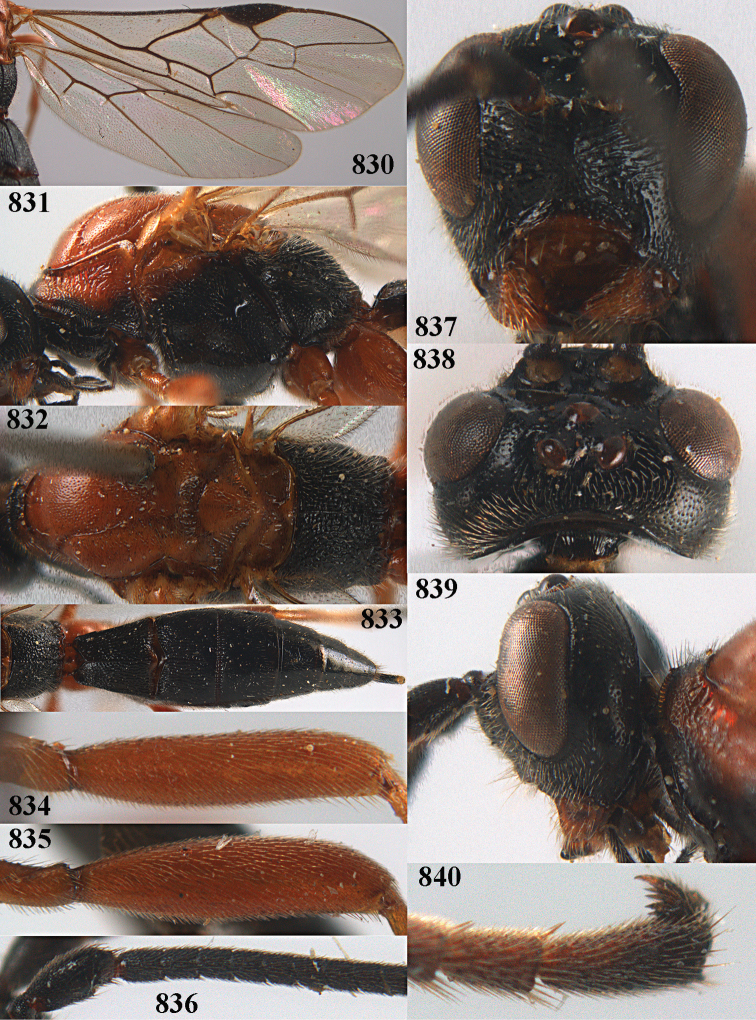
*Aleiodes zwakhalsi* sp. nov., ♀, holotype **830** wings **831** mesosoma lateral **832** mesosoma dorsal **833** metasoma dorsal **834** fore femur lateral **835** hind femur lateral **836** base of antenna **837** head anterior **838** head dorsal **839** head lateral **840** outer hind tarsal claw lateral.

## Erratum for Part 1

In the key given by van Achterberg and Shaw (2016) the newly described species *A. carminatus* van Achterberg & Shaw was inserted at a late stage, which led to an error in couplet 14, as there is also a weak apical comb at the apex of the inner side of the hind tibia present in *A. angustipterus* van Achterberg & Shaw. The other characters provided should easily separate the two species.

## Supplementary Material

XML Treatment for Aleiodes

XML Treatment for Aleiodes apicalis

XML Treatment for Aleiodes aestuosus

XML Treatment for Aleiodes agilis

XML Treatment for Aleiodes apicalis

XML Treatment for Aleiodes arnoldii

XML Treatment for Aleiodes aterrimus

XML Treatment for Aleiodes carbonarius

XML Treatment for Aleiodes carbonaroides

XML Treatment for Aleiodes caucasicus

XML Treatment for Aleiodes coriaceus

XML Treatment for Aleiodes cruentus

XML Treatment for Aleiodes desertus

XML Treatment for Aleiodes dissector

XML Treatment for Aleiodes diversus

XML Treatment for Aleiodes eurinus

XML Treatment for Aleiodes fahringeri

XML Treatment for Aleiodes fortipes

XML Treatment for Aleiodes gasterator

XML Treatment for Aleiodes grassator

XML Treatment for Aleiodes hemipterus

XML Treatment for Aleiodes hirtus

XML Treatment for Aleiodes improvisus

XML Treatment for Aleiodes krulikowskii

XML Treatment for Aleiodes miniatus

XML Treatment for Aleiodes morio

XML Treatment for Aleiodes nigrifemur

XML Treatment for Aleiodes nobilis

XML Treatment for Aleiodes pallidicornis

XML Treatment for Aleiodes pallidistigmus

XML Treatment for Aleiodes periscelis

XML Treatment for Aleiodes pulchripes

XML Treatment for Aleiodes quadrum

XML Treatment for Aleiodes ruficeps

XML Treatment for Aleiodes ruficornis

XML Treatment for Aleiodes rufipes

XML Treatment for Aleiodes rugulosus

XML Treatment for Aleiodes schewyrewi

XML Treatment for Aleiodes schirjajewi

XML Treatment for Aleiodes sibiricus

XML Treatment for Aleiodes turcicus

XML Treatment for Aleiodes unipunctator

XML Treatment for Aleiodes venustulus

XML Treatment for Aleiodes zwakhalsi

## Appendix 1

**Table:** List of barcoded specimens.

Genus	Species or species code	Voucher code/ Sample ID	Country	BOLD Process ID (if applicable)	GenBank accession number (s)
* Aleiodes *	* abraxanae *	MRS636	UK	ASQSP978-10	HQ551278/HQ551264
	* adorabelleae *	BCLDQ0252	Thailand	ASQSP222-08	JF963436
	* aestuosus *	MRS004	Turkey	GBAH4104-09	EU979573
	aff. wyomingensis	BIOUG01036-F12	USA	JSHYN023-11	KR791670
	* albitibia *	MRS383	Sweden	ASQSP075-08	JF962835
	* alternator *	MRS161	UK	ASQBR258-09	MK585882
	* angustipterus *	MRS276	UK	–	KU682232
	* antescutum *	BCLDQ00210	Thailand	ASQSP462-08	JF962536
	* apicalis *	MRS008	Turkey	ASQBR099-09	EU979575
		MRS111	Turkey	ASQBR099-09	JF962839
		MRS112	Turkey	ASQBR100-09	MK585857
		MRS181	Russia	ASQBR101-09	MK585870
		MRS869	Sweden	BCNCA217-18	MK585872
	* apiculatus *	MRS079	UK	–	KU682222
	* assimilis *	MRS843	France	BCNCA194-18	MK585863
	* aterrimus *	MRS024	UK	ASQBR103-09	JN000875
		MRS147	UK	GBAH4101-09	EU979577
	* bicolor *	MRS197	UK	ASQBR086-09	MK585862
	* bobwhartoni *	BCLDQ0730	Thailand	ASQSQ011-09	JF271188
	* bucculentus *	BCLDQ00454	USA	ASQSP644-08	JF962486
	* buoculus *	BCLDQ00927	USA	ASQSQ223-09	MH272394
	* buzuritriplus *	BCLDQ01479	Thailand	ASQSR027-11	JN278254
	*cameronii* Janz01	DHJPAR0021064	Costa Rica	–	JF792897
	* cantherius *	MRS777	Sweden	GBMIN74555-17	KU682249
	* carbonarius *	MRS162	Hungary	ASQBR120-09	MK585853
		MRS163	Hungary	ASQAS220-11	JF962848
		MRS164	Hungary	ASQBR121-09	MK585851
	* carminatus *	MRS055	France	ASQBR152-09	JF962818
	* castaneus *	BCLDQ0245	Thailand	ASQSP215-08	JQ388461
	* caudalis *	MRS693	France	ASQSP1001-10	HQ551216
	* circumscriptus *	MRS073	UK	–	KU682256
	* compressor *	MRS170	UK	GBAH4098-09	EU979580
	* concoronarius *	BCLDQ01515	Thailand	ASQSR079-11	JN278271
	* coriaceus *	MRS311	Sweden	ASQBR140-09	JF962853
		MRS377	Sweden	ASQBR141-09	MK585885
	*coronopus*	BCLDQ00764	Thailand	ASQSQ045-09	JQ388389
	* corrusciput *	BCLDQ01555	Thailand	ASQSR119-11	JN278306
	* coxalis *	MRS606	UK	ASQSP553-08	MK585874
	* cruentus *	MRS558	France	ASQSP119-08	MK585876
		MRS624	Germany	ASQSP989-10	HQ551274
		MRS625	Germany	ASQSP977-10	HQ551263
	* curticornis *	MRS343	Italy	ASQBR105-09	JF962826/ KU682237
	* damus *	BCLDQ00126	Thailand	ASQSP378-08	JQ388354
	* diarsianae *	MRS030	UK	ASQSP006-08	JF962600
	* dissector *	MRS007	Turkey	ASQBR115-09	JF957045
		MRS025	Turkey	ASQBR116-09	MK585881
		MRS145	UK	ASQBR117-09	MK585849
* Aleiodes *	* dissector *	MRS146	UK	GBAH2876-07	EF115471
	* esenbeckii *	MRS500	Spain	ASQBR092-09	JF962845/ KU682240
	* esenbeckii * f. dendrolimi	MRS180	Finland	GBAH4097-09	EU979581
	* flavostriatus *	BCLDQ0628	Thailand	ASQSP849-08	JF962636
	* fortipes *	MRS650	France	ASQSP966-10	HQ551254
		MRS807	Poland	ASQBR957-18	MH272207
	* gasterator *	MRS046	France	ASQAS218-11	EU979582
		MRS048	France	ASQBR118-09	MK585879
		MRS892/ BF002924	Spain	ASQBR119-09	JF962819
	*gastritor* agg spG1	MRS225	UK	ASQBR273-09	MK585878
	*gastritor* agg spG2	MRS017	UK	ASQBR285-09	MK585867
	*gastritor* agg spG3	MRS351	UK	ASQSP101-08	MK585847
	*gastritor* agg spG4	MRS153	UK	ASQBR296-09	MK585887
	*gastritor* agg spG5	MRS208/AL422	UK	ASQBR959-19	MK585859
	* grassator *	MRS215	UK	ASQBR122-09	JF957046
		MRS721	UK	ASQBR960-19	MK585855
		MRS725	UK	ASQBR961-19	MK585886
	* hirtus *	MRS619	UK	ASQSP1000-10	HQ551215
		MRS882/ BCLDQ0505	Romania	ASQSP695-08	MK585888
	* hirtus *	MRS883	Romania	ASQSP694-08	MK585850
	* jakowlewi *	MRS355	Finland	ASQBR123-09	JF962849
	* leptofemur *	MRS156	UK	ASQBR196-09	JF962813
	* melanopterus *	BCLDQ0799	Brazil	ASQSQ080-09	MH272348
	* mellificus *	AL0037	Thailand	ASQBR476-09	JF962707
	* mexicanus *	BMNHE897778	Belize	ASQSQ642-10	HQ551341
	* miniatus *	MRS950	Sweden	ALEIO030-19	MN968689
		MRS951	Sweden	ALEIO031-19	MN968690
	* modestus *	MRS282	UK	ASQBR124-09	JF962850
	* mubfsi *	AL0323	Uganda	GBAH2900-07	EF115447
	* nigriceps *	MRS613	UK	ASQSP769-08	KU682243
	* nigricornis *	MRS216	UK	GBAH4093-09	EU979585
	* nobilis *	MRS401	Finland	ASQBR093-09	MK585856
		MRS880/ BCLDQ0277	Russia	ASQSP247-08	MK585871
		MRS881/ BCLDQ00123	UK	ASQSP375-08	JF962562
	* nunbergi *	MRS723	Austria	ASQBR930-18	MH272254
	* pallidator *	MRS001	Turkey	GBAH4092-09	EU979586
	* pallidicornis *	MRS885	Russia	ASQSP742-08	JF957043
	* pappi *	CCDB-27844-E04	Kenya	BBTH766-17	MH272335
	* paulmarshi *	CK0002	Thailand	ASQSP942-10	HQ551236
	* pictus *	MRS556	Austria	GBMIN74565-17	KU682242
	* praetor *	MRS654	Bulgaria	ASQSP979-10	HQ551265/KU682244
	* probuzurae *	BCLDQ1202	Thailand	ASQSQ410-09	HM435162
	* pulchripes *	MRS847	Sweden	BCNCA197-18	MK585848
		MRS873/ BC-ZSM-HYM-27497-A05	Sweden	BCHYM15694-17	MK585880
	* punctipes *	MRS212	UK	GBAH4091-09	EU979587
	* quadrum *	MRS824	Bulgaria	AAHYM658-16	MK585865
		MRS796	France	ASQBR939-18	MH272232
* Aleiodes *	* reticulatus *	MRS808	Poland	GBAHB1497-18	KU682262
	* risaae *	BCLDQ1268	Thailand	ASQSQ476-09	HM435190
	* rivulus *	BCLDQ01646	Thailand	ASQSR210-11	JN278372
	* ruficornis *	MRS140	UK	ASQBR132-09	MK585892
		MRS877	Sweden	BCNCA221-18	MK585860
		MRS887/ AL0149	UK	ASQBR131-09	MK585846
		MRS888	UK	GBAH2871-07	EF115476
		MRS889	?	ASQBR127-09	EF115475
		MRS890	UK	ASQBR962-19	MK585883
		MRS891	UK	GBAH5262-09	EF115477
	nr ruficornis	MRS886	Hungary	ASQSP806-08	JF962480
	* rufipes *	MRS294	Sweden	ASQBR134-09	MK585884
		MRS312	Sweden	ASQSP069-08	MH272380
		MRS314	Sweden	ASQBR135-09	MK585873
		MRS673/ SwedFin2	Finland	ASQBR137-09	MK585864
		MRS674/ SwedFin5	Finland	ASQBR138-09	MK585854
		MRS676/ SwedFin17	Finland	ASQBR136-09	JF962855
		MRS680/ SwedFin28	Finland	ASQBR139-09	JF962854
	* rugulosus *	MRS191	Hungary	ASQBR142-09	JF962857
		MRS217	UK	ASQBR143-09	JF962856
		MRS398	France	ASQSP011-08	MH272286
		MRS884/ BCLDQ0509	Poland	ASQSP699-08	MK585875
		CollHH1599	Norway	COLHH1790-18	MK585877
	* ryrholmi *	MRS395	Sweden	ASQBR322-09	JF962792
	*seriatus* agg	MRS616	UK	ASQSP754-08	MH272311
	* sibiricus *	MRS310	Sweden	ASQBR157-09	JF962862
		MRS313	Sweden	ASQBR158-09	MH272159
		MRS805	France	BCNCA185-18	MK585861
	* signatus *	MRS844	Austria	BCNCA195-18	MK585889
	* similis *	MRS696	Austria	ASQSQ727-10	HQ551413
	* sophieae *	BCLDQ01065	Thailand	ASQSQ362-09	JQ388368
	sp M3	MRS703/ MRSA 703	Hungary	ASQSR240-11	MK585852
	* spurivena *	BCLDQ00003	Vietnam	–	KY621612
	* terminalis *	BCLDQ0692	USA	ASQSP913-08	JF962663
	* testaceus *	MRS072	UK	ASQBR963-19	MK585866
	* trevelyanae *	AL0226	Uganda	–	EF115433
	* trianguliscleroma *	CCDB27844-E03	Malawi	BBTH765-17	MH272236
	* trisphaeropyx *	BCLDQ01643	Thailand	ASQSR207-11	JQ388329
	* turcicus *	MRS126	Turkey	ASQAS219-11	JF962613
	* ungularis *	MRS604	France	ASQSP757-08	JF962867
	* unipunctator *	MRS211	UK	ASQAS221-11	JF962868
		MRS221	Germany	ASQBR171-09	MK585858
		MRS354	UK	ASQSP035-08	MK585890
		MRS893	UK	BCNCA223-18	MK585891
		CollHH1603	Norway	COLHH1794-18	MK585893
		CollHH1604	Norway	COLHH1795-18	MK585868
		CollHH1605	Norway	COLHH1796-18	MK585869
	* valinus *	BCLDQ0267	Thailand	ASQSP237-08	JF963430
	* varius *	MRS446	Russia	–	HQ551275
* Heterogamus *	* dispar *	MRS066	UK	ASQBR042-09	JF963405
	* excavatus *	MRS717	Sweden	ASQBR935-18	MH272379
	* fasciatipennis *	MRS669	Sweden	ASQBR044-09	MH272347
